# One-Pot Metal-Free Synthesis of 3-CF_3_-1,3-Oxazinopyridines by Reaction of Pyridines with CF_3_CO-Acetylenes

**DOI:** 10.3390/molecules24193594

**Published:** 2019-10-06

**Authors:** Vasiliy M. Muzalevskiy, Zoia A. Sizova, Kseniya V. Belyaeva, Boris A. Trofimov, Valentine G. Nenajdenko

**Affiliations:** 1 M. V. Lomonosov Moscow State University, Department of Chemistry, Leninskie Gory 1, 119991 Moscow, Russiasyzova@mail.ru (Z.A.S.); 2 A. E. Favorsky Irkutsk Institute of Chemistry, Siberian Branch, Russian Academy of Sciences, 1 Favorsky Str., 664033 Irkutsk, Russia; belyaeva@irioch.irk.ru

**Keywords:** pyridine, CF_3_CO-acetylenes, 1,3-oxazines, fluorinated heterocycles

## Abstract

The reaction of pyridines with trifluoroacetylated acetylenes was investigated. It was found that the reaction of various pyridines with two molecules of CF_3_CO-acetylenes proceeds under mild metal-free conditions. As a result, efficient stereoselective synthesis of 3-arylethynyl-3-trifluoromethyl-1,3-oxazinopyridines was elaborated. Target heterocycles can be prepared in up to quantitative yields.

## 1. Introduction

Pyridine motif is the one of the most recognizable frameworks among heterocyclic molecules. A lot of attention has been paid to the chemistry of this class of heterocyclic compounds since the very beginning of its discovery. Nowadays the flow of the articles concerning pyridine is still far from the drying out. The high attractiveness of pyridine chemistry can be explained by high biological activity of pyridine derivatives both naturally occurred and prepared in the lab. Therefore, almost 300 alkaloids, having pyridine moiety (not including derivatives with fused pyridine ring, such as isoquinoline), were listed in “The Dictionary of Alkaloids” [1].

**Figure 1:**
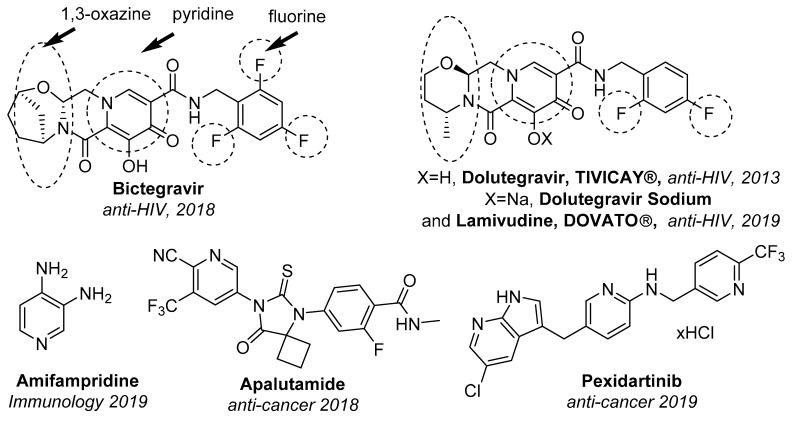
Selected FDA approved drugs in 2018 and 2019 containing pyridine moiety, fluorine atoms, 1,3-oxazine moiety.

The pyridine scaffold is also a privileged structure for design of novel pharmaceuticals. Structural analysis of US FDA approved drugs showed that pyridine core is a consistent part of 62 marketed drugs (second place after piperidine) in the list of most frequent nitrogen heterocycles in structure of approved drugs [2,3]. One can also found 15 derivatives of pyridine among the “Top 200 Pharmaceutical Products by Retail Sales in 2018” which made together about $27 billion during 2018 alone [4]. Some pyridine-based drugs were approved by FDA in 2019 (for examples, see Figure 1).

On the other hand, investigation of organofluorine compounds is one of the most important trends in modern organic chemistry [5,6,7,8,9]. Due to unique physicochemical and biological properties, organofluorine compounds are widely used as construction materials, components of liquid crystalline compositions, agrochemicals and pharmaceuticals [10,11,12,13,14,15]. By some estimation, about 20–25% of currently used drugs [16,17,18,19,20,21,22,23] and agrochemicals [24,25,26,27] contain at least one fluorine atom. As for the year 2018, that value is even higher, because three out of ten drugs approved by the US FDA in 2018 contain this atom (18 out of 59 drugs) [28]. Heterocyclic compounds are also an important object for medicinal chemistry, which can be found among numerous drugs (about 59% of small-molecule drugs [2], approved by the US FDA before 2014). Last year, 35 out of 59 drugs contain any heterocyclic fragment, with 16 of them also having at least one fluorine atom, including six with fluorinated heterocyclic motif (Figure 1). It is not surprising that novel effective methodologies for the synthesis of fluorinated heterocycles have been in great demand in recent decades [29,30,31,32,33,34,35].

**Scheme 1:**
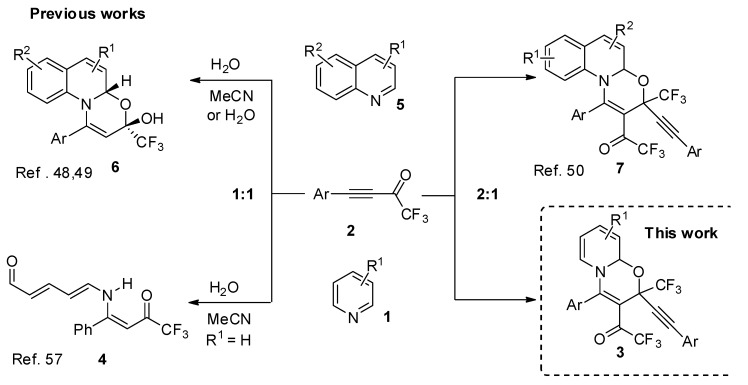
CF_3_-ynones in the reactions with quinolines and pyridines.

α,β-Unsaturated CF_3_-ketones have been shown as versatile building blocks for the synthesis of various fluorinated heterocyclic compounds [36,37,38,39,40]. In a series of works, we have demonstrated a great potential of CF_3_-ynones in different heterocyclizations to prepare fluorinated derivatives of diazepines [41], pyrimidines [42], thiophenes [43], triazoles [44], pyrazoles [45,46,47]. Recently, we focused our attention on the reactions of CF_3_-ynones with azines. It was found that, depending on nature of azine and the acetylene–azine ratio, various products can be obtained very efficiently. The reaction with quinolines opened access to 1,3-oxazinoquinolines **6** [48,49] or **7** (Scheme 1) [50]. 1,3-Oxazine moiety has been experienced a growing interest in recent years [51,52] and became perspective targets for drug design [49,53,54]. For example, Dolutegravir (Tivicay® approved in 2013 [55] and in combination with Lamivudine as Dovato® approved in 2019) and Bictegravir (Biktarvy® approved in 2018) [56] are used for treatment of patients with HIV (Figure 1).

In contrast to the reaction with quinolines, our attempt to involve pyridines into 1,3-oxazine assembling reaction with CF_3_-ynones has been less successful. The reaction of pyridine with equal amount of CF_3_-ynone in wet acetonitrile afforded the corresponding ring opening product. Polyunsaturated 5-amino-2,4-pentadienal **7** has been isolated as a result of cascade transformation (Scheme 1) [57].

## 2. Results and Discussion

This study is devoted to the next step of our systematic study of the reactions of fluorinated acetylenes with azines. A simple and highly efficient approach towards 3-arylethynyl-3-trifluoromethyl-1,3-oxazinopyridines **3** is presented by the reaction of CF_3_-ynones with pyridines in 2:1 ratio.

We assumed that using dry conditions and excess of ketone the reaction course could be redirected to formation of the corresponding trifluoromethylated 1,3-oxazines. Indeed, being mixed together without solvent, pyridine and CF_3_-ynone **2a** 1:2 ratio new transformation was observed to form viscous mass in a few minutes.

**Figure 2:**
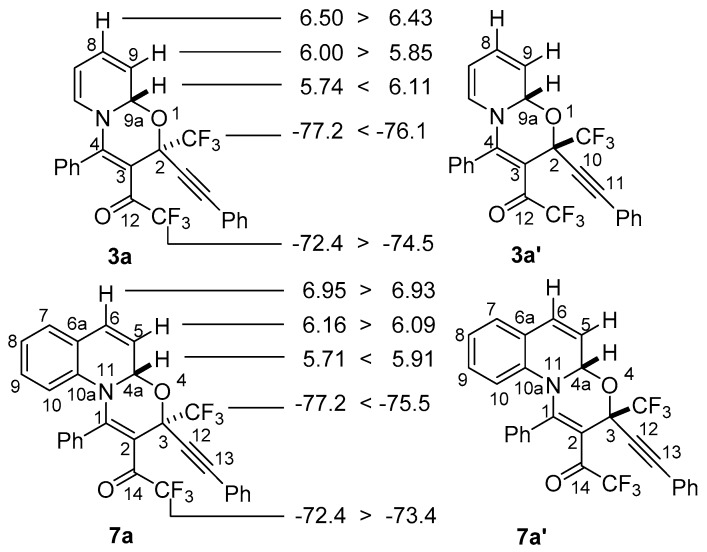
Comparison of characteristic values of chemical shifts of diastereomers of **3a** in ^1^H- and ^19^F-NMR spectra with the corresponding quinoline derivative **7a**.

Analysis of the reaction mixture by NMR showed clean formation of **3a** and unreacted starting materials. After addition of a small amount of MeCN to form homogeneous solution the reaction mixture was left overnight. As a result, oxazine **3a** was isolated in 94% yield in stereoselective manner. According to NMR a 90:10 mixture of 2S*,9aS* and 2R*,9aS* diastereomers was formed. Assignment of both diastereomers was maintained by careful comparison with 3-arylethynyl-3-trifluoromethyl-1,3-oxazinoquinolines **7** having similar structures (Figure 2) [50]. Therefore, values of δ(^1^H-8), δ(^1^H-9), δ(^19^F-COCF_3_) in 2S*,9aS*-**3a** are larger than in 2R*,9aS*-**3a′** while values of δ(^1^H-9a) and δ(^19^F-CF_3_) are the other way around. The same regularity can be seen in the NMR of 3S*,4aS*- and 3R*,4aS*-diastereomers of **7a**.

**Scheme 2:**
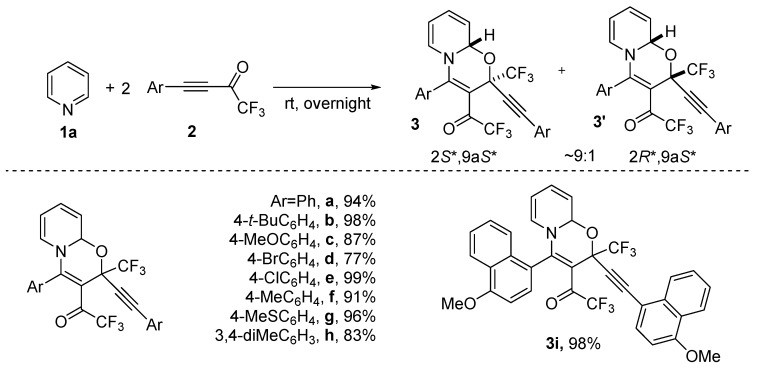
Reactions of pyridine with CF_3_-ynones **2a-i** to form 1,3-oxazinopyridines **3a**–**i.**

Next, the reaction scope was studied. For this aim, the interaction of parent pyridine with various CF_3_-ynones was investigated. To our delight, it was found that the reaction has no restrictions in terms of CF_3_-ynones. The corresponding 1,3-oxazinoquinolines **3a**–**i** were isolated in 77–99% yield (Scheme 2). Similar stereoselectivity was observed for all these products. Compounds **3a**–**i** were formed as a mixture of 2S*,9aS* and 2R*,9aS* diastereomers in near 9:1 ratio in most cases.

**Scheme 3:**
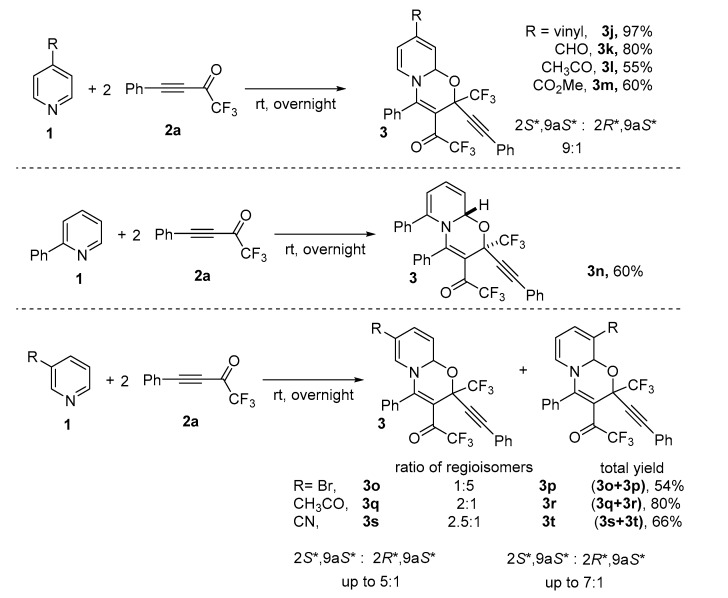
Reactions of pyridine **1b**–**j** with CF_3_-ynones **2a** to form 1,3-oxazinopyridines **3j**–**t.**

Next, the reaction of CF_3_-ynone **2a** with several pyridines was studied in order to investigate the influence of nature of pyridine component of the reaction. A series of 4, 3 and 2-substituted pyridines was involved into reaction with **2a** (Scheme 3).

It was found that the reaction has broad scope in terms of pyridines and nature of substituents. However, the reaction is very sensitive to structure of starting pyridine. An especially important influence on the reaction is the nature of a substituent, pKa value of pyridine and its nucleophilicity, and the position of a substituent in the molecule of pyridine. Therefore, the reaction with 4-substituted pyridines afforded the corresponding oxazines **3j**–**m** in high yields. Again, a mixture of diastereomers in near to 8:1–9:1 ratio was formed in all cases (Scheme 3, compounds **3j**–**m**). In contrast, 2-substituted pyridines (2-phenylpyridine) reacted with CF_3_-ynone **2a** 100% stereoselectively to form 2S*, 9aS* diastereomer exclusively (Scheme 3, compound **3n**). A more complex picture was observed for the reaction with 3-substituted pyridines. Due to the presence of two possible positions for cyclization in the pyridine framework, 7- and 9-isomers were formed in about 2:1 ratio for pyridines with electron-withdrawing acetyl- and cyano groups, having strong −M effect. In contrast mostly 9-isomer (in ratio 5:1 with 7-isomer) was formed in the reaction with 3-bromopyridine having bromine atom with slight +M effect (Scheme 3, compounds **3o–t**). It is noteworthy that both increase (30 °C) and decrease (7 °C) of the temperature did not change dramatically the regioselectivity of the reaction. However, the stereoselectivity of formation of compounds **3p**–**t** was again high to give 2S*,9a*S*-isomer as a major one in up to 7:1 ratio with minor 2R*,9aS*-isomer.

Some restrictions were also found. We observed that pKa of azine and therefore its nucleophilicity plays a decisive role in the possibility of the reaction to occur. Therefore, pyridines with pKa lower than ~1, 2-bromopyridine (0.79), 2-fluoropyridine (−0.43), 2-methoxy-5-bromopyridine (1.04) do not react with CF_3_-ynone **2a**.

**Scheme 4:**
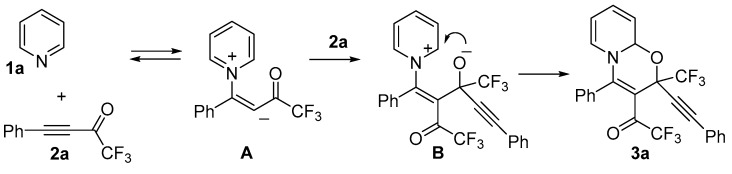
Possible mechanism of the reaction of pyridines with CF_3_-ynones.

Based on our previous mechanistic rationalizations regarding interaction of CF_3_-ynones with azines [48,49,58,59], the possible reaction mechanism can be proposed. The domino assembly of oxazinoquinolines **3** is initiated by the reversible formation of the intermediate zwitterion **A** resulted from the nitrogen nucleophilic addition to the triple bond. In contrast to 1:1 reaction, the carbanionic site of **A** is selectively attacked by the carbonyl group of the second molecule of **2a** to form anion **B**. Cyclization of **B** undergoes by the attacks of oxygen into alpha-position of the pyridine ring to give the corresponding 1,3-oxazine **3** (Scheme 4).

## 3. Materials and Methods

### 3.1. General Details

^1^H-, ^13^C- and ^19^F-NMR spectra were recorded on Bruker AVANCE 400 MHz spectrometer (Bruker Corp., Karlsruhe, Germany) in CD_3_CN and CDCl_3_ at 400.1, 100.6 and 376.3 MHz respectively (Supplementary Information). Chemical shifts (*δ*) in ppm were reported with the use of the residual CHD_2_CN and chloroform signals (1.94 and 7.25 for ^1^H and 77.0 for ^13^C) as internal reference. The ^19^F chemical shifts were referenced to C_6_F_6_, (−162.9 ppm). HRMS (ESI-TOF) spectra were measured with an Orbitrap Elite instrument (TermoFisher, Paisley, UK). TLC analysis was performed on “Merck 60 F_254_” plates. Visualization was accomplished by UV light (254 nm) at Vilber Lourmat UV lamp. Silica gel (silica 60, 0.063–0.2 mm, 70–230 mesh), Screw neck vials (clear, flat bottom, 4 mL) and Screw caps were purchased at MACHEREY-NAGEL (Duren, Germany). All reagents were purchased at Sigma-Aldrich (Muenchen, Germany) and Acros companies (Geel, Belgium). The reagents were of reagent grade and were used as such or distilled prior to use. CF_3_-ynones **2** were prepared as reported previously [46]. Melting points were determined on an Electrothermal 9100 apparatus.

### 3.2. Reaction of CF_3_-Ynones and Pyridines (General Procedure)

A 4 mL vial with a screw cap was charged with CF_3_-ynone **2** (1–1.05 mmol, 2–2.1 equiv.)* and then pyridine **1** (0.5 mmol, 1 equiv.) was added in one portion. After vigorous stirring for several minutes the reaction mixture became viscous due to crystallization of the product. At that moment MeCN (0.5 mL) was added to form homogeneous solution again and the reaction mixture was left overnight at stirring. Next volatiles were evaporated in vacuo, the residue was crystallized from appropriate amount of ether-hexane mixtures or purified via column chromatography on silica gel using mixtures of hexane with CH_2_Cl_2_. * In case of solid CF_3_-ynones **2** MeCN (0.1–0.2 mL) was added to form clear solution.

*2,2,2-Trifluoro-1-(4-phenyl-2-(phenylethynyl)-2-(trifluoromethyl)-2H,9aH-pyrido[2,1-b][1,3]oxazin-3-yl)ethan-1-one* (**3a**). Obtained from pyridine **1a** (0.042 g, 0.53 mmol) and CF_3_-ynone **2a** (0.212 g, 1.071 mmol). Yellow-brown powder, m.p. 109.4–111.8 °C (hexane), yield 0.238 g (94%). (2S*,9aS*):(2R*,9aS*)-isomers ratio is 90:10 (^19^F-NMR). HRMS (ESI-TOF): *m*/*z* [M + H]^+^ Calcd for C_25_H_16_F_6_NO_2_^+^: 476.1080; found: 476.1085.

(2S*,9aS*)-**3a**: ^1^H-NMR (400.1 MHz, CDCl_3_): δ 7.68–7.39 (m, 7H), 7.37–7.27 (m, 3H), 6.50 (dd, ^3^*J*_8,9_ = 9.7 Hz, ^3^*J*_7,8_ = 6.1 Hz, 1H, H-8), 6.46 (d, ^3^*J*_6,7_ = 7.7 Hz, 1H, H-6), 6.00 (dd, ^3^*J*_8,9_ = 9.7 Hz, ^3^*J*_9a,9_ = 3.9 Hz, 1H, H-9), 5.74 (d, ^3^*J*_9a,9_ = 3.9 Hz, 1H, H-9a), 5.50 (pseudo-td, ^3^*J* ~ 7 Hz, ^4^*J* ~ 1 Hz, 1H, H-7). ^13^C-NMR (100.6 MHz, CDCl_3_): δ 180.7 (q, ^2^*J*_CF_ = 35.0 Hz, C-12), 160.3 (C-4), 133.2, 132.1, 131.4 (C*_i′_* from Ar), 129.5, 129.3, 129.2, 128.3 (C-8), 126.2 (C-6), 125.8, 122.6 (q, ^1^*J*_CF_ = 286.6 Hz, CF_3_), 121.1 (C*_i_* from Ar), 116.5 (C-9), 115.5 [q, ^1^*J*_CF_ = 292.7 Hz, C(O)CF_3_], 104.0 (C-7), 88.4 (C-11), 81.3 (C-10), 79.1 (C-9a), 73.7 (q, ^2^*J*_CF_ = 33.9 Hz, C-2). ^19^F-NMR (376.3 MHz, CDCl_3_): δ −72.5 [C(O)CF_3_], −77.3 (CF_3_).

(2R*,9aS*)-**3a′**: ^1^H-NMR (400.1 MHz, CDCl_3_): δ 6.43 (dd, ^3^*J*_8,9_ = 9.8 Hz, ^3^*J*_7,8_ = 6.1 Hz, 1H, H-8), 6.28 (d, ^3^*J*_6,7_ = 7.6 Hz, 1H, H-6), 6.11 (d, ^3^*J*_9a,9_ = 4.0 Hz, 1H, H-9a), 5.85 (dd, ^3^*J*_8,9_ = 9.8 Hz, ^3^*J*_9a,9_ = 4.0 Hz, 1H, H-9), 5.34 (pseudo-td, ^3^*J* ~ 7 Hz, ^4^*J* = 1 Hz, 1H, H-7). Other signals are overlapped with those of major isomer. ^13^C-NMR (100.6 MHz, CDCl_3_): δ 133.9, 132.3, 128.9, 126.5, 126.3, 115.0, 109.4, 102.2. Other signals are overlapped with those of major isomer or cannot be seen in the spectrum due to the low concentration of minor isomer. ^19^F-NMR (376.3 MHz, CDCl_3_): δ −74.6 [C(O)CF_3_], −76.2 (CF_3_).

*1-(4-(4-(Tert-butyl)phenyl)-2-((4-(tert-butyl)phenyl)ethynyl)-2-(trifluoromethyl)-2H,9aH-pyrido[2,1-b][1,3]oxazin-3-yl)-2,2,2-trifluoroethan-1-one* (**3b**). Obtained from pyridine **1a** (0.041 g, 0.518 mmol) and CF_3_-ynone **2b** (0.267 g, 1.051 mmol). Yellow powder, m.p. 130.0–132.7 °C (hexane), yield 0.300 g (98%). (2S*,9aS*):(2R*,9aS*)-isomers ratio is 90:10 (^19^F-NMR). HRMS (ESI-TOF): *m/z* [M + H]^+^ Calcd for C_33_H_32_F_6_NO_2_^+^: 588.2332; found: 588.2340.

(2S*,9aS*)-**3b**: ^1^H-NMR (400.1 MHz, CDCl_3_): δ 7.52 (d, ^3^*J* = 8.4 Hz, 2H), 7.47–7.36 (m, 4H), 7.32 (d, ^3^*J* = 8.4 Hz, 2H), 6.52 (d, ^3^*J*_6,7_ = 7.8 Hz, 1H, H-6), 6.48 (dd, ^3^*J*_8,9_ = 9.8 Hz, ^3^*J*_7,8_ = 6.1 Hz, 1H, H-8), 5.99 (dd, ^3^*J*_8,9_ = 9.8 Hz, ^3^*J*_9a,9_ = 3.9 Hz, 1H, H-9), 5.72 (d, ^3^*J*_9a,9_ = 3.9 Hz, 1H, H-9a), 5.49 (psedo-t, ^3^*J* ~ 7 Hz, 1H, H-7), 1.35 (s, 9H, 3Me from *t*-Bu), 1.29 (s, 9H, 3Me from *t*-Bu). ^13^C-NMR (100.6 MHz, CDCl_3_): δ 180.8 (q, ^2^*J*_CF_ = 35.0 Hz, C-12), 160.4 (C-4), 157.2 (C*_p_* from Ar), 152.7 (C*_p′_* from Ar), 131.9 (C*_m,m′_* from Ph), 128.6 (q, ^3^*J*_CF_ = 2.2 Hz, C-3), 126.4, 126.3 (C-8), 126.1 (C-6), 125.3 (C*_o,o′_* from Ar), 122.6 (q, ^1^*J*_CF_ = 286.6 Hz, CF_3_), 118.1 (C*_i′_* from Ar), 116.5 (C-9), 115.7 [q, ^1^*J*_CF_ = 292.5 Hz, C(O)CF_3_], 103.6 (C-7), 88.5 (C-11), 80.9 (C-10), 79.0 (C-9a), 73.8 (q, ^2^*J*_CF_ = 34.6Hz, C-2), 35.2, 34.8, 31.1, 31.0. ^19^F-NMR (376.3 MHz, CDCl_3_): δ −72.5 [C(O)CF_3_], −77.4 (CF_3_).

(2R*,9aS*)-**3b′**: ^1^H-NMR (400.1 MHz, CDCl_3_): δ 6.43 (dd, ^3^*J*_8,9_ = 9.8 Hz, ^3^*J*_7,8_ = 6.0 Hz, 1H, H-8), 6.33 (d, ^3^*J*_6,7_ = 7.6 Hz, 1H, H-6), 6.10 (d, ^3^*J*_9a,9_ = 3.9 Hz, 1H, H-9a), 5.84 (dd, ^3^*J*_8,9_ = 9.8 Hz, ^3^*J*_9a,9_ = 3.9 Hz, 1H, H-9), 5.33 (pseudo-t, ^3^*J* ~ 7 Hz, 1H, H-7), 1.33 (s, 9H, 3Me from *t*-Bu), 1.30 (s, 9H, 3Me from *t*-Bu). Other signals are overlapped with those of major isomer. ^13^C-NMR (100.6 MHz, CDCl_3_): δ 132.0, 126.6, 126.51, 126.49, 125.2, 109.2, 35.1, 31.2, 30.9. Other signals are overlapped with those of major isomer or cannot be seen in the spectrum due to the low concentration of minor isomer. ^19^F-NMR (376.3 MHz, CDCl_3_): δ −74.6 [C(O)CF_3_], −76.2 (CF_3_).

*1-(4-(4-Methoxyphenyl)-2-((4-methoxyphenyl)ethynyl)-2-(trifluoromethyl)-2H,9aH-pyrido[2,1-b][1,3]oxazin-3-yl)-2,2,2-trifluoroethan-1-one* (**3c**). Obtained from pyridine **1a** (0.041 g, 0.518 mmol) and CF_3_-ynone **2c** (0.239 g, 1.048 mmol). Light brown powder, m.p. 117.3–118.7 °C (hexane), yield 0.242 g (87%). (2S*,9aS*):(2R*,9aS*)-isomers ratio is 90:10 (^19^F-NMR). HRMS (ESI-TOF): *m/z* [M + H]^+^ Calcd for C_27_H_20_F_6_NO_4_^+^: 536.1291; found: 536.1296.

(2S*,9aS*)-**3c**: ^1^H-NMR (400.1 MHz, CDCl_3_): δ 7.48–7.30 (m, 4H), 7.07–6.91 (m, 2H), 6.82 (d, ^3^*J* = 8.9 Hz, 2H), 6.49 (d, ^3^*J*_6,7_ = 7.0 Hz, 1H, H-6), 6.48 (dd, ^3^*J*_8,9_ = 9.8 Hz, ^3^*J*_7,8_ = 6.1 Hz, 1H, H-8), 5.99 (dd, ^3^*J*_8,9_ = 9.8 Hz, ^3^*J*_9a,9_ = 4.1 Hz, 1H, H-9), 5.71 (d, ^3^*J*_9a,9_ = 4.1 Hz, 1H, H-9a), 5.49 (pseudo-td, ^3^*J* ~ 7 Hz, ^3^*J* ~ 1 Hz, 1H, H-7), 3.88 (s, 3H, MeO), 3.79 (s, 3H, MeO). ^13^C-NMR (100.6 MHz, CDCl_3_): δ 180.6 (q, ^2^*J*_CF_ = 34.7 Hz, C-12), 163.7, 160.4 (C-4), 160.3, 133.7, 126.3 (C-8), 126.0 (C-6), 123.7, 122.7 (q, ^1^*J*_CF_ = 286.8 Hz, CF_3_), 116.5 (C-9), 115.7 [q, ^1^*J*_CF_ = 293.0 Hz, C(O)CF_3_], 113.9, 113.2, 108.6, 103.8 (C-7), 88.3 (C-11), 80.3 (C-10), 78.9 (C-9a), 73.9 (q, ^2^*J*_CF_ = 34.3 Hz, C-2), 55.6, 55.2. ^19^F-NMR (376.3 MHz, CDCl_3_): δ −72.4 [C(O)CF_3_], −77.5 (CF_3_).

(2R*,9aS*)-**3c′**: ^1^H-NMR (400.1 MHz, CDCl_3_): δ 7.52 (d, ^3^*J* = 8.9 Hz, 2H), 6.42 (dd, ^3^*J*_8,9_ = 9.8 Hz, ^3^*J*_7,8_ = 6.1 Hz, 1H, H-8), 6.31 (d, ^3^*J*_6,7_ = 7.5 Hz, 1H, H-6), 6.07 (d, ^3^*J*_9a,9_ = 4.1 Hz, 1H, H-9a), 5.84 (dd, ^3^*J*_8,9_ = 9.8 Hz, ^3^*J*_9a,9_ = 4.1 Hz, 1H, H-9), 5.33 (pseudo-t, ^3^*J* ~ 7 Hz, 1H, H-7), 3.86 (s, 3H, MeO), 3.81 (s, 3H, MeO). Other signals are overlapped with those of major isomer. ^13^C-NMR (100.6 MHz, CDCl_3_): δ 136.0, 133.8, 131.8, 126.51, 126.48, 114.4, 55.5. Other signals are overlapped with those of major isomer or cannot be seen in the spectrum due to the low concentration of minor isomer. ^19^F-NMR (376.3 MHz, CDCl_3_): δ −74.6 [C(O)CF_3_], −76.2 (CF_3_).

*1-(4-(4-Bromophenyl)-2-((4-bromophenyl)ethynyl)-2-(trifluoromethyl)-2H,9aH-pyrido[2,1-b][1,3]oxazin-3-yl)-2,2,2-trifluoroethan-1-one* (**3d**). Obtained from pyridine **1a** (0.0395 g, 0.5 mmol) and CF_3_-ynone **2d** (0.292 g, 1.054 mmol). Yellow-brown powder, m.p. 83.9–86.7 °C (hexane), yield 0.244 g (77%). (2S*,9aS*):(2R*,9aS*)-isomers ratio is 89:11 (^19^F-NMR). HRMS (ESI-TOF): *m/z* [M + H]^+^ Calcd for C_25_H_14_Br_2_F_6_NO_2_^+^: 633.9270; found: 633.9282.

(2S*,9aS*)-**3d**: ^1^H-NMR (400.1 MHz, CDCl_3_): δ 7.68–7.29 (m, 8H), 6.49 (dd, ^3^*J*_8,9_ = 9.8 Hz, ^3^*J*_7,8_ = 6.1 Hz, 1H, H-8), 6.41 (d, ^3^*J*_6,7_ = 7.6 Hz, 1H, H-6), 6.00 (dd, ^3^*J*_8,9_ = 9.8 Hz, ^3^*J*_9a,9_ = 3.9 Hz, 1H, H-9), 5.69 (d, ^3^*J*_9a,9_ = 3.9 Hz, 1H, H-9a), 5.53 (pseudo-t, ^3^*J* ~ 7 Hz, 1H, H-7). ^13^C-NMR (100.6 MHz, CDCl_3_): δ 180.4 (q, ^2^*J*_CF_ = 35.4 Hz, C-12), 159.3 (C-4), 149.6, 133.5, 132.9, 131.6, 130.2, 128.5, 126.3 (C-8), 125.4 (C-6), 123.9, 122.4 (q, ^1^*J*_CF_ = 286.8 Hz, CF_3_), 119.9, 116.7 (C-9), 115.5 [q, ^1^*J*_CF_ = 292.7 Hz, C(O)CF_3_], 109.4, 104.5 (C-7), 87.4 (C-11), 82.2 (C-10), 79.2 (C-9a), 73.6 (q, ^2^*J*_CF_ = 34.3 Hz, C-2). ^19^F-NMR (376.3 MHz, CDCl_3_): δ −72.3 [C(O)CF_3_], −77.3 (CF_3_).

(2R*,9aS*)-**3d′**: ^1^H-NMR (400.1 MHz, CDCl_3_): δ 6.45–6.42 (m, 1H, H-8), 6.22 (d, ^3^*J*_6,7_ = 7.5 Hz, 1H, H-6), 6.07 (d, ^3^*J*_9a,9_ = 4.0 Hz, 1H, H-9a), 5.84 (dd, ^3^*J*_8,9_ = 9.8 Hz, ^3^*J*_9a,9_ = 4.0 Hz, 1H, H-9), 5.36 (pseudo-t, ^3^*J* = 6.8 Hz, 1H, H-7). Other signals are overlapped with those of major isomer. ^13^C-NMR (100.6 MHz, CDCl_3_): δ 136.1, 135.1, 133.7, 131.6, 126.5, 126.0, 123.8, 115.1, 102.6. Other signals are overlapped with those of major isomer or cannot be seen in the spectrum due to the low concentration of minor isomer. ^19^F-NMR (376.3 MHz, CDCl_3_): δ −74.5 [C(O)CF_3_], −76.2 (CF_3_).

*1-(4-(4-Chlorophenyl)-2-((4-chlorophenyl)ethynyl)-2-(trifluoromethyl)-2H,9aH-pyrido[2,1-b][1,3]oxazin-3-yl)-2,2,2-trifluoroethan-1-one* (**3e**). Obtained from pyridine **1a** (0.042 g, 0.53 mmol) and CF_3_-ynone **2e** (0.254 g, 1.09 mmol). Yellow-brown powder, m.p. 68–70 °C (hexane), yield 0.286 g (99%). (2S*,9aS*):(2R*,9aS*)-isomers ratio is 89:11 (^19^F-NMR). HRMS (ESI-TOF): *m/z* [M + H]^+^ Calcd for C_25_H_14_Cl_2_F_6_NO_2_^+^: 544.0300; found: 544.0308.

(2S*,9aS*)-**3e**: ^1^H-NMR (400.1 MHz, CDCl_3_): δ 7.52–7.28 (m, 8H), 6.49 (dd, ^3^*J*_8,9_ = 9.8 Hz, ^3^*J*_7,8_ = 6.1 Hz, 1H, H-8), 6.41 (d, ^3^*J*_6,7_ = 7.6 Hz, 1H, H-6), 6.00 (dd, ^3^*J*_8,9_ = 9.8 Hz, ^3^*J*_9a,9_ = 4.0 Hz, 1H, H-9), 5.69 (d, ^3^*J*_9a,9_ = 4.0 Hz, 1H, H-9a), 5.53 (pseudo-t, ^3^*J* ~ 7 Hz, 1H, H-7). ^1^H-NMR (400.1 MHz, CD_3_CN): δ 7.73–7.31 (m, 8H), 6.57–6.52 (m, 2H, H-8, H-6), 6.03 (dd, ^3^*J*_8,9_ = 9.8 Hz, ^3^*J*_9a,9_ = 3.8 Hz, 1H, H-9), 5.75 (d, ^3^*J*_9a,9_ = 3.8 Hz, 1H, H-9a), 5.61 (pseudo-t, ^3^*J* = 7.2 Hz, 1H, H-7). ^13^C-NMR (100.6 MHz, CDCl_3_): δ 180.3 (q, ^2^*J*_CF_ = 34.7 Hz, C-12), 159.2 (C-4), 149.6, 140.0, 135.6, 133.3, 129.9, 128.7, 126.3 (C-8), 125.4 (C-6), 122.4 (q, ^1^*J*_CF_ = 286.8 Hz, CF_3_), 119.4, 116.7 (C-9), 115.5 [q, ^1^*J*_CF_ = 292.7 Hz, C(O)CF_3_], 109.4, 104.5 (C-7), 87.3 (C-11), 82.0 (C-10), 79.1 (C-9a), 73.6 (q, ^2^*J*_CF_ = 34.3 Hz, C-2). ^19^F-NMR (376.3 MHz, CD_3_CN): δ −70.0 [C(O)CF_3_], −75.4 (CF_3_). ^19^F-NMR (376.3 MHz, CDCl_3_): δ −72.2 [C(O)CF_3_], −77.3 (CF_3_).

(2R*,9aS*)-**3e′**: ^1^H-NMR (400.1 MHz, CDCl_3_): δ 6.22 (d, ^3^*J*_6,7_ = 7.6 Hz, 1H, H-6), 6.07 (d, ^3^*J*_9a,9_ = 4.0 Hz, 1H, H-9a), 5.85 (dd, ^3^*J*_8,9_ = 9.8 Hz, ^3^*J*_9a,9_ = 4.0 Hz, 1H, H-9), 5.36 (pseudo-t, ^3^*J* ~ 7 Hz, 1H, H-7). Other signals are overlapped with those of major isomer. ^1^H-NMR (400.1 MHz, CD_3_CN): δ 6.48–6.42 (m, 1H, H-8), 6.32 (d, ^3^*J*_6,7_ = 7.5 Hz, 1H, H-6), 5.89 (dd, ^3^*J*_8,9_ = 9.8 Hz, ^3^*J*_9a,9_ = 3.9 Hz, 1H, H-9), 5.42 (pseudo-t, ^3^*J* = 6.8 Hz, 1H, H-7). Other signals are overlapped with those of major isomer. ^13^C-NMR (100.6 MHz, CDCl_3_): δ 136.1, 133.5, 129.7, 128.6, 126.0, 123.9, 115.1, 102.6. Other signals are overlapped with those of major isomer or cannot be seen in the spectrum due to the low concentration of minor isomer. ^19^F-NMR (376.3 MHz, CD_3_CN): δ −72.2 [C(O)CF_3_], −74.1 (CF_3_). ^19^F-NMR (376.3 MHz, CDCl_3_): δ −74.4 [C(O)CF_3_], −76.1 (CF_3_).

*1-(4-(4-Methylphenyl)-2-((4-methylphenyl)ethynyl)-2-(trifluoromethyl)-2H,9aH-pyrido[2,1-b][1,3]oxazin-3-yl)-2,2,2-trifluoroethan-1-one* (**3f**). Obtained from pyridine **1a** (0.044 g, 0.556 mmol) and CF_3_-ynone **2f** (0.240 g, 1.13 mmol). Yellow-brown powder, m.p. 95.2–99.1 °C (hexane), yield 0.256 g (91%). (2S*,9aS*):(2R*,9aS*)-isomers ratio is 91:9 (^19^F-NMR). HRMS (ESI-TOF): *m/z* [M + H]^+^ Calcd for C_27_H_20_F_6_NO_2_^+^: 504.1393; found: 504.1401.

(2S*,9aS*)-**3f**: ^1^H-NMR (400.1 MHz, CDCl_3_): δ 7.54–7.10 (m, 8H), 6.50–6.47 (m, 2H, H-8, H-6), 6.00 (dd, ^3^*J*_8,9_ = 10.0 Hz, ^3^*J*_9a,9_ = 3.8 Hz, 1H, H-9), 5.74 (d, ^3^*J*_9a,9_ = 3.8 Hz, 1H, H-9a), 5.49 (pseudo-t, ^3^*J* = 6.7 Hz, 1H, H-7), 2.44 (s, 3H, Me), 2.33 (s, 3H, Me). ^13^C-NMR (100.6 MHz, CDCl_3_): δ 180.7 (q, ^2^*J*_CF_ = 35.0 Hz, C-12), 160.5 (C-4), 139.5, 134.0, 132.0, 130.2, 129.0, 128.7, 126.2 (C-8), 125.9 (C-6), 122.6 (q, ^1^*J*_CF_ = 286.8 Hz, CF_3_), 118.0, 116.4 (C-9), 115.6 [q, ^1^*J*_CF_ = 293.4 Hz, C(O)CF_3_], 109.0, 103.8 (C-7), 88.5 (C-11), 80.8 (C-10), 79.0 (C-9a), 73.8 (q, ^2^*J*_CF_ = 34.3 Hz, C-2), 21.6, 21.5. ^19^F-NMR (376.3 MHz, CDCl_3_): δ −72.3 [C(O)CF_3_], −77.2 (CF_3_).

(2R*,9aS*)-**3f′**: ^1^H-NMR (400.1 MHz, CDCl_3_): δ 6.47–6.41 (m, 1H, H-8), 6.30 (d, ^3^*J*_6,7_ = 7.6 Hz, 1H, H-6), 6.10 (d, ^3^*J*_9a,9_ = 3.8 Hz, 1H, H-9a), 5.84 (dd, ^3^*J*_8,9_ = 9.6 Hz, ^3^*J*_9a,9_ = 3.8 Hz, 1H, H-9), 5.33 (pseudo-t, ^3^*J* ~ 7 Hz, 1H, H-7), 2.42 (s, 3H, Me), 2.36 (s, 3H, Me). Other signals are overlapped with those of major isomer. ^13^C-NMR (100.6 MHz, CDCl_3_): δ 143.2, 139.4, 132.1, 126.4, 118.4, 115.0, 102.0, 79.8. Other signals are overlapped with those of major isomer or can not be seen in the spectrum due to the low concentration of minor isomer. ^19^F-NMR (376.3 MHz, CDCl_3_): δ −74.4 [C(O)CF_3_], −76.1 (CF_3_).

*1-(4-(4-Methylthiophenyl)-2-((4-methylthiophenyl)ethynyl)-2-(trifluoromethyl)-2H,9aH-pyrido[2,1-b][1,3]oxazin-3-yl)-2,2,2-trifluoroethan-1-one* (**3g**). Obtained from pyridine **1a** (0.040 g, 0.506 mmol) and CF_3_-ynone **2g** (0.256 g, 1.05 mmol). Brown powder, m.p. 120.5–123.2 °C (hexane), yield 0.274 g (96%). (2S*,9aS*):(2R*,9aS*)-isomers ratio is 92:8 (^19^F-NMR). HRMS (ESI-TOF): *m/z* [M + H]^+^ Calcd for C_27_H_20_F_6_NO_2_S_2_^+^: 568.0834; found: 568.0834.

(2S*,9aS*)-**3g**: ^1^H-NMR (400.1 MHz, CDCl_3_): δ 7.49–7.25 (m, 6H), 7.14 (d, 2H, ^3^*J* = 8.5 Hz), 6.51–6.47 (m, 2H, H-8, H-6), 6.00 (dd, ^3^*J*_8,9_ = 9.7 Hz, ^3^*J*_9a,9_ = 3.8 Hz, 1H, H-9), 5.70 (d, ^3^*J*_9a,9_ = 3.8 Hz, 1H, H-9a), 5.50 (pseudo-t, ^3^*J* = 6.4 Hz, 1H, H-7), 2.53 (s, 3H, Me), 2.46 (s, 3H, Me). ^1^H-NMR (400.1 MHz, CD_3_CN): δ 7.58–7.30 (m, 6H), 7.24 (d, 2H, ^3^*J* = 8.7 Hz), 6.57 (d, ^3^*J*_6,7_ = 7.6 Hz, 1H, H-6), 6.54 (dd, ^3^*J*_8,9_ = 9.8 Hz, ^3^*J*_7,8_ = 6.0 Hz, 1H, H-8), 6.00 (dd, ^3^*J*_8,9_ = 9.8 Hz, ^3^*J*_9a,9_ = 4.0 Hz, 1H, H-9), 5.74 (d, ^3^*J*_9a,9_ = 4.0 Hz, 1H, H-9a), 5.59 (pseudo-t, ^3^*J* = 6.8 Hz, 1H, H-7), 2.53 (s, 3H, Me), 2.47 (s, 3H, Me). ^13^C-NMR (100.6 MHz, CD_3_CN): δ 180.8 (q, ^2^*J*_CF_ = 34.1 Hz, C-12), 162.6 (C-4), 148.1, 142.6, 135.2, 132.9, 131.8, 129.8, 127.1, 126.8 (C-8), 126.5 (C-6), 123.8 (q, ^1^*J*_CF_ = 285.8 Hz, CF_3_), 117.8, 117.5, 116.7 [q, ^1^*J*_CF_ = 292.2 Hz, C(O)CF_3_], 108.7, 105.4 (C-7), 88.4 (C-11), 82.5 (C-10), 80.1 (C-9a), 74.5 (q, ^2^*J*_CF_ = 33.7 Hz, C-2), 15.1, 14.7. ^19^F-NMR (376.3 MHz, CDCl_3_): δ −72.3 [C(O)CF_3_], −77.4 (CF_3_). ^19^F-NMR (376.3 MHz, CD_3_CN): δ −70.0 [C(O)CF_3_], −75.5 (CF_3_).

(2R*,9aS*)-**3g′**: ^1^H-NMR (400.1 MHz, CDCl_3_): δ 6.47–6.41 (m, 1H, H-8), 6.30 (d, ^3^*J*_6,7_ = 7.5 Hz, 1H, H-6), 6.07 (d, ^3^*J*_9a,9_ = 4.0 Hz, 1H, H-9a), 5.84 (dd, ^3^*J*_8,9_ = 9.7 Hz, ^3^*J*_9a,9_ = 4.0 Hz, 1H, H-9), 5.34 (pseudo-t, ^3^*J* = 6.8 Hz, 1H, H-7), 2.51 (s, 3H, Me), 2.47 (s, 3H, Me). Other signals are overlapped with those of major isomer. ^1^H-NMR (400.1 MHz, CD_3_CN): δ 6.45–6.37 (m, 2H, H-8, H-9a), 6.20 (d, ^3^*J*_6,7_ = 7.0 Hz, 1H, H-6), 5.87 (dd, ^3^*J*_8,9_ = 9.8 Hz, ^3^*J*_9a,9_ = 4.0 Hz, 1H, H-9), 5.42 (t, ^3^*J* = 6.4 Hz, 1H, H-7), 2.52 (s, 3H, Me), 2.50 (s, 3H, Me). Other signals are overlapped with those of major isomer. ^13^C-NMR (100.6 MHz, CD_3_CN): δ 145.5, 142.8, 136.9, 133.1, 129.5, 129.0, 128.2, 126.7, 126.3, 104.4, 84.7, 15.1, 14.7. Other signals are overlapped with those of major isomer or cannot be seen in the spectrum due to the low concentration of minor isomer. ^19^F-NMR (376.3 MHz, CDCl_3_): δ −74.4 [s, 3F, C(O)CF_3_], −76.1 (s, 3F, CF_3_). ^19^F-NMR (376.3 MHz, CD_3_CN): δ −72.2 [C(O)CF_3_], −74.0 (CF_3_).

*1-(4-(3,4-Dimethylphenyl)-2-((3,4-dimethylphenyl)ethynyl)-2-(trifluoromethyl)-2H,9aH-pyrido[2,1-b][1,3]oxazin-3-yl)-2,2,2-trifluoroethan-1-one* (**3h**). Obtained from pyridine **1a** (0.039 g, 0.49 mmol) and CF_3_-ynone **2h** (0.232 g, 1.027 mmol). Yellow-brown powder, m.p. 72.6–74.6 °C (hexane), yield 0.215 g (83%). (2S*,9aS*):(2R*,9aS*)-isomers ratio is 92:8 (^19^F-NMR) HRMS (ESI-TOF): *m/z* [M + H]^+^ Calcd for C_29_H_24_F_6_NO_2_^+^: 532.1706; found: 532.1717.

(2S*,9aS*)-**3h**: ^1^H-NMR (400.1 MHz, CDCl_3_): δ 7.38–7.02 (m, 6H), 6.50–6.46 (m, 2H, H-8, H-6), 5.98 (dd, ^3^*J*_8,9_ = 10.0 Hz, ^3^*J*_9a,9_ = 3.9 Hz, 1H, H-9), 5.71 (d, ^3^*J*_9a,9_ = 3.9 Hz, 1H, H-9a), 5.47 (pseudo-t, ^3^*J* = 6.5 Hz, 1H, H-7), 2.34 (s, 3H, Me), 2.31 (s, 3H, Me), 2.24 (s, 3H, Me), 2.21 (s, 3H, Me). ^13^C-NMR (100.6 MHz, CDCl_3_): δ 180.8 (q, ^2^*J*_CF_ = 35.0 Hz, C-12), 160.6 (C-4), 138.3, 136.6, 133.0, 130.6, 129.5, 129.1, 126.2 (C-8), 126.1 (C-6), 122.7 (q, ^1^*J*_CF_ = 286.8 Hz, CF_3_), 118.3, 116.4 (C-9), 115.6 [q, ^1^*J*_CF_ = 293.0 Hz, C(O)CF_3_], 109.0, 103.6 (C-7), 88.6 (C-11), 80.6 (C-10), 78.9 (C-9a), 73.8 (q, ^2^*J*_CF_ = 34.3 Hz, C-2), 20.0, 19.7, 19.6 (br s), 19.4. ^19^F-NMR (376.3 MHz, CDCl_3_): δ −72.3 [C(O)CF_3_], −77.3 (CF_3_).

(2R*,9aS*)-**3h′**: ^1^H-NMR (400.1 MHz, CDCl_3_): δ 6.44–6.40 (m, 1H, H-8), 6.31 (d, ^3^*J*_6,7_ = 7.6 Hz, 1H, H-6), 6.08 (d, ^3^*J*_9a,9_ = 4.0 Hz, 1H, H-9a), 5.83 (dd, ^3^*J*_8,9_ = 9.9 Hz, ^3^*J*_9a,9_ = 4.0 Hz, 1H, H-9), 5.31 (pseudo-t, ^3^*J* = 7.2 Hz, 1H, H-7), 2.32 (s, 3H, Me), 2.28 (s, 3H, Me), 2.25 (s, 3H, Me). Other signals are overlapped with those of major isomer. ^13^C-NMR (100.6 MHz, CDCl_3_): δ 142.7, 138.1, 134.8, 133.2, 131.7, 130.3, 129.7, 126.6, 126.5, 118.7, 114.9, 108.5, 101.8, 79.7, 20.2, 19.8. Other signals are overlapped with those of major isomer or cannot be seen in the spectrum due to the low concentration of minor isomer. ^19^F-NMR (376.3 MHz, CDCl_3_): δ −74.4 [C(O)CF_3_], −77.3 (CF_3_).

*2,2,2-Trifluoro-1-(4-(4-methoxynaphthalen-1-yl)-2-((4-methoxynaphthalen-1-yl)ethynyl)-2-(trifluoromethyl)-2H,9aH-pyrido[2,1-b][1,3]oxazin-3-yl)ethan-1-one* (**3i**). Obtained from pyridine **1a** (0.0405 g, 0.51 mmol) and CF_3_-ynone **2i** (0.296 g, 1.06 mmol). Yellow-brown powder, m.p. 143.5–145.5 °C (hexane), yield 0.320 g (98%). (2S*,9aS*):(2R*,9aS*)-isomers ratio is 94:6. Rotamers ratio is (93:1):(4:2) (^19^F-NMR). HRMS (ESI-TOF): *m/z* [M + H]^+^ Calcd for C_35_H_24_F_6_NO_4_^+^: 636.1604; found: 636.1608.

(2S*,9aS*)-**3i**: ^1^H-NMR (400.1 MHz, CDCl_3_): δ 8.39–8.36 (m, 1H), 8.25–8.22 (m, 2H), 7.70–7.47 (m, 7H), 6.87 (d, ^3^*J* = 8.1 Hz, 1H), 6.75 (d, ^3^*J* = 8.1 Hz, 1H), 6.49 (dd, ^3^*J*_8,9_ = 9.7 Hz, ^3^*J*_7,8_ = 6.1 Hz, 1H, H-8), 6.11–6.02 (m, 3H, H-6, H-9, H-9a), 5.36 (pseudo-t, ^3^*J* = 7.2 Hz, 1H, H-7), 4.08 (s, 3H, MeO), 4.01 (s, 3H, MeO). ^13^C-NMR (100.6 MHz, CDCl_3_): δ 179.9 (q, ^2^*J*_CF_ = 34.7 Hz, C-12), 160.8 (C-4), 160.2, 156.7, 137.4, 134.4, 132.3, 131.7, 128.8, 127.6, 126.3 (C-8), 126.2 (C-6), 125.93, 125.87, 125.8, 125.1, 124.8, 123.1 (q, ^1^*J*_CF_ = 287.1 Hz, CF_3_), 123.1, 122.1, 120.7, 117.0 (C-9), 115.7 [q, ^1^*J*_CF_ = 292.9 Hz, C(O)CF_3_], 111.0, 109.7, 104.0, 103.5, 103.3, 86.7 (C-11), 84.9 (C-10), 78.6 (C-9a), 74.2 (q, ^2^*J*_CF_ = 33.9 Hz, C-2), 55.9, 55.6. ^19^F-NMR (376.3 MHz, CDCl_3_): δ major rotamer −71.6 [C(O)CF_3_], −76.8 (CF_3_); minor rotamer −73.2 [C(O)CF_3_], −78.1 (CF_3_).

(2R*,9aS*)-**3i′**: ^1^H-NMR (400.1 MHz, CDCl_3_): δ 8.32 (d, ^3^*J* = 8.4 Hz, 1H), 7.96 (d, ^3^*J* = 8.2 Hz, 1H), 6.86 (d, ^3^*J* = 8.1 Hz, 1H), 6.81 (d, ^3^*J* = 7.9 Hz, 1H), 4.04 (s, 3H, MeO), 4.02 (s, 3H, MeO). Other signals are overlapped with those of major isomer. ^13^C-NMR (100.6 MHz, CDCl_3_): δ 137.5, 135.0, 129.0, 126.6, 125.0, 122.9, 56.0, 55.8. Other signals are overlapped with those of major isomer or cannot be seen in the spectrum due to the low concentration of minor isomer. ^19^F-NMR (376.3 MHz, CDCl_3_): δ major rotamer −74.1 [C(O)CF_3_], −75.9 (CF_3_); minor rotamer −74.3 [C(O)CF_3_], −76.2 (CF_3_).

*2,2,2-Trifluoro-1-(4-phenyl-2-(phenylethynyl)-2-(trifluoromethyl)-8-vinyl-2H,9aH-pyrido[2,1-b][1,3]oxazin-3-yl)ethan-1-one* (**3j**). Obtained from pyridine **1b** (0.054 g, 0.51 mmol) and CF_3_-ynone **2a** (0.206 g, 1.04 mmol). Brown powder, m.p. 80–83 °C (hexane), yield 0.249 g (97%). (2S*,9aS*):(2R*,9aS*)-isomers ratio is 89:11 (^19^F-NMR). HRMS (ESI-TOF): *m*/*z* [M + H]^+^ Calcd for C_27_H_18_F_6_NO_2_^+^: 502.1246; found: 502.1246.

(2S*,9aS*)-**3j**: ^1^H-NMR (400.1 MHz, CDCl_3_): δ 7.67–7.44 (m, 7H), 7.37–7.28 (m, 3H), 6.49 (d, ^3^*J*_6,7_ = 8.0 Hz, 1H, H-6), 6.44 (dd, ^3^*J* = 17.6 Hz, ^3^*J* = 11.0 Hz, 1H, CH=CH_2_), 5.90 (d, ^3^*J*_9a,9_ = 4.5 Hz, 1H, H-9), 5.78 (d, ^3^*J*_9a,9_ = 4.5 Hz, 1H H-9a), 5.77 (dd, ^3^*J_6,7_* = 8.0 Hz, ^4^*J_7,9_* = 1.5 Hz, 1H, H-7), 5.56 (d, ^3^*J* = 17.6 Hz, 1H, CH=CH_2_), 5.33 (d, ^3^*J* = 11.0 Hz, 1H, CH_2_, CH=CH_2_). ^13^C-NMR (100.6 MHz, CDCl_3_): δ 180.9 (q, ^2^*J*_CF_ = 34.8 Hz, C-12), 160.2 (C-4), 135.2 (C, CH=CH_2_), 134.3, 133.3, 132.1 (C*_m,m_^,^*from Ar), 131.4 (q, ^1^*J*_CF_ = 1.7 Hz, C-3), 129.5 (br s), 129.3, 128.2 (C*_o,o_^,^*from Ar), 126.0 (C-6), 122.6, (q, ^1^*J*_CF_ = 286.9 Hz, CF_3_), 121.0, 116.6 (CH=CH_2_), 114.3 (CH=CH_2_), 115.5 [q, ^1^*J*_CF_ = 292.8 Hz, C(O)CF_3_], 109.3, 101.7 (C-7), 88.4 (C-11), 81.3 (C-10), 79.4 (C-9a), 73.9 (q, ^2^*J*_CF_ = 34.1 Hz, C-2). ^19^F-NMR (376.3 MHz, CDCl_3_): δ −72.5 [C(O)CF_3_], −77.2 (CF_3_).

(2R*,9aS*)-**3j′**: ^1^H-NMR (400.1 MHz, CDCl_3_): δ 6.31 (d, ^3^*J*_6,7_ = 7.9 Hz, 1H, H-6), 6.13–6.11 (m, 2H, H-9, H-9a), 5.60 (dd, ^3^*J* = 7.9 Hz, ^3^*J* = 1.6 Hz, 1H, H-7), 5.53 (d, ^3^*J* = 17.4 Hz, 1H, CH=CH_2_), 5.51 (d, ^3^*J* = 17.2 Hz, 1H, CH=CH_2_), 5.31 (d, ^3^*J* = 10.0 Hz, 1H, CH=CH_2_). Other signals are overlapped with those of major isomer. ^13^C-NMR (100.6 MHz, CDCl_3_): δ 137.1, 135.4, 132.4, 132.2, 129.2, 128.7, 128.2, 127.2, 126.6, 126.0, 117.6, 116.5, 113.0, 99.8. Other signals are overlapped with those of major isomer or can not be seen in the spectrum due to the low concentration of minor isomer. ^19^F-NMR (376.3 MHz, CDCl_3_): δ −74.6 [C(O)CF_3_], −76.2 (CF_3_).

*4-Phenyl-2-(phenylethynyl)-3-(trifluoroacetyl)-2-(trifluoromethyl)-2H,9aH-pyrido[2,1-b][1,3]oxazine-8-carbaldehyde* (**3k**). Obtained from pyridine **1c** (0.0475 g, 0.44 mmol) and CF_3_-ynone **2a** (0.198 g, 1 mmol). Yellow powder, m.p. 77–79 °C (hexane), yield 0.178 g (80%). (2S*,9aS*):(2R*,9aS*)-isomers ratio is 89:11 (^19^F-NMR). HRMS (ESI-TOF): *m*/*z* [M + H]^+^ Calcd for C_26_H_16_F_6_NO_3_^+^: 504.1029; found: 504.1035.

(2S*,9aS*)-**3k**: ^1^H-NMR (400.1 MHz, CD_3_CN): 9.70 (s, 1H, CHO), δ 7.75–7.25 (m, 10H), 6.81 (d, ^3^*J* = 4.2 Hz, 1H, H-9), 6.66 (d, ^3^*J* = 7.8 Hz, 1H, H-6), 6.06 (d, ^3^*J* = 4.2 Hz, 1H, H-9a), 5.94 (dd, ^3^*J* = 7.8 Hz, ^3^*J* = 1.5 Hz, 1H, H-7). ^13^C-NMR (100.6 MHz, CD_3_CN): δ 192.2 (CHO), 181.7 (q, ^2^*J*_CF_ = 34.8 Hz, C-12), 162.0 (C-4), 137.5, 134.9, 134.7, 134.0, 132.2 (q, ^3^*J*_CF_ = 1.8 Hz), 130.9, 130.6 (br s), 130.2, 129.8, 129.6, 128.7, 123.7 (q, ^1^*J*_CF_ = 286.0 Hz, CF_3_), 121.5, 116.5 [q, ^1^*J*_CF_ = 292.5 Hz, C(O)CF_3_], 110.1, 99.0, 89.5 (C-11), 82.1 (C-10), 80.1 (C-9a), 75.2 (q, ^2^*J*_CF_ = 34.3 Hz, C-2). ^19^F-NMR (376.3 MHz, CD_3_CN): δ −70.2 [C(O)CF_3_], −75.2 (CF_3_). ^19^F-NMR (376.3 MHz, CDCl_3_): δ −71.7 [C(O)CF_3_], −76.0 (CF_3_).

(2S*,9aS*)-**3k′**: ^1^H-NMR (400.1 MHz, CD_3_CN): 9.66 (s, 1H, CHO), 6.52–6.46 (m, 2H), 6.37 (d, ^3^*J* = 4.2 Hz, 1H), 5.77 (dd, ^3^*J* = 7.7 Hz, ^3^*J* = 1.5 Hz, 1H). Other signals are overlapped with those of major isomer. ^13^C-NMR (100.6 MHz, CD_3_CN): δ 133.8, 132.9, 130.0, 129.7, 129.6, 129.4, 128.1, 115.1, 97.1, 83.8, 78.2. Other signals are overlapped with those of major isomer or cannot be seen in the spectrum due to the low concentration of minor isomer. ^19^F-NMR (376.3 MHz, CD_3_CN): δ −72.6 [C(O)CF_3_], −74.4 (CF_3_). ^19^F-NMR (376.3 MHz, CDCl_3_): δ −73.6 [C(O)CF_3_], −75.2 (CF_3_).

*1-(8-Acetyl-4-phenyl-2-(phenylethynyl)-2-(trifluoromethyl)-2H,9aH-pyrido[2,1-b][1,3]oxazin-3-yl)-2,2,2-trifluoroethan-1-one* (**3l**). Obtained from pyridine **1d** (0.030 g, 0.25 mmol) and CF_3_-ynone **2a** (0.101 g, 0.51 mmol). Yellow powder, m.p. 122.8–124.2 °C (hexane), yield 0.071 g (55%). (2S*,9aS*):(2R*,9aS*)-isomers ratio is 87:13 (^19^F-NMR). HRMS (ESI-TOF): *m*/*z* [M + H]^+^ Calcd for C_27_H_18_F_6_NO_3_^+^: 518.1185; found: 518.1214.

(2S*,9aS*)-**3l**: ^1^H-NMR (400.1 MHz, CDCl_3_): δ 7.66–7.45 (m, 7H), 7.38–7.29 (m, 3H), 6.72 (pseudo-d, ^3^*J*_9a,9_ ~ 4 Hz, 1H, H-9), 6.54 (d, ^3^*J*_6,7_ = 7.8 Hz, 1H, H-6), 6.04 (dd, ^3^*J*_6,7_ = 7.8, Hz, ^3^*J*_7,9_ = 1.6 Hz, 1H, H-7), 5.91 (d, ^3^*J*_9a,9_ = 4.2 Hz, 1H, H-9a), 2.48 (s, 3H, Me). ^13^C-NMR (100.6 MHz, CDCl_3_): δ 195.6, 180.9 (q, ^2^*J*_CF_ = 35.4 Hz, C-12), 159.3 (C-4), 136.5, 133.4, 132.1, 131.1 (q, ^1^*J*_CF_ = 1.7 Hz, C-3), 129.6 (br s), 129.5, 128.3 (C*_o,o_^,^*from Ar), 126.7 (C-6), 122.4 (q, ^1^*J*_CF_ = 286.6 Hz, CF_3_), 121.2 (C-9), 120.8 (C*_i_* from Ar), 115.4 [q, ^1^*J*_CF_ = 292.8 Hz, C(O)CF_3_], 110.0, 100.3 (C-7), 89.1 (C-11), 80.9 (C-10), 78.8 (C-9a), 74.4 (q, ^2^*J*_CF_ = 34.1 Hz, C-2), 25.3. ^19^F-NMR (376.3 MHz, CDCl_3_): δ −72.7 [C(O)CF_3_], −77.2 (CF_3_).

(2R*,9aS*)-**3l′**: ^1^H-NMR (400.1 MHz, CDCl_3_): δ 6.57 (pseudo-d, ^3^*J* ~ 4 Hz, 1H, H-9), 6.37 (d, ^3^*J*_6,7_ = 7.8 Hz, 1H, H-6), 6.28 (d, ^3^*J*_9a,9_ = 4.3 Hz, 1H, H-9a), 5.88 (dd, ^3^*J*_6,7_ = 7.8 Hz, ^3^*J*_7,9_ = 1.5 Hz, 1H, H-7), 2.46 (s, 3H, Me). Other signals are overlapped with those of major isomer. ^13^C-NMR (100.6 MHz, CDCl_3_): δ 136.6, 132.6, 132.3, 129.4, 128.3, 127.3, 119.9, 98.5, 29.7. Other signals are overlapped with those of major isomer or cannot be seen in the spectrum due to the low concentration of minor isomer. ^19^F-NMR (376.3 MHz, CDCl_3_): δ −74.7 [C(O)CF_3_], −76.3 (CF_3_).

*Methyl 4-phenyl-2-(phenylethynyl)-3-(2,2,2-trifluoroacetyl)-2-(trifluoromethyl)-2H,9aH- pyrido[2,1-b][1,3]oxazine-8-carboxylate* (**3m**). Obtained from pyridine **1e** (0.048 g, 0.35 mmol) and CF_3_-ynone **2a** (0.147 g, 0.74 mmol). Pale yellow powder, m.p. 115.4–116.5 °C (hexane), yield 0.112 g (60%). (2S*,9aS*):(2R*,9aS*)-isomers ratio is 87:13 (^19^F-NMR). HRMS (ESI-TOF): *m*/*z* [M + H]^+^ Calcd for C_27_H_18_F_6_NO_4_^+^: 534.1135; found: 534.1140.

(2S*,9aS*)-**3m**: ^1^H-NMR (400.1 MHz, CDCl_3_): δ 7.66–7.44 (m, 7H), 7.37–7.29 (m, 3H), 6.89 (pseudo-d, ^3^*J* ~ 4 Hz, 1H, H-9), 6.52 (d, ^3^*J*_6,7_ = 7.8 Hz, 1H, H-6), 5.99 (dd, ^3^*J*_6,7_ = 7.8 Hz, ^3^*J*_7,9_ = 1.5 Hz, 1H, H-7), 5.87 (d, ^3^*J*_9a,9_ = 4.2 Hz, 1H, H-9a), 3.85 (s, 3H, Me). ^13^C-NMR (100.6 MHz, CDCl_3_): δ 181.0 (q, ^2^*J*_CF_ = 35.4 Hz, C-12), 164.6 (C-4), 159.2 (CO_2_Me), 133.4, 132.1, 131.2 (q, ^1^*J*_CF_ = 1.7 Hz, C-3), 130.1, 129.6 (br s), 129.5 (C-8), 128.3 (C*_o,o_^,^*from Ar), 126.5 (C-6), 122.4, (q, ^1^*J*_CF_ = 287.3 Hz, CF_3_), 121.5 (C-9), 120.8 (C*_i_* from Ar), 115.4 (q, ^1^*J*_CF_ = 293.0 Hz, C(O)CF_3_), 110.3, 101.5 (C-7), 89.0 (C-11), 80.9 (C-10), 78.8 (C-9a), 74.3 (q, ^2^*J*_CF_ = 34.8 Hz, C-2), 52.5. ^19^F-NMR (376.3 MHz, CDCl_3_): δ −72.7 [C(O)CF_3_], −77.2 (CF_3_).

(2R*,9aS*)-**3m′**: ^1^H-NMR (400.1 MHz, CDCl_3_): δ 6.74 (pseudo-d, ^3^*J* ~ 4 Hz, 1H, H-9), 6.35 (d, ^3^*J*_6,7_ = 7.8 Hz, 1H, H-6), 6.24 (d, ^3^*J*_9a,9_ = 4.3 Hz, 1H, H-9a), 5.83 (dd, ^3^*J*_6,7_ = 7.8 Hz, ^3^*J*_7,9_ = 1.5 Hz, 1H, H-7), 3.84 (s, 3H, Me). Other signals are overlapped with those of major isomer. ^13^C-NMR (100.6 MHz, CDCl_3_): δ 132.6, 132.3, 130.3, 129.4, 128.3, 127.1, 120.1, 99.8, 52.5. Other signals are overlapped with those of major isomer or cannot be seen in the spectrum due to the low concentration of minor isomer. ^19^F-NMR (376.3 MHz, CDCl_3_): δ −74.7 [C(O)CF_3_], −76.4 (CF_3_).

(2S*,9aS*)-1-(4,6-diPhenyl-2-(phenylethynyl)-2-(trifluoromethyl)-2H,9aH-pyrido[2,1-b][1,3]oxazin-3-yl)-2,2,2-trifluoroethan-1-one (**3n**). Obtained from pyridine **1f** (0.079 g, 0.51 mmol) and CF_3_-ynone **2a** (0.204 g, 1.03 mmol). Orange powder, m.p. 90–91 °C (hexane), yield 0.168 g (60%). HRMS (ESI-TOF): *m/z* [M + H]^+^ Calcd for C_31_H_20_F_6_NO_2_^+^: 552.1393; found: 552.1393. (2S*,9aS*)-**3o**: ^1^H-NMR (400.1 MHz, CDCl_3_): δ 7.46–7.43 (m, 2H), 7.38–7.28 (m, 3H), 7.17–6.99 (m, 5H), 6.93 (br s, 5H), 6.61 (ddd, ^3^J_8,9_ = 9.7 Hz, ^3^J_7,8_ = 6.1 Hz, ^4^J_8,9a_ = 0.8 Hz, 1H, H-8), 6.00 (ddd, ^3^J_8,9_ = 9.7 Hz, ^3^J_9a,9_ = 4.2 Hz, ^4^J_7,9_ = 0.7 Hz, 1H, H-9), 5.84 (d, ^3^J_9a,9_ = 4.2 Hz, 1H, H-9a), 5.43 (dd, ^3^J_7,8_ = 6.1 Hz, ^3^J_7,9_ = 0.7 Hz, 1H, H-7). ^13^C-NMR (100.6 MHz, CDCl_3_): δ 184.2 (q, ^2^J_CF_ = 36.1 Hz, C-12), 157.3 (C-4), 139.2, 136.4, 134.7, 133.9, 132.5, 132.1, 131.4, 129.5, 128.9, 128.4, 128.3, 127.8, 127.7, 127.2, 122.8 (q, ^1^J_CF_ = 285.7 Hz, CF_3_), 120.7, 115.0 [q, ^1^J_CF_ = 293.6 Hz, C(O)CF_3_], 114.7, 106.7, 89.7 (C-11), 81.5 (C-9a), 81.2 (C-10), 74.5 (q, ^2^J_CF_ = 34.8 Hz, C-2). ^19^F-NMR (376.3 MHz, CDCl_3_): δ −73.9 [C(O)CF_3_], −76.0 (CF_3_).

*1-(9-Bromo-4-phenyl-2-(phenylethynyl)-2-(trifluoromethyl)-2H,9aH-pyrido[2,1-b][1,3]oxazin-3-yl)-2,2,2-trifluoroethan-1-one* (**3p**). Major (9-Br)-regioisomer, obtained as a mixture (1:5) with minor (7-Br)-regioisomer (**3o**) from pyridine **1g** (0.082 g, 0.52 mmol) and CF_3_-ynone **2a** (0.208 g, 1.05 mmol). Yellow powder, m.p. 76.0–77.8 °C (hexane), yield 0.153 g (53%). (2S*,9aS*):(2R*,9aS*)-isomers ratio of **3p** is 80:20 (^19^F-NMR). HRMS (ESI-TOF) for the mixture of **3o** and **3p**: *m*/*z* [M + H]^+^ Calcd for C_25_H_15_F_6_BrNO_2_^+^: 554.0185; found: 554.0190.

(2S*,9aS*)-**3p**: ^1^H-NMR (400.1 MHz, CDCl_3_): δ 7.65–7.30 (m, 10H), 6.80 (d, ^3^*J*_6,7_ = 6.6 Hz, 1H, H-6), 6.45 (d, ^3^*J*_7,8_ = 7.5 Hz, 1H, H-8), 5.79 (s, 1H, H-9a), 5.38 (pseudo-t, ^3^*J* ~ 7 Hz, 1H, H-7). ^13^C-NMR (100.6 MHz, CDCl_3_): δ 181.1 (q, ^2^*J*_CF_ = 35.6 Hz, C-12), 158.7 (C-4), 133.4, 132.3, 132.1, 131.1 (q, ^3^*J*_CF_ = 1.8 Hz, C-3), 129.6 (br s), 129.4 (C-8), 128.7 (C-6), 128.3, 125.2, 122.4 (q, ^1^*J*_CF_ = 286.4 Hz, CF_3_), 121.0 (C*_i_* from Ar), 117.4 (C-9), 115.4 [q, ^1^*J*_CF_ = 292.6 Hz, C(O)CF_3_], 109.9, 103.3 (C-7), 89.2 (C-11), 82.9 (C-10), 80.5 (C-9a), 74.7 (q, ^2^*J*_CF_ = 34.8 Hz, C-2). ^19^F-NMR (376.3 MHz, CDCl_3_): δ −72.7 [C(O)CF_3_], −76.8 (CF_3_).

(2R*,9aS*)-**3p′**: ^1^H-NMR (400.1 MHz, CDCl_3_): δ 6.75 (d, ^3^*J*_6,7_ = 6.7 Hz, 1H, H-6), 6.30 (d, ^3^*J*_7,8_ = 7.6 Hz, 1H, H-8), 6.16 (s, 1H, H-9a), 5.25 (pseudo-t, ^3^*J* ~ 7 Hz, 1H, H-7). Other signals are overlapped with those of major isomer. ^13^C-NMR of (2R*,9aS*)-**3p′** and ^13^C-NMR of (2S*,9aS*)-**3o** (100.6 MHz, CDCl_3_): δ 158.6 (C-4), 152.3, 133.5, 132.6, 132.2, 130.8, 131.0 (q, ^3^*J*_CF_ = 1.3 Hz, C-3), 130.5, 129.5, 129.3, 128.94, 128.91, 128.36, 128.31, 125.7, 121.5, 121.3, 120.8, 110.8, 101.8, 89.02 and 88.98 (C-11), 83.53 and 83.49 (C-10), 81.3 and 80.9 (C-9a), 77.8. Due to low concentration and equal amounts of (2R*,9aS*)-**3p′** and ^13^C-NMR of (2S*,9aS*)-**3o** assignment of their signals cannot be done. ^13^C-NMR are reported together. Other signals are overlapped with those of major isomer **3p** or cannot be seen in the spectrum due to the low concentration of minor isomers. ^19^F-NMR (376.3 MHz, CDCl_3_): δ −74.7 [C(O)CF_3_], −75.9 (CF_3_).

*1-(7-Bromo-4-phenyl-2-(phenylethynyl)-2-(trifluoromethyl)-2H,9aH-pyrido[2,1-b][1,3]oxazin-3-yl)-2,2,2-trifluoroethan-1-one* (**3p**). Minor (7-Br)-regioisomer, obtained as a mixture with major (9-Br)-regioisomer (**3q**) (see above). (2S*,9aS*):(2R*,9aS*)-isomers ratio is 78:22 (^19^F-NMR). HRMS (ESI-TOF) for the mixture of **3o** and **3p**: *m/z* [M + H]^+^ Calcd for C_25_H_15_F_6_BrNO_2_^+^: 554.0185; found: 554.0190.

(2S*,9aS*)-**3o**: ^1^H-NMR (400.1 MHz, CDCl_3_): δ 6.65 (s, 1H, H-6), 6.55 (d, ^3^*J*_8,9_ = 10.1 Hz, 1H, H-8), 5.98 (dd, ^3^*J*_8,9_ = 10.1 Hz, ^3^*J*_9a,9_ = 3.9 Hz, 1H, H-9), 5.71 (d, ^3^*J*_9,9a_ = 3.9 Hz, 1H, H-9a). Other signals are overlapped with those of major isomer. ^13^C-NMR (100.6 MHz, CDCl_3_): See above in **3p** section. ^19^F-NMR (376.3 MHz, CDCl_3_): δ −72.7 [C(O)CF_3_], −77.1 (CF_3_).

(2R*,9aS*)-**3o′**: ^1^H-NMR (400.1 MHz, CDCl_3_): δ 6.62 (s, 1H, H-6), 6.40 (d, ^3^*J*_8,9_ = 10.1 Hz, 1H, H-8), 5.84 (dd, ^3^*J*_8,9_ = 10.1 Hz, ^3^*J*_9a,9_ = 4.2 Hz, 1H, H-9), 6.06 (d, ^3^*J*_9,9a_ = 4.2 Hz, 1H, H-9a). Other signals are overlapped with those of major isomer. ^13^C-NMR (100.6 MHz, CDCl_3_): cannot be seen in the spectrum due to the low concentration of minor isomer. ^19^F-NMR (376.3 MHz, CDCl_3_): δ −74.7 [C(O)CF_3_], −75.6 (CF_3_).

*1-(7-Acetyl-4-phenyl-2-(phenylethynyl)-2-(trifluoromethyl)-2H,9aH-pyrido[2,1-b][1,3]oxazin-3-yl)-2,2,2-trifluoroethan-1-one* (**3q**). Major (7-Ac)-regioisomer, obtained as a mixture (2:1) with minor (9-Ac)-regioisomer (**3r**) from pyridine **1h** (0.065 g, 0.54 mmol) and CF_3_-ynone **2a** (0.214 g, 1.08 mmol). Yellow powder, m.p. 114.4–115.3 °C (hexane), yield 0.224 g (80%). (2S*,9aS*):(2R*,9aS*)-isomers ratio is 83:17 (^1^H-NMR). HRMS (ESI-TOF) for the mixture of **3q** and **3r**: *m*/*z* [M + H]^+^ Calcd for C_27_H_18_F_6_NO_3_^+^: 518.1185; found: 518.1196.

(2S*,9aS*)-**3q**: ^1^H-NMR (400.1 MHz, CDCl_3_): δ 7.69–7.29 (m, 11H), 7.10 (d, ^3^*J*_8,9_ = 10.1 Hz, 1H, H-8), 6.00 (dd, ^3^*J*_8,9_ = 10.1 Hz, ^3^*J*_9a,9_ = 3.6 Hz, 1H, H-9), 5.88 (d, ^3^*J*_9a,9_ = 3.6 Hz, 1H, H-9a), 2.12 (s, 3H, Me). ^13^C-NMR (100.6 MHz, CDCl_3_): δ 193.1, 182.1 (q, ^2^*J*_CF_ = 36.3 Hz, C-12), 156.2 (C-4), 133.7, 133.6, 132.1, 129.9, 129.7, 128.4 (C-8), 130.4 (q, ^3^*J*_CF_ = 1.3 Hz, C-3), 124.2 (C-6), 122.2 (q, ^1^*J*_CF_ = 286.0 Hz, CF_3_), 120.4 (C*_i_* from Ar), 115.3 (C-9), 115.0 (q, ^1^*J*_CF_ = 293.0 Hz, C(O)CF_3_), 102.3, 90.1 (C-11), 80.3 (C-10), 79.1 (C-9a), 74.2 (q, ^2^*J*_CF_ = 34.8 Hz, C-2), 25.0. ^19^F-NMR (376.3 MHz, CDCl_3_): δ −73.7 [C(O)CF_3_], −76.8 (CF_3_).

(2R*,9aS*)-**3q′**: ^1^H-NMR (400.1 MHz, CDCl_3_): δ 7.17 (s, 1H, H-6), 7.05 (d, ^3^*J*_8,9_ = 10.2 Hz, 1H, H-8), 6.17 (d, ^3^*J*_9a,9_ = 3.6 Hz, 1H, H-9a), 5.90 (dd, ^3^*J*_8,9_ = 10.2 Hz, ^3^*J*_9a,9_ = 3.6 Hz, 1H, H-9), 2.06 (s, 3H, Me). ^13^C-NMR (100.6 MHz, CDCl_3_): δ 193.0, 131.8, 128.3, 114.5, 24.9. Other signals are overlapped with those of major isomer or cannot be seen in the spectrum due to the low concentration of minor isomer. ^19^F-NMR (376.3 MHz, CDCl_3_): δ −74.9 [C(O)CF_3_], −75.5 (CF_3_).

*1-(9-Acetyl-4-phenyl-2-(phenylethynyl)-2-(trifluoromethyl)-2H,9aH-pyrido[2,1-b][1,3]oxazin-3-yl)-2,2,2-trifluoroethan-1-one* (**3r**). Minor (9-Ac)-regioisomer, obtained as a mixture with major (7-Ac)-regioisomer (**3q**) (see above). (2S*,9aS*):(2R*,9aS*)-isomers ratio is 87:13 (^1^H-NMR). HRMS (ESI-TOF) for the mixture of **3q** and **3r**: *m/z* [M + H]^+^ Calcd for C_27_H_18_F_6_NO_3_^+^: 518.1185; found: 518.1196.

(2S*,9aS*)-**3r**: ^1^H-NMR (400.1 MHz, CDCl_3_): δ 7.69–7.29 (m, 11H), 6.65 (d, ^3^*J*_6,7_ = 7.3 Hz, 1H, H-6), 6.20 (s, 1H, H-9a), 5.62 (pseudo-t, ^3^*J* ~ 7 Hz, 1H, H-7), 2.47 (s, 3H, Me). ^13^C-NMR (100.6 MHz, CDCl_3_): δ 194.6, 181.5 (q, ^2^*J*_CF_ = 35.8 Hz, C-12), 158.0 (C-4), 134.2, 133.5, 132.1, 131.0 (q, ^3^*J*_CF_ = 1.7 Hz, C-3), 129.4, 128.3, (C-8), 124.9 (C-6), 122.3 (q, ^1^*J*_CF_ = 286.6 Hz, CF_3_), 120.9 (C*_i_* from Ar), 115.7 (C-9), 115.2 (q, ^1^*J*_CF_ = 293.0 Hz, C(O)CF_3_), 89.6 (C-11), 80.0 (C-10), 77.7 (C-9a), 74.4 (q, ^2^*J*_CF_ = 34.5 Hz, C-2), 25.7. ^19^F-NMR (376.3 MHz, CDCl_3_): δ −73.0 [C(O)CF_3_], −76.8 (CF_3_).

(2R*,9aS*)-**3r′**: ^1^H-NMR (400.1 MHz, CDCl_3_): δ 6.58 (s, 1H, H-9a), 6.51 (d, ^3^*J*_6,7_ = 7.4 Hz, 1H, H-6), 5.62 (pseudo-t, ^3^*J* ~ 7 Hz, 1H, H-7), 2.43 (s, 3H, Me). Other signals are overlapped with those of major isomer. ^13^C-NMR (100.6 MHz, CDCl_3_): δ 194.7, 134.3, 133.0, 132.2, 129.5, 114.9, 25.5. Other signals are overlapped with those of major isomer or cannot be seen in the spectrum due to the low concentration of minor isomer. ^19^F-NMR (376.3 MHz, CDCl_3_): δ −74.9 [C(O)CF_3_], −76.4 (CF_3_).

*4-Phenyl-2-(phenylethynyl)-3-(2,2,2-trifluoroacetyl)-2-(trifluoromethyl)-2H,9aH-pyrido[2,1-b][1,3]oxazine-7-carbonitrile* (**3s**). Major (7-CN)-regioisomer, obtained as a mixture (2.5:1) with minor (9-CN)-regioisomer (**3t**) from pyridine **1i** (0.054 g, 0.5 mmol) and CF_3_-ynone **2a** (0.208 g, 1.05 mmol). Yellow powder, m.p. 95–96 °C (hexane), yield 0.165 g (66%). (2S*,9aS*):(2R*,9aS*)-isomers ratio is 76:24 (^19^F-NMR). HRMS (ESI-TOF) for the mixture of **3s** and **3t**: *m*/*z* [M + H]^+^ Calcd for C_26_H_15_F_6_N_2_O_2_^+^: 501.1032; found: 501.1055.

(2S*,9aS*)-**3s**: ^1^H-NMR (400.1 MHz, CDCl_3_): δ 7.69–7.28 (m, 10H), 6.98 (s, 1H, H-6), 6.49 (d, ^3^*J*_8,9_ = 10.0 Hz, 1H, H-8), 6.00 (dd, ^3^*J*_8,9_ = 10.0 Hz, ^3^*J*_9a,9_ = 3.5 Hz, 1H, H-9), 5.92–5.89 (m, 1H, H-9a). ^13^C-NMR (100.6 MHz, CDCl_3_): δ 182.0 (q, ^2^*J*_CF_ = 37.0 Hz, C-12), 154.5 (C-4), 136.0, 133.7, 132.0, 130.0, 129.9, 128.4, 130.3 (q, ^4^*J*_CF_ = 1.7 Hz, C-3), 124.4 (C-6), 122.1 (q, ^1^*J*_CF_ = 286.4 Hz, CF_3_), 120.2 (C*_i_* from Ar), 116.3 (C-9), 114.9 [q, ^1^*J*_CF_ = 293.0 Hz, C(O)CF_3_], 113.3 (CN), 101.4, 88.5 (C-11), 79.9 (C-10), 78.3 (C-9a), 74.3 (q, ^2^*J*_CF_ = 34.7 Hz, C-2). ^19^F-NMR (376.3 MHz, CDCl_3_): δ −73.9 [C(O)CF_3_], −76.8 (CF_3_).

(2R*,9aS*)-**3s′**: ^1^H-NMR (400.1 MHz, CDCl_3_): δ 6.87 (s, 1H, H-6), 6.44 (d, ^3^*J*_8,9_ = 10.0 Hz, 1H, H-8), 6.20 (d, ^3^*J*_9a,9_ = 3.6 Hz, 1H, H-9a). ^13^C-NMR (100.6 MHz, CDCl_3_): δ 149.8, 136.5, 133.1, 132.2, 120.8, 115.6, 112.4 (CN), 100.3, 86.8, 80.1, 78.8 (q, ^4^*J*_CF_ = 3.7 Hz, C-10), 72.1 (q, ^2^*J*_CF_ = 32.4 Hz, C-2). Other signals are overlapped with those of major isomer or cannot be seen in the spectrum due to the low concentration of minor isomer. ^19^F-NMR (376.3 MHz, CDCl_3_): δ −75.0 [C(O)CF_3_], −76.5 (CF_3_).

*4-Phenyl-2-(phenylethynyl)-3-(2,2,2-trifluoroacetyl)-2-(trifluoromethyl)-2H,9aH-pyrido[2,1-b][1,3]oxazine-7-carbonitrile* (**3t**). Minor (9-CN)-regioisomer, obtained as a mixture with major (7-CN)-regioisomer (**3s**) (see above). (2S*,9aS*):(2R*,9aS*)-isomers ratio is 86:14 (^19^F-NMR). HRMS (ESI-TOF) for the mixture of **3s** and **3t**: *m*/*z* [M + H]^+^ Calcd for C_26_H_15_F_6_N_2_O_2_^+^: 501.1032; found: 501.1055.

(2S*,9aS*)-**3t**: ^1^H-NMR (400.1 MHz, CDCl_3_): δ 7.69–7.28 (m, 10H), 7.14 (d, ^3^*J*_7,8_ = 6.5 Hz, 1H, H-8), 6.64 (d, ^3^*J*_6,7_ = 7.5 Hz, 1H, H-6), 5.92–5.89 (m, 1H, H-9a), 5.54 (pseudo-t, ^3^*J* ~ 7 Hz, 1H, H-7). ^13^C-NMR (100.6 MHz, CDCl_3_): δ 182.5 (q, ^2^*J*_CF_ = 37.2 Hz, C-12), 156.4 (C-4), 139.3, 133.6, 132.1, 131.3, 129.7, 129.6, 129.3, 128.3, 124.3 (C-6), 122.1 (q, ^1^*J*_CF_ = 286.6 Hz, CF_3_), 120.4 (C*_i_* from Ar), 117.2 (C-9), 115.1 [q, ^1^*J*_CF_ = 293.2 Hz, C(O)CF_3_], 112.5 (CN), 98.7, 90.7 (C-11), 87.7, 79.8 (C-10), 78.2 (C-9a), 74.5 (q, ^2^*J*_CF_ = 35.6 Hz, C-2). ^19^F-NMR (376.3 MHz, CDCl_3_): δ −73.5 [C(O)CF_3_], −76.8 (CF_3_).

(2R*,9aS*)-**3t′**: ^1^H-NMR (400.1 MHz, CDCl_3_): δ 7.09 (d, ^3^*J*_7,8_ = 6.4 Hz, 1H, H-8), 6.23 (s, 1H, H-9a). Other signals are overlapped with those of major isomer. ^13^C-NMR (100.6 MHz, CDCl_3_): δ 149.5, 132.9, 132.7, 97.8, 86.3, 90.4. Other signals are overlapped with those of major isomer or cannot be seen in the spectrum due to the low concentration of minor isomer. ^19^F-NMR (376.3 MHz, CDCl_3_): δ −74.9 [C(O)CF_3_], −76.5 (CF_3_).

## 4. Conclusions

In conclusion, a new efficient pathway towards to trifluoromethylated oxazinopyridines was elaborated on the base of a one-pot, metal-free 1:2 assembly of pyridines and CF_3_-ynones. The reaction has a broad scope in terms of both pyridines and CF_3_-ynones used. Therefore, pyridines with electron withdrawing as well as electron donating groups afforded corresponding products in up to 99% yield. Various CF_3_-ynones including bulky ones can also be involved in the reaction. High stereoselectivity (up to 100% for 2-substitueted pyridines) is the advantage of the method. However, dramatic influence of the pKa values of pyridines on the reaction course was observed. Pyridines having pKa lower than ~1 do not react with CF_3_-ynones. The possible mechanism of the reaction includes a cascade of ionic transformations triggered by attack of the nitrogen of pyridine molecule by electron-deficient triple bond of CF_3_-ynone.

## Supplementary Materials

Copy of all ^1^H-, ^13^C- and ^19^F-NMR spectra are available online at https://www.mdpi.com/1420-3049/24/19/3594/s1.
