# Supplementary material for: One-Pot Metal-Free Synthesis of 3-CF3-1,3-Oxazinopyridines by Reaction of Pyridines with CF3CO-Acetylenes
Source: Molecules. 2019 Oct 6;24(19):3594. doi: 10.3390/molecules24193594 (PMC6804262; doi:10.3390/molecules24193594)
Supplement: Supplementary file 1 [file molecules-24-03594-s001.pdf]

One-pot metal free synthesis of 3-CF_3-1,3-oxazinopyridines by assembly of pyridines with CF_3CO-acetylenes

Vasiliy M. Muzalevskiy[1], Zoia A. Sizova[1], Kseniya V. Belyaeva[2], Boris A. Trofimov*[2], Valentine G. Nenajdenko*[1]

[1] M. V. Lomonosov Moscow State University, Department of Chemistry, Leninskie Gory 1, Moscow, 119991 Russian Federation; muzvas@mail.ru (V.M.M); syzova@mail.ru (Z.A.S.)

[2] A. E. Favorsky Irkutsk Institute of Chemistry, Siberian Branch, Russian Academy of Sciences, 1 Favorsky Str., Irkutsk, 664033 Russian Federation; Fax: (395-2)41-93-46; belyaeva@irioch.irk.ru (K.V.B.)

* Correspondence: boris_trofimov@irioch.irk.ru (B.A.T.); nenajdenko@gmail.com (V.G.N.);

Table of contents

General Details S2

Reaction of CF_3-ynones and pyridines (general procedure) S2

Characterization data of compounds 3 S2-S12

Copies of all NMR spectra of compounds 3 S13-S72

General details

^{1}H, ^{13}C and ^{19}F NMR spectra were recorded on Bruker AVANCE 400 MHz spectrometer in CD_3CN and $CDCl_3$ at 400.1, 100.6 and 376.3 MHz respectively. Chemical shifts (*δ*) in ppm are reported with the use of the residual CHD_2CN and chloroform signals (1.94 and 7.25 for ^{1}H and 77.0 for ^{13}C) as internal reference. The ^{19}F chemical shifts were referenced to C_6F_6, (-162.9 ppm). HRMS (ESI-TOF) spectra were measured with an Orbitrap Elite instrument. TLC analysis was performed on "Merck 60 F_{254}" plates. All reagents were of reagent grade and were used as such or distilled prior to use. CF_3-ynones **2** were prepared as reported previously.[Muzalevskiy, V. M. Rulev, A. Yu.; Romanov, A. R.; Kondrashov, E. V.; Ushakov, I. A.; Chertkov, V. A.; Nenajdenko, V. G. Selective, Metal-Free Approach to 3- or 5-CF_3-Pyrazoles: Solvent Switchable Reaction of CF_3-Ynones with Hydrazines. *J. Org. Chem.* **2017**, *82*, 7200-7214.] Melting points were determined on an Electrothermal 9100 apparatus.

Reaction of CF_3-ynones and pyridines (general procedure)

A 4 mL vial with a screw cap was charged with CF_3-ynone **2** (1-1.05 mmol, 2-2.1 equiv.)* and then pyridine **1** (0.5 mmol, 1 equiv.) was added in one portion. After vigorous stirring for several minutes the reaction mixture became viscous due to crystallization of the product. At that moment MeCN (0.5 mL) was added to form homogeneous solution again and the reaction mixture was left overnight at stirring. Next volatiles were evaporated in vacuo, the residue was crystallized from appropriate amount of ether-hexane mixtures or purified via column chromatography on silica gel using mixtures of hexane with CH_2Cl_2.

*-In case of solid CF_3-ynones **2** minimal amount (as much as possible) of MeCN (0.1-0.2 mL) was added to form clear solution.

2,2,2-Trifluoro-1-(4-phenyl-2-(phenylethynyl)-2-(trifluoromethyl)-2*H*,9a*H*-pyrido[2,1-*b*][1,3]oxazin-3-yl)ethan-1-one (3a). Obtained from pyridine **1a** (0.042 g, 0.53 mmol) and CF_3-ynone **2a** (0.212 g, 1.071 mmol). Yellow-brown powder, m.p. 109.4-111.8 °C (hexane), yield 0.238 g (94%). (2*S**,9a*S**):(2*R**,9a*S**)-isomers ratio is 90:10 (^{19}F NMR). HRMS (ESI-TOF): m/z [M+H]$^+$ Calcd for $C_{25}H_{16}F_6NO_2^+$: 476.1080; found: 476.1085.

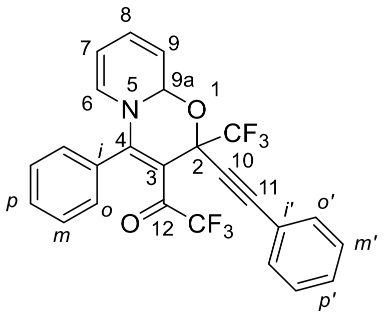

(2*S**,9a*S**)-**3a**: ^{1}H NMR (400.1 MHz, $CDCl_3$): δ 7.68-7.39 (m, 7H), 7.37-7.27 (m, 3H), 6.50 (dd, $^3J_{8,9}$ = 9.7 Hz, $^3J_{7,8}$ = 6.1 Hz, 1H, H-8), 6.46 (d, $^3J_{6,7}$ = 7.7 Hz, 1H, H-6), 6.00 (dd, $^3J_{8,9}$ = 9.7 Hz, $^3J_{9a,9}$ = 3.9 Hz, 1H, H-9), 5.74 (d, $^3J_{9a,9}$ = 3.9 Hz, 1H, H-9a), 5.50 (pseudo-td, ^3J ~ 7 Hz, ^4J ~ 1 Hz, 1H, H-7) ppm. ^{13}C NMR (100.6 MHz, $CDCl_3$): δ 180.7 (q, $^2J_{CF}$ = 35.0 Hz, C-12), 160.3 (C-4), 133.2, 132.1, 131.4 ($C_{i'}$ from Ar), 129.5, 129.3, 129.2, 128.3 (C-8), 126.2 (C-6), 125.8, 122.6 (q, $^1J_{CF}$ = 286.6 Hz, CF_3), 121.1 (C_i from Ar), 116.5 (C-9), 115.5 [q, $^1J_{CF}$ = 292.7 Hz, C(O)CF_3], 104.0 (C-7), 88.4 (C-11), 81.3 (C-10), 79.1 (C-9a), 73.7 (q, $^2J_{CF}$ = 33.9 Hz, C-2) ppm. ^{19}F NMR (376.3 MHz, $CDCl_3$): δ -72.5 [C(O)CF_3], -77.3 (CF_3) ppm.

(2*R**,9a*S**)-**3a'**: ^{1}H NMR (400.1 MHz, $CDCl_3$): δ 6.43 (dd, $^3J_{8,9}$ = 9.8 Hz, $^3J_{7,8}$ = 6.1 Hz, 1H, H-8), 6.28 (d, $^3J_{6,7}$ = 7.6 Hz, 1H, H-6), 6.11 (d, $^3J_{9a,9}$ = 4.0 Hz, 1H, H-9a), 5.85 (dd, $^3J_{8,9}$ = 9.8 Hz, $^3J_{9a,9}$ = 4.0 Hz, 1H, H-9), 5.34 (pseudo-td, ^3J ~ 7 Hz, ^4J = 1 Hz, 1H, H-7) ppm. Other signals are overlapped

with those of major isomer. ^{13}C NMR (100.6 MHz, $CDCl_3$): δ 133.9, 132.3, 128.9, 126.5, 126.3, 115.0, 109.4, 102.2 ppm. Other signals are overlapped with those of major isomer or can not be seen in the spectrum due to the low concentration of minor isomer. ^{19}F NMR (376.3 MHz, $CDCl_3$): δ -74.6 [C(O)CF_3], -76.2 (CF_3) ppm.

1-(4-(4-(*Tert*-butyl)phenyl)-2-((4-(*tert*-butyl)phenyl)ethynyl)-2-(trifluoromethyl)-2*H*,9a*H*-pyrido[2,1-*b*][1,3]oxazin-3-yl)-2,2,2-trifluoroethan-1-one (3b). Obtained from pyridine **1a** (0.041 g, 0.518 mmol) and CF_3-ynone **2b** (0.267 g, 1.051 mmol). Yellow powder, m.p. 130.0-132.7 °C (hexane), yield 0.300 g (98%). (2*S**,9a*S**):(2*R**,9a*S**)-isomers ratio is 90:10 (^{19}F NMR). HRMS (ESI-TOF): m/z $[M+H]^+$ Calcd for $C_{33}H_{32}F_6NO_2^+$: 588.2332; found: 588.2340.

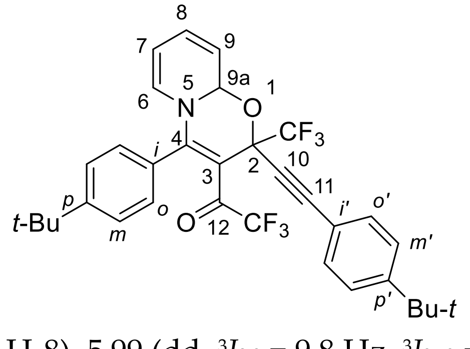

(2*S**,9a*S**)-**3b**: ^{1}H NMR (400.1 MHz, $CDCl_3$): δ 7.52 (d, ^3J = 8.4 Hz, 2H), 7.47-7.36 (m, 4H), 7.32 (d, ^3J = 8.4 Hz, 2H), 6.52 (d, $^3J_{6,7}$ = 7.8 Hz, 1H, H-6), 6.48 (dd, $^3J_{8,9}$ = 9.8 Hz, $^3J_{7,8}$ = 6.1 Hz, 1H, H-8), 5.99 (dd, $^3J_{8,9}$ = 9.8 Hz, $^3J_{9a,9}$ = 3.9 Hz, 1H, H-9), 5.72 (d, $^3J_{9a,9}$ = 3.9 Hz, 1H, H-9a), 5.49 (psedo-t, ^3J ~ 7 Hz, 1H, H-7), 1.35 (s, 9H, 3Me from *t*-Bu), 1.29 (s, 9H, 3Me from *t*-Bu) ppm. ^{13}C NMR (100.6 MHz, $CDCl_3$): δ 180.8 (q, $^2J_{CF}$ = 35.0 Hz, C-12), 160.4 (C-4), 157.2 (C_p from Ar), 152.7 ($C_{p'}$ from Ar), 131.9 ($C_{m,m'}$ from Ph), 128.6 (q, $^3J_{CF}$ = 2.2 Hz, C-3), 126.4, 126.3 (C-8), 126.1 (C-6), 125.3 ($C_{o,o'}$ from Ar), 122.6 (q, $^1J_{CF}$ = 286.6 Hz, CF_3), 118.1 ($C_{i'}$ from Ar), 116.5 (C-9), 115.7 [q, $^1J_{CF}$ = 292.5 Hz, C(O)CF_3], 103.6 (C-7), 88.5 (C-11), 80.9 (C-10), 79.0 (C-9a), 73.8 (q, $^2J_{CF}$ = 34.6Hz, C-2), 35.2, 34.8, 31.1, 31.0 ppm. ^{19}F NMR (376.3 MHz, $CDCl_3$): δ -72.5 [C(O)CF_3], -77.4 (CF_3) ppm.

(2*R**,9a*S**)-**3b'**: ^{1}H NMR (400.1 MHz, $CDCl_3$): δ 6.43 (dd, $^3J_{8,9}$ = 9.8 Hz, $^3J_{7,8}$ = 6.0 Hz, 1H, H-8), 6.33 (d, $^3J_{6,7}$ = 7.6 Hz, 1H, H-6), 6.10 (d, $^3J_{9a,9}$ = 3.9 Hz, 1H, H-9a), 5.84 (dd, $^3J_{8,9}$ = 9.8 Hz, $^3J_{9a,9}$ = 3.9 Hz, 1H, H-9), 5.33 (pseudo-t, ^3J ~ 7 Hz, 1H, H-7), 1.33 (s, 9H, 3Me from *t*-Bu), 1.30 (s, 9H, 3Me from *t*-Bu) ppm. Other signals are overlapped with those of major isomer. ^{13}C NMR (100.6 MHz, $CDCl_3$): δ 132.0, 126.6, 126.51, 126.49, 125.2, 109.2, 35.1, 31.2, 30.9 ppm. Other signals are overlapped with those of major isomer or can not be seen in the spectrum due to the low concentration of minor isomer. ^{19}F NMR (376.3 MHz, $CDCl_3$): δ -74.6 [C(O)CF_3], -76.2 (CF_3) ppm.

1-(4-(4-Methoxyphenyl)-2-((4-methoxyphenyl)ethynyl)-2-(trifluoromethyl)-2*H*,9a*H*-pyrido[2,1-*b*][1,3]oxazin-3-yl)-2,2,2-trifluoroethan-1-one (3c). Obtained from pyridine **1a** (0.041 g, 0.518 mmol) and CF_3-ynone **2c** (0.239 g, 1.048 mmol). Light brown powder, m.p. 117.3-118.7 °C (hexane), yield 0.242 g (87%). (2*S**,9a*S**):(2*R**,9a*S**)-isomers ratio is 90:10 (^{19}F NMR). HRMS (ESI-TOF): m/z $[M+H]^+$ Calcd for $C_{27}H_{20}F_6NO_4^+$: 536.1291; found: 536.1296.

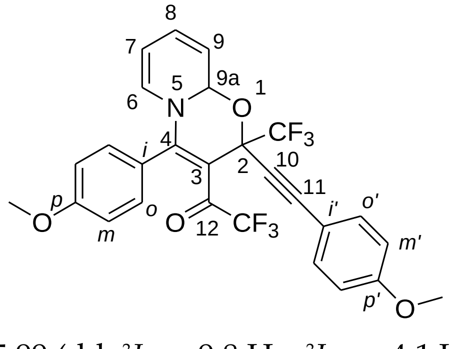

(2*S**,9a*S**)-**3c**: ^{1}H NMR (400.1 MHz, $CDCl_3$): δ 7.48-7.30 (m, 4H), 7.07-6.91 (m, 2H), 6.82 (d, ^3J = 8.9 Hz, 2H), 6.49 (d, $^3J_{6,7}$ = 7.0 Hz, 1H, H-6), 6.48 (dd, $^3J_{8,9}$ = 9.8 Hz, $^3J_{7,8}$ = 6.1 Hz, 1H, H-8), 5.99 (dd, $^3J_{8,9}$ = 9.8 Hz, $^3J_{9a,9}$ = 4.1 Hz, 1H, H-9), 5.71 (d, $^3J_{9a,9}$ = 4.1 Hz, 1H, H-9a), 5.49 (pseudo-td, ^3J ~ 7 Hz, ^3J ~ 1 Hz, 1H, H-7), 3.88 (s, 3H, MeO), 3.79 (s, 3H, MeO) ppm. ^{13}C NMR (100.6 MHz, $CDCl_3$): δ 180.6 (q, $^2J_{CF}$ = 34.7 Hz, C-12), 163.7, 160.4 (C-4), 160.3, 133.7, 126.3 (C-8), 126.0 (C-6), 123.7, 122.7 (q, $^1J_{CF}$ = 286.8 Hz, CF_3), 116.5 (C-9), 115.7 [q, $^1J_{CF}$ = 293.0 Hz, C(O)CF_3], 113.9, 113.2, 108.6, 103.8 (C-7), 88.3 (C-11), 80.3 (C-10), 78.9 (C-9a), 73.9 (q, $^2J_{CF}$ = 34.3 Hz, C-2), 55.6, 55.2 ppm. ^{19}F NMR (376.3 MHz, $CDCl_3$): δ -72.4 [C(O)CF_3], -77.5 (CF_3) ppm.

(2*R**,9a*S**)-**3c′**: ^{1}H NMR (400.1 MHz, $CDCl_3$): δ 7.52 (d, ^{3}J = 8.9 Hz, 2H), 6.42 (dd, $^{3}J_{8,9}$ = 9.8 Hz, $^{3}J_{7,8}$ = 6.1 Hz, 1H, H-8), 6.31 (d, $^{3}J_{6,7}$ = 7.5 Hz, 1H, H-6), 6.07 (d, $^{3}J_{9a,9}$ = 4.1 Hz, 1H, H-9a), 5.84 (dd, $^{3}J_{8,9}$ = 9.8 Hz, $^{3}J_{9a,9}$ = 4.1 Hz, 1H, H-9), 5.33 (pseudo-t, ^{3}J ~ 7 Hz, 1H, H-7), 3.86 (s, 3H, MeO), 3.81 (s, 3H, MeO) ppm. Other signals are overlapped with those of major isomer. ^{13}C NMR (100.6 MHz, $CDCl_3$): δ 136.0, 133.8, 131.8, 126.51, 126.48, 114.4, 55.5 ppm. Other signals are overlapped with those of major isomer or can not be seen in the spectrum due to the low concentration of minor isomer. ^{19}F NMR (376.3 MHz, $CDCl_3$): δ -74.6 [C(O)CF_3], -76.2 (CF_3) ppm.

1-(4-(4-Bromophenyl)-2-((4-bromophenyl)ethynyl)-2-(trifluoromethyl)-2*H*,9a*H*-pyrido[2,1-*b*][1,3]oxazin-3-yl)-2,2,2-trifluoroethan-1-one (3d). Obtained from pyridine **1a** (0.0395 g, 0.5 mmol) and CF_3-ynone **2d** (0.292 g, 1.054 mmol). Yellow-brown powder, m.p. 83.9-86.7 °C (hexane), yield 0.244 g (77%). (2*S**,9a*S**):(2*R**,9a*S**)-isomers ratio is 89:11 (^{19}F NMR). HRMS (ESI-TOF): m/z [M+H]$^+$ Calcd for $C_{25}H_{14}Br_2F_6NO_2^+$: 633.9270; found: 633.9282.

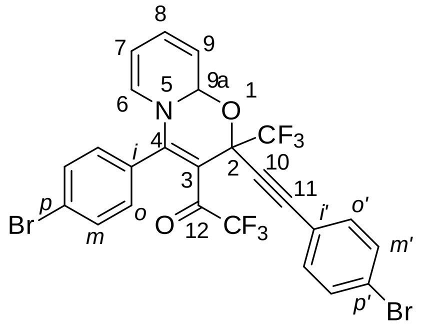

(2*S**,9a*S**)-**3d**: ^{1}H NMR (400.1 MHz, $CDCl_3$): δ 7.68-7.29 (m, 8H), 6.49 (dd, $^{3}J_{8,9}$ = 9.8 Hz, $^{3}J_{7,8}$ = 6.1 Hz, 1H, H-8), 6.41 (d, $^{3}J_{6,7}$ = 7.6 Hz, 1H, H-6), 6.00 (dd, $^{3}J_{8,9}$ = 9.8 Hz, $^{3}J_{9a,9}$ = 3.9 Hz, 1H, H-9), 5.69 (d, $^{3}J_{9a,9}$ = 3.9 Hz, 1H, H-9a), 5.53 (pseudo-t, ^{3}J ~ 7 Hz, 1H, H-7) ppm. ^{13}C NMR (100.6 MHz, $CDCl_3$): δ 180.4 (q, $^{2}J_{CF}$ = 35.4 Hz, C-12), 159.3 (C-4), 149.6, 133.5, 132.9, 131.6, 130.2, 128.5, 126.3 (C-8), 125.4 (C-6), 123.9, 122.4 (q, $^{1}J_{CF}$ = 286.8 Hz, CF_3), 119.9, 116.7 (C-9), 115.5 [q, $^{1}J_{CF}$ = 292.7 Hz, C(O)CF_3], 109.4, 104.5 (C-7), 87.4 (C-11), 82.2 (C-10), 79.2 (C-9a), 73.6 (q, $^{2}J_{CF}$ = 34.3 Hz, C-2) ppm. ^{19}F NMR (376.3 MHz, $CDCl_3$): δ -72.3 [C(O)CF_3], -77.3 (CF_3) ppm.

(2*R**,9a*S**)-**3d′**: ^{1}H NMR (400.1 MHz, $CDCl_3$): δ 6.45-6.42 (m, 1H, H-8), 6.22 (d, $^{3}J_{6,7}$ = 7.5 Hz, 1H, H-6), 6.07 (d, $^{3}J_{9a,9}$ = 4.0 Hz, 1H, H-9a), 5.84 (dd, $^{3}J_{8,9}$ = 9.8 Hz, $^{3}J_{9a,9}$ = 4.0 Hz, 1H, H-9), 5.36 (pseudo-t, ^{3}J = 6.8 Hz, 1H, H-7) ppm. Other signals are overlapped with those of major isomer. ^{13}C NMR (100.6 MHz, $CDCl_3$): δ 136.1, 135.1, 133.7, 131.6, 126.5, 126.0, 123.8, 115.1, 102.6 ppm. Other signals are overlapped with those of major isomer or can not be seen in the spectrum due to the low concentration of minor isomer. ^{19}F NMR (376.3 MHz, $CDCl_3$): δ -74.5 [C(O)CF_3], -76.2 (CF_3) ppm.

1-(4-(4-Chlorophenyl)-2-((4-chlorophenyl)ethynyl)-2-(trifluoromethyl)-2*H*,9a*H*-pyrido[2,1-*b*][1,3]oxazin-3-yl)-2,2,2-trifluoroethan-1-one (3e). Obtained from pyridine **1a** (0.042 g, 0.53 mmol) and CF_3-ynone **2e** (0.254 g, 1.09 mmol). Yellow-brown powder, m.p. 68-70 °C (hexane), yield 0.286 g (99%). (2*S**,9a*S**):(2*R**,9a*S**)-isomers ratio is 89:11 (^{19}F NMR). HRMS (ESI-TOF): m/z [M+H]$^+$ Calcd for $C_{25}H_{14}Cl_2F_6NO_2^+$: 544.0300; found: 544.0308.

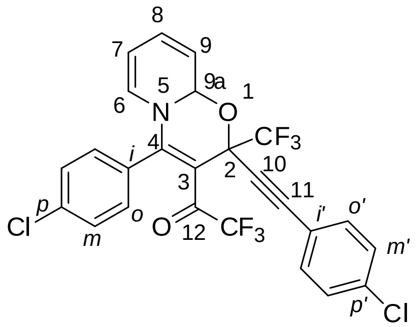

(2*S**,9a*S**)-**3e**: ^{1}H NMR (400.1 MHz, $CDCl_3$): δ 7.52-7.28 (m, 8H), 6.49 (dd, $^{3}J_{8,9}$ = 9.8 Hz, $^{3}J_{7,8}$ = 6.1 Hz, 1H, H-8), 6.41 (d, $^{3}J_{6,7}$ = 7.6 Hz, 1H, H-6), 6.00 (dd, $^{3}J_{8,9}$ = 9.8 Hz, $^{3}J_{9a,9}$ = 4.0 Hz, 1H, H-9), 5.69 (d, $^{3}J_{9a,9}$ = 4.0 Hz, 1H, H-9a), 5.53 (pseudo-t, ^{3}J ~ 7 Hz, 1H, H-7) ppm. ^{1}H NMR (400.1 MHz, CD_3CN): δ 7.73-7.31 (m, 8H), 6.57-6.52 (m, 2H, H-8, H-6), 6.03 (dd, $^{3}J_{8,9}$ = 9.8 Hz, $^{3}J_{9a,9}$ = 3.8 Hz, 1H, H-9), 5.75 (d, $^{3}J_{9a,9}$ = 3.8 Hz, 1H, H-9a), 5.61 (pseudo-t, ^{3}J = 7.2 Hz, 1H, H-7) ppm. ^{13}C NMR (100.6 MHz, $CDCl_3$): δ 180.3 (q, $^{2}J_{CF}$ = 34.7 Hz, C-12), 159.2 (C-4), 149.6, 140.0, 135.6, 133.3, 129.9, 128.7, 126.3 (C-8), 125.4 (C-6), 122.4 (q, $^{1}J_{CF}$ = 286.8 Hz, CF_3), 119.4, 116.7 (C-9), 115.5 [q, $^{1}J_{CF}$ = 292.7 Hz,

C(O)CF_3], 109.4, 104.5 (C-7), 87.3 (C-11), 82.0 (C-10), 79.1 (C-9a), 73.6 (q, $^2J_{CF}$ = 34.3 Hz, C-2) ppm. ^{19}F NMR (376.3 MHz, CD_3CN): δ -70.0 [C(O)CF_3], -75.4 (CF_3) ppm. ^{19}F NMR (376.3 MHz, $CDCl_3$): δ -72.2 [C(O)CF_3], -77.3 (CF_3) ppm.

(2*R**,9a*S**)-**3e′**: ^{1}H NMR (400.1 MHz, $CDCl_3$): δ 6.22 (d, $^3J_{6,7}$ = 7.6 Hz, 1H, H-6), 6.07 (d, $^3J_{9a,9}$ = 4.0 Hz, 1H, H-9a), 5.85 (dd, $^3J_{8,9}$ = 9.8 Hz, $^3J_{9a,9}$ = 4.0 Hz, 1H, H-9), 5.36 (pseudo-t, ^3J ~ 7 Hz, 1H, H-7) ppm. Other signals are overlapped with those of major isomer. ^{1}H NMR (400.1 MHz, CD_3CN): δ 6.48-6.42 (m, 1H, H-8), 6.32 (d, $^3J_{6,7}$ = 7.5 Hz, 1H, H-6), 5.89 (dd, $^3J_{8,9}$ = 9.8 Hz, $^3J_{9a,9}$ = 3.9 Hz, 1H, H-9), 5.42 (pseudo-t, ^3J = 6.8 Hz, 1H, H-7) ppm. Other signals are overlapped with those of major isomer. ^{13}C NMR (100.6 MHz, $CDCl_3$): δ 136.1, 133.5, 129.7, 128.6, 126.0, 123.9, 115.1, 102.6 ppm. Other signals are overlapped with those of major isomer or can not be seen in the spectrum due to the low concentration of minor isomer. ^{19}F NMR (376.3 MHz, CD_3CN): δ -72.2 [C(O)CF_3], -74.1 (CF_3) ppm. ^{19}F NMR (376.3 MHz, $CDCl_3$): δ -74.4 [C(O)CF_3], -76.1 (CF_3) ppm.

1-(4-(4-Methylphenyl)-2-((4-methylphenyl)ethynyl)-2-(trifluoromethyl)-2*H*,9a*H*-pyrido[2,1-*b*][1,3]oxazin-3-yl)-2,2,2-trifluoroethan-1-one (3f). Obtained from pyridine **1a** (0.044 g, 0.556 mmol) and CF_3-ynone **2f** (0.240 g, 1.13 mmol). Yellow-brown powder, m.p. 95.2-99.1 °C (hexane), yield 0.256 g (91%). (2*S**,9a*S**):(2*R**,9a*S**)-isomers ratio is 91:9 (^{19}F NMR). HRMS (ESI-TOF): m/z [M+H]$^+$ Calcd for $C_{27}H_{20}F_6NO_2^+$: 504.1393; found: 504.1401.

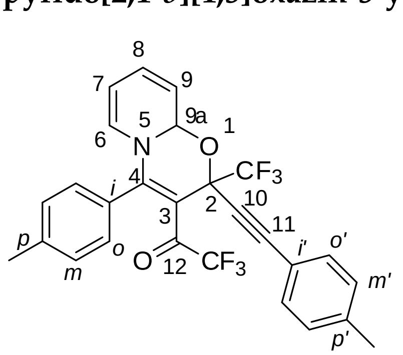

(2*S**,9a*S**)-**3f**: ^{1}H NMR (400.1 MHz, $CDCl_3$): δ 7.54-7.10 (m, 8H), 6.50-6.47 (m, 2H, H-8, H-6), 6.00 (dd, $^3J_{8,9}$ = 10.0 Hz, $^3J_{9a,9}$ = 3.8 Hz, 1H, H-9), 5.74 (d, $^3J_{9a,9}$ = 3.8 Hz, 1H, H-9a), 5.49 (pseudo-t, ^3J = 6.7 Hz, 1H, H-7), 2.44 (s, 3H, Me), 2.33 (s, 3H, Me) ppm. ^{13}C NMR (100.6 MHz, $CDCl_3$): δ 180.7 (q, $^2J_{CF}$ = 35.0 Hz, C-12), 160.5 (C-4), 139.5, 134.0, 132.0, 130.2, 129.0, 128.7, 126.2 (C-8), 125.9 (C-6), 122.6 (q, $^1J_{CF}$ = 286.8 Hz, CF_3), 118.0, 116.4 (C-9), 115.6 [q, $^1J_{CF}$ = 293.4 Hz, C(O)CF_3], 109.0, 103.8 (C-7), 88.5 (C-11), 80.8 (C-10), 79.0 (C-9a), 73.8 (q, $^2J_{CF}$ = 34.3 Hz, C-2), 21.6, 21.5 ppm. ^{19}F NMR (376.3 MHz, $CDCl_3$): δ -72.3 [C(O)CF_3], -77.2 (CF_3) ppm.

(2*R**,9a*S**)-**3f′**: ^{1}H NMR (400.1 MHz, $CDCl_3$): δ 6.47-6.41 (m, 1H, H-8), 6.30 (d, $^3J_{6,7}$ = 7.6 Hz, 1H, H-6), 6.10 (d, $^3J_{9a,9}$ = 3.8 Hz, 1H, H-9a), 5.84 (dd, $^3J_{8,9}$ = 9.6 Hz, $^3J_{9a,9}$ = 3.8 Hz, 1H, H-9), 5.33 (pseudo-t, ^3J ~ 7 Hz, 1H, H-7), 2.42 (s, 3H, Me), 2.36 (s, 3H, Me) ppm. Other signals are overlapped with those of major isomer. ^{13}C NMR (100.6 MHz, $CDCl_3$): δ 143.2, 139.4, 132.1, 126.4, 118.4, 115.0, 102.0, 79.8 ppm. Other signals are overlapped with those of major isomer or can not be seen in the spectrum due to the low concentration of minor isomer. ^{19}F NMR (376.3 MHz, $CDCl_3$): δ -74.4 [C(O)CF_3], -76.1 (CF_3) ppm.

1-(4-(4-Methylthiophenyl)-2-((4-methylthiophenyl)ethynyl)-2-(trifluoromethyl)-2*H*,9a*H*-pyrido[2,1-*b*][1,3]oxazin-3-yl)-2,2,2-trifluoroethan-1-one (3g). Obtained from pyridine **1a** (0.040 g, 0.506 mmol) and CF_3-ynone **2g** (0.256 g, 1.05 mmol). Brown powder, m.p. 120.5-123.2 °C (hexane), yield 0.274 g (96%). (2*S**,9a*S**):(2*R**,9a*S**)-isomers ratio is 92:8 (^{19}F NMR). HRMS (ESI-TOF): m/z [M+H]$^+$ Calcd for $C_{27}H_{20}F_6NO_2S_2^+$: 568.0834; found: 568.0834.

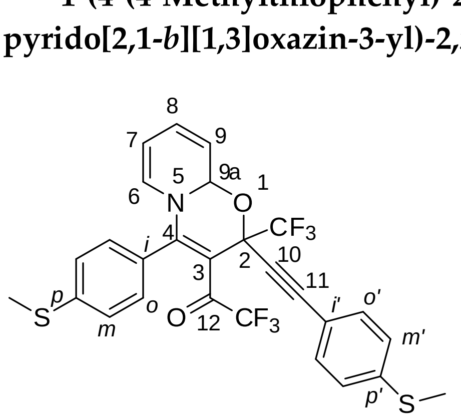

(2*S**,9a*S**)-**3g**: ^{1}H NMR (400.1 MHz, $CDCl_3$): δ 7.49-7.25 (m, 6H), 7.14 (d, 2H, ^3J = 8.5 Hz), 6.51-6.47 (m, 2H, H-8, H-6), 6.00 (dd, $^3J_{8,9}$ = 9.7 Hz, $^3J_{9a,9}$ = 3.8 Hz, 1H, H-9), 5.70 (d, $^3J_{9a,9}$ = 3.8 Hz,

1H, H-9a), 5.50 (pseudo-t, ^3J = 6.4 Hz, 1H, H-7), 2.53 (s, 3H, Me), 2.46 (s, 3H, Me) ppm. ^{1}H NMR (400.1 MHz, CD_3CN): δ 7.58-7.30 (m, 6H), 7.24 (d, 2H, ^3J = 8.7 Hz), 6.57 (d, $^3J_{6,7}$ = 7.6 Hz, 1H, H-6), 6.54 (dd, $^3J_{8,9}$ = 9.8 Hz, $^3J_{7,8}$ = 6.0 Hz, 1H, H-8), 6.00 (dd, $^3J_{8,9}$ = 9.8 Hz, $^3J_{9a,9}$ = 4.0 Hz, 1H, H-9), 5.74 (d, $^3J_{9a,9}$ = 4.0 Hz, 1H, H-9a), 5.59 (pseudo-t, ^3J = 6.8 Hz, 1H, H-7), 2.53 (s, 3H, Me), 2.47 (s, 3H, Me) ppm. ^{13}C NMR (100.6 MHz, CD_3CN): δ 180.8 (q, $^2J_{CF}$ = 34.1 Hz, C-12), 162.6 (C-4), 148.1, 142.6, 135.2, 132.9, 131.8, 129.8, 127.1, 126.8 (C-8), 126.5 (C-6), 123.8 (q, $^1J_{CF}$ = 285.8 Hz, CF_3), 117.8, 117.5, 116.7 [q, $^1J_{CF}$ = 292.2 Hz, C(O)CF_3], 108.7, 105.4 (C-7), 88.4 (C-11), 82.5 (C-10), 80.1 (C-9a), 74.5 (q, $^2J_{CF}$ = 33.7 Hz, C-2), 15.1, 14.7 ppm. ^{19}F NMR (376.3 MHz, $CDCl_3$): δ -72.3 [C(O)CF_3], -77.4 (CF_3) ppm. ^{19}F NMR (376.3 MHz, CD_3CN): δ -70.0 [C(O)CF_3], -75.5 (CF_3) ppm.

(2*R**,9a*S**)-**3g'**: ^{1}H NMR (400.1 MHz, $CDCl_3$): δ 6.47-6.41 (m, 1H, H-8), 6.30 (d, $^3J_{6,7}$ = 7.5 Hz, 1H, H-6), 6.07 (d, $^3J_{9a,9}$ = 4.0 Hz, 1H, H-9a), 5.84 (dd, $^3J_{8,9}$ = 9.7 Hz, $^3J_{9a,9}$ = 4.0 Hz, 1H, H-9), 5.34 (pseudo-t, ^3J = 6.8 Hz, 1H, H-7), 2.51 (s, 3H, Me), 2.47 (s, 3H, Me) ppm. Other signals are overlapped with those of major isomer. ^{1}H NMR (400.1 MHz, CD_3CN): δ 6.45-6.37 (m, 2H, H-8, H-9a), 6.20 (d, $^3J_{6,7}$ = 7.0 Hz, 1H, H-6), 5.87 (dd, $^3J_{8,9}$ = 9.8 Hz, $^3J_{9a,9}$ = 4.0 Hz, 1H, H-9), 5.42 (t, ^3J = 6.4 Hz, 1H, H-7), 2.52 (s, 3H, Me), 2.50 (s, 3H, Me) ppm. Other signals are overlapped with those of major isomer. ^{13}C NMR (100.6 MHz, CD_3CN): δ 145.5, 142.8, 136.9, 133.1, 129.5, 129.0, 128.2, 126.7, 126.3, 104.4, 84.7, 15.1, 14.7 ppm. Other signals are overlapped with those of major isomer or can not be seen in the spectrum due to the low concentration of minor isomer. ^{19}F NMR (376.3 MHz, $CDCl_3$): δ -74.4 [s, 3F, C(O)CF_3], -76.1 (s, 3F, CF_3) ppm. ^{19}F NMR (376.3 MHz, CD_3CN): δ -72.2 [C(O)CF_3], -74.0 (CF_3) ppm.

1-(4-(3,4-diMethylphenyl)-2-((3,4-dimethylphenyl)ethynyl)-2-(trifluoromethyl)-2*H*,9a*H*-pyrido[2,1-*b*][1,3]oxazin-3-yl)-2,2,2-trifluoroethan-1-one (3h). Obtained from pyridine **1a** (0.039 g, 0.49 mmol) and CF_3-ynone **2h** (0.232 g, 1.027 mmol). Yellow-brown powder, m.p. 72.6-74.6 °C (hexane), yield 0.215 g (83%). (2*S**,9a*S**):(2*R**,9a*S**)-isomers ratio is 92:8 (^{19}F NMR) HRMS (ESI-TOF): m/z [M+H]$^+$ Calcd for $C_{29}H_{24}F_6NO_2^+$: 532.1706; found: 532.1717.

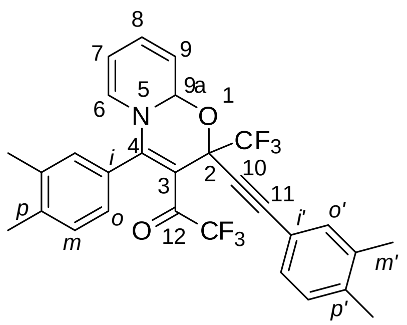

(2*S**,9a*S**)-**3h**: ^{1}H NMR (400.1 MHz, $CDCl_3$): δ 7.38-7.02 (m, 6H), 6.50-6.46 (m, 2H, H-8, H-6), 5.98 (dd, $^3J_{8,9}$ = 10.0 Hz, $^3J_{9a,9}$ = 3.9 Hz, 1H, H-9), 5.71 (d, $^3J_{9a,9}$ = 3.9 Hz, 1H, H-9a), 5.47 (pseudo-t, ^3J = 6.5 Hz, 1H, H-7), 2.34 (s, 3H, Me), 2.31 (s, 3H, Me), 2.24 (s, 3H, Me), 2.21 (s, 3H, Me) ppm. ^{13}C NMR (100.6 MHz, $CDCl_3$): δ 180.8 (q, $^2J_{CF}$ = 35.0 Hz, C-12), 160.6 (C-4), 138.3, 136.6, 133.0, 130.6, 129.5, 129.1, 126.2 (C-8), 126.1 (C-6), 122.7 (q, $^1J_{CF}$ = 286.8 Hz, CF_3), 118.3, 116.4 (C-9), 115.6 [q, $^1J_{CF}$ = 293.0 Hz, C(O)CF_3], 109.0, 103.6 (C-7), 88.6 (C-11), 80.6 (C-10), 78.9 (C-9a), 73.8 (q, $^2J_{CF}$ = 34.3 Hz, C-2), 20.0, 19.7, 19.6 (br s), 19.4 ppm. ^{19}F NMR (376.3 MHz, $CDCl_3$): δ -72.3 [C(O)CF_3], -77.3 (CF_3) ppm.

(2*R**,9a*S**)-**3h'**: ^{1}H NMR (400.1 MHz, $CDCl_3$): δ 6.44-6.40 (m, 1H, H-8), 6.31 (d, $^3J_{6,7}$ = 7.6 Hz, 1H, H-6), 6.08 (d, $^3J_{9a,9}$ = 4.0 Hz, 1H, H-9a), 5.83 (dd, $^3J_{8,9}$ = 9.9 Hz, $^3J_{9a,9}$ = 4.0 Hz, 1H, H-9), 5.31 (pseudo-t, ^3J = 7.2 Hz, 1H, H-7), 2.32 (s, 3H, Me), 2.28 (s, 3H, Me), 2.25 (s, 3H, Me) ppm. Other signals are overlapped with those of major isomer. ^{13}C NMR (100.6 MHz, $CDCl_3$): δ 142.7, 138.1, 134.8, 133.2, 131.7, 130.3, 129.7, 126.6, 126.5, 118.7, 114.9, 108.5, 101.8, 79.7, 20.2, 19.8 ppm. Other signals are overlapped with those of major isomer or can not be seen in the spectrum due to the low concentration of minor isomer. ^{19}F NMR (376.3 MHz, $CDCl_3$): δ -74.4 [C(O)CF_3], -77.3 (CF_3) ppm.

2,2,2-Trifluoro-1-(4-(4-methoxynaphthalen-1-yl)-2-((4-methoxynaphthalen-1-yl)ethynyl)-2-(trifluoromethyl)-2*H*,9a*H*-pyrido[2,1-*b*][1,3]oxazin-3-yl)ethan-1-one (3i). Obtained from pyridine **1a** (0.0405 g, 0.51 mmol) and CF_3-ynone **2i** (0.296 g, 1.06 mmol). Yellow-brown powder, m.p. 143.5-145.5 °C (hexane), yield 0.320 g (98%). (2*S**,9a*S**):(2*R**,9a*S**)-isomers ratio is 94:6. Rotamers ratio is (93:1):(4:2) (^{19}F NMR). HRMS (ESI-TOF): m/z [M+H]$^+$ Calcd for $C_{35}H_{24}F_6NO_4^+$: 636.1604; found: 636.1608.

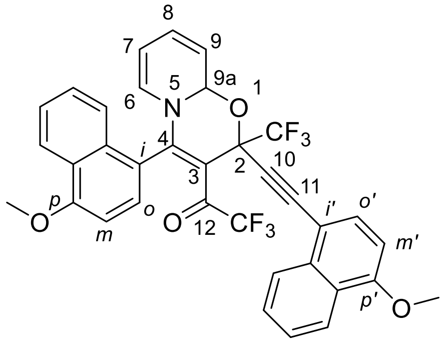

(2*S**,9a*S**)-**3i**: ^{1}H NMR (400.1 MHz, $CDCl_3$): δ 8.39-8.36 (m, 1H), 8.25-8.22 (m, 2H), 7.70-7.47 (m, 7H), 6.87 (d, ^3J = 8.1 Hz, 1H), 6.75 (d, ^3J = 8.1 Hz, 1H), 6.49 (dd, $^3J_{8,9}$ = 9.7 Hz, $^3J_{7,8}$ = 6.1 Hz, 1H, H-8), 6.11-6.02 (m, 3H, H-6, H-9, H-9a), 5.36 (pseudo-t, ^3J = 7.2 Hz, 1H, H-7), 4.08 (s, 3H, MeO), 4.01 (s, 3H, MeO) ppm. ^{13}C NMR (100.6 MHz, $CDCl_3$): δ 179.9 (q, $^2J_{CF}$ = 34.7 Hz, C-12), 160.8 (C-4), 160.2, 156.7, 137.4, 134.4, 132.3, 131.7, 128.8, 127.6, 126.3 (C-8), 126.2 (C-6), 125.93, 125.87, 125.8, 125.1, 124.8, 123.1 (q, $^1J_{CF}$ = 287.1 Hz, CF_3), 123.1, 122.1, 120.7, 117.0 (C-9), 115.7 [q, $^1J_{CF}$ = 292.9 Hz, C(O)CF_3], 111.0, 109.7, 104.0, 103.5, 103.3, 86.7 (C-11), 84.9 (C-10), 78.6 (C-9a), 74.2 (q, $^2J_{CF}$ = 33.9 Hz, C-2), 55.9, 55.6 ppm. ^{19}F NMR (376.3 MHz, $CDCl_3$): δ major rotamer -71.6 [C(O)CF_3], -76.8 (CF_3); minor rotamer -73.2 [C(O)CF_3], -78.1 (CF_3) ppm.

(2*R**,9a*S**)-**3i′**: ^{1}H NMR (400.1 MHz, $CDCl_3$): δ 8.32 (d, ^3J = 8.4 Hz, 1H), 7.96 (d, ^3J = 8.2 Hz, 1H), 6.86 (d, ^3J = 8.1 Hz, 1H), 6.81 (d, ^3J = 7.9 Hz, 1H), 4.04 (s, 3H, MeO), 4.02 (s, 3H, MeO) ppm. Other signals are overlapped with those of major isomer. ^{13}C NMR (100.6 MHz, $CDCl_3$): δ 137.5, 135.0, 129.0, 126.6, 125.0, 122.9, 56.0, 55.8 ppm. Other signals are overlapped with those of major isomer or can not be seen in the spectrum due to the low concentration of minor isomer. ^{19}F NMR (376.3 MHz, $CDCl_3$): δ major rotamer -74.1 [C(O)CF_3], -75.9 (CF_3); minor rotamer -74.3 [C(O)CF_3], -76.2 (CF_3) ppm.

2,2,2-Trifluoro-1-(4-phenyl-2-(phenylethynyl)-2-(trifluoromethyl)-8-vinyl-2*H*,9a*H*-pyrido[2,1-*b*][1,3]oxazin-3-yl)ethan-1-one (3j). Obtained from pyridine **1b** (0.054 g, 0.51 mmol) and CF_3-ynone **2a** (0.206 g, 1.04 mmol). Brown powder, m.p. 80-83 °C (hexane), yield 0.249 g (97%). (2*S**,9a*S**):(2*R**,9a*S**)-isomers ratio is 89:11 (^{19}F NMR). HRMS (ESI-TOF): m/z [M+H]$^+$ Calcd for $C_{27}H_{18}F_6NO_2^+$: 502.1246; found: 502.1246.

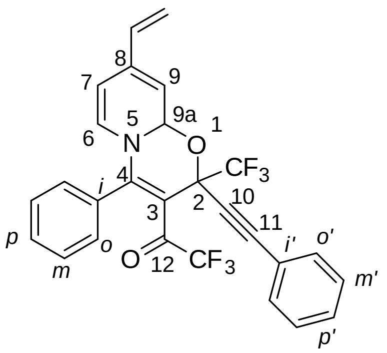

(2*S**,9a*S**)-**3j**: ^{1}H NMR (400.1 MHz, $CDCl_3$): δ 7.67-7.44 (m, 7H), 7.37-7.28 (m, 3H), 6.49 (d, $^3J_{6,7}$ = 8.0 Hz, 1H, H-6), 6.44 (dd, ^3J = 17.6 Hz, ^3J = 11.0 Hz, 1H, C<u>H</u>=CH_2), 5.90 (d, $^3J_{9a,9}$ = 4.5 Hz, 1H, H-9), 5.78 (d, $^3J_{9a,9}$ = 4.5 Hz, 1H H-9a), 5.77 (dd, $^3J_{6,7}$ = 8.0 Hz, $^4J_{7,9}$ = 1.5 Hz, 1H, H-7), 5.56 (d, ^3J = 17.6 Hz, 1H, CH=C<u>H</u>$_2$), 5.33 (d, ^3J = 11.0 Hz, 1H, CH_2, CH=C<u>H</u>$_2$) ppm. ^{13}C NMR (100.6 MHz, $CDCl_3$): δ 180.9 (q, $^2J_{CF}$ = 34.8 Hz, C-12), 160.2 (C-4), 135.2 (C, <u>C</u>H=CH_2), 134.3, 133.3, 132.1 ($C_{m,m'}$from Ar), 131.4 (q, $^1J_{CF}$ = 1.7 Hz, C-3), 129.5 (br s), 129.3, 128.2 ($C_{o,o'}$from Ar), 126.0 (C-6), 122.6, (q, $^1J_{CF}$ = 286.9 Hz, CF_3), 121.0, 116.6 (CH=<u>C</u>H_2), 114.3 (CH=<u>C</u>H_2), 115.5 [q, $^1J_{CF}$ = 292.8 Hz, C(O)CF_3], 109.3, 101.7 (C-7), 88.4 (C-11), 81.3 (C-10), 79.4 (C-9a), 73.9 (q, $^2J_{CF}$ = 34.1 Hz, C-2) ppm. ^{19}F NMR (376.3 MHz, $CDCl_3$): δ -72.5 [C(O)CF_3], -77.2 (CF_3) ppm.

(2*R**,9a*S**)-**3j′**: ^{1}H NMR (400.1 MHz, $CDCl_3$): δ 6.31 (d, $^3J_{6,7}$ = 7.9 Hz, 1H, H-6), 6.13-6.11 (m, 2H, H-9, H-9a), 5.60 (dd, ^3J = 7.9 Hz, ^3J = 1.6 Hz, 1H, H-7), 5.53 (d, ^3J = 17.4 Hz, 1H, CH=C<u>H</u>$_2$), 5.51

(d, ^3J = 17.2 Hz, 1H, CH=C$\underline{H}_2$), 5.31 (d, ^3J = 10.0 Hz, 1H, CH=C$\underline{H}_2$) ppm. Other signals are overlapped with those of major isomer. ^{13}C NMR (100.6 MHz, $CDCl_3$): δ 137.1, 135.4, 132.4, 132.2, 129.2, 128.7, 128.2, 127.2, 126.6, 126.0, 117.6, 116.5, 113.0, 99.8 ppm. Other signals are overlapped with those of major isomer or can not be seen in the spectrum due to the low concentration of minor isomer. ^{19}F NMR (376.3 MHz, $CDCl_3$): δ -74.6 [C(O)CF_3], -76.2 (CF_3) ppm.

4-Phenyl-2-(phenylethynyl)-3-(trifluoroacetyl)-2-(trifluoromethyl)-2*H*,9a*H*-pyrido[2,1-*b*][1,3]oxazine-8-carbaldehyde (3k). Obtained from pyridine **1c** (0.0475 g, 0.44 mmol) and CF_3-ynone **2a** (0.198 g, 1 mmol). Yellow powder, m.p. 77-79 °C (hexane), yield 0.178 g (80%). (2*S**,9a*S**):(2*R**,9a*S**)-isomers ratio is 89:11 (^{19}F NMR). HRMS (ESI-TOF): m/z [M+H]$^+$ Calcd for $C_{26}H_{16}F_6NO_3^+$: 504.1029; found: 504.1035.

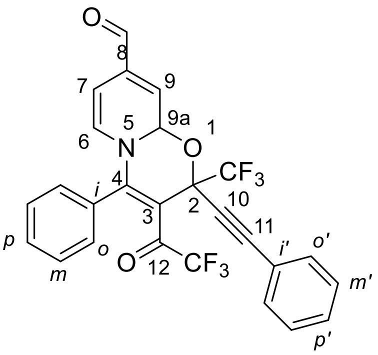

(2*S**,9a*S**)-**3k**: ^{1}H NMR (400.1 MHz, CD_3CN): 9.70 (s, 1H, CHO), δ 7.75-7.25 (m, 10H), 6.81 (d, ^3J = 4.2 Hz, 1H, H-9), 6.66 (d, ^3J = 7.8 Hz, 1H, H-6), 6.06 (d, ^3J = 4.2 Hz, 1H, H-9a), 5.94 (dd, ^3J = 7.8 Hz, ^3J = 1.5 Hz, 1H, H-7) ppm. ^{13}C NMR (100.6 MHz, CD_3CN): δ 192.2 (CHO), 181.7 (q, $^2J_{CF}$ = 34.8 Hz, C-12), 162.0 (C-4), 137.5, 134.9, 134.7, 134.0, 132.2 (q, $^3J_{CF}$ = 1.8 Hz), 130.9, 130.6 (br s), 130.2, 129.8, 129.6, 128.7, 123.7 (q, $^1J_{CF}$ = 286.0 Hz, CF_3), 121.5, 116.5 [q, $^1J_{CF}$ = 292.5 Hz, C(O)CF_3], 110.1, 99.0, 89.5 (C-11), 82.1 (C-10), 80.1 (C-9a), 75.2 (q, $^2J_{CF}$ = 34.3 Hz, C-2) ppm. ^{19}F NMR (376.3 MHz, CD_3CN): δ -70.2 [C(O)CF_3], -75.2 (CF_3) ppm. ^{19}F NMR (376.3 MHz, $CDCl_3$): δ -71.7 [C(O)CF_3], -76.0 (CF_3) ppm.

(2*S**,9a*S**)-**3k′**: ^{1}H NMR (400.1 MHz, CD_3CN): 9.66 (s, 1H, CHO), 6.52-6.46 (m, 2H), 6.37 (d, ^3J = 4.2 Hz, 1H), 5.77 (dd, ^3J = 7.7 Hz, ^3J = 1.5 Hz, 1H) ppm. Other signals are overlapped with those of major isomer. ^{13}C NMR (100.6 MHz, CD_3CN): δ 133.8, 132.9, 130.0, 129.7, 129.6, 129.4, 128.1, 115.1, 97.1, 83.8, 78.2 ppm. Other signals are overlapped with those of major isomer or can not be seen in the spectrum due to the low concentration of minor isomer. ^{19}F NMR (376.3 MHz, CD_3CN): δ -72.6 [C(O)CF_3], -74.4 (CF_3) ppm. ^{19}F NMR (376.3 MHz, $CDCl_3$): δ -73.6 [C(O)CF_3], -75.2 (CF_3) ppm.

1-(8-Acetyl-4-phenyl-2-(phenylethynyl)-2-(trifluoromethyl)-2*H*,9a*H*-pyrido[2,1-*b*][1,3]oxazin-3-yl)-2,2,2-trifluoroethan-1-one (3l). Obtained from pyridine **1d** (0.030 g, 0.25 mmol) and CF_3-ynone **2a** (0.101 g, 0.51 mmol). Yellow powder, m.p. 122.8-124.2 °C (hexane), yield 0.071 g (55%). (2*S**,9a*S**):(2*R**,9a*S**)-isomers ratio is 87:13 (^{19}F NMR). HRMS (ESI-TOF): m/z [M+H]$^+$ Calcd for $C_{27}H_{18}F_6NO_3^+$: 518.1185; found: 518.1214.

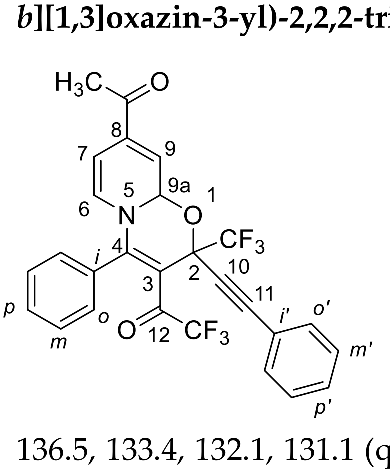

(2*S**,9a*S**)-**3l**: ^{1}H NMR (400.1 MHz, $CDCl_3$): δ 7.66-7.45 (m, 7H), 7.38-7.29 (m, 3H), 6.72 (pseudo-d, $^3J_{9a,9}$ ~ 4 Hz, 1H, H-9), 6.54 (d, $^3J_{6,7}$ = 7.8 Hz, 1H, H-6), 6.04 (dd, $^3J_{6,7}$ = 7.8, Hz, $^3J_{7,9}$ = 1.6 Hz, 1H, H-7), 5.91 (d, $^3J_{9a,9}$ = 4.2 Hz, 1H, H-9a), 2.48 (s, 3H, Me) ppm. ^{13}C NMR (100.6 MHz, $CDCl_3$): δ 195.6, 180.9 (q, $^2J_{CF}$ = 35.4 Hz, C-12), 159.3 (C-4), 136.5, 133.4, 132.1, 131.1 (q, $^1J_{CF}$ = 1.7 Hz, C-3), 129.6 (br s), 129.5, 128.3 ($C_{o,o'}$from Ar), 126.7 (C-6), 122.4 (q, $^1J_{CF}$ = 286.6 Hz, CF_3), 121.2 (C-9), 120.8 (C_i from Ar), 115.4 [q, $^1J_{CF}$ = 292.8 Hz, C(O)CF_3], 110.0, 100.3 (C-7), 89.1 (C-11), 80.9 (C-10), 78.8 (C-9a), 74.4 (q, $^2J_{CF}$ = 34.1 Hz, C-2), 25.3 ppm. ^{19}F NMR (376.3 MHz, $CDCl_3$): δ -72.7 [C(O)CF_3], -77.2 (CF_3) ppm.

(2*R**,9a*S**)-**3l′**: ^{1}H NMR (400.1 MHz, $CDCl_3$): δ 6.57 (pseudo-d, ^3J ~ 4 Hz, 1H, H-9), 6.37 (d, $^3J_{6,7}$ = 7.8 Hz, 1H, H-6), 6.28 (d, $^3J_{9a,9}$ = 4.3 Hz, 1H, H-9a), 5.88 (dd, $^3J_{6,7}$ = 7.8 Hz, $^3J_{7,9}$ = 1.5 Hz, 1H, H-7), 2.46 (s, 3H, Me) ppm. Other signals are overlapped with those of major isomer. ^{13}C NMR (100.6 MHz, $CDCl_3$): δ 136.6, 132.6, 132.3, 129.4, 128.3, 127.3, 119.9, 98.5, 29.7 ppm. Other signals are overlapped with those of major isomer or can not be seen in the spectrum due to the low concentration of minor isomer. ^{19}F NMR (376.3 MHz, $CDCl_3$): δ -74.7 [C(O)CF_3], -76.3 (CF_3) ppm.

Methyl 4-phenyl-2-(phenylethynyl)-3-(2,2,2-trifluoroacetyl)-2-(trifluoromethyl)-2*H*,9a*H*-pyrido[2,1-*b*][1,3]oxazine-8-carboxylate (3m). Obtained from pyridine **1e** (0.048 g, 0.35 mmol) and CF_3-ynone **2a** (0.147 g, 0.74 mmol). Pale yellow powder, m.p. 115.4-116.5 °C (hexane), yield 0.112 g (60%). (2*S**,9a*S**):(2*R**,9a*S**)-isomers ratio is 87:13 (^{19}F NMR). HRMS (ESI-TOF): m/z [M+H]$^+$ Calcd for $C_{27}H_{18}F_6NO_4^+$: 534.1135; found: 534.1140.

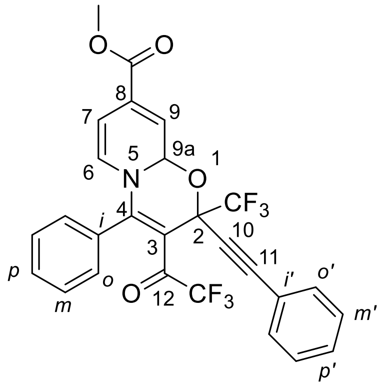

(2*S**,9a*S**)-**3m**: ^{1}H NMR (400.1 MHz, $CDCl_3$): δ 7.66-7.44 (m, 7H), 7.37-7.29 (m, 3H), 6.89 (pseudo-d, ^3J ~ 4 Hz, 1H, H-9), 6.52 (d, $^3J_{6,7}$ = 7.8 Hz, 1H, H-6), 5.99 (dd, $^3J_{6,7}$ = 7.8 Hz, $^3J_{7,9}$ = 1.5 Hz, 1H, H-7), 5.87 (d, $^3J_{9a,9}$ = 4.2 Hz, 1H, H-9a), 3.85 (s, 3H, Me) ppm. ^{13}C NMR (100.6 MHz, $CDCl_3$): δ 181.0 (q, $^2J_{CF}$ = 35.4 Hz, C-12), 164.6 (C-4), 159.2 (<u>C</u>O_2Me), 133.4, 132.1, 131.2 (q, $^1J_{CF}$ = 1.7 Hz, C-3), 130.1, 129.6 (br s), 129.5 (C-8), 128.3 ($C_{o,o'}$from Ar), 126.5 (C-6), 122.4, (q, $^1J_{CF}$ = 287.3 Hz, CF_3), 121.5 (C-9), 120.8 (C_i from Ar), 115.4 (q, $^1J_{CF}$ = 293.0 Hz, C(O)CF_3), 110.3, 101.5 (C-7), 89.0 (C-11), 80.9 (C-10), 78.8 (C-9a), 74.3 (q, $^2J_{CF}$ = 34.8 Hz, C-2), 52.5 ppm. ^{19}F NMR (376.3 MHz, $CDCl_3$): δ -72.7 [C(O)CF_3], -77.2 (CF_3) ppm.

(2*R**,9a*S**)-**3m′**: ^{1}H NMR (400.1 MHz, $CDCl_3$): δ 6.74 (pseudo-d, ^3J ~ 4 Hz, 1H, H-9), 6.35 (d, $^3J_{6,7}$ = 7.8 Hz, 1H, H-6), 6.24 (d, $^3J_{9a,9}$ = 4.3 Hz, 1H, H-9a), 5.83 (dd, $^3J_{6,7}$ = 7.8 Hz, $^3J_{7,9}$ = 1.5 Hz, 1H, H-7), 3.84 (s, 3H, Me) ppm. Other signals are overlapped with those of major isomer. ^{13}C NMR (100.6 MHz, $CDCl_3$): δ 132.6, 132.3, 130.3, 129.4, 128.3, 127.1, 120.1, 99.8, 52.5 ppm. Other signals are overlapped with those of major isomer or can not be seen in the spectrum due to the low concentration of minor isomer. ^{19}F NMR (376.3 MHz, $CDCl_3$): δ -74.7 [C(O)CF_3], -76.4 (CF_3) ppm.

(2*S,9a*S**)-1-(4,6-diPhenyl-2-(phenylethynyl)-2-(trifluoromethyl)-2*H*,9a*H*-pyrido[2,1-*b*][1,3]oxazin-3-yl)-2,2,2-trifluoroethan-1-one (3n)**. Obtained from pyridine **1f** (0.079 g, 0.51 mmol) and CF_3-ynone **2a** (0.204 g, 1.03 mmol). Orange powder, m.p. 90-91 °C (hexane), yield 0.168 g (60%). HRMS (ESI-TOF): m/z [M+H]$^+$ Calcd for $C_{31}H_{20}F_6NO_2^+$: 552.1393; found: 552.1393.

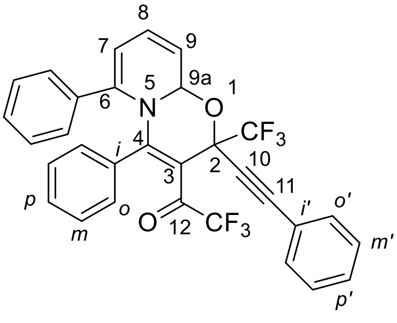

(2*S**,9a*S**)-**3o**: ^{1}H NMR (400.1 MHz, $CDCl_3$): δ 7.46-7.43 (m, 2H), 7.38-7.28 (m, 3H), 7.17-6.99 (m, 5H), 6.93 (br s, 5H), 6.61 (ddd, $^3J_{8,9}$ = 9.7 Hz, $^3J_{7,8}$ = 6.1 Hz, $^4J_{8,9a}$ = 0.8 Hz, 1H, H-8), 6.00 (ddd, $^3J_{8,9}$ = 9.7 Hz, $^3J_{9a,9}$ = 4.2 Hz, $^4J_{7,9}$ = 0.7 Hz, 1H, H-9), 5.84 (d, $^3J_{9a,9}$ = 4.2 Hz, 1H, H-9a), 5.43 (dd, $^3J_{7,8}$ = 6.1 Hz, $^3J_{7,9}$ = 0.7 Hz, 1H, H-7) ppm. ^{13}C NMR (100.6 MHz, $CDCl_3$): δ 184.2 (q, $^2J_{CF}$ = 36.1 Hz, C-12), 157.3 (C-4), 139.2, 136.4, 134.7, 133.9, 132.5, 132.1, 131.4, 129.5, 128.9, 128.4, 128.3, 127.8, 127.7, 127.2, 122.8 (q, $^1J_{CF}$ = 285.7 Hz, CF_3), 120.7, 115.0 [q, $^1J_{CF}$ = 293.6 Hz, C(O)CF_3], 114.7, 106.7, 89.7 (C-11), 81.5 (C-9a), 81.2 (C-10), 74.5 (q, $^2J_{CF}$ = 34.8 Hz, C-2) ppm. ^{19}F NMR (376.3 MHz, $CDCl_3$): δ -73.9 [C(O)CF_3], -76.0 (CF_3) ppm.

1-(9-Bromo-4-phenyl-2-(phenylethynyl)-2-(trifluoromethyl)-2*H*,9a*H*-pyrido[2,1-*b*][1,3]oxazin-3-yl)-2,2,2-trifluoroethan-1-one (3p). Major (9-Br)-regioisomer, obtained as a mixture (1:5) with minor (7-Br)-regioisomer **(3o)** from pyridine **1g** (0.082 g, 0.52 mmol) and CF_3-ynone **2a** (0.208 g, 1.05 mmol). Yellow powder, m.p. 76.0-77.8 °C (hexane), yield 0.153 g (53%). (2*S**,9a*S**):(2*R**,9a*S**)-isomers ratio of **3p** is 80:20 (^{19}F NMR). HRMS (ESI-TOF) for the mixture of **3o** and **3p**: m/z $[M+H]^+$ Calcd for $C_{25}H_{15}F_6BrNO_2^+$: 554.0185; found: 554.0190.

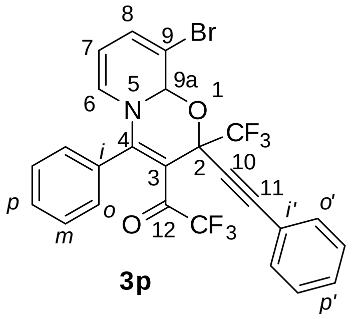

3p

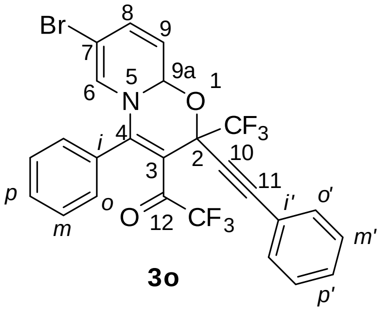

3o

(2*S**,9a*S**)-**3p**: ^{1}H NMR (400.1 MHz, $CDCl_3$): δ 7.65-7.30 (m, 10H), 6.80 (d, $^3J_{6,7}$ = 6.6 Hz, 1H, H-6), 6.45 (d, $^3J_{7,8}$ = 7.5 Hz, 1H, H-8), 5.79 (s, 1H, H-9a), 5.38 (pseudo-t, ^3J ~ 7 Hz, 1H, H-7) ppm. ^{13}C NMR (100.6 MHz, $CDCl_3$): δ 181.1 (q, $^2J_{CF}$ = 35.6 Hz, C-12), 158.7 (C-4), 133.4, 132.3, 132.1, 131.1 (q, $^3J_{CF}$ = 1.8 Hz, C-3), 129.6 (br s), 129.4 (C-8), 128.7 (C-6), 128.3, 125.2, 122.4 (q, $^1J_{CF}$ = 286.4 Hz, CF_3), 121.0 (C_i from Ar), 117.4 (C-9), 115.4 [q, $^1J_{CF}$ = 292.6 Hz, C(O)CF_3], 109.9, 103.3 (C-7), 89.2 (C-11), 82.9 (C-10), 80.5 (C-9a), 74.7 (q, $^2J_{CF}$ = 34.8 Hz, C-2) ppm. ^{19}F NMR (376.3 MHz, $CDCl_3$): δ -72.7 [C(O)CF_3], -76.8 (CF_3) ppm.

(2*R**,9a*S**)-**3p′**: ^{1}H NMR (400.1 MHz, $CDCl_3$): δ 6.75 (d, $^3J_{6,7}$ = 6.7 Hz, 1H, H-6), 6.30 (d, $^3J_{7,8}$ = 7.6 Hz, 1H, H-8), 6.16 (s, 1H, H-9a), 5.25 (pseudo-t, ^3J ~ 7 Hz, 1H, H-7) ppm. Other signals are overlapped with those of major isomer. ^{13}C NMR of (2*R**,9a*S**)-**3p′** and ^{13}C NMR of (2*S**,9a*S**)-**3o** (100.6 MHz, $CDCl_3$): δ 158.6 (C-4), 152.3, 133.5, 132.6, 132.2, 130.8, 131.0 (q, $^3J_{CF}$ = 1.3 Hz, C-3), 130.5, 129.5, 129.3, 128.94, 128.91, 128.36, 128.31, 125.7, 121.5, 121.3, 120.8, 110.8, 101.8, 89.02 and 88.98 (C-11), 83.53 and 83.49 (C-10), 81.3 and 80.9 (C-9a), 77.8 ppm. Due to low concentration and equal amounts of (2*R**,9a*S**)-**3p′** and ^{13}C NMR of (2*S**,9a*S**)-**3o** assignment of their signals can not be done. ^{13}C NMR are reported together. Other signals are overlapped with those of major isomer **3p** or can not be seen in the spectrum due to the low concentration of minor isomers. ^{19}F NMR (376.3 MHz, $CDCl_3$): δ -74.7 [C(O)CF_3], -75.9 (CF_3) ppm.

1-(7-Bromo-4-phenyl-2-(phenylethynyl)-2-(trifluoromethyl)-2*H*,9a*H*-pyrido[2,1-*b*][1,3]oxazin-3-yl)-2,2,2-trifluoroethan-1-one (3p). Minor (7-Br)-regioisomer, obtained as a mixture with major (9-Br)-regioisomer **(3q)** (see above). (2*S**,9a*S**):(2*R**,9a*S**)-isomers ratio is 78:22 (^{19}F NMR). HRMS (ESI-TOF) for the mixture of **3o** and **3p**: m/z $[M+H]^+$ Calcd for $C_{25}H_{15}F_6BrNO_2^+$: 554.0185; found: 554.0190.

(2*S**,9a*S**)-**3o**: ^{1}H NMR (400.1 MHz, $CDCl_3$): δ 6.65 (s, 1H, H-6), 6.55 (d, $^3J_{8,9}$ = 10.1 Hz, 1H, H-8), 5.98 (dd, $^3J_{8,9}$ = 10.1 Hz, $^3J_{9a,9}$ = 3.9 Hz, 1H, H-9), 5.71 (d, $^3J_{9,9a}$ = 3.9 Hz, 1H, H-9a) ppm. Other signals are overlapped with those of major isomer. ^{13}C NMR (100.6 MHz, $CDCl_3$) : See above in **3p** section. ^{19}F NMR (376.3 MHz, $CDCl_3$): δ -72.7 [C(O)CF_3], -77.1 (CF_3) ppm.

(2*R**,9a*S**)-**3o′**: ^{1}H NMR (400.1 MHz, $CDCl_3$): δ 6.62 (s, 1H, H-6), 6.40 (d, $^3J_{8,9}$ = 10.1 Hz, 1H, H-8), 5.84 (dd, $^3J_{8,9}$ = 10.1 Hz, $^3J_{9a,9}$ = 4.2 Hz, 1H, H-9), 6.06 (d, $^3J_{9,9a}$ = 4.2 Hz, 1H, H-9a) ppm. Other signals are overlapped with those of major isomer. ^{13}C NMR (100.6 MHz, $CDCl_3$): can not be seen in the spectrum due to the low concentration of minor isomer. ^{19}F NMR (376.3 MHz, $CDCl_3$): δ -74.7 [C(O)CF_3], -75.6 (CF_3) ppm.

1-(7-Acetyl-4-phenyl-2-(phenylethynyl)-2-(trifluoromethyl)-2*H*,9a*H*-pyrido[2,1-*b*][1,3]oxazin-3-yl)-2,2,2-trifluoroethan-1-one (3q). Major (7-Ac)-regioisomer, obtained as a mixture (2:1) with minor (9-Ac)-regioisomer **(3r)** from pyridine **1h** (0.065 g, 0.54 mmol) and CF_3-ynone **2a** (0.214 g, 1.08 mmol). Yellow powder, m.p. 114.4-115.3 °C (hexane), yield 0.224 g (80%). (2*S**,9a*S**):(2*R**,9a*S**)-isomers ratio is 83:17 (^{1}H NMR). HRMS (ESI-TOF) for the mixture of **3q** and **3r**: m/z $[M+H]^+$ Calcd for $C_{27}H_{18}F_6NO_3^+$: 518.1185; found: 518.1196.

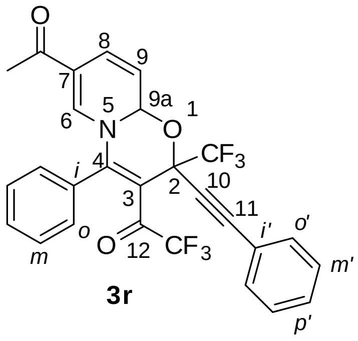

3r

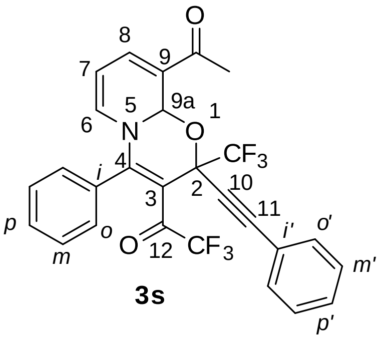

3s

(2*S**,9a*S**)-**3q**: ^{1}H NMR (400.1 MHz, $CDCl_3$): δ 7.69-7.29 (m, 11H), 7.10 (d, $^3J_{8,9}$ = 10.1 Hz, 1H, H-8), 6.00 (dd, $^3J_{8,9}$ = 10.1 Hz, $^3J_{9a,9}$ = 3.6 Hz, 1H, H-9), 5.88 (d, $^3J_{9a,9}$ = 3.6 Hz, 1H, H-9a), 2.12 (s, 3H, Me) ppm. ^{13}C NMR (100.6 MHz, $CDCl_3$): δ 193.1, 182.1 (q, $^2J_{CF}$ = 36.3 Hz, C-12), 156.2 (C-4), 133.7, 133.6, 132.1, 129.9, 129.7, 128.4 (C-8), 130.4 (q, $^3J_{CF}$ = 1.3 Hz, C-3), 124.2 (C-6), 122.2 (q, $^1J_{CF}$ = 286.0 Hz, CF_3), 120.4 (C_i from Ar), 115.3 (C-9), 115.0 (q, $^1J_{CF}$ = 293.0 Hz, C(O)CF_3), 102.3, 90.1 (C-11), 80.3 (C-10), 79.1 (C-9a), 74.2 (q, $^2J_{CF}$ = 34.8 Hz, C-2), 25.0 ppm. ^{19}F NMR (376.3 MHz, $CDCl_3$): δ -73.7 [C(O)CF_3], -76.8 (CF_3) ppm.

(2*R**,9a*S**)-**3q′**: ^{1}H NMR (400.1 MHz, $CDCl_3$): δ 7.17 (s, 1H, H-6), 7.05 (d, $^3J_{8,9}$ = 10.2 Hz, 1H, H-8), 6.17 (d, $^3J_{9a,9}$ = 3.6 Hz, 1H, H-9a), 5.90 (dd, $^3J_{8,9}$ = 10.2 Hz, $^3J_{9a,9}$ = 3.6 Hz, 1H, H-9), 2.06 (s, 3H, Me) ppm. ^{13}C NMR (100.6 MHz, $CDCl_3$): δ 193.0, 131.8, 128.3, 114.5, 24.9 ppm. Other signals are overlapped with those of major isomer or can not be seen in the spectrum due to the low concentration of minor isomer. ^{19}F NMR (376.3 MHz, $CDCl_3$): δ -74.9 [C(O)CF_3], -75.5 (CF_3) ppm.

1-(9-Acetyl-4-phenyl-2-(phenylethynyl)-2-(trifluoromethyl)-2*H*,9a*H*-pyrido[2,1-*b*][1,3]oxazin-3-yl)-2,2,2-trifluoroethan-1-one (3r). Minor (9-Ac)-regioisomer, obtained as a mixture with major (7-Ac)-regioisomer **(3q)** (see above). (2*S**,9a*S**):(2*R**,9a*S**)-isomers ratio is 87:13 (^{1}H NMR). HRMS (ESI-TOF) for the mixture of **3q** and **3r**: m/z $[M+H]^+$ Calcd for $C_{27}H_{18}F_6NO_3^+$: 518.1185; found: 518.1196.

(2*S**,9a*S**)-**3r**: ^{1}H NMR (400.1 MHz, $CDCl_3$): δ 7.69-7.29 (m, 11H), 6.65 (d, $^3J_{6,7}$ = 7.3 Hz, 1H, H-6), 6.20 (s, 1H, H-9a), 5.62 (pseudo-t, ^3J ~ 7 Hz, 1H, H-7), 2.47 (s, 3H, Me) ppm. ^{13}C NMR (100.6 MHz, $CDCl_3$): δ 194.6, 181.5 (q, $^2J_{CF}$ = 35.8 Hz, C-12), 158.0 (C-4), 134.2, 133.5, 132.1, 131.0 (q, $^3J_{CF}$ = 1.7 Hz, C-3), 129.4, 128.3, (C-8), 124.9 (C-6), 122.3 (q, $^1J_{CF}$ = 286.6 Hz, CF_3), 120.9 (C_i from Ar), 115.7 (C-9), 115.2 (q, $^1J_{CF}$ = 293.0 Hz, C(O)CF_3), 89.6 (C-11), 80.0 (C-10), 77.7 (C-9a), 74.4 (q, $^2J_{CF}$ = 34.5 Hz, C-2), 25.7 ppm. ^{19}F NMR (376.3 MHz, $CDCl_3$): δ -73.0 [C(O)CF_3], -76.8 (CF_3) ppm.

(2*R**,9a*S**)-**3r′**: ^{1}H NMR (400.1 MHz, $CDCl_3$): δ 6.58 (s, 1H, H-9a), 6.51 (d, $^3J_{6,7}$ = 7.4 Hz, 1H, H-6), 5.62 (pseudo-t, ^3J ~ 7 Hz, 1H, H-7), 2.43 (s, 3H, Me) ppm. Other signals are overlapped with those of major isomer. ^{13}C NMR (100.6 MHz, $CDCl_3$): δ 194.7, 134.3, 133.0, 132.2, 129.5, 114.9, 25.5 ppm. Other signals are overlapped with those of major isomer or can not be seen in the spectrum due to the low concentration of minor isomer. ^{19}F NMR (376.3 MHz, $CDCl_3$): δ -74.9 [C(O)CF_3], -76.4 (CF_3) ppm

4-Phenyl-2-(phenylethynyl)-3-(2,2,2-trifluoroacetyl)-2-(trifluoromethyl)-2*H*,9a*H*-pyrido[2,1-*b*][1,3]oxazine-7-carbonitrile (3s). Major (7-CN)-regioisomer, obtained as a mixture (2.5:1) with minor (9-CN)-regioisomer **(3t)** from pyridine **1i** (0.054 g, 0.5 mmol) and CF_3-ynone **2a** (0.208 g, 1.05 mmol). Yellow powder, m.p. 95-96 °C (hexane), yield 0.165 g (66%). (2*S**,9a*S**):(2*R**,9a*S**)-isomers ratio is 76:24 (^{19}F NMR). HRMS (ESI-TOF) for the mixture of **3s** and **3t**: m/z $[M+H]^+$ Calcd for $C_{26}H_{15}F_6N_2O_2^+$: 501.1032; found: 501.1055.

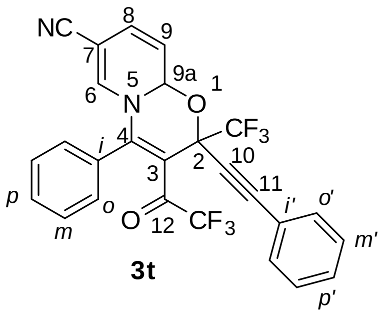

3t

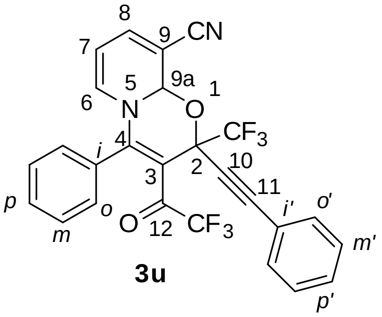

3u

(2*S**,9a*S**)-**3s**: ^{1}H NMR (400.1 MHz, $CDCl_3$): δ 7.69-7.28 (m, 10H), 6.98 (s, 1H, H-6), 6.49 (d, $^3J_{8,9}$ = 10.0 Hz, 1H, H-8), 6.00 (dd, $^3J_{8,9}$ = 10.0 Hz, $^3J_{9a,9}$ = 3.5 Hz, 1H, H-9), 5.92-5.89 (m, 1H, H-9a) ppm. ^{13}C NMR (100.6 MHz, $CDCl_3$): δ 182.0 (q, $^2J_{CF}$ = 37.0 Hz, C-12), 154.5 (C-4), 136.0, 133.7, 132.0, 130.0, 129.9, 128.4, 130.3 (q, $^4J_{CF}$ = 1.7 Hz, C-3), 124.4 (C-6), 122.1 (q, $^1J_{CF}$ = 286.4 Hz, CF_3), 120.2 (C_i from Ar), 116.3 (C-9), 114.9 [q, $^1J_{CF}$ = 293.0 Hz, C(O)CF_3], 113.3 (CN), 101.4, 88.5 (C-11), 79.9 (C-10), 78.3 (C-9a), 74.3 (q, $^2J_{CF}$ = 34.7 Hz, C-2) ppm. ^{19}F NMR (376.3 MHz, $CDCl_3$): δ -73.9 [C(O)CF_3], -76.8 (CF_3) ppm.

(2*R**,9a*S**)-**3s′**: ^{1}H NMR (400.1 MHz, $CDCl_3$): δ 6.87 (s, 1H, H-6), 6.44 (d, $^3J_{8,9}$ = 10.0 Hz, 1H, H-8), 6.20 (d, $^3J_{9a,9}$ = 3.6 Hz, 1H, H-9a) ppm. ^{13}C NMR (100.6 MHz, $CDCl_3$): δ 149.8, 136.5, 133.1, 132.2, 120.8, 115.6, 112.4 (CN), 100.3, 86.8, 80.1, 78.8 (q, $^4J_{CF}$ = 3.7 Hz, C-10), 72.1 (q, $^2J_{CF}$ = 32.4 Hz, C-2) ppm. Other signals are overlapped with those of major isomer or can not be seen in the spectrum due to the low concentration of minor isomer. ^{19}F NMR (376.3 MHz, $CDCl_3$): δ -75.0 [C(O)CF_3], -76.5 (CF_3) ppm.

4-Phenyl-2-(phenylethynyl)-3-(2,2,2-trifluoroacetyl)-2-(trifluoromethyl)-2*H*,9a*H*-pyrido[2,1-*b*][1,3]oxazine-7-carbonitrile (3t). Minor (9-CN)-regioisomer, obtained as a mixture with major (7-CN)-regioisomer **(3s)** (see above). (2*S**,9a*S**):(2*R**,9a*S**)-isomers ratio is 86:14 (^{19}F NMR). HRMS (ESI-TOF) for the mixture of **3s** and **3t**: m/z $[M+H]^+$ Calcd for $C_{26}H_{15}F_6N_2O_2^+$: 501.1032; found: 501.1055.

(2*S**,9a*S**)-**3t**: ^{1}H NMR (400.1 MHz, $CDCl_3$): δ 7.69-7.28 (m, 10H), 7.14 (d, $^3J_{7,8}$ = 6.5 Hz, 1H, H-8), 6.64 (d, $^3J_{6,7}$ = 7.5 Hz, 1H, H-6), 5.92-5.89 (m, 1H, H-9a), 5.54 (pseudo-t, ^3J ~ 7 Hz, 1H, H-7) ppm. ^{13}C NMR (100.6 MHz, $CDCl_3$): δ 182.5 (q, $^2J_{CF}$ = 37.2 Hz, C-12), 156.4 (C-4), 139.3, 133.6, 132.1, 131.3, 129.7, 129.6, 129.3, 128.3, 124.3 (C-6), 122.1 (q, $^1J_{CF}$ = 286.6 Hz, CF_3), 120.4 (C_i from Ar), 117.2 (C-9), 115.1 [q, $^1J_{CF}$ = 293.2 Hz, C(O)CF_3], 112.5 (CN), 98.7, 90.7 (C-11), 87.7, 79.8 (C-10), 78.2 (C-9a), 74.5 (q, $^2J_{CF}$ = 35.6 Hz, C-2) ppm. ^{19}F NMR (376.3 MHz, $CDCl_3$): δ -73.5 [C(O)CF_3], -76.8 (CF_3) ppm.

(2*R**,9a*S**)-**3t′**: ^{1}H NMR (400.1 MHz, $CDCl_3$): δ 7.09 (d, $^3J_{7,8}$ = 6.4 Hz, 1H, H-8), 6.23 (s, 1H, H-9a), ppm. Other signals are overlapped with those of major isomer. ^{13}C NMR (100.6 MHz, $CDCl_3$): δ 149.5, 132.9, 132.7, 97.8, 86.3, 90.4. Other signals are overlapped with those of major isomer or can not be seen in the spectrum due to the low concentration of minor isomer. ^{19}F NMR (376.3 MHz, $CDCl_3$): δ -74.9 [C(O)CF_3], -76.5 (CF_3) ppm

28 Jan 2019

FW	475.3826	Formula	$C_{25}H_{15}F_6NO_2$

Acquisition Time (sec)	2.9295	Comment	Imported from UXNMR.			Date	14 Apr 2018 22:33:46		
File Name	C:\BM_DATA\BM-1344\BM-1344_001001r			Frequency (MHz)	400.13	Nucleus	1H	Number of Transients	8
Original Points Count	16384	Points Count	65536	Pulse Sequence	zg30	Solvent	CHLOROFORM-D		
Sweep Width (Hz)	5592.84	Temperature (degree C)	27.000						

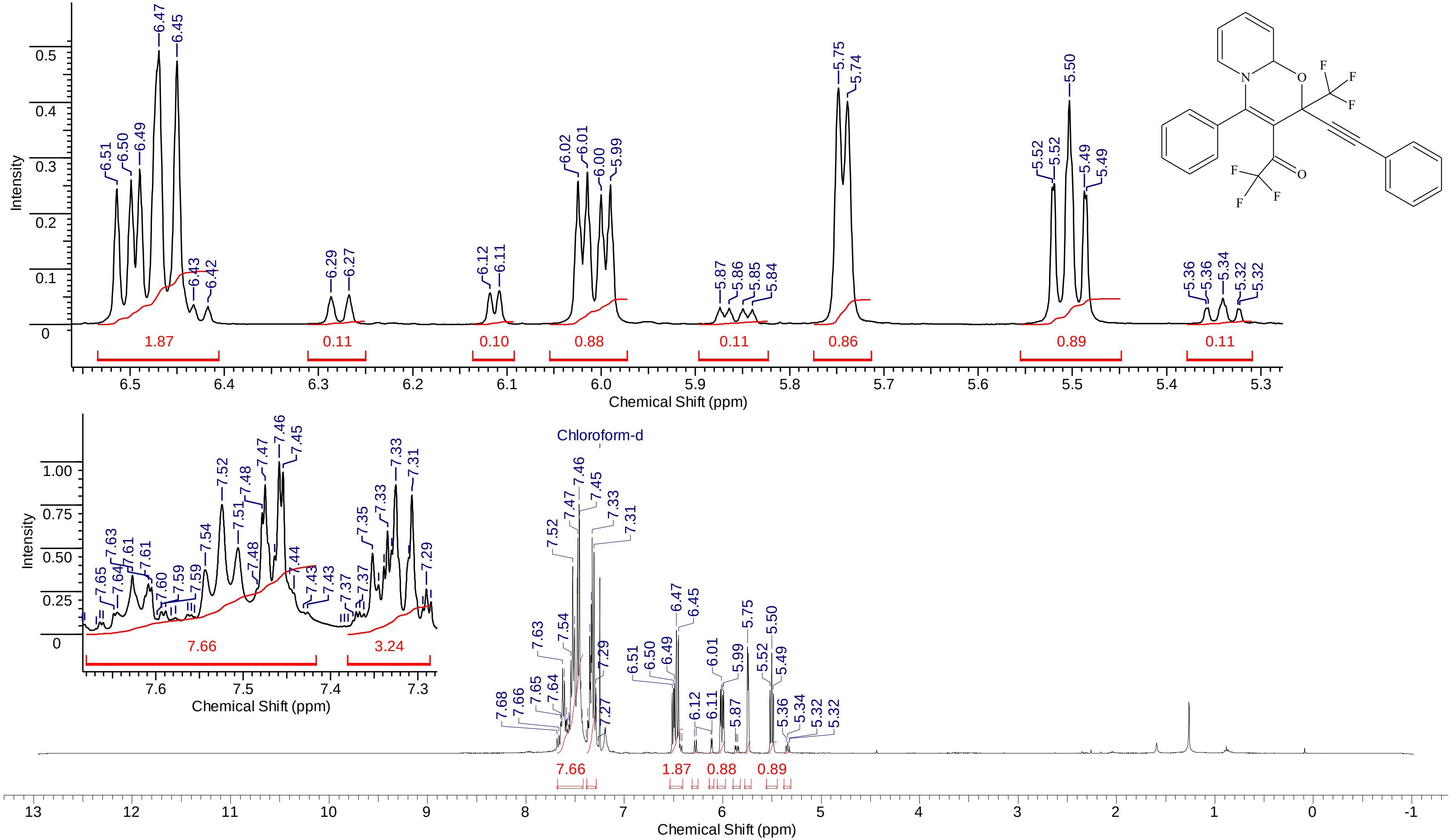

^{1}H NMR spectrum of **3a** (400.1 MHz, $CDCl_3$)

28 Jan 2019

FW	475.3826	Formula	$C_{25}H_{15}F_6NO_2$

Acquisition Time (sec)	0.6783	Comment	Imported from UXNMR.			Date	14 Apr 2018 22:39:56		
File Name	C:\BM_DATA\BM-1344\BM-1344_002001r			Frequency (MHz)	100.61	Nucleus	13C	Number of Transients	128
Original Points Count	16384	Points Count	131072	Pulse Sequence	zgpg30	Solvent	CHLOROFORM-D		
Sweep Width (Hz)	24154.59	Temperature (degree C)	27.000						

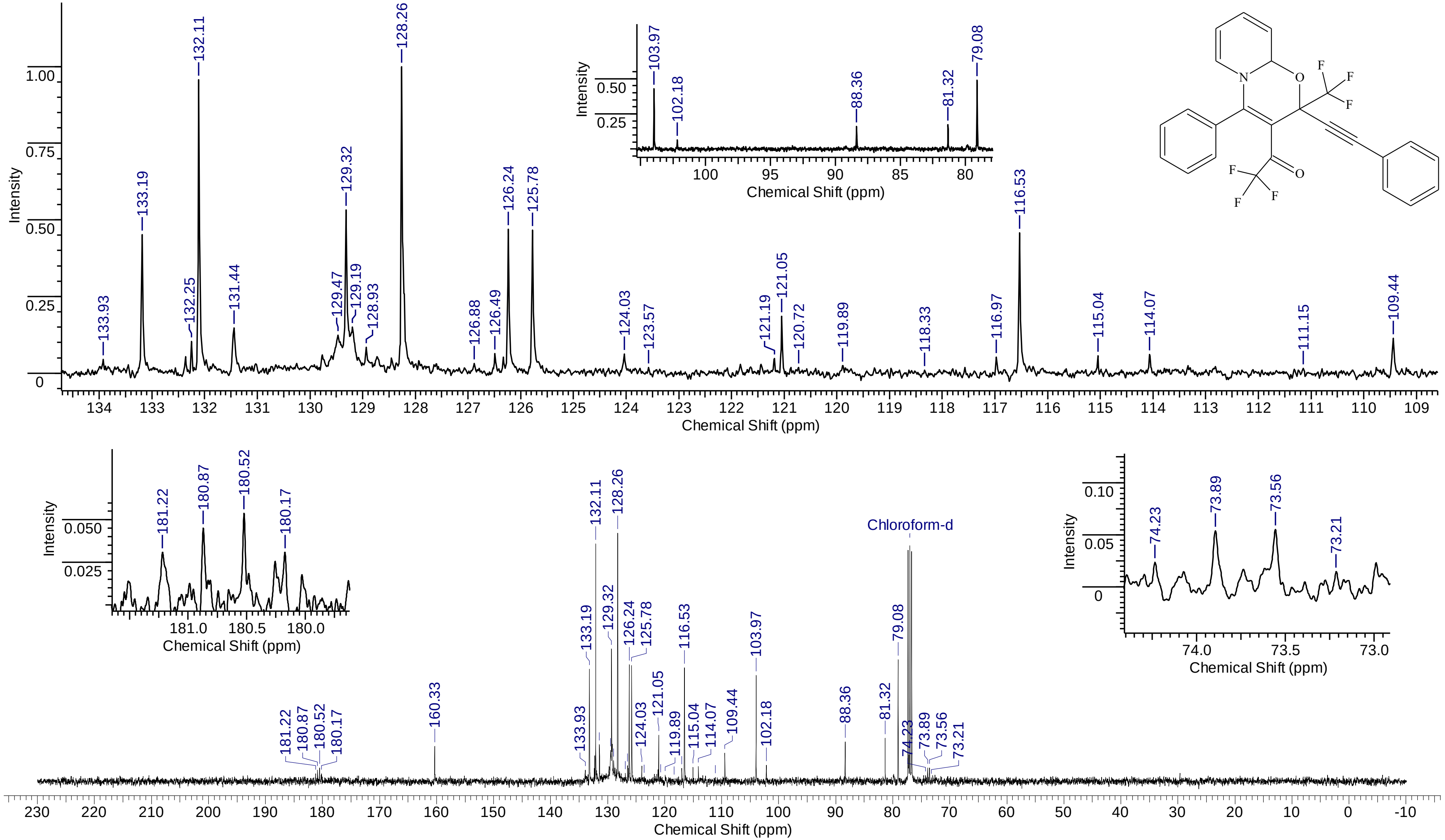

^{13}C NMR spectrum of **3a** (100.6 MHz, $CDCl_3$)

28 Jan 2019

FW	475.3826	***Formula***	$C_{25}H_{15}F_{6}NO_{2}$

Acquisition Time (sec)	0.7340	***Date***	Apr 13 2018	***File Name***	I:\SPEC_BM_F_2018.04.27\BM-1344-R_20180413_01\FLUORINE_01		
Frequency (MHz)	376.31	***Nucleus***	19F	***Number of Transients***	16	***Original Points Count***	65536
Points Count	65536	***Pulse Sequence***	s2pul	***Solvent***	CHLOROFORM-D		
Sweep Width (Hz)	89285.71	***Temperature (degree C)***	22.000				

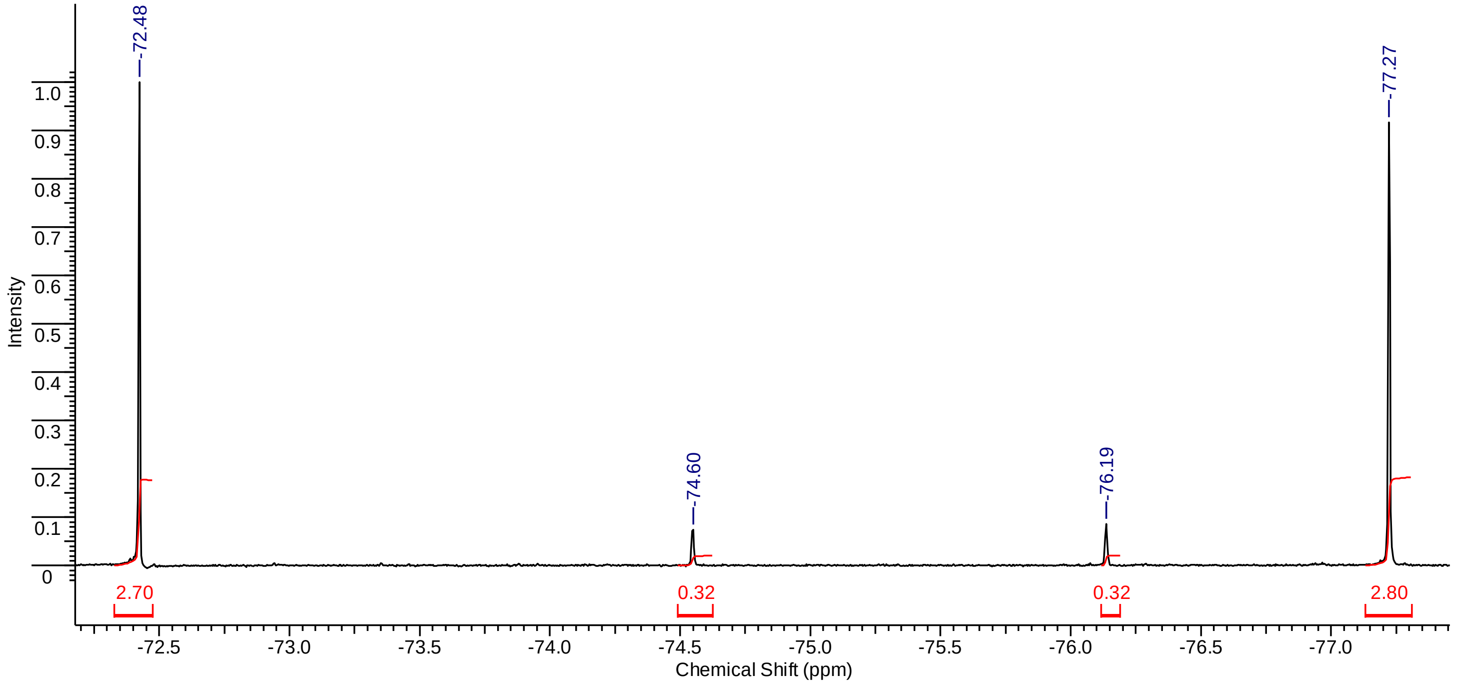

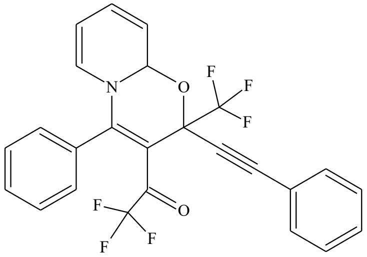

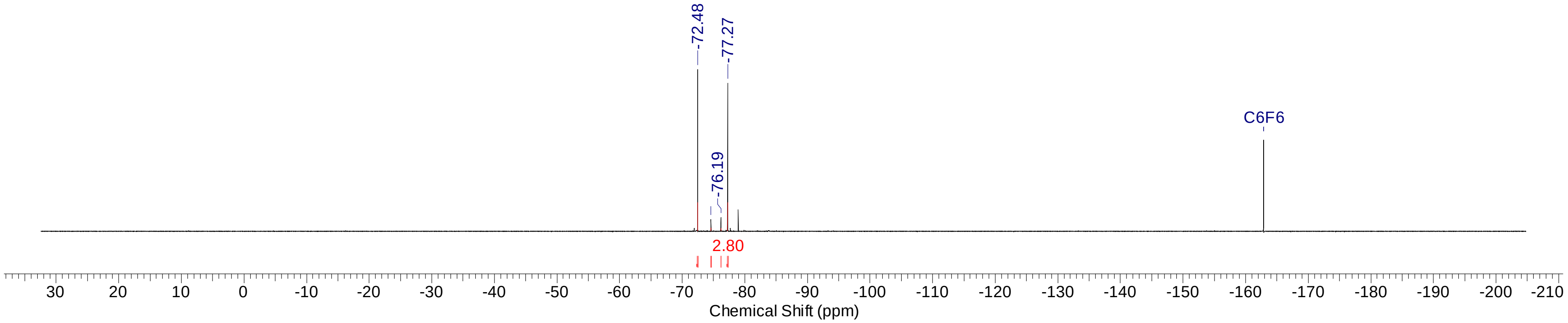

^{19}F NMR spectrum of **3a** (376.3 MHz, $CDCl_3$)

28 Jan 2019

FW	587.5952	Formula	$C_{33}H_{31}F_6NO_2$

Acquisition Time (sec)	4.0894	Comment	Imported from UXNMR.	Date	10 Nov 2018 13:08:10
File Name	C:\DOCS\BM\SPEC_H,C_I-XII.2018\BM-1430-C_001001r	Frequency (MHz)	400.13	Nucleus	1H
Number of Transients	8	Original Points Count 32768	Points Count 131072	Pulse Sequence	zg30
Solvent	CHLOROFORM-D		Sweep Width (Hz) 8012.82	Temperature (degree C)	27.000

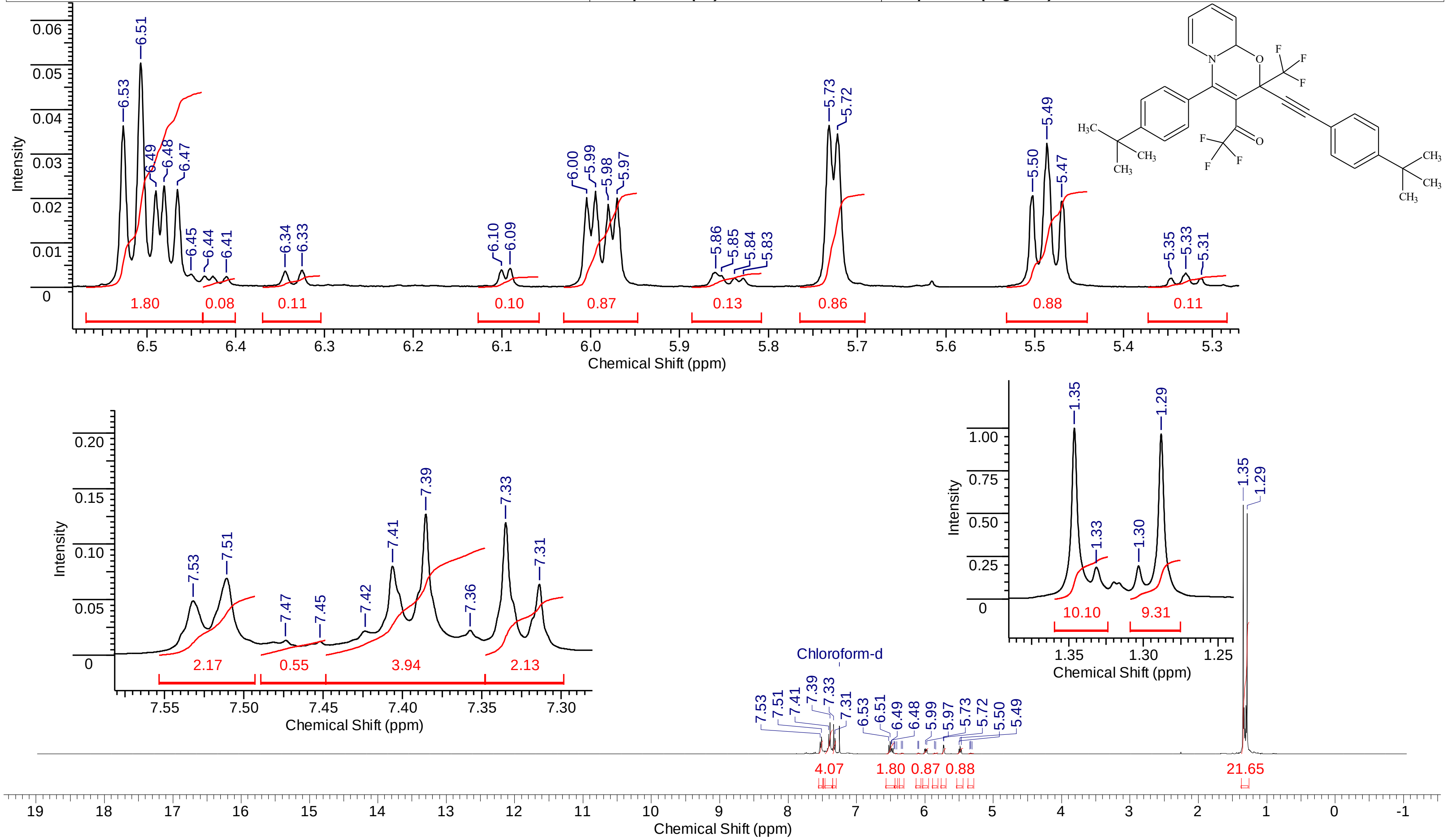

^{1}H NMR spectrum of **3b** (400.1 MHz, $CDCl_3$)

FW	587.5952	Formula	$C_{33}H_{31}F_6NO_2$

Acquisition Time (sec)	0.6783	Comment	Imported from UXNMR.			Date	10 Nov 2018 13:23:28
File Name	C:\DOCS\BM\SPEC_H,C_I-XII.2018\BM-1430-C_002001r			Frequency (MHz)	100.61	Nucleus	13C
Number of Transients	368	Original Points Count	16384	Points Count	131072	Pulse Sequence	zgpg30
Solvent	CHLOROFORM-D			Sweep Width (Hz)	24154.59	Temperature (degree C)	27.000

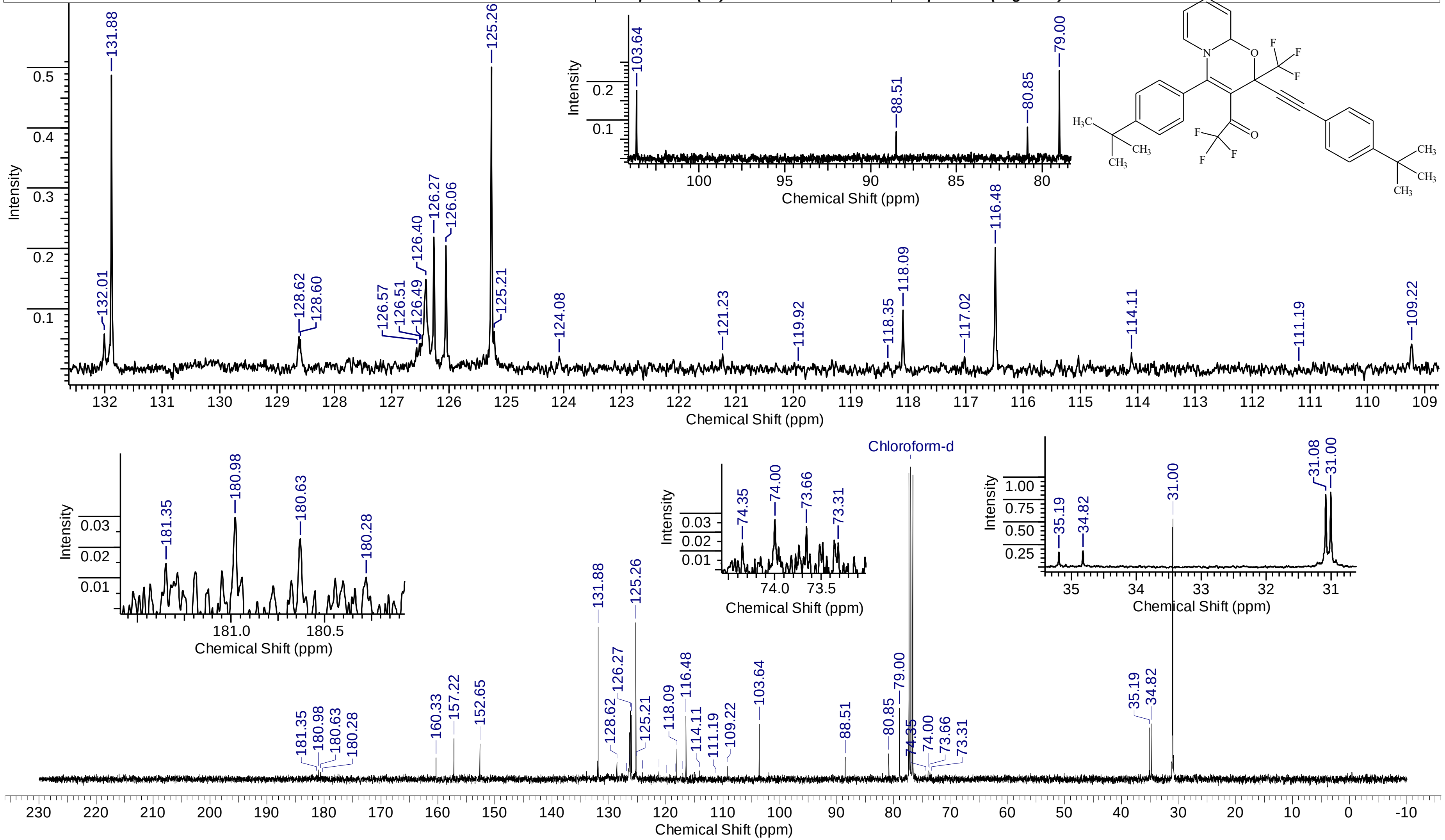

^{13}C NMR spectrum of **3b** (100.6 MHz, $CDCl_3$)

29 Jan 2019

Acquisition Time (sec)	2.0000	***Date***	Nov 12 2018	***File Name***	C:\DOCS\BM\SPEC_BM_F_2018.12.25\BM-1430-C_20181112_01\FLUORINE_01		
Frequency (MHz)	376.31	***Nucleus***	19F	***Number of Transients***	16	***Original Points Count***	178571
Points Count	262144	***Pulse Sequence***	s2pul	***Solvent***	CHLOROFORM-D		
Sweep Width (Hz)	89285.71	***Temperature (degree C)***	22.000				

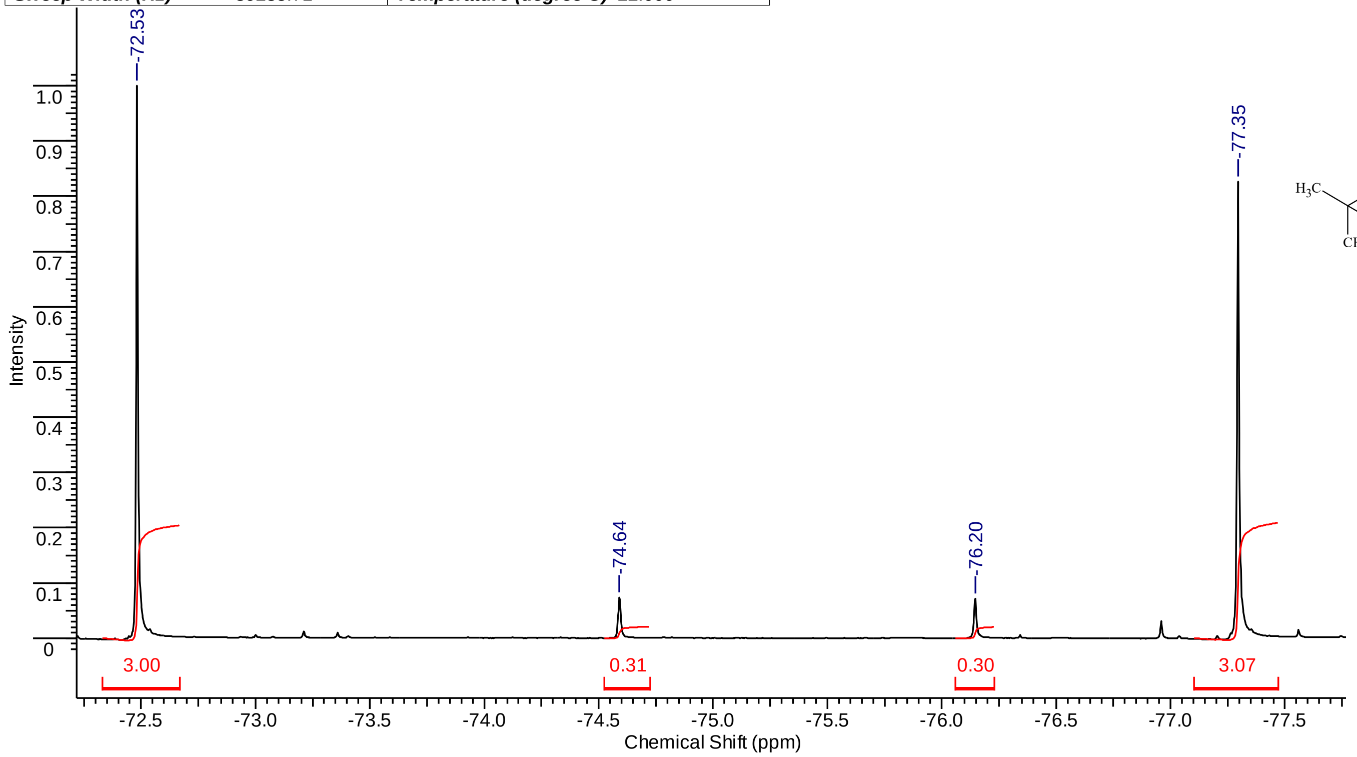

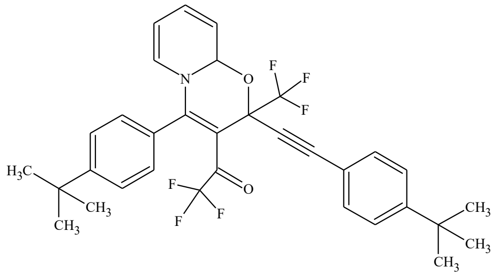

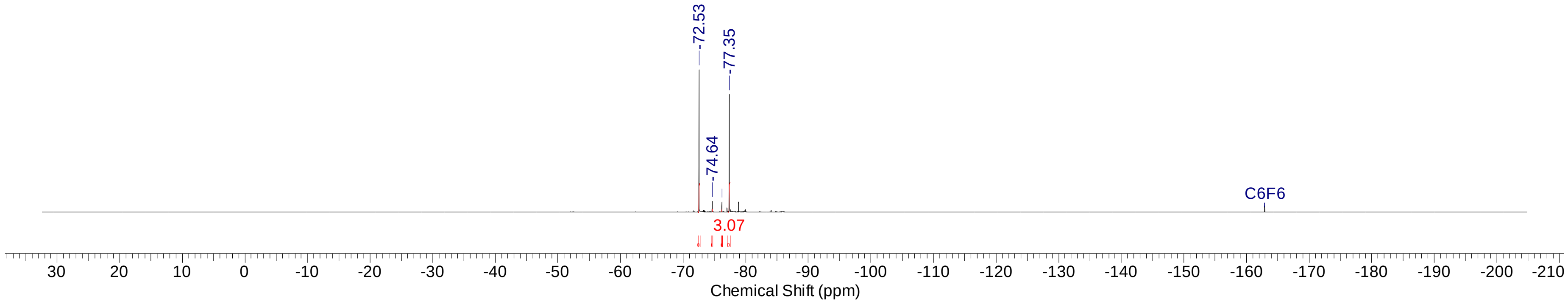

^{19}F NMR spectrum of **3b** (376.3 MHz, $CDCl_3$)

6 May 2019

Acquisition Time (sec)	4.0894	***Comment***	Imported from UXNMR.			***Date***	30 Oct 2018 16:01:54
File Name	C:\DOCS\OUTPUT_301\2018\10.î èöÿáðü\BM-1417.H_001001r					***Frequency (MHz)***	400.13
Nucleus	1H	***Number of Transients***	6	***Original Points Count***	32768	***Points Count***	131072
Pulse Sequence	zg30	***Solvent***	CHLOROFORM-D			***Sweep Width (Hz)***	8012.82
Temperature (degree C)	27.000						

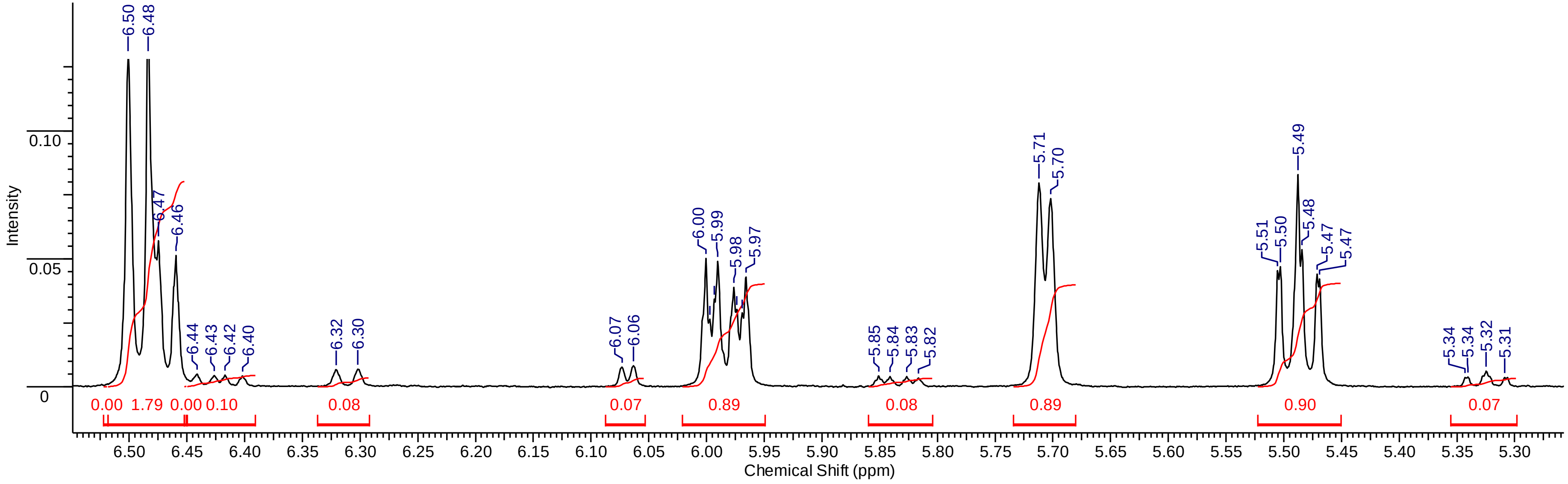

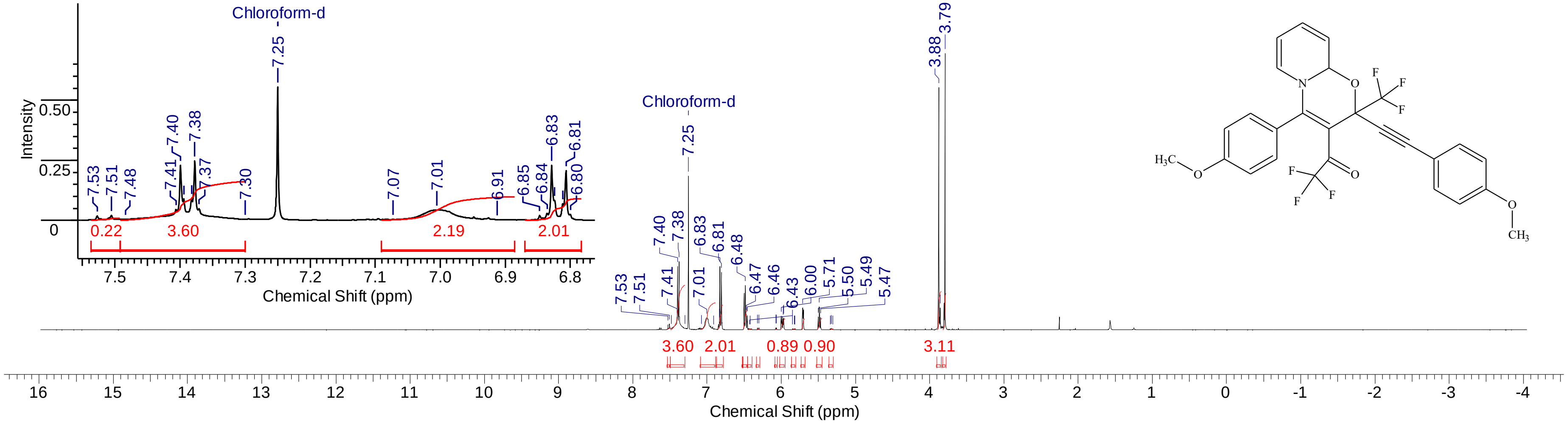

^{1}H NMR spectrum of **3c** (400.1 MHz, $CDCl_3$)

Acquisition Time (sec)	0.4999	***Comment***	Imported from UXNMR.			***Date***	08 Nov 2018 12:28:40
File Name	C:\DOCS\BM\SPEC_H,C_I-XII.2018\BM-1417.C_002001r			***Frequency (MHz)***	100.61	***Nucleus***	13C
Number of Transients	1086	***Original Points Count***	12076	***Points Count***	65536	***Pulse Sequence***	zgpg30
Solvent	CHLOROFORM-D			***Sweep Width (Hz)***	24154.59	***Temperature (degree C)***	27.000

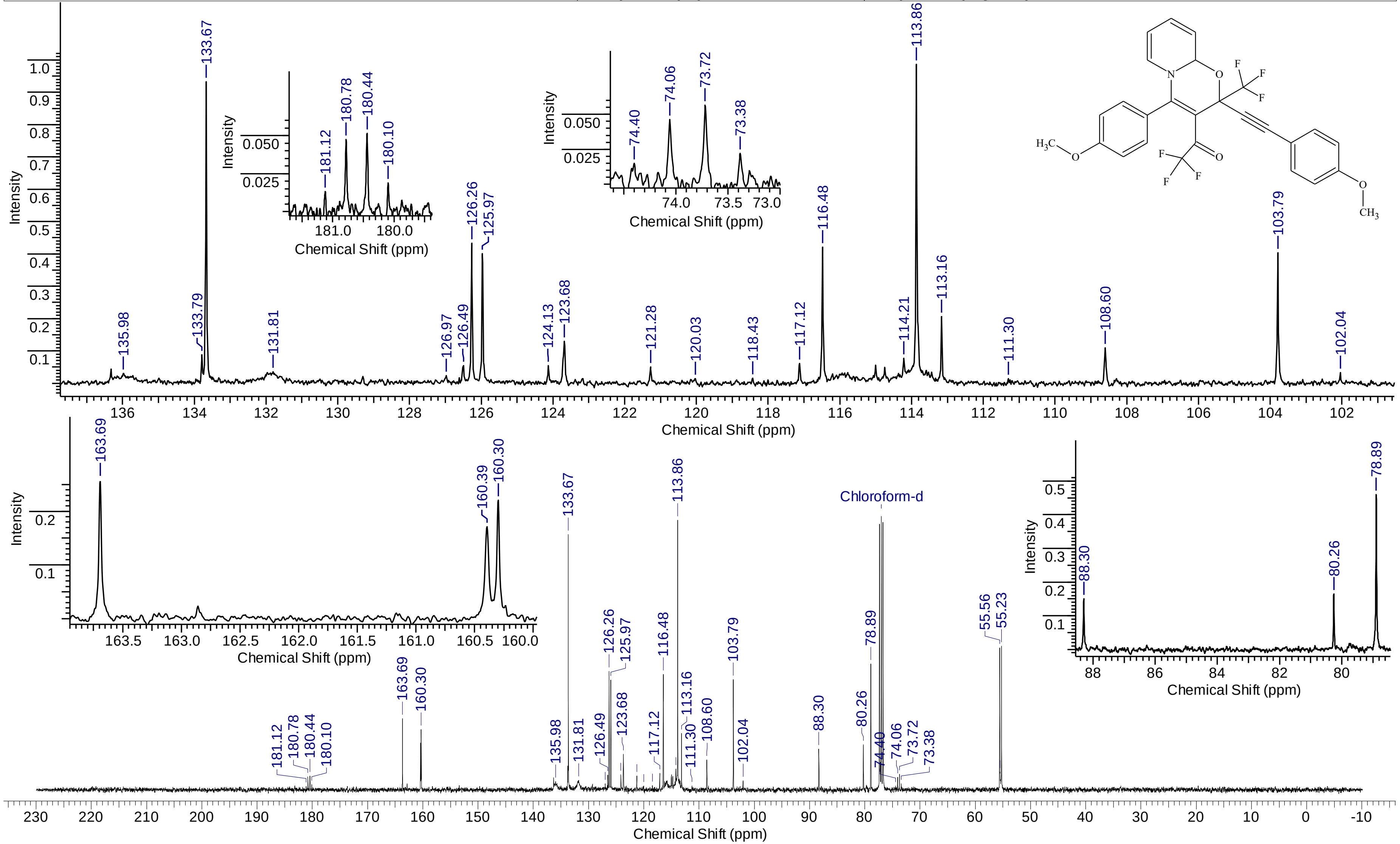

^{13}C NMR spectrum of **3c** (100.6 MHz, $CDCl_3$)

6 May 2019

Acquisition Time (sec)	0.7340	***Date***	Oct 30 2018	***File Name***	C:\DOCS\BM\SPEC_BM_F_2018.12.25\bm1417-f_20181030_01\FLUORINE_01		
Frequency (MHz)	376.31	***Nucleus***	19F	***Number of Transients***	100	***Original Points Count***	65536
Points Count	65536	***Pulse Sequence***	s2pul	***Solvent***	DMSO-D6	***Sweep Width (Hz)***	89285.71
Temperature (degree C)	22.000						

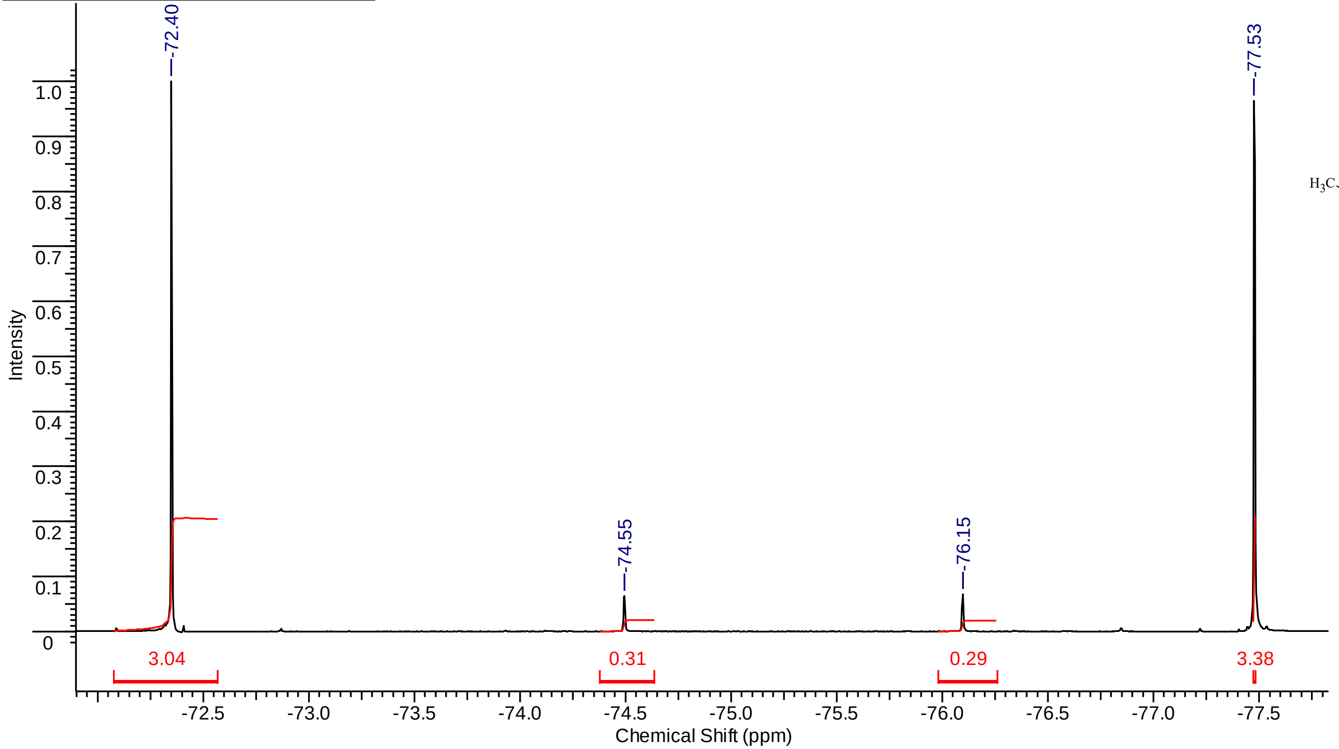

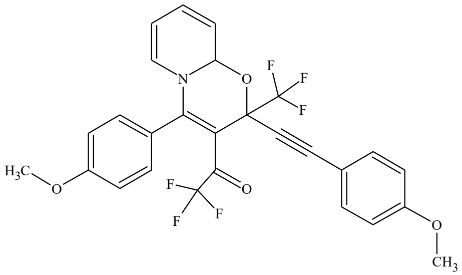

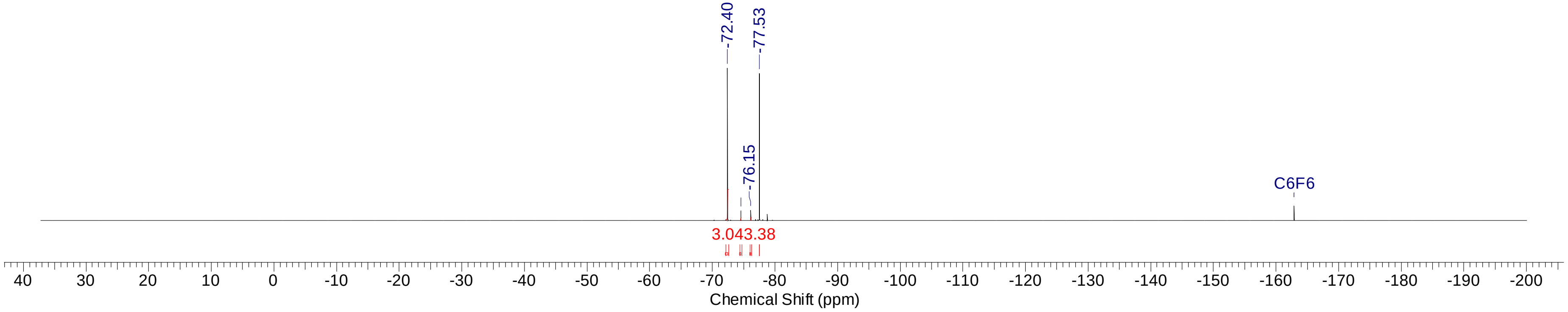

^{19}F NMR spectrum of **3c** (376.3 MHz, $CDCl_3$)

Acquisition Time (sec)	2.5559	***Comment***	Imported from UXNMR.			***Date***	13 Nov 2018 15:26:36
File Name	C:\DOCS\BM\SPEC_H,C_I-XII.2018\BM-1425-C.H_001001r			***Frequency (MHz)***	400.13	***Nucleus***	1H
Number of Transients	6	***Original Points Count***	16384	***Points Count***	65536	***Pulse Sequence***	zg30
Solvent	CHLOROFORM-D			***Sweep Width (Hz)***	6410.26	***Temperature (degree C)***	27.000

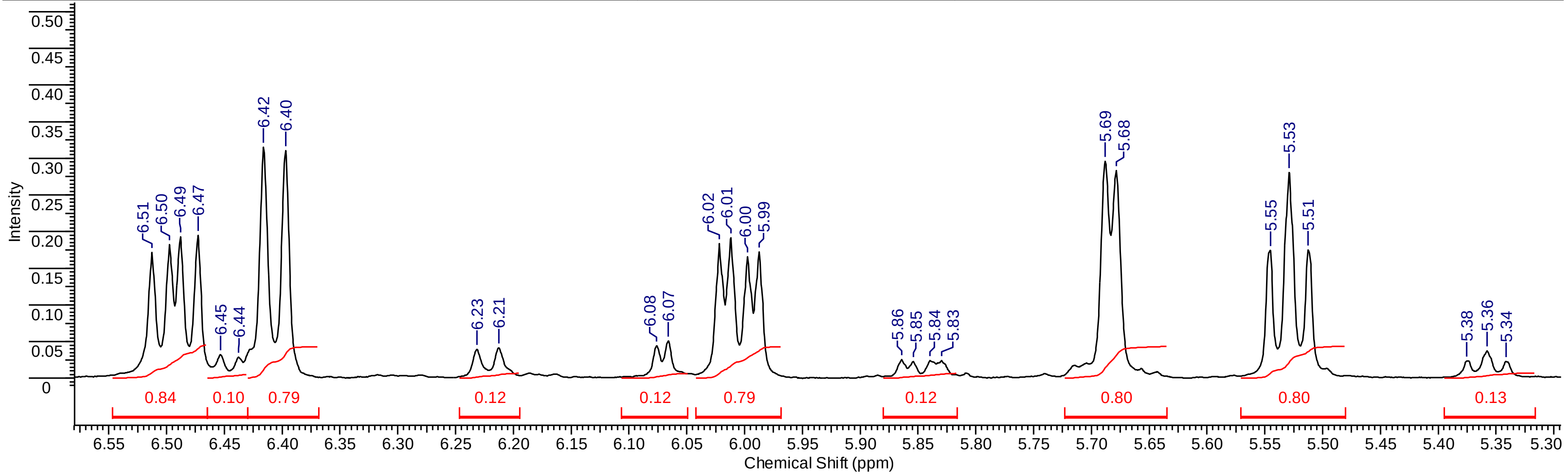

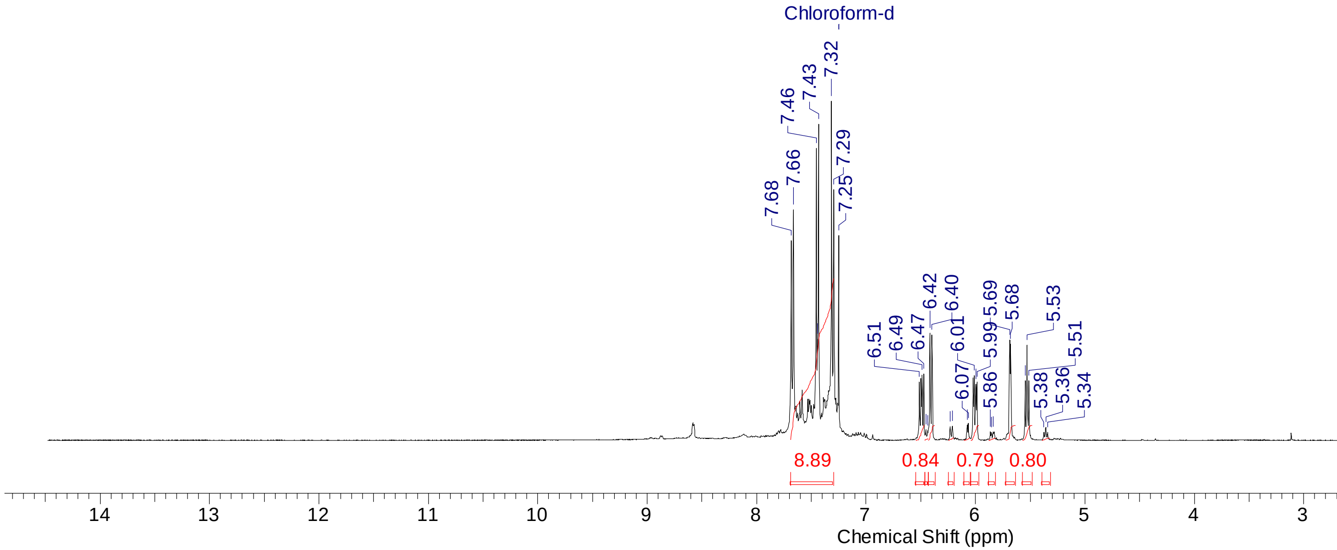

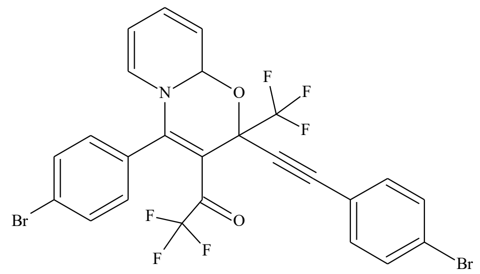

^{1}H NMR spectrum of **3d** (400.1 MHz, $CDCl_3$)

10 May 2019

Acquisition Time (sec)	0.4999	***Comment***	Imported from UXNMR.			***Date***	13 Nov 2018 15:58:00
File Name	C:\DOCS\BM\SPEC_H,C_I-XII.2018\BM-1425-C.C_002001r			***Frequency (MHz)***	100.61	***Nucleus***	13C
Number of Transients	1156	***Original Points Count***	12076	***Points Count***	65536	***Pulse Sequence***	zgpg30
Solvent	DMSO-D6	***Sweep Width (Hz)***	24154.59	***Temperature (degree C)***	27.000		

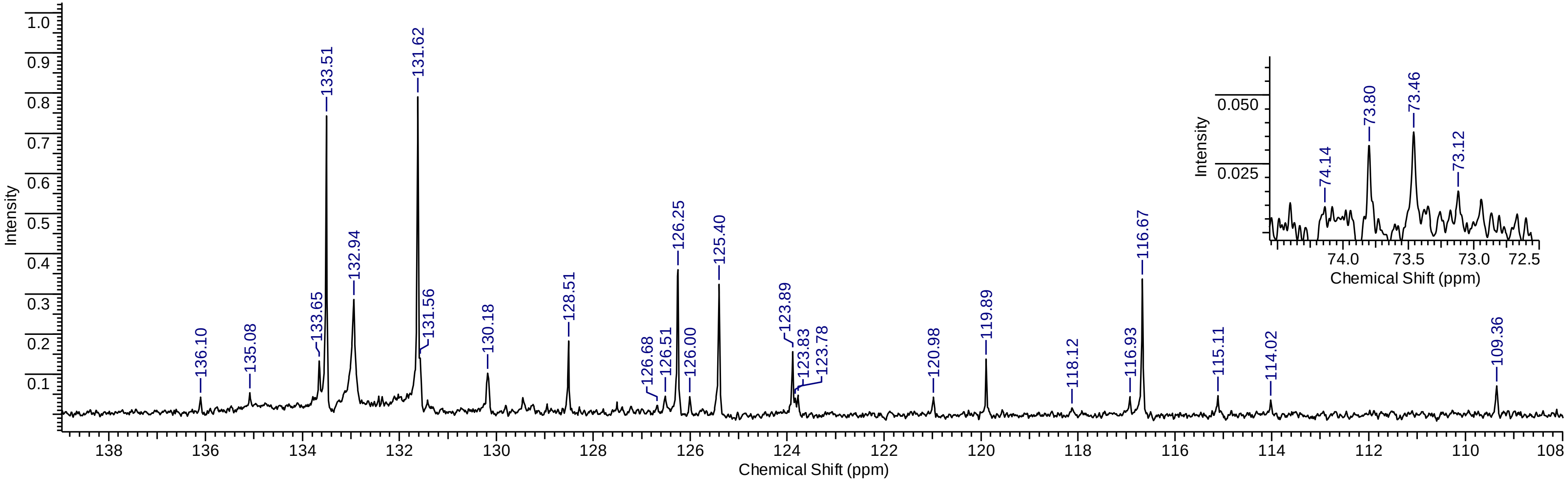

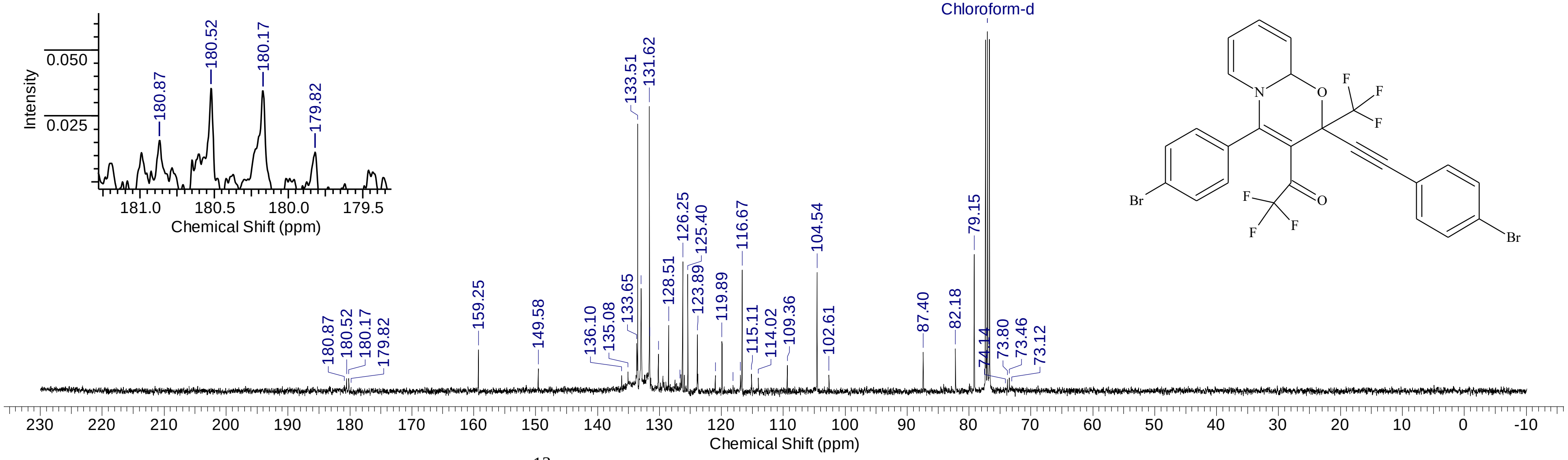

^{13}C NMR spectrum of **3d** (100.6 MHz, $CDCl_3$)

9 May 2019

Acquisition Time (sec)	1.0000	Date	Nov 2 2018	File Name	C:\DOCS\BM\SPEC_BM_F_2018.12.25\BM-1425_20181102_01\FLUORINE_01		
Frequency (MHz)	376.31	Nucleus	19F	Number of Transients	16	Original Points Count	89286
Points Count	131072	Pulse Sequence	s2pul	Solvent	CHLOROFORM-D		
Sweep Width (Hz)	89285.71	Temperature (degree C)	22.000				

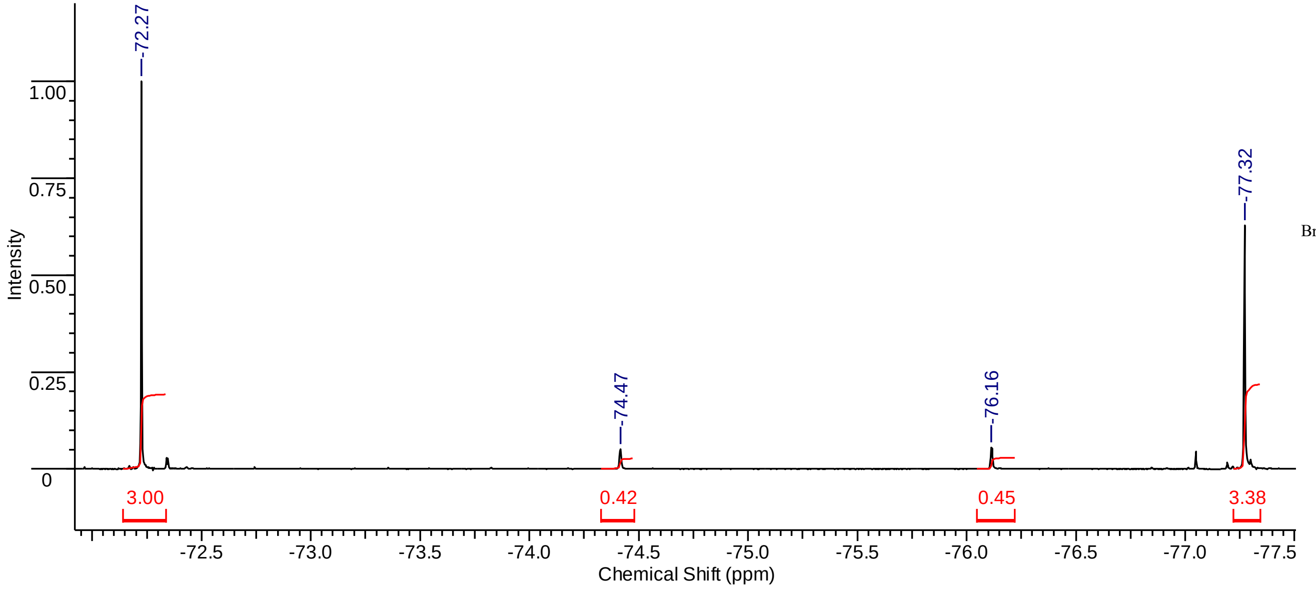

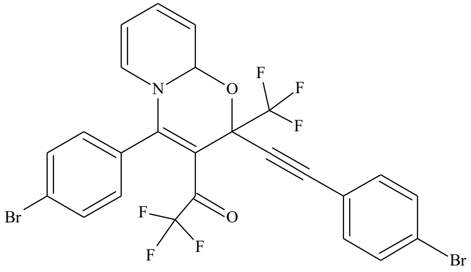

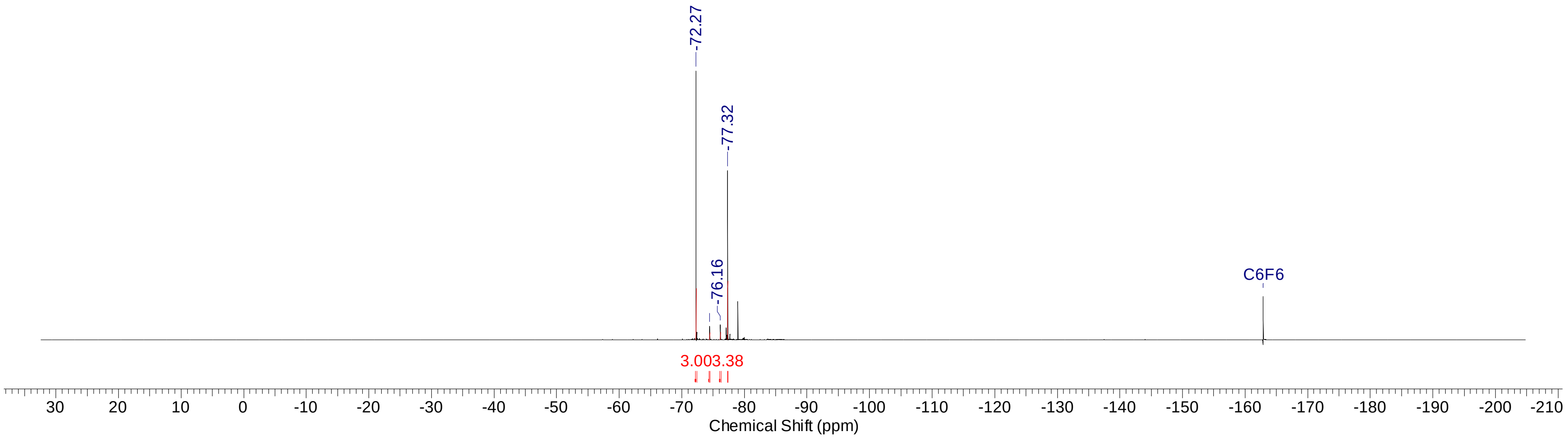

^{19}F NMR spectrum of **3d** (376.3 MHz, $CDCl_3$)

13 Jun 2019

Acquisition Time (sec)	4.0894	***Comment***	Imported from UXNMR.			***Date***	07 Nov 2018 17:10:10
File Name	C:\DOCS\BM\SPEC_H,C_I-XII.2018\BM-1426.H_001001r			***Frequency (MHz)***	400.13	***Nucleus***	1H
Number of Transients	5	***Original Points Count***	32768	***Points Count***	131072	***Pulse Sequence***	zg30
Solvent	ACETONITRILE-D3			***Sweep Width (Hz)***	8012.82	***Temperature (degree C)***	27.000

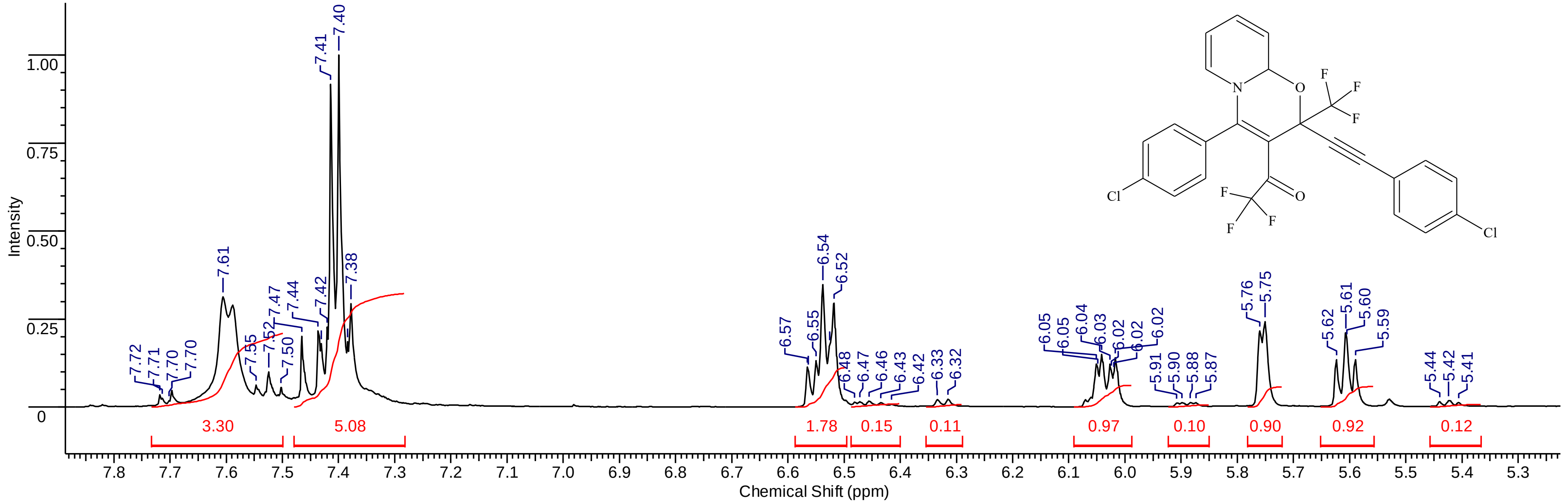

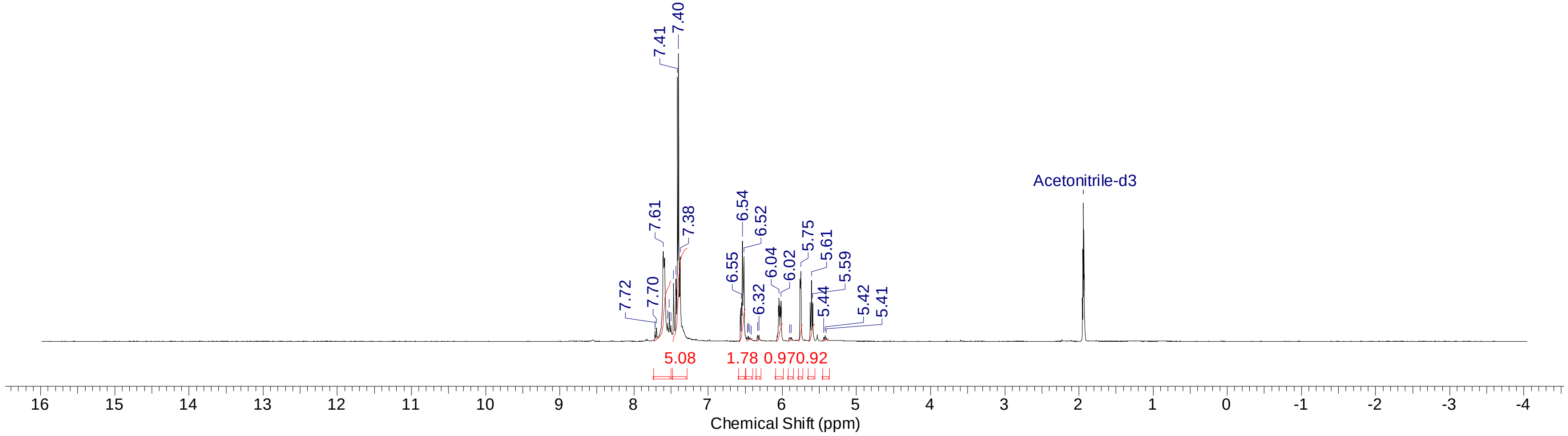

^{1}H NMR spectrum of **3e** (400.1 MHz, CD_3CN)

13 Jun 2019

Acquisition Time (sec)	4.0894	Comment	Imported from UXNMR.			Date	06 Oct 2018 12:51:42
File Name	F:\COMP_PRAK\DOCS\OUTPUT_301\2018\10.î êòÿáðü\181006\BM-1390-2_001001r					Frequency (MHz)	400.13
Nucleus	1H	Number of Transients	8	Original Points Count	32768	Points Count	131072
Pulse Sequence	zg30	Solvent	CHLOROFORM-D			Sweep Width (Hz)	8012.82
Temperature (degree C)	27.000						

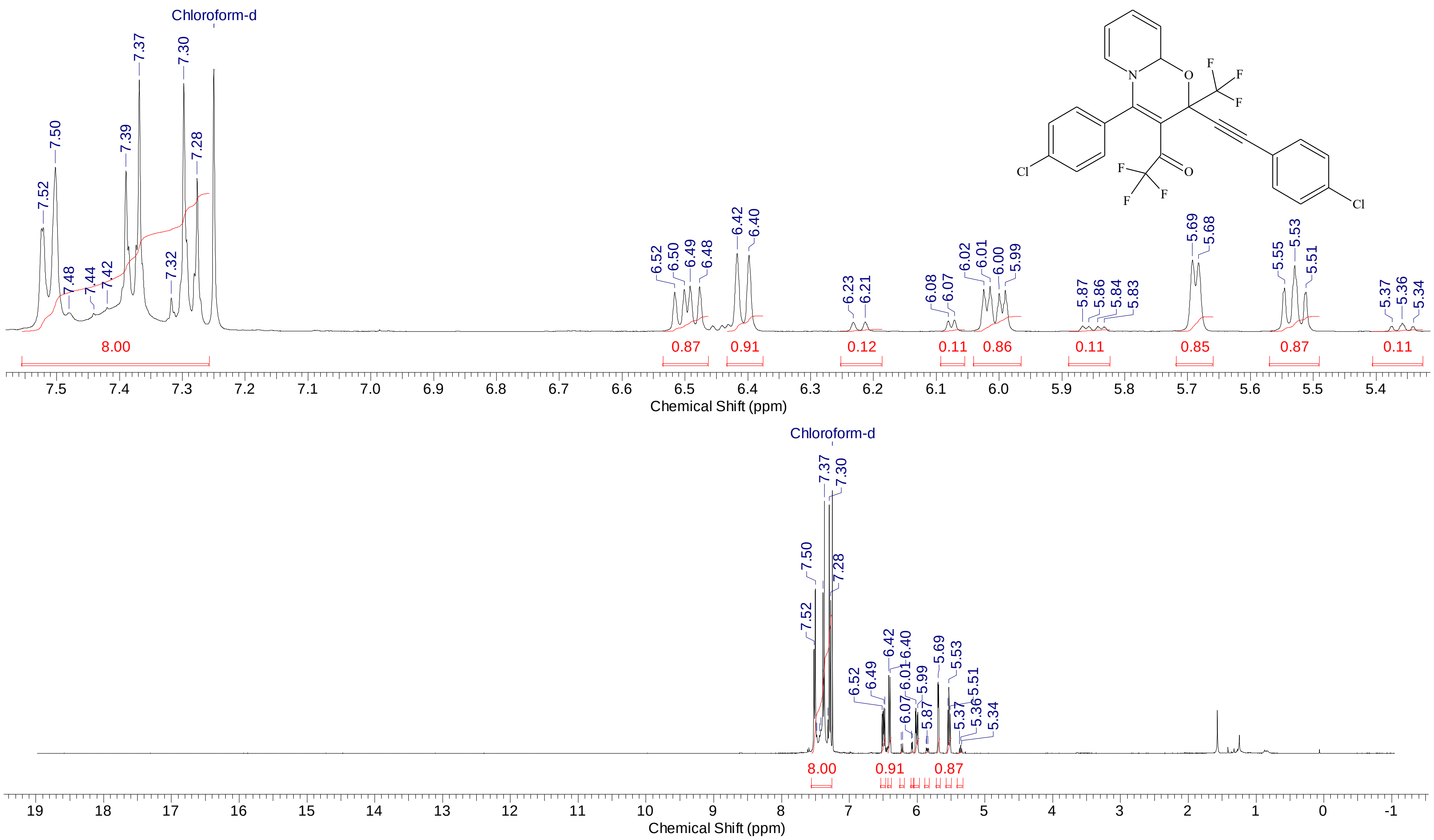

^{1}H NMR spectrum of **3e** (400.1 MHz, $CDCl_3$)

Acquisition Time (sec)	0.4999	***Comment***	Imported from UXNMR.	***Date***	07 Nov 2018 17:38:58
File Name	C:\DOCS\BM\SPEC_H,C_I-XII.2018\BM-1426.C_002001r	***Frequency (MHz)***	100.61	***Nucleus***	13C
Number of Transients	1024	***Original Points Count*** 12076 / ***Points Count***	65536	***Pulse Sequence***	zgpg30
Solvent	CHLOROFORM-D	***Sweep Width (Hz)***	24154.59	***Temperature (degree C)***	27.000

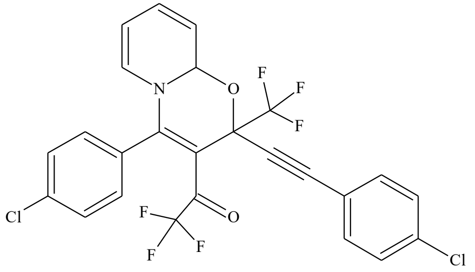

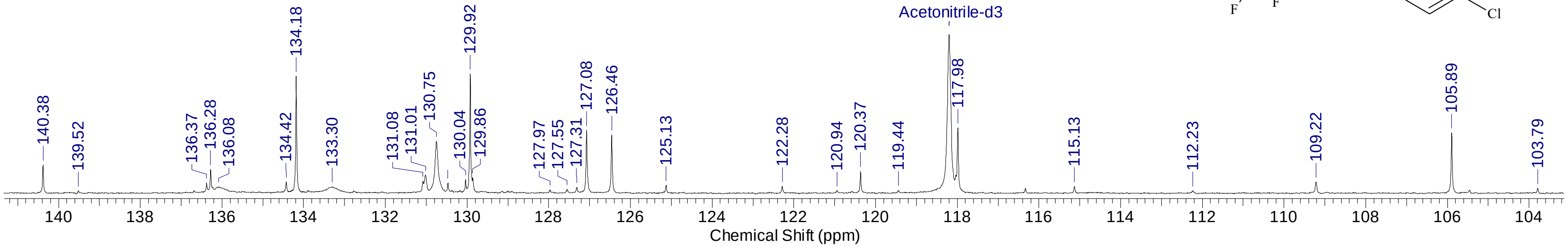

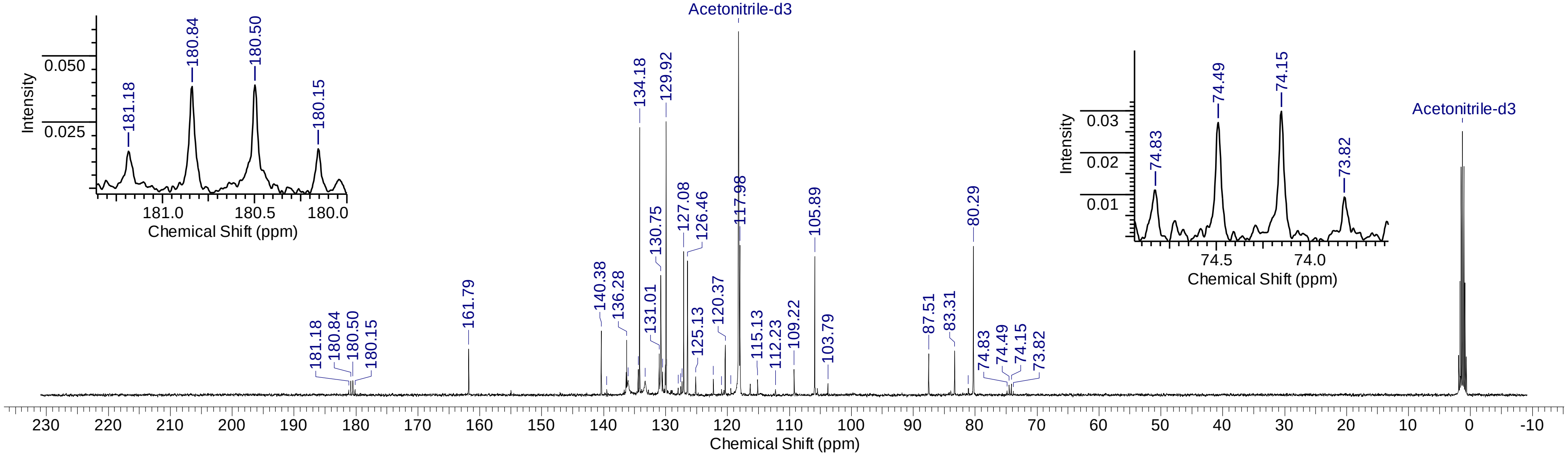

^{13}C NMR spectrum of **3e** (100.6 MHz, CD_3CN)

13 Jun 2019

Acquisition Time (sec)	0.4999	***Comment***	Imported from UXNMR.			***Date***	05 Oct 2018 15:54:24
File Name	F:\COMP_PRAK\DOCS\OUTPUT_301\2018\10.î êòÿáðü\BM-1390.C_002001r					***Frequency (MHz)***	100.61
Nucleus	13C	***Number of Transients***	260	***Original Points Count***	12076	***Points Count***	65536
Pulse Sequence	zgpg30	***Solvent***	DMSO-D6	***Sweep Width (Hz)***	24154.59	***Temperature (degree C)***	27.000

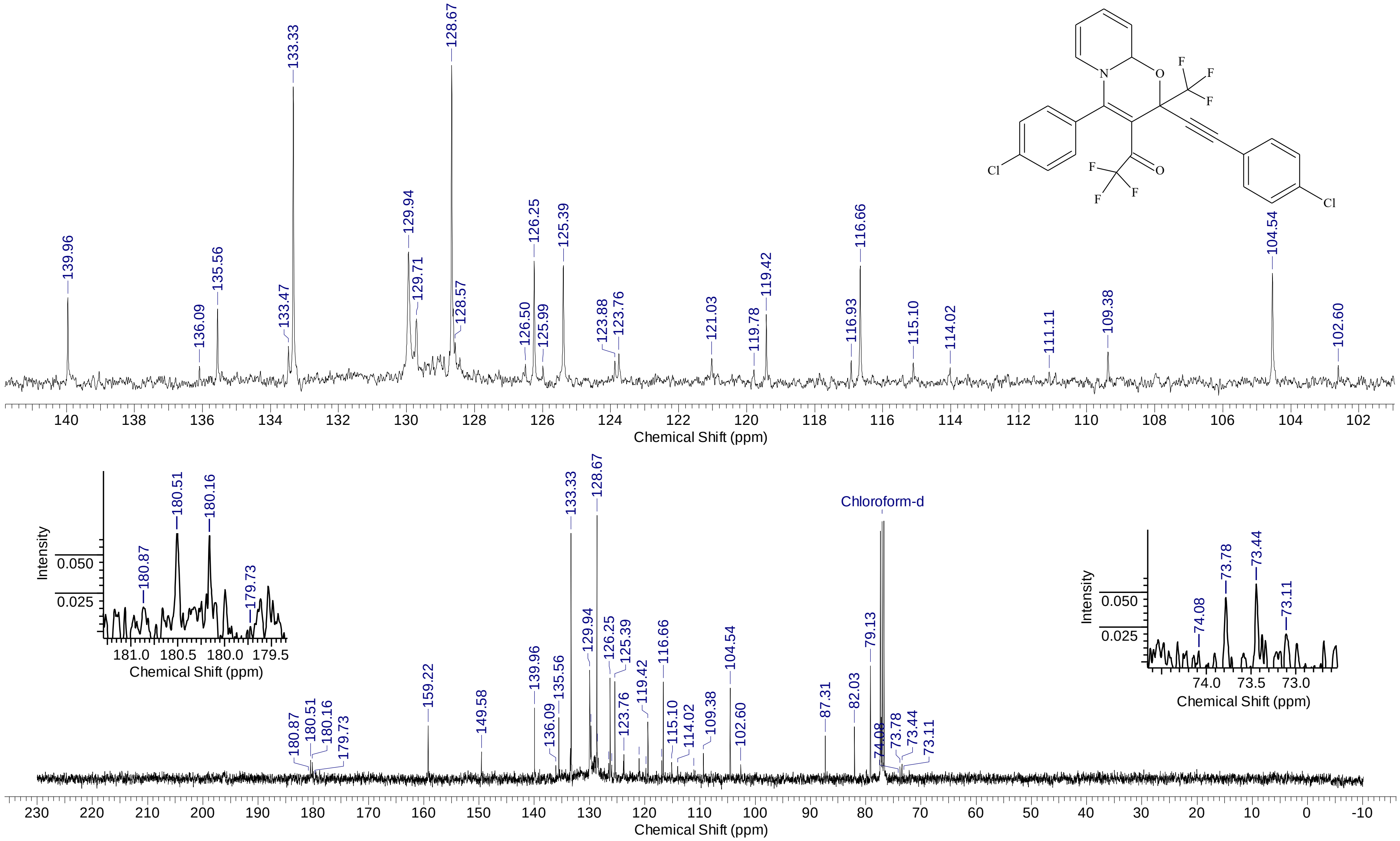

^{13}C NMR spectrum of **3e** (100.6 MHz, $CDCl_3$)

13 Jun 2019

Acquisition Time (sec)	1.0000	***Date***	Oct 5 2018	***File Name***	F:\COMP_PRAK\DOCS\OUTPUT_301\F19\2018.10.05\BM-1390_20181005_01\FLUORINE_01		
Frequency (MHz)	376.31	***Nucleus***	19F	***Number of Transients***	16	***Original Points Count***	89286
Points Count	131072	***Pulse Sequence***	s2pul	***Solvent***	CHLOROFORM-D		
Sweep Width (Hz)	89285.71	***Temperature (degree C)***	22.000				

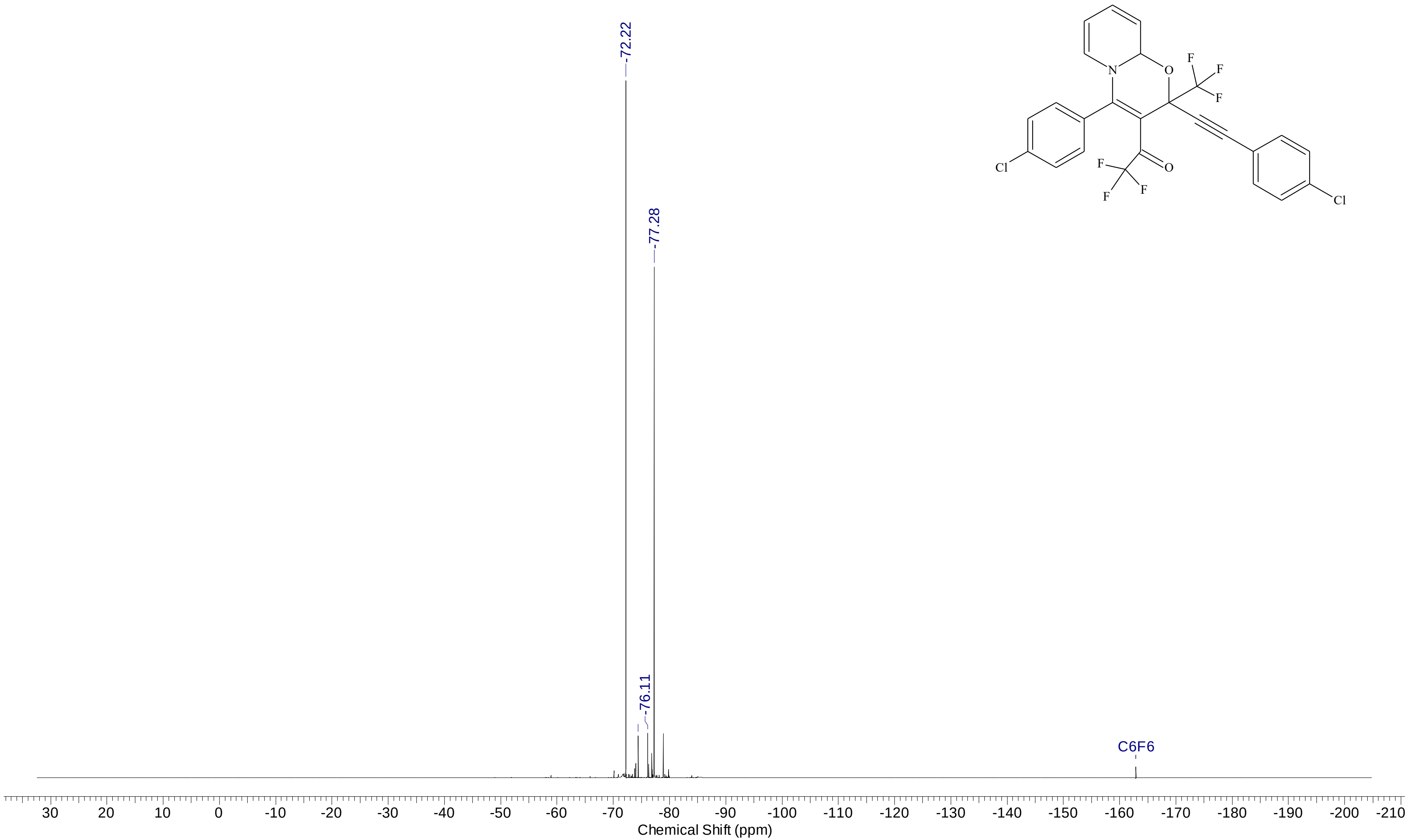

^{19}F NMR spectrum of **3e** (376.3 MHz, $CDCl_3$)

16 Sep 2019

Acquisition Time (sec)	2.3069	***Date***	Nov 8 2018	***File Name***	C:\DOCS\BM\SPEC_BM_F_2018.12.25\BM-1426-d-F_20181108_01\FLUORINE_01		
Frequency (MHz)	376.32	***Nucleus***	19F	***Number of Transients***	8	***Original Points Count***	262144
Points Count	262144	***Pulse Sequence***	s2pul	***Solvent***	ACETONITRILE-D3		
Sweep Width (Hz)	113636.37	***Temperature (degree C)***	22.000				

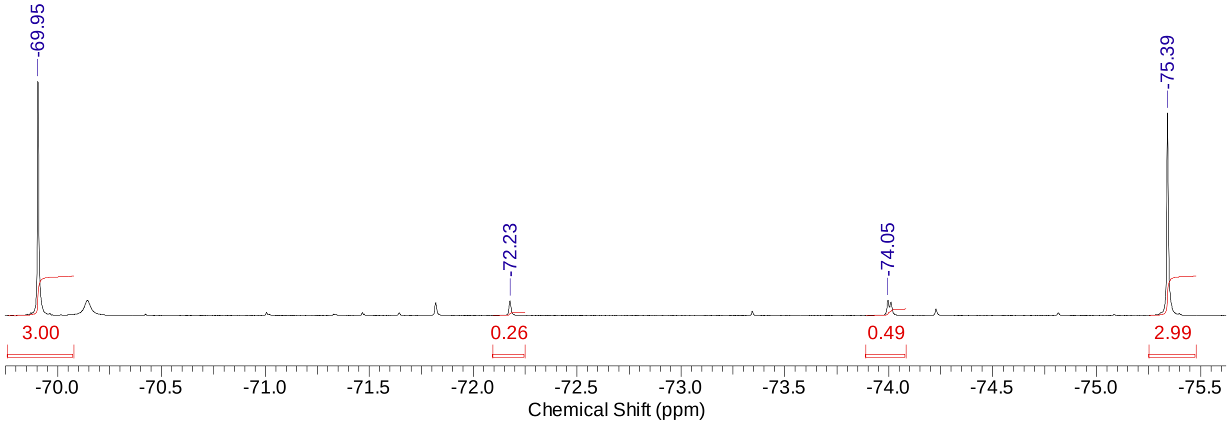

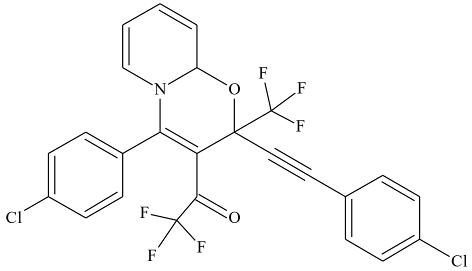

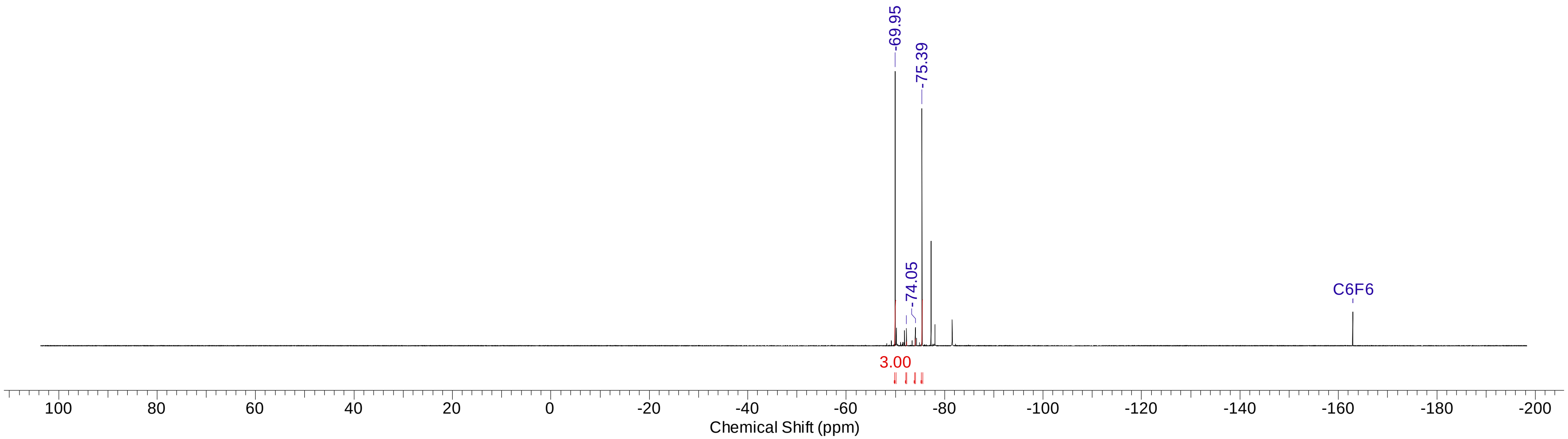

^{19}F NMR spectrum of **3e** (376.3 MHz, CD_3CN)

14 Jun 2019

Acquisition Time (sec)	2.5559	***Comment***	Imported from UXNMR.	***Date***	03 Oct 2018 17:53:56
File Name	F:\COMP_PRAK\DOCS\OUTPUT_301\2018\10.î êòÿáðü\BM-1387.H_001001r			***Frequency (MHz)***	400.13
Nucleus	1H	***Number of Transients***	8 ***Original Points Count*** 16384	***Points Count***	65536
Pulse Sequence	zg30	***Solvent***	CHLOROFORM-D	***Sweep Width (Hz)***	6410.26
Temperature (degree C)	27.000				

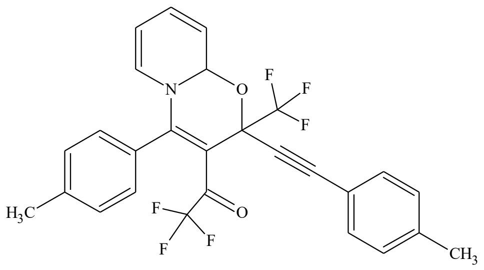

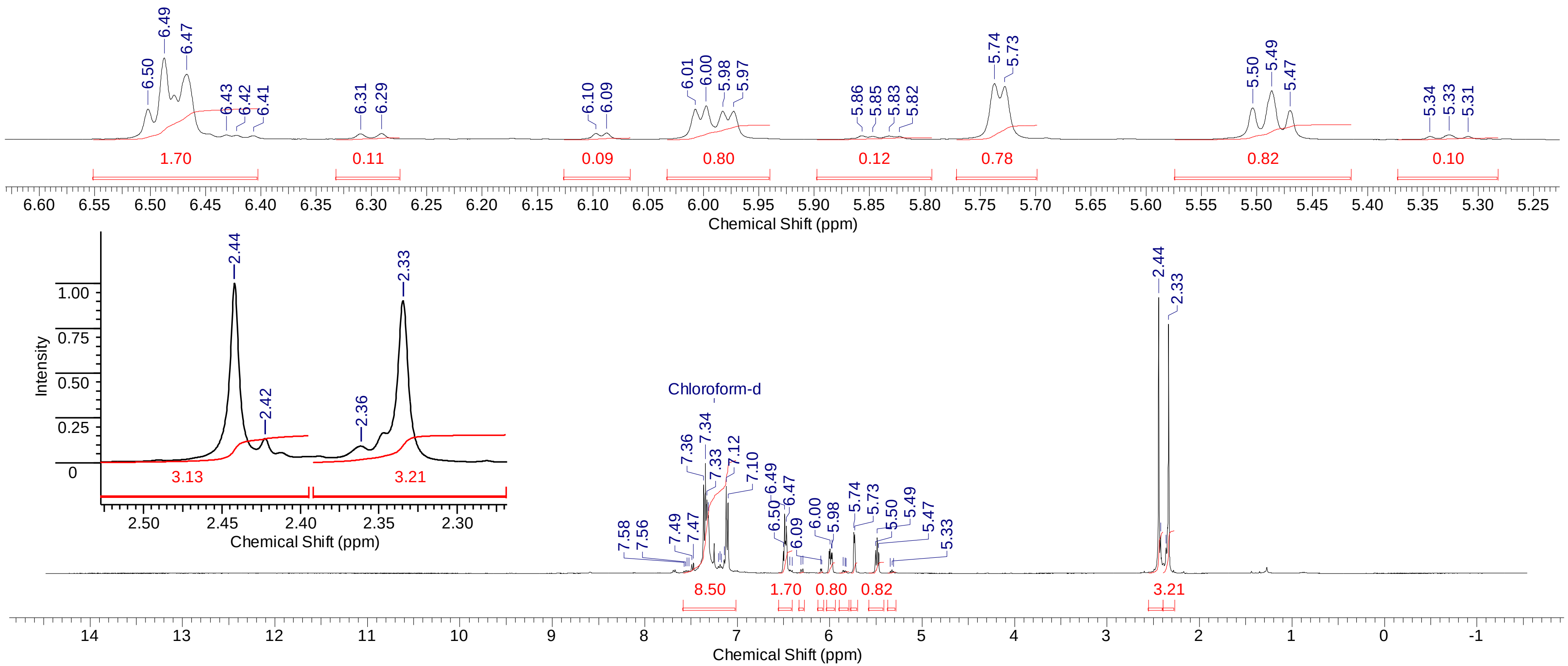

^{1}H NMR spectrum of **3f** (400.1 MHz, $CDCl_3$)

14 Jun 2019

Acquisition Time (sec)	0.4999	Comment	Imported from UXNMR.			Date	03 Oct 2018 18:09:32
File Name	F:\COMP_PRAK\DOCS\OUTPUT_301\2018\10.î êòÿáðü\BM-1387.C_002001r					Frequency (MHz)	100.61
Nucleus	13C	Number of Transients	512	Original Points Count	12076	Points Count	65536
Pulse Sequence	zgpg30	Solvent	DMSO-D6	Sweep Width (Hz)	24154.59	Temperature (degree C)	27.000

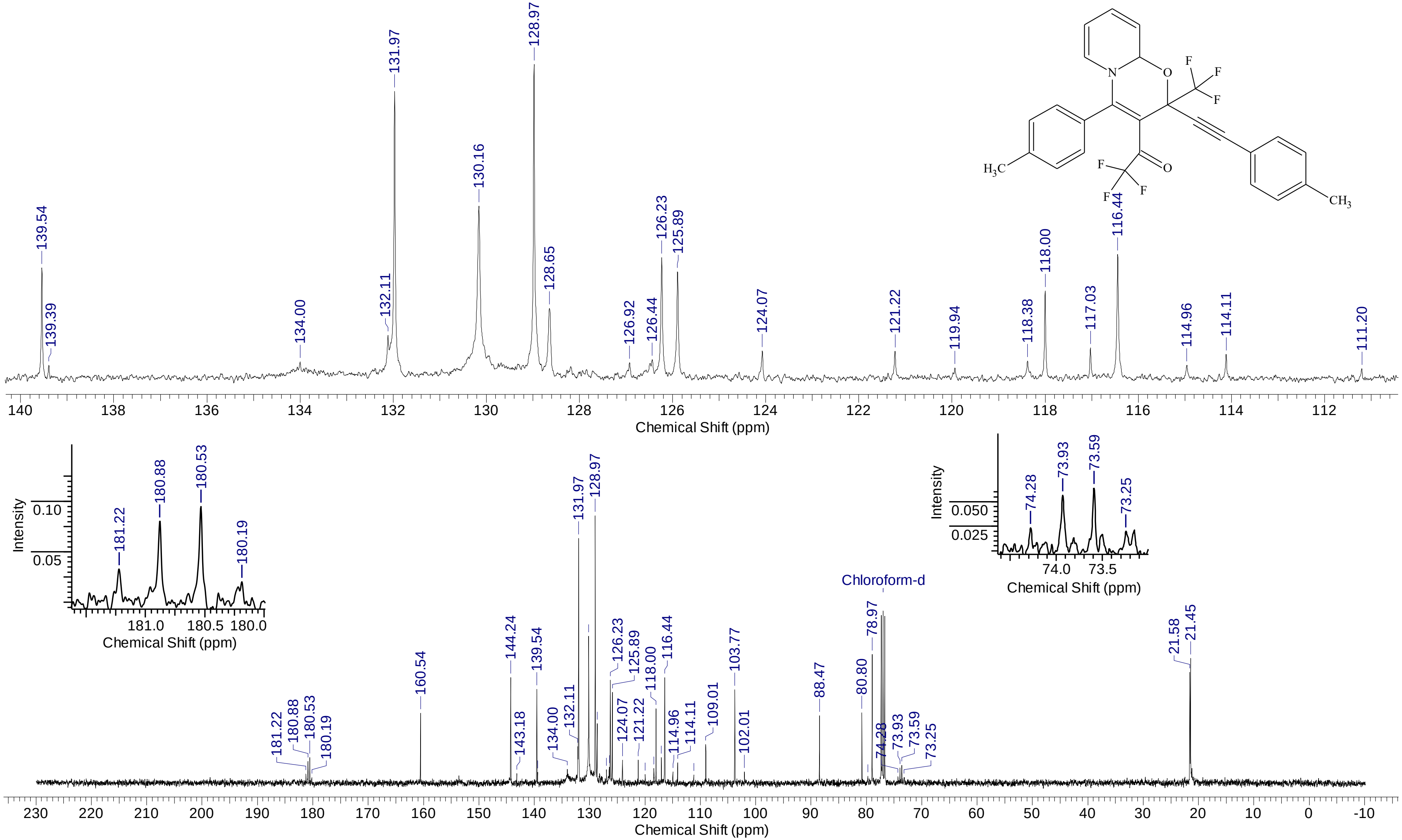

^{13}C NMR spectrum of **3f** (100.6 MHz, $CDCl_3$)

14 Jun 2019

Acquisition Time (sec)	2.3069	***Date***	Oct 4 2018				
File Name	F:\COMP_PRAK\DOCS\OUTPUT_301\F19\2018.10.04\BM-13878-F_20181004_01\FLUORINE_01					***Frequency (MHz)***	376.32
Nucleus	19F	***Number of Transients***	8	***Original Points Count***	262144	***Points Count***	262144
Pulse Sequence	s2pul	***Solvent***	CHLOROFORM-D			***Sweep Width (Hz)***	113636.37
Temperature (degree C)	22.000						

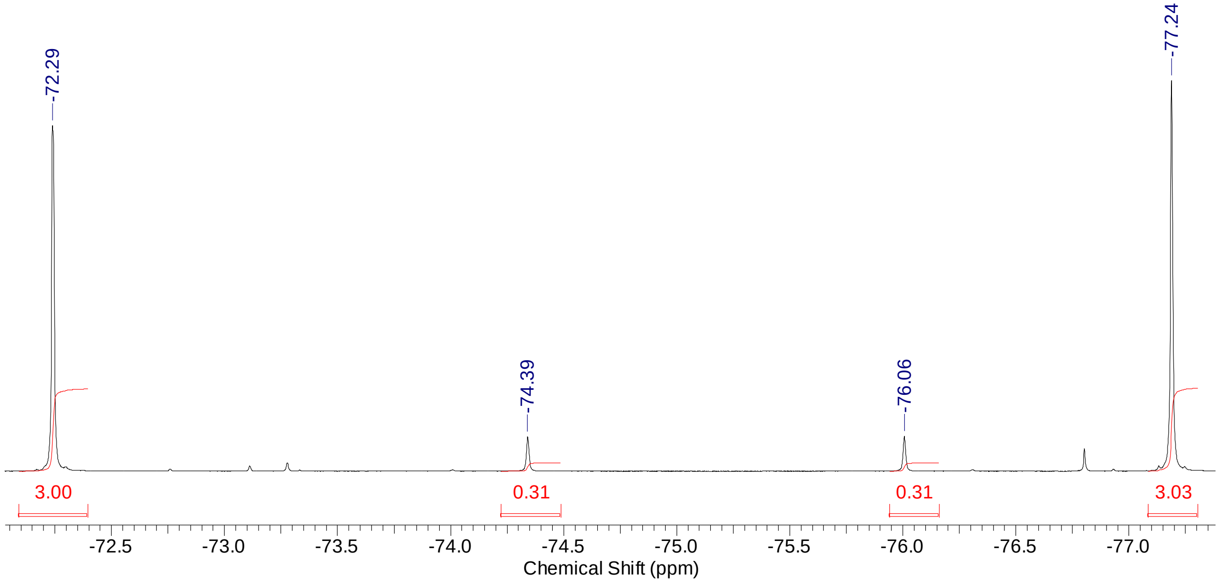

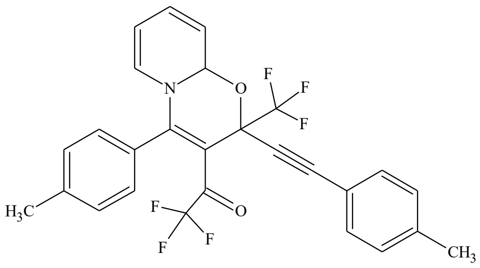

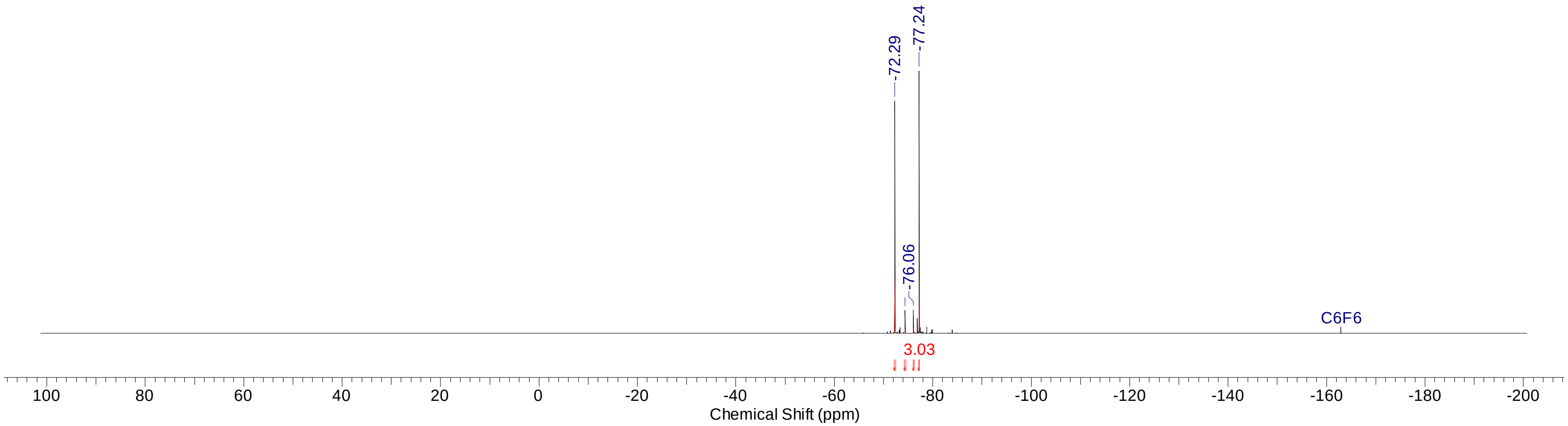

^{19}F NMR spectrum of **3f** (376.3 MHz, $CDCl_3$)

14 Jun 2019

Acquisition Time (sec)	2.5559	***Comment***	Imported from UXNMR.			***Date***	10 Nov 2018 13:34:36
File Name	C:\DOCS\BM\SPEC_H,C_I-XII.2018\BM-1429.H_001001r			***Frequency (MHz)***	400.13	***Nucleus***	1H
Number of Transients	8	***Original Points Count***	16384	***Points Count***	65536	***Pulse Sequence***	zg30
Solvent	ACETONITRILE-D3			***Sweep Width (Hz)***	6410.26	***Temperature (degree C)***	27.000

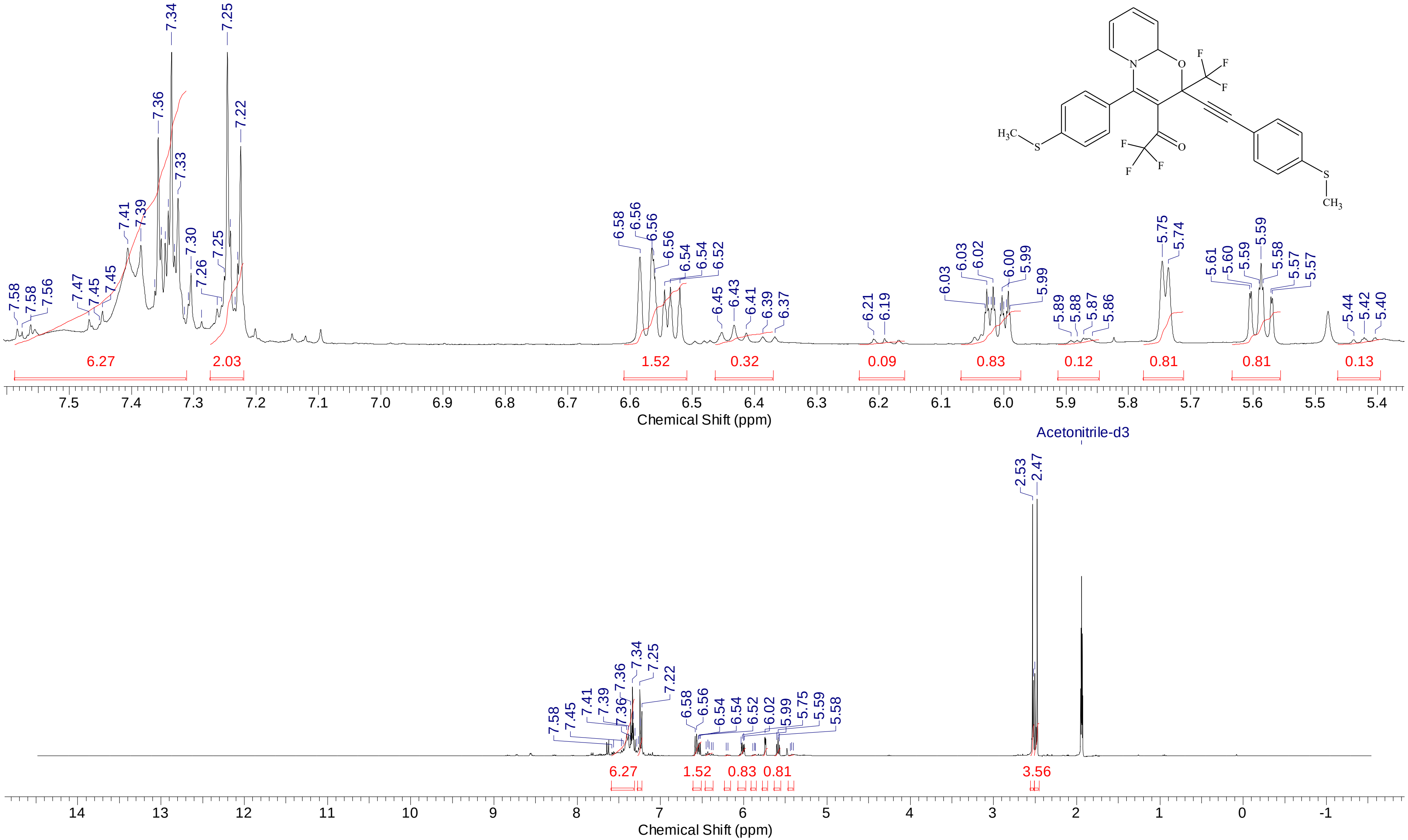

^{1}H NMR spectrum of **3g** (400.1 MHz, CD_3CN)

14 Jun 2019

Acquisition Time (sec)	4.0894	***Comment***	Imported from UXNMR.			***Date***	30 Oct 2018 21:06:58
File Name	C:\DOCS\BM\SPEC_H,C_I-XII.2018\BM-1421\BM-1421_001001r					***Frequency (MHz)***	400.13
Nucleus	1H	***Number of Transients***	8	***Original Points Count***	32768	***Points Count***	131072
Pulse Sequence	zg30	***Solvent***	CHLOROFORM-D			***Sweep Width (Hz)***	8012.82
Temperature (degree C)	27.000						

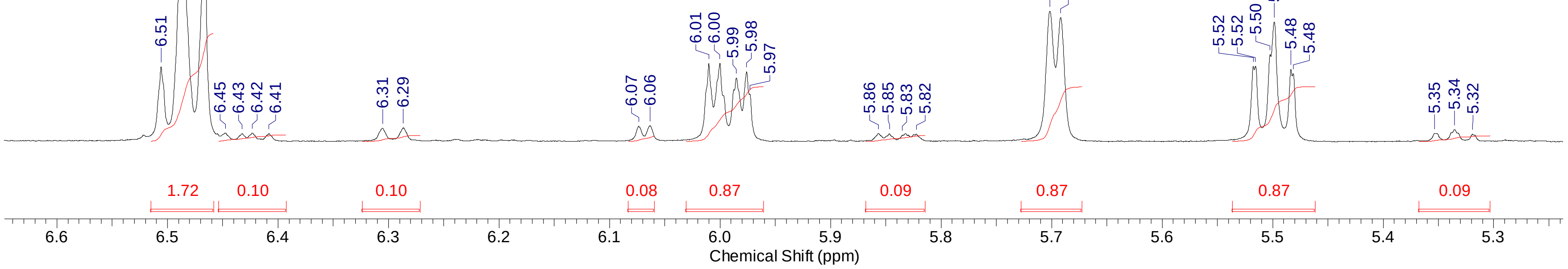

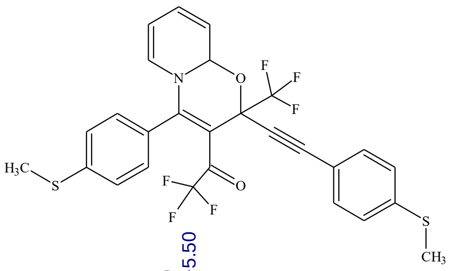

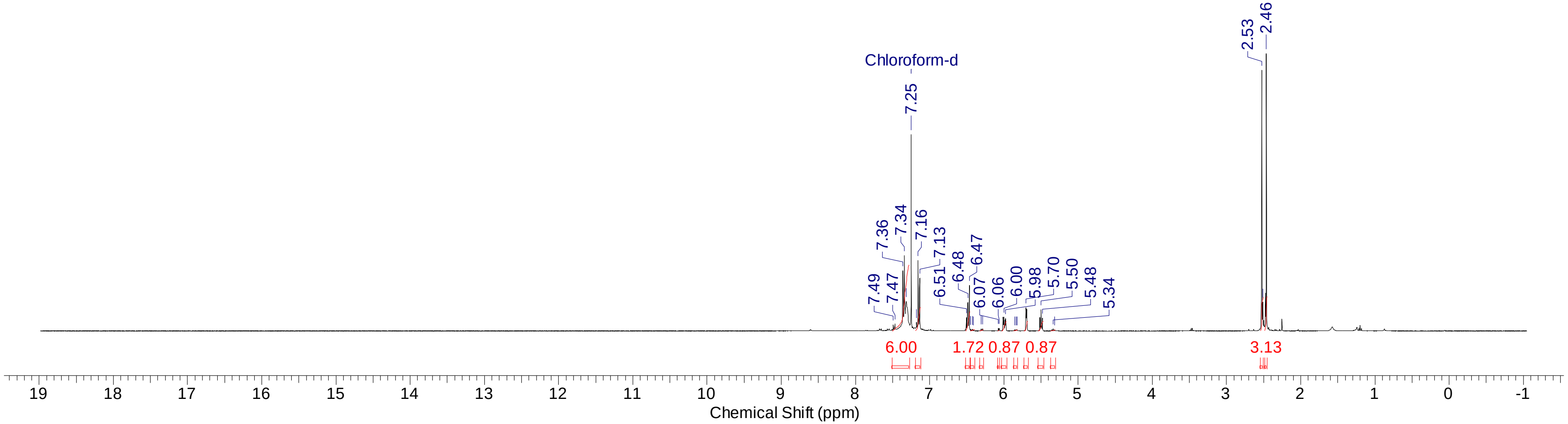

^{1}H NMR spectrum of **3g** (400.1 MHz, $CDCl_3$)

14 Jun 2019

Acquisition Time (sec)	0.6783	***Comment***	Imported from UXNMR.			***Date***	12 Nov 2018 15:20:40
File Name	C:\DOCS\BM\SPEC_H,C_I-XII.2018\BM-1429.C_002001r			***Frequency (MHz)***	100.61	***Nucleus***	13C
Number of Transients	1025	***Original Points Count***	16384	***Points Count***	131072	***Pulse Sequence***	zgpg30
Solvent	ACETONITRILE-D3			***Sweep Width (Hz)***	24154.59	***Temperature (degree C)***	27.000

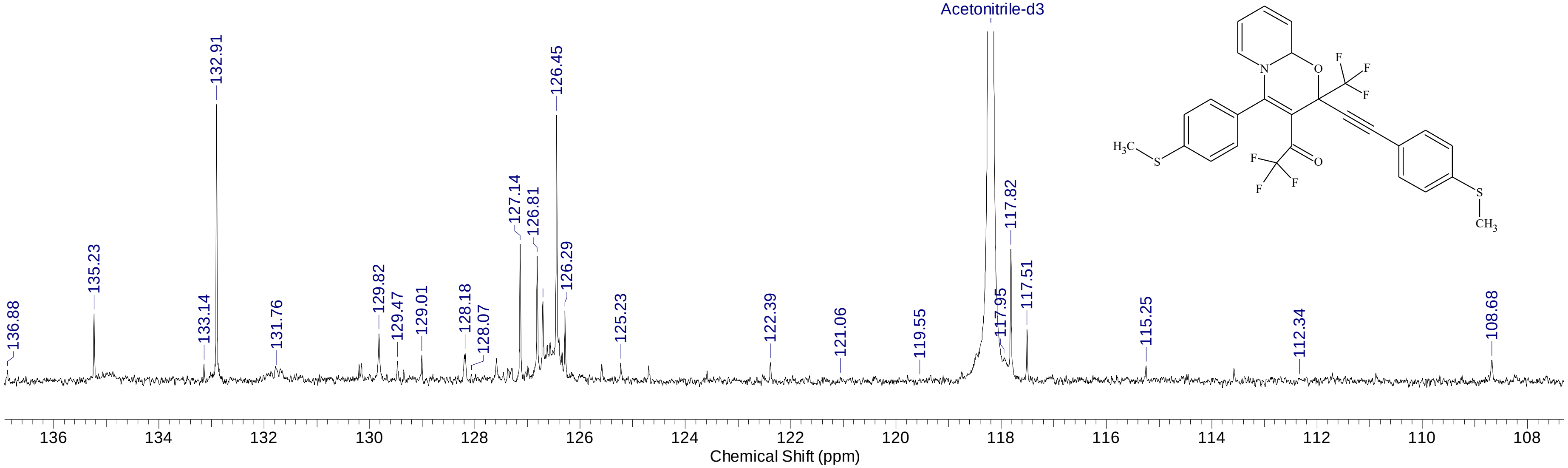

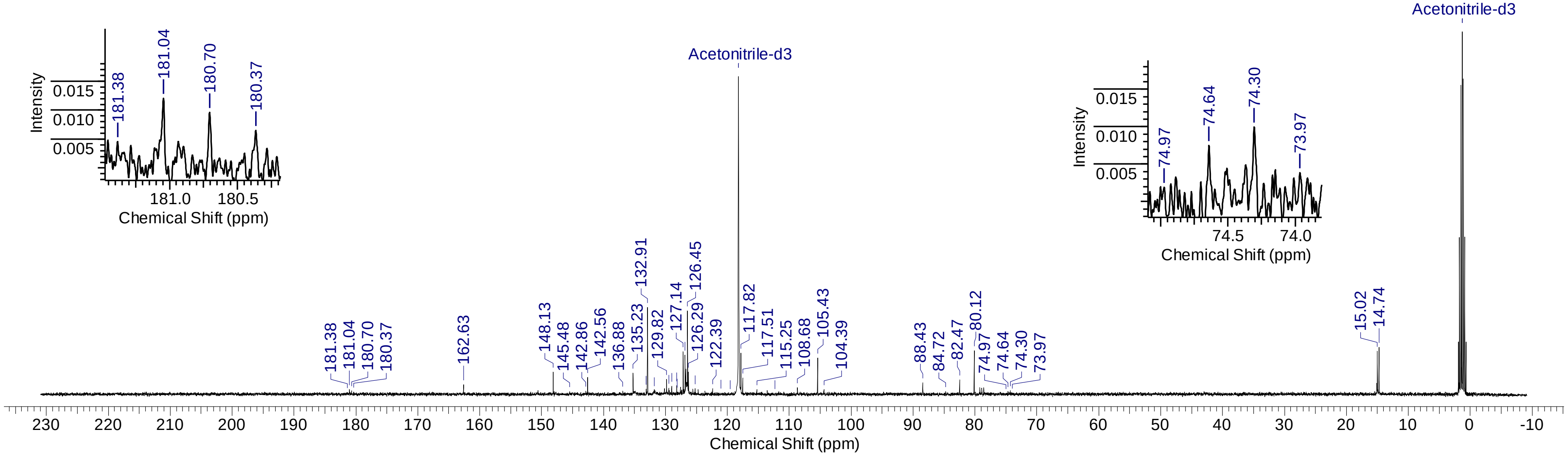

^{13}C NMR spectrum of **3g** (100.6 MHz, CD_3CN)

14 Jun 2019

Acquisition Time (sec)	1.0000	***Date***	Nov 9 2018	***File Name***	C:\DOCS\BM\SPEC_BM_F_2018.12.25\BM-1429_20181109_01\FLUORINE_01		
Frequency (MHz)	376.31	***Nucleus***	19F	***Number of Transients***	16	***Original Points Count***	89286
Points Count	131072	***Pulse Sequence***	s2pul	***Solvent***	ACETONITRILE-D3		
Sweep Width (Hz)	89285.71	***Temperature (degree C)***	22.000				

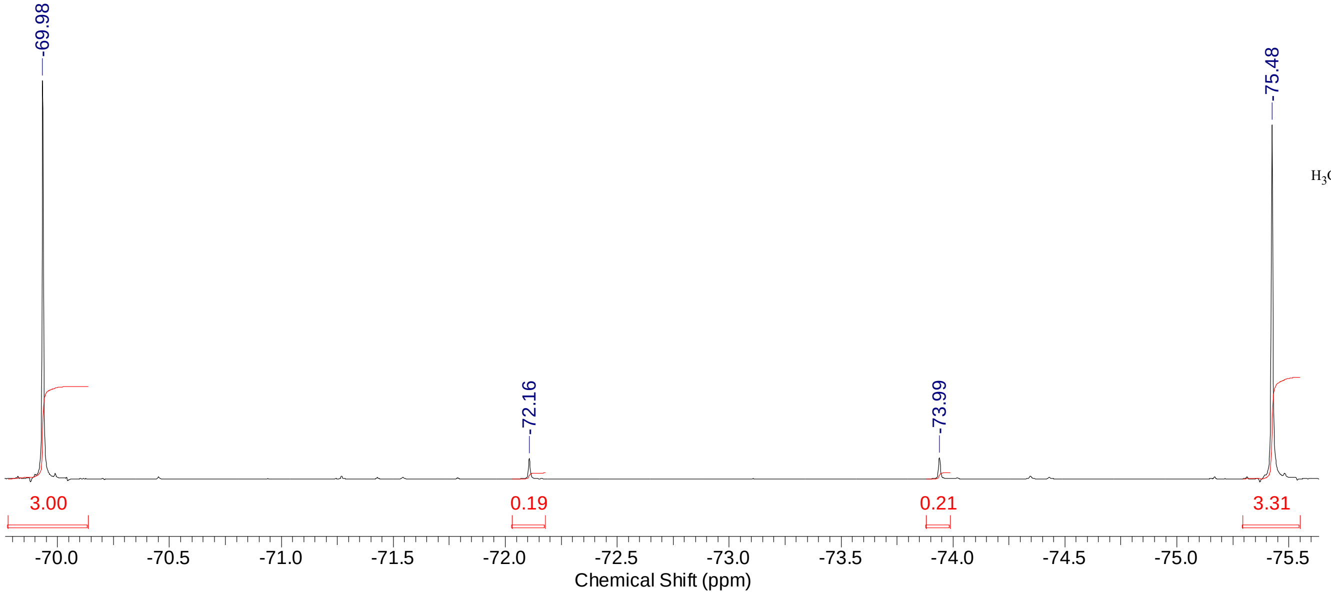

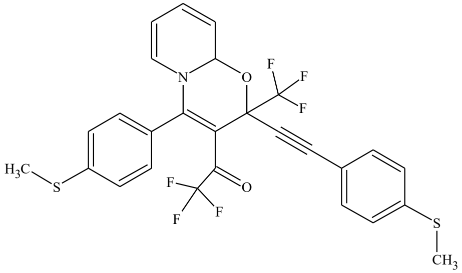

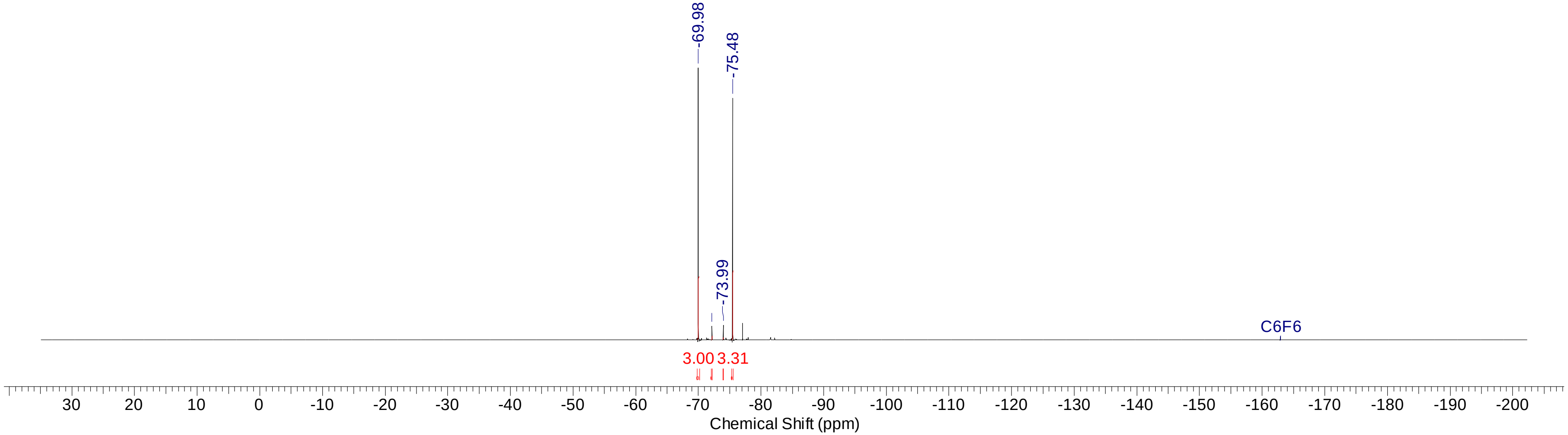

^{19}F NMR spectrum of **3g** (376.3 MHz, CD_3CN)

14 Jun 2019

Acquisition Time (sec)	2.0000	Date	Nov 1 2018	File Name	C:\DOCS\BM\SPEC_BM_F_2018.12.25\BM-1421_20181101_01\FLUORINE_01		
Frequency (MHz)	376.31	Nucleus	19F	Number of Transients	16	Original Points Count	178571
Points Count	262144	Pulse Sequence	s2pul	Solvent	CHLOROFORM-D		
Sweep Width (Hz)	89285.71	Temperature (degree C)	22.000				

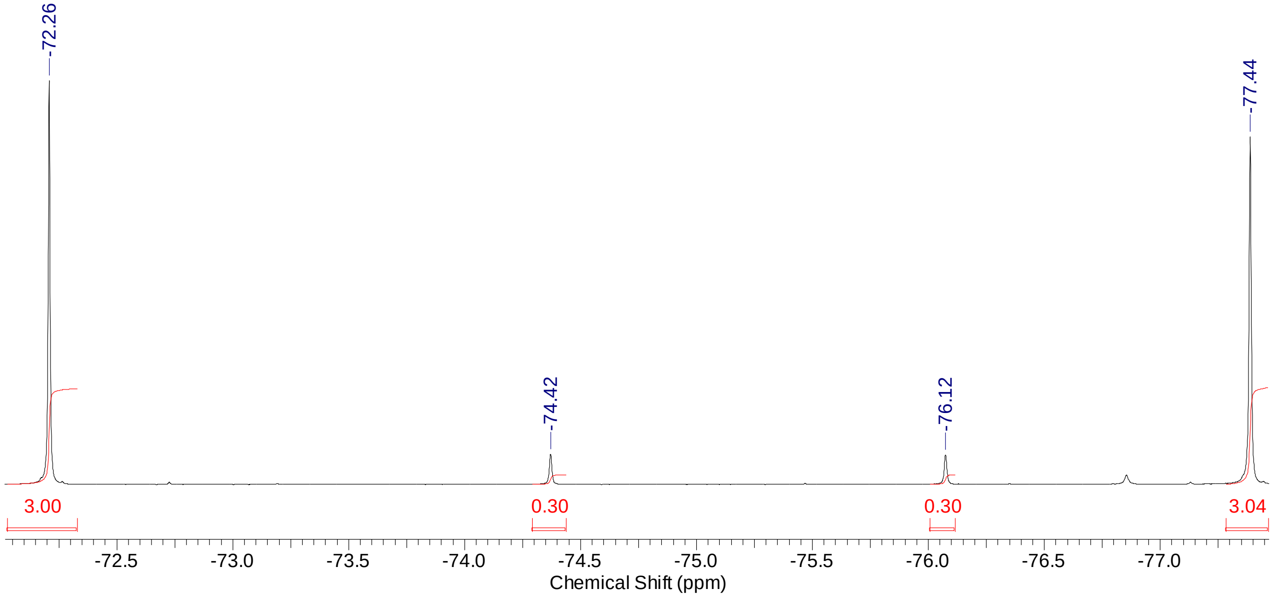

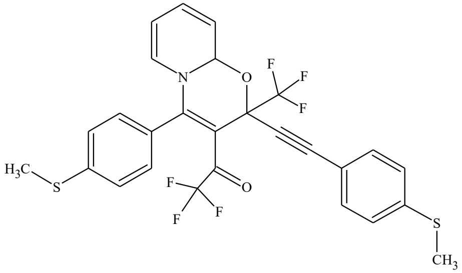

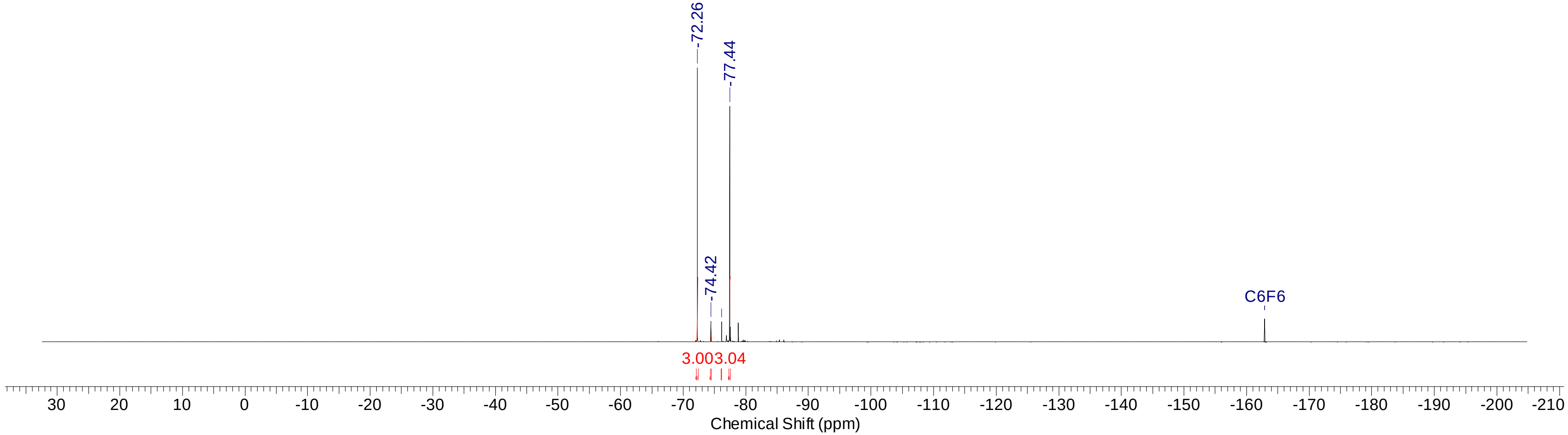

^{19}F NMR spectrum of **3g** (376.3 MHz, $CDCl_3$)

14 Jun 2019

Acquisition Time (sec)	2.5559	Comment	Imported from UXNMR.	Date	31 Oct 2018 17:11:36
File Name	C:\BM_DATA\DOCS\SPEC_BM_H,C\BM-1424.H_001001r	Frequency (MHz)	400.13	Nucleus	1H
Number of Transients	5 Original Points Count 16384	Points Count	65536	Pulse Sequence	zg30
Solvent	CHLOROFORM-D	Sweep Width (Hz)	6410.26	Temperature (degree C)	27.000

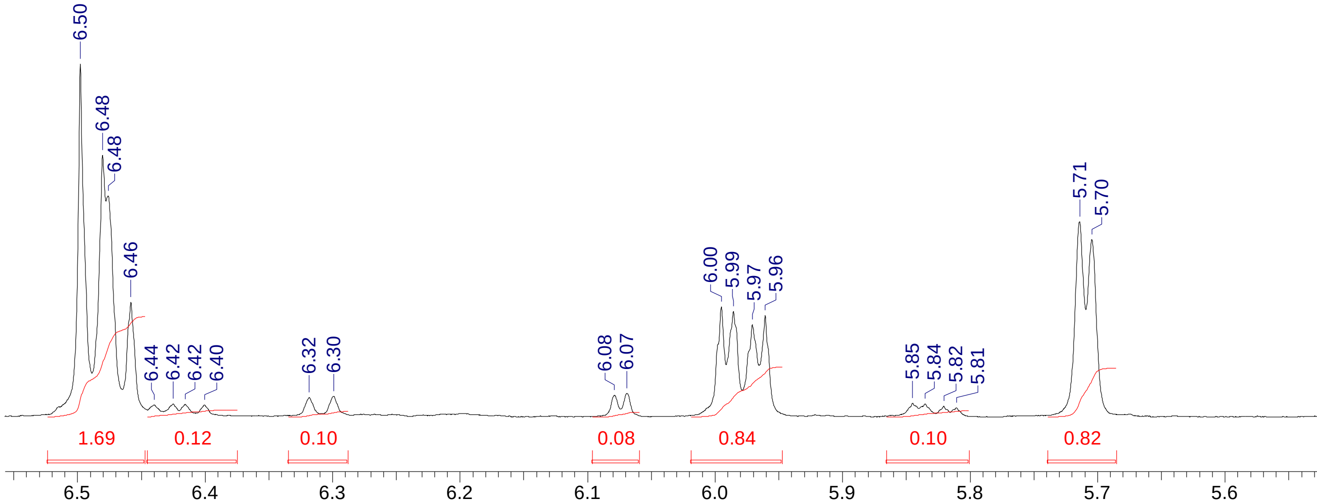

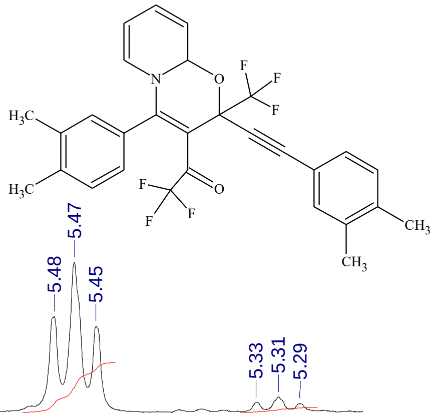

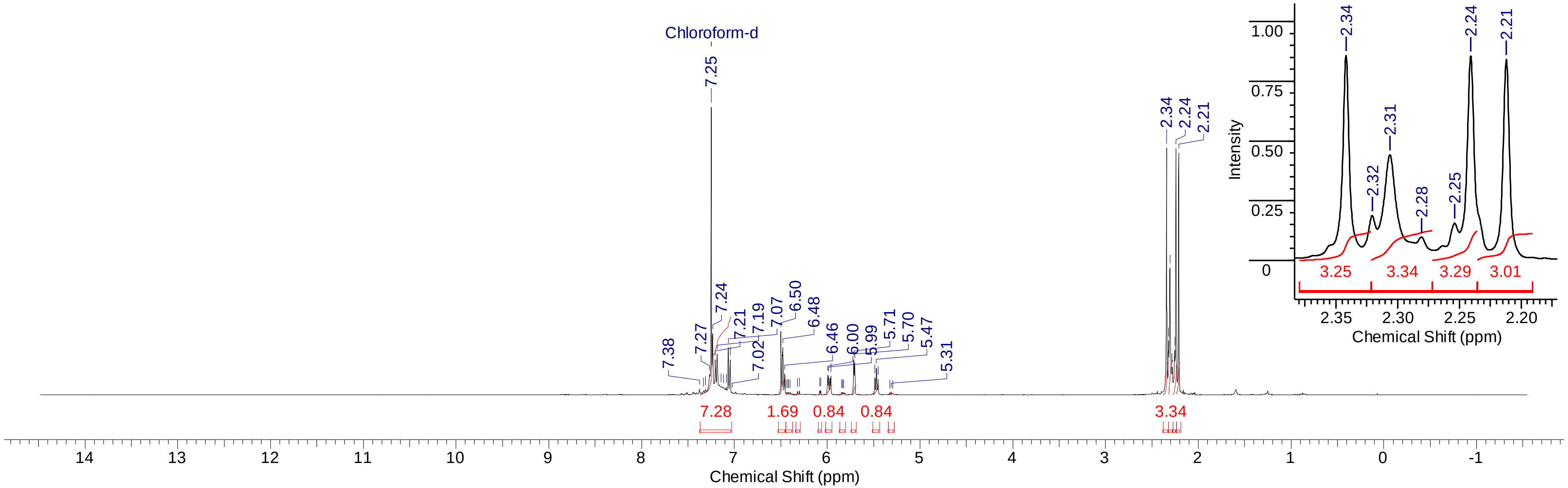

^{1}H NMR spectrum of **3h** (400.1 MHz, $CDCl_3$)

14 Jun 2019

Acquisition Time (sec)	0.4999	***Comment***	Imported from UXNMR.			***Date***	13 Nov 2018 15:22:52
File Name	C:\BM_DATA\DOCS\SPEC_BM_H,C\BM-1424-C.C_002001r					***Frequency (MHz)***	100.61
Nucleus	13C	***Number of Transients***	839	***Original Points Count***	12076	***Points Count***	65536
Pulse Sequence	zgpg30	***Solvent***	DMSO-D6	***Sweep Width (Hz)***	24154.59	***Temperature (degree C)***	27.000

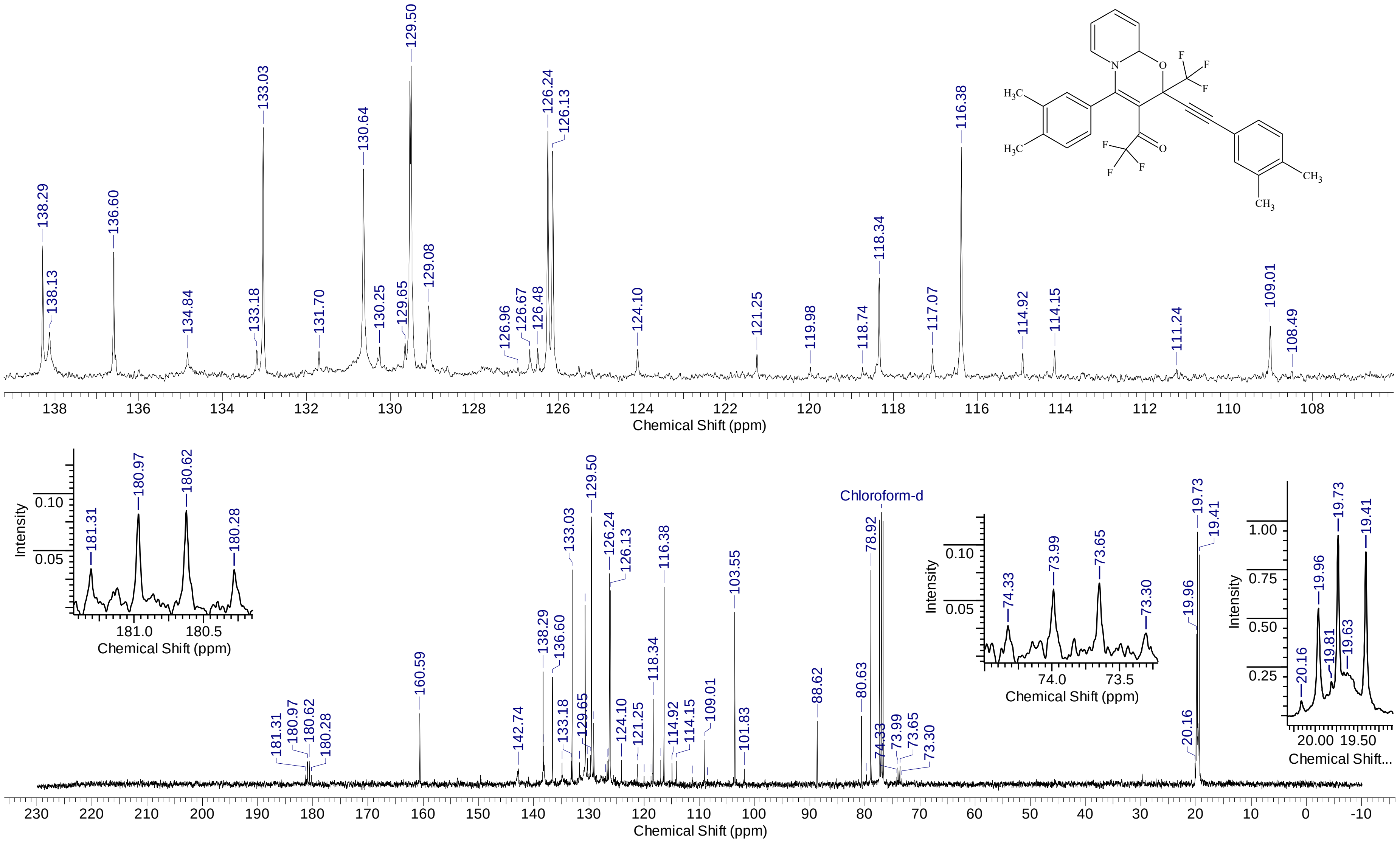

^{13}C NMR spectrum of **3h** (100.6 MHz, $CDCl_3$)

14 Jun 2019

FW	531.4889	Formula	$C_{29}H_{23}F_6NO_2$

Acquisition Time (sec)	1.3664	Comment	Imported from UXNMR.			Date	13 Nov 2018 21:24:44
File Name	C:\BM_DATA\DOCS\SPEC_BM_H,C\BM-1424-C_004001r			Frequency (MHz)	100.61	Nucleus	13C
Number of Transients	68	Original Points Count	32768	Points Count	131072	Pulse Sequence	jmod
Solvent	CHLOROFORM-D			Sweep Width (Hz)	23980.81	Temperature (degree C)	27.000

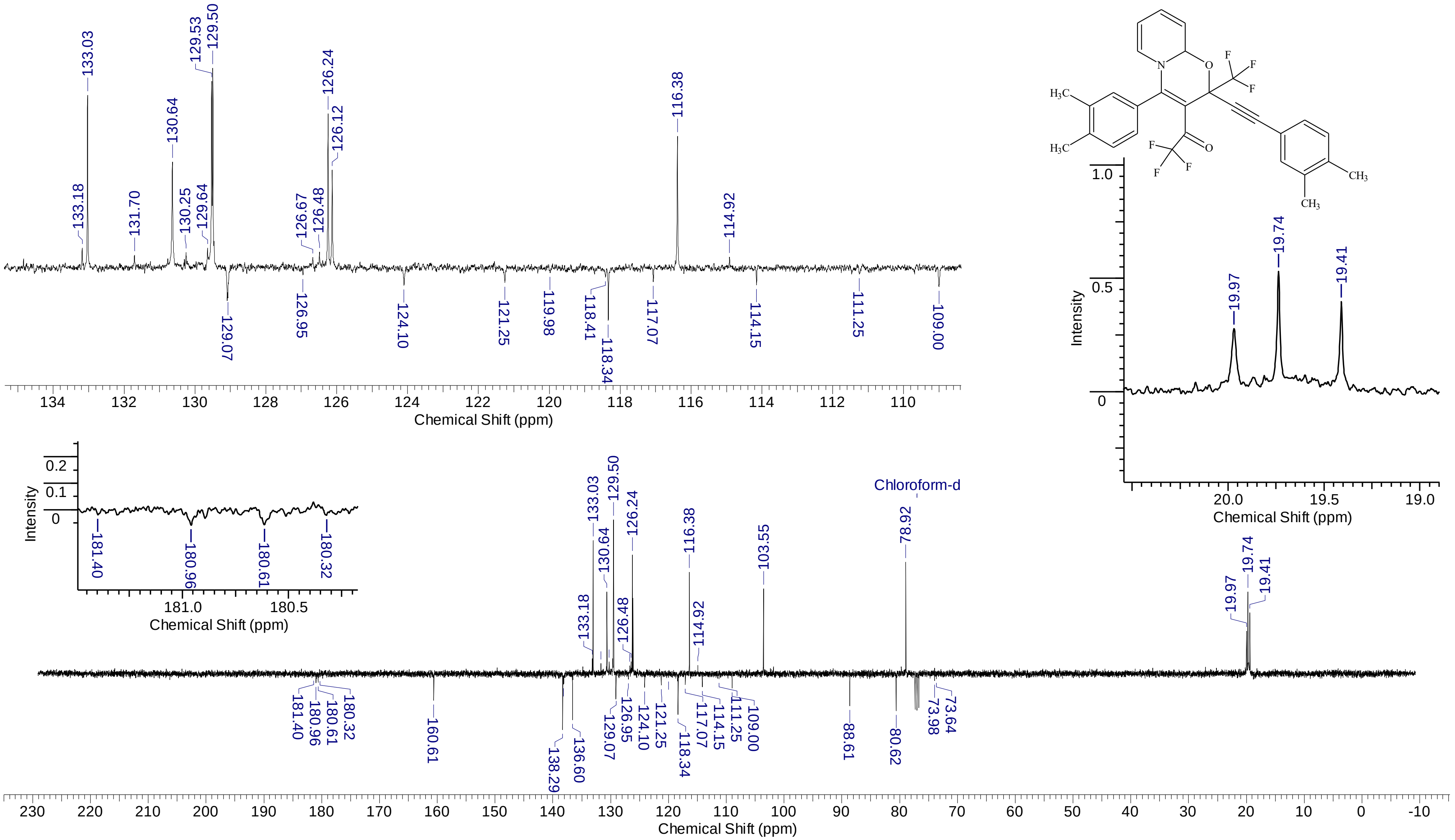

^{13}C APT NMR spectrum of **3h** (100.6 MHz, $CDCl_3$)

14 Jun 2019

Acquisition Time (sec)	2.0000	***Date***	Nov 1 2018	***File Name***	F:\COMP_PRAK\DOCS\OUTPUT_301\F19\2018.11.01\BM-1424_20181101_01\FLUORINE_01		
Frequency (MHz)	376.31	***Nucleus***	19F	***Number of Transients***	16	***Original Points Count***	178571
Points Count	262144	***Pulse Sequence***	s2pul	***Solvent***	CHLOROFORM-D		
Sweep Width (Hz)	89285.71	***Temperature (degree C)***	22.000				

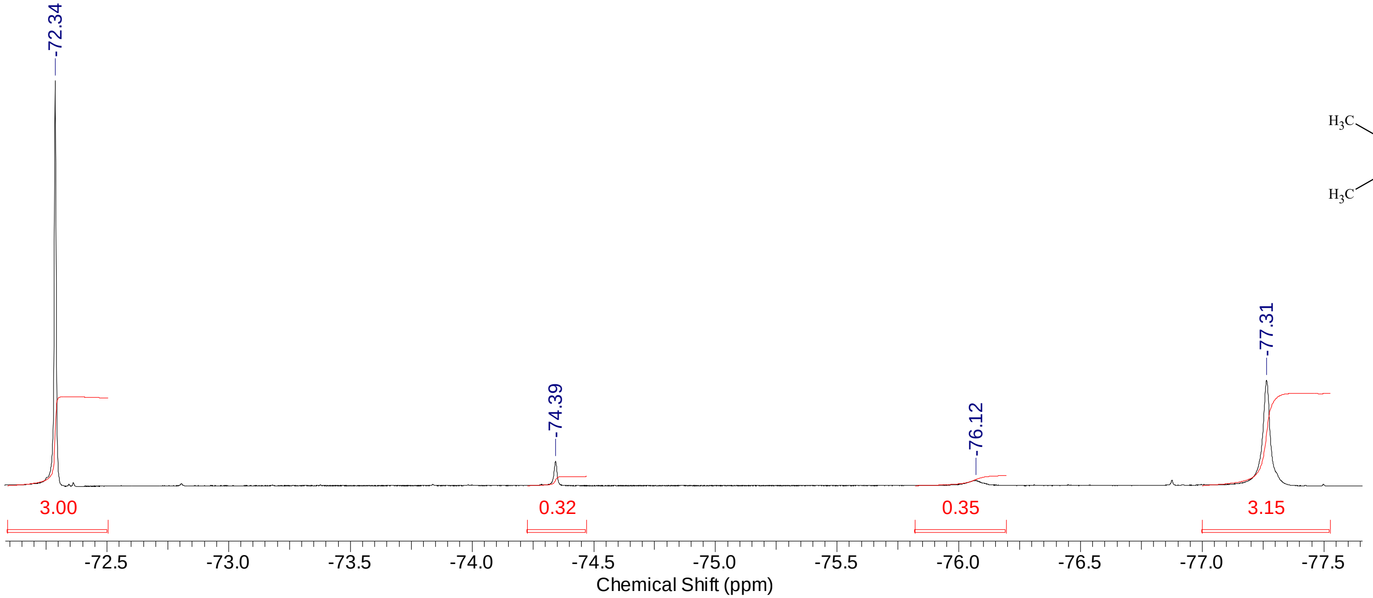

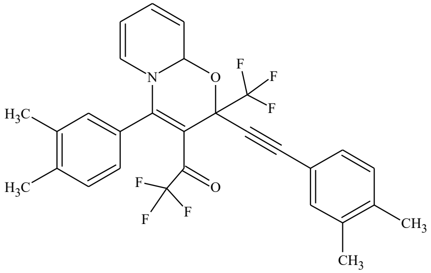

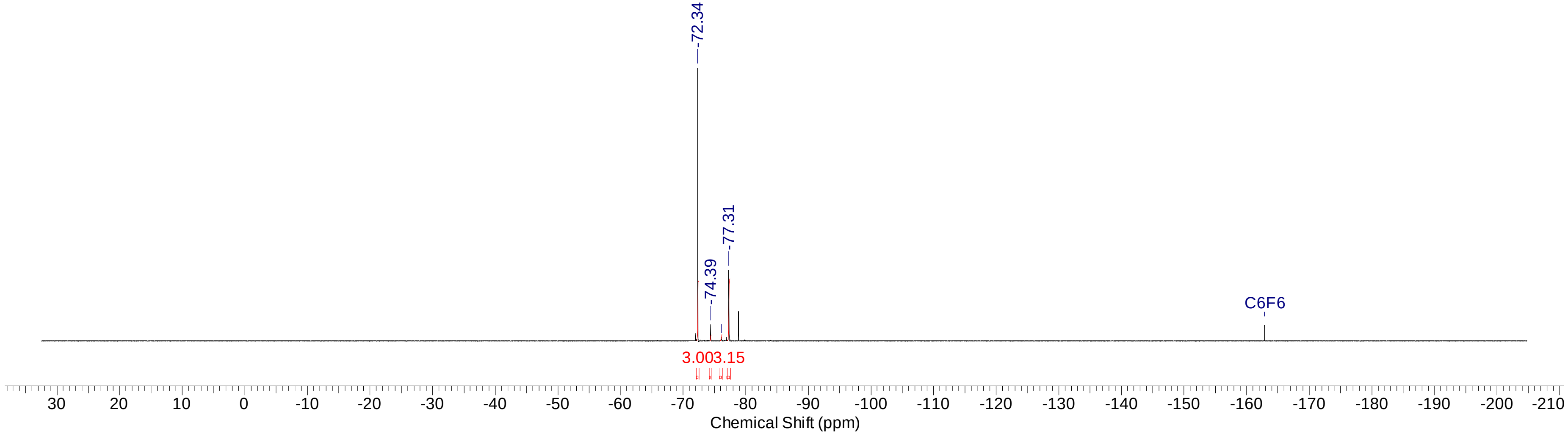

^{19}F NMR spectrum of **3h** (376.3 MHz, $CDCl_3$)

15 Jun 2019

FW	635.5519	Formula	$C_{35}H_{23}F_6NO_4$

**Acquisition Time (sec)**	2.5559	**Comment**	Imported from UXNMR.			**Date**	31 Oct 2018 17:13:52
**File Name**	C:\BM_DATA\DOCS\SPEC_BM_H,C\BM-1422.H_001001r			**Frequency (MHz)**	400.13	**Nucleus**	1H
**Number of Transients**	7	**Original Points Count**	16384	**Points Count**	65536	**Pulse Sequence**	zg30
**Solvent**	CHLOROFORM-D			**Sweep Width (Hz)**	6410.26	**Temperature (degree C)**	27.000

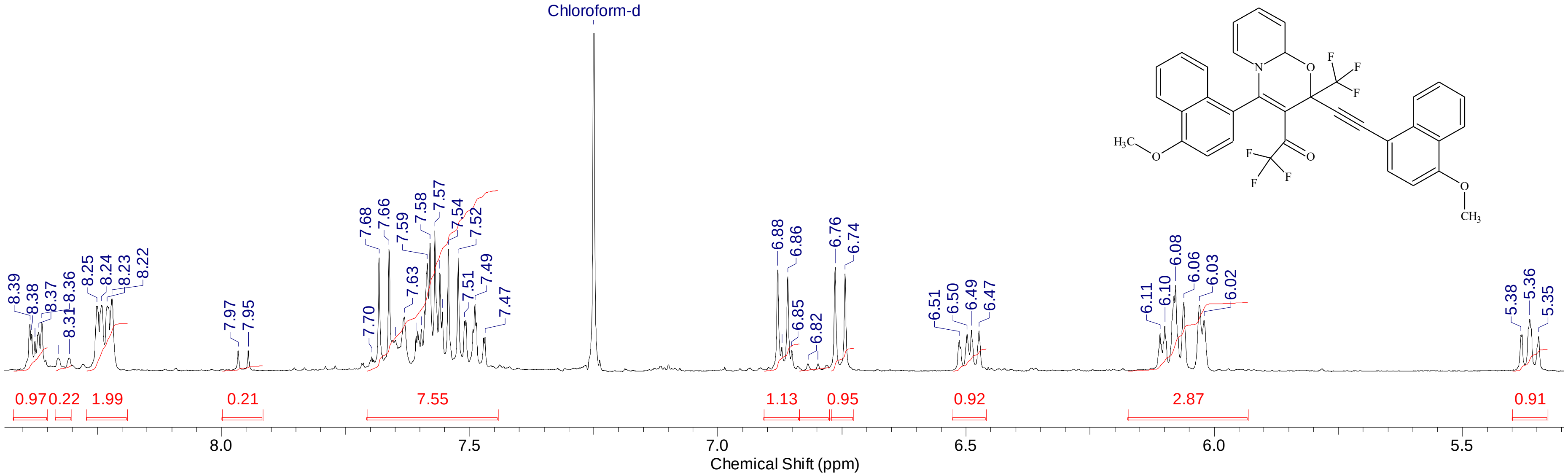

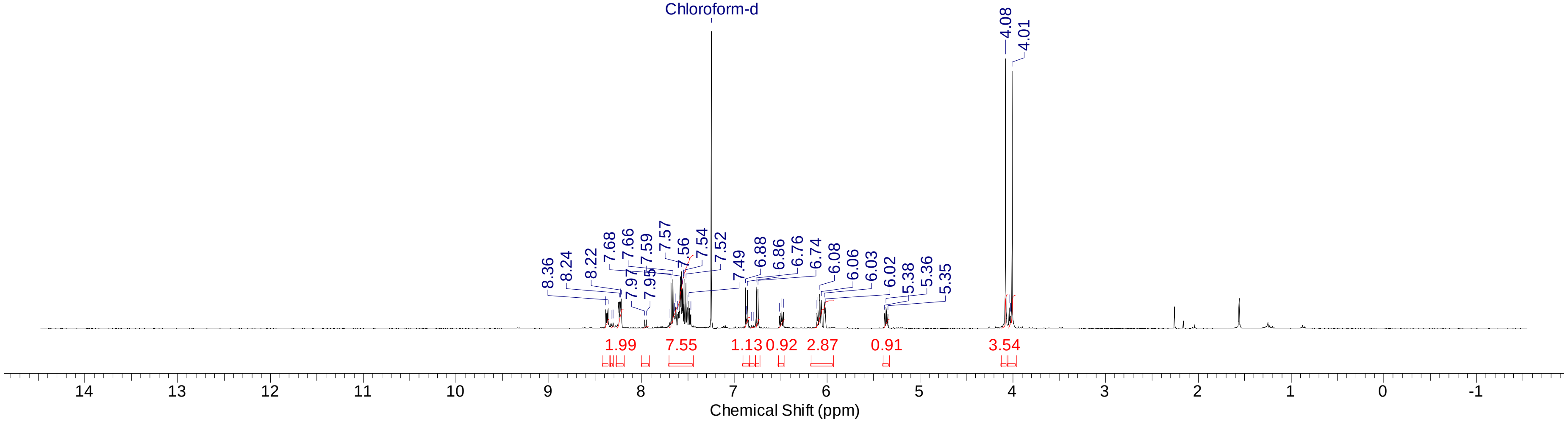

^{1}H NMR spectrum of **3i** (400.1 MHz, $CDCl_3$)

FW	635.5519	*Formula*	$C_{35}H_{23}F_6NO_4$

Acquisition Time (sec)	0.4999	***Comment***	Imported from UXNMR.			***Date***	13 Nov 2018 14:57:20
File Name	C:\BM_DATA\DOCS\SPEC_BM_H,C\BM-1422-C.C_002001r					***Frequency (MHz)***	100.61
Nucleus	13C	***Number of Transients***	1121	***Original Points Count***	12076	***Points Count***	65536
Pulse Sequence	zgpg30	***Solvent***	DMSO-D6	***Sweep Width (Hz)***	24154.59	***Temperature (degree C)***	27.000

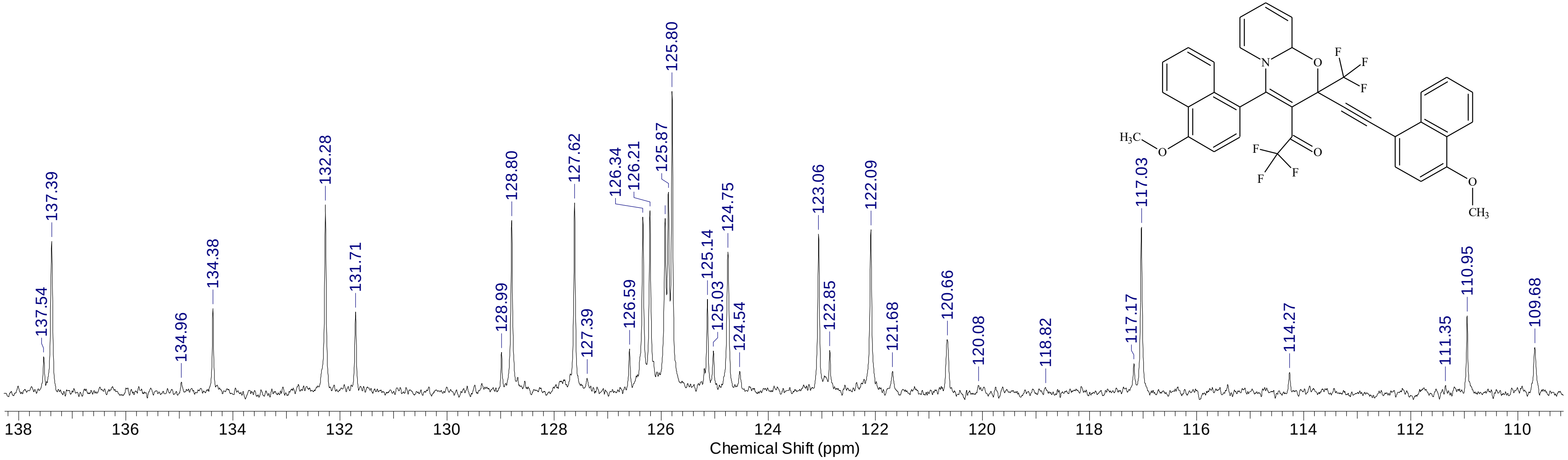

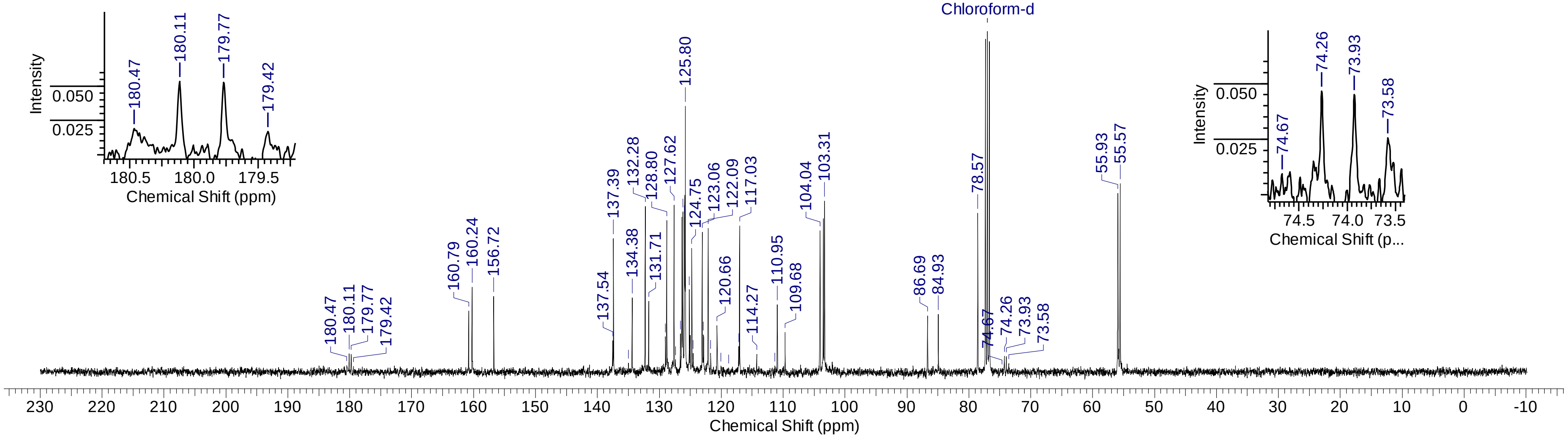

^{13}C NMR spectrum of **3i** (100.6 MHz, $CDCl_3$)

FW	635.5519	Formula	$C_{35}H_{23}F_6NO_4$

Acquisition Time (sec)	1.3664	Comment	Imported from UXNMR.			Date	13 Nov 2018 21:32:04
File Name	C:\BM_DATA\DOCS\SPEC_BM_H,C\bm181113\BM-1422-C_004001r					Frequency (MHz)	100.61
Nucleus	13C	Number of Transients	64	Original Points Count	32768	Points Count	131072
Pulse Sequence	jmod	Solvent	CHLOROFORM-D			Sweep Width (Hz)	23980.81
Temperature (degree C)	27.000						

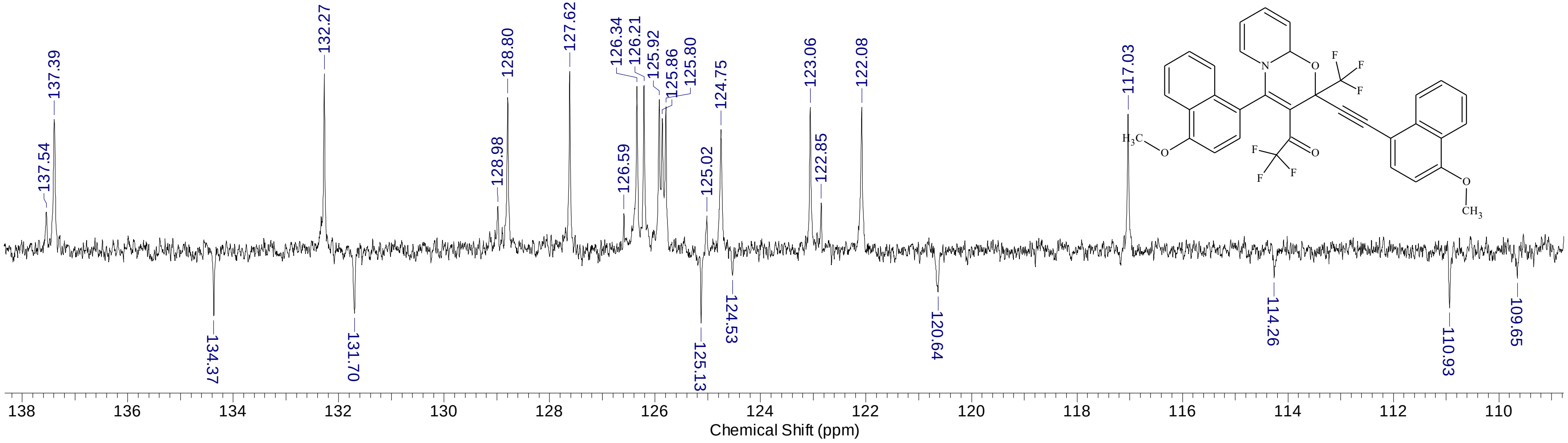

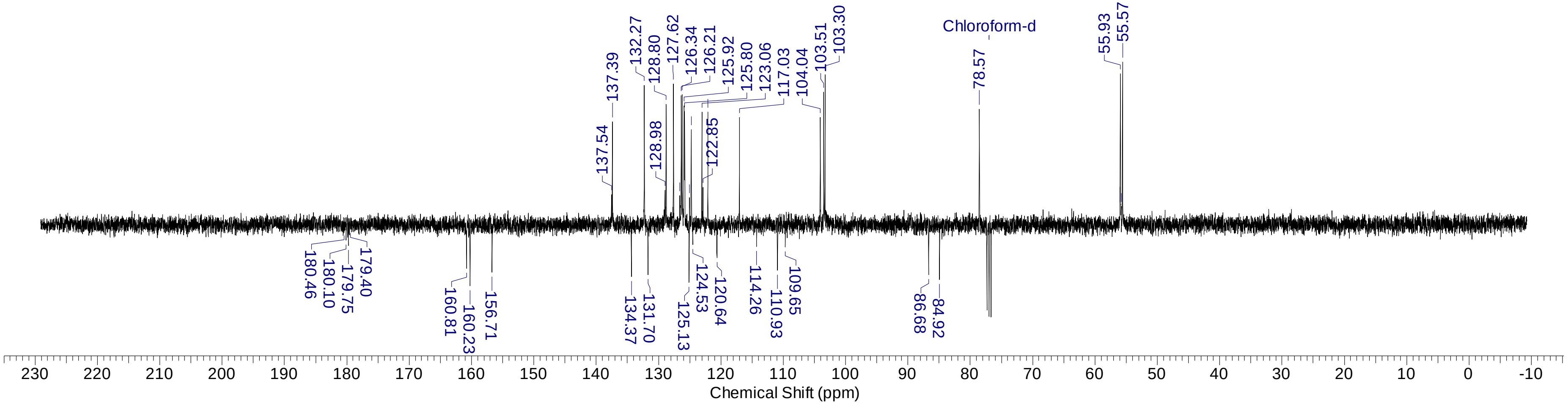

^{13}C APT NMR spectrum of **3i** (100.6 MHz, $CDCl_3$)

15 Jun 2019

FW	635.5519	***Formula***	$C_{35}H_{23}F_6NO_4$

Acquisition Time (sec)	2.0000	***Date***	Nov 1 2018	***File Name***	F:\COMP_PRAK\DOCS\OUTPUT_301\F19\2018.11.01\BM-1422_20181101_01\FLUORINE_01		
Frequency (MHz)	376.31	***Nucleus***	19F	***Number of Transients***	16	***Original Points Count***	178571
Points Count	262144	***Pulse Sequence***	s2pul	***Solvent***	CHLOROFORM-D		
Sweep Width (Hz)	89285.71	***Temperature (degree C)***	22.000				

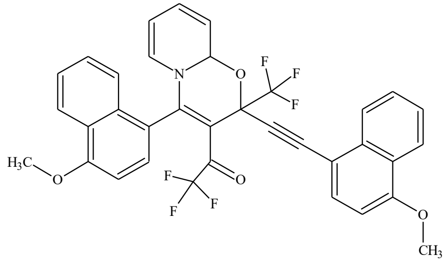

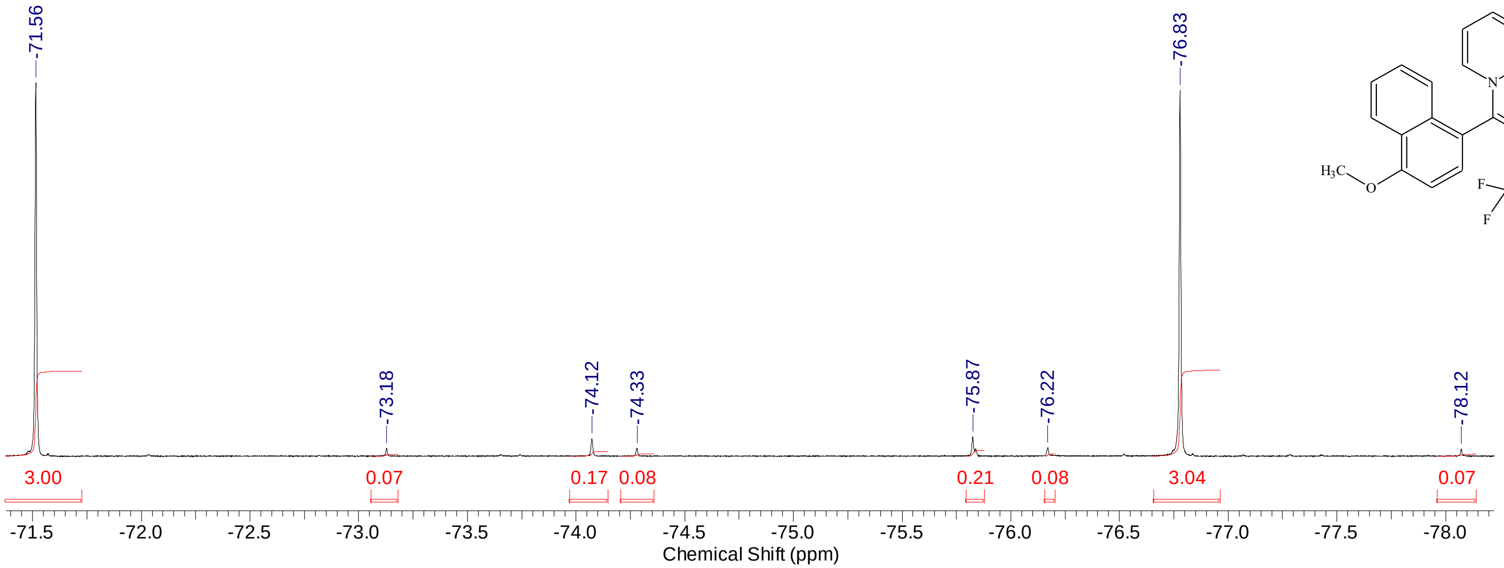

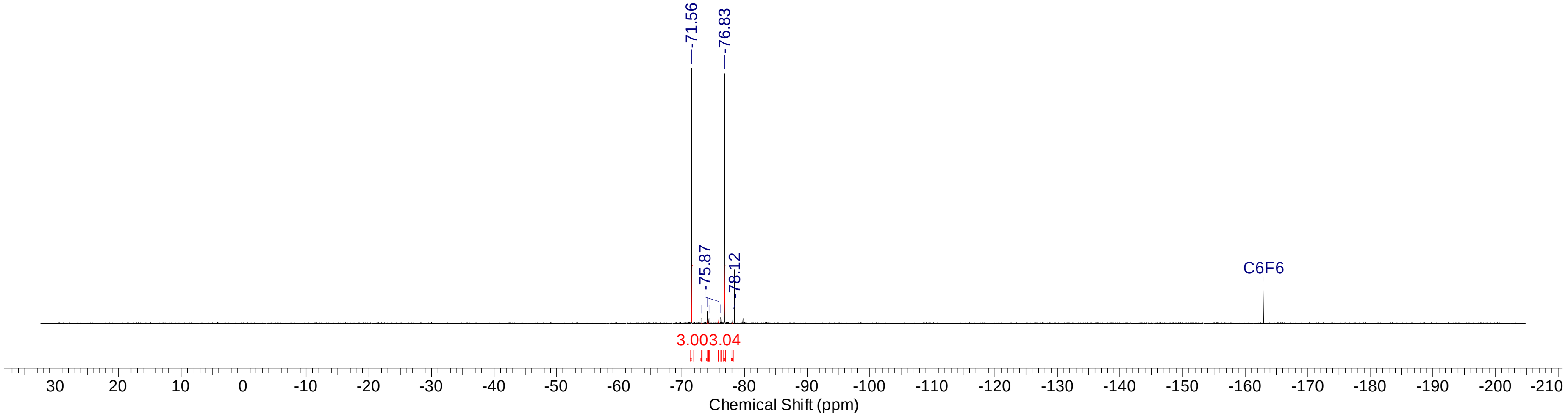

^{19}F NMR spectrum of **3i** (376.3 MHz, $CDCl_3$)

1 Sep 2019

Acquisition Time (sec)	4.0894	***Comment***	Imported from UXNMR.	***Date***	10 Apr 2019 17:07:26
File Name	F:\COMP_PRAK\DOCS\OUTPUT_301\2019\04.àï ðåëü\SZA-093-1p.H_001001r			***Frequency (MHz)***	400.13
Nucleus	1H	***Number of Transients***	4 ***Original Points Count*** 32768	***Points Count***	131072
Pulse Sequence	zg30	***Solvent***	CHLOROFORM-D	***Sweep Width (Hz)***	8012.82
Temperature (degree C)	27.000				

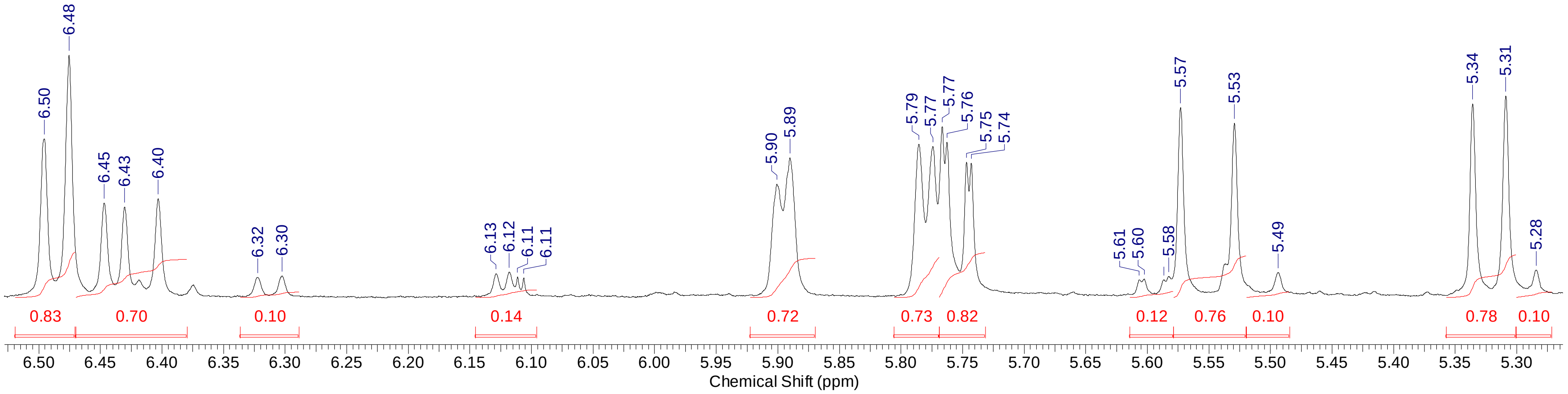

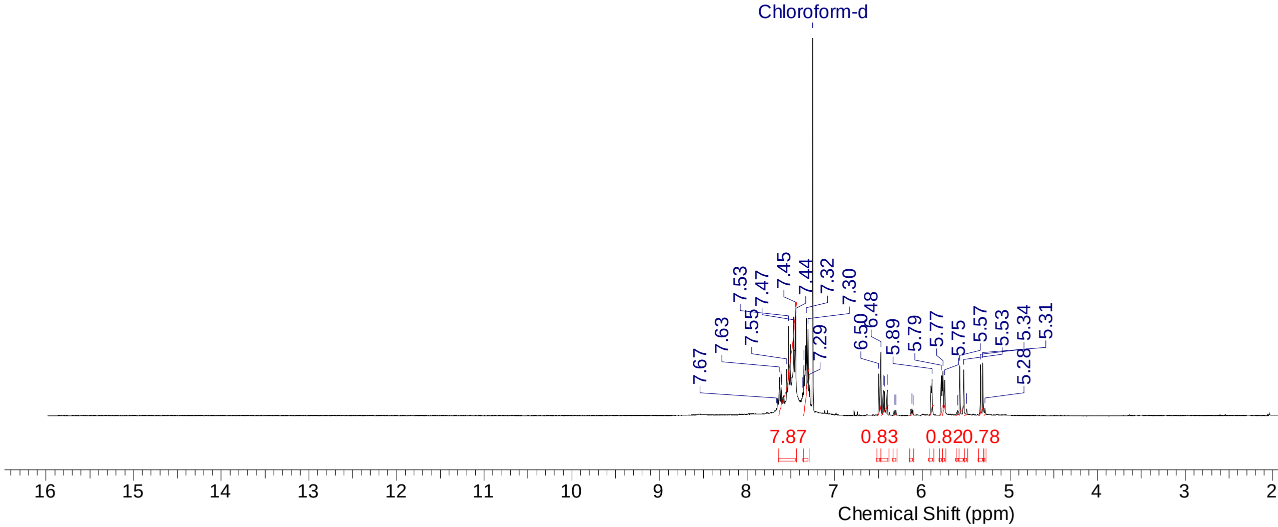

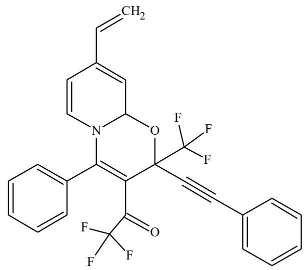

^{1}H NMR spectrum of **3j** (400.1 MHz, $CDCl_3$)

Acquisition Time (sec)	0.6783	Comment	Imported from UXNMR.			Date	12 Apr 2019 15:41:54
File Name	F:\COMP_PRAK\DOCS\OUTPUT_301\2019\04.àï ðåëü\SZA-093-1p-2.C_002001r					Frequency (MHz)	100.61
Nucleus	13C	Number of Transients	353	Original Points Count	16384	Points Count	131072
Pulse Sequence	zgpg30	Solvent	CHLOROFORM-D			Sweep Width (Hz)	24154.59
Temperature (degree C)	27.000						

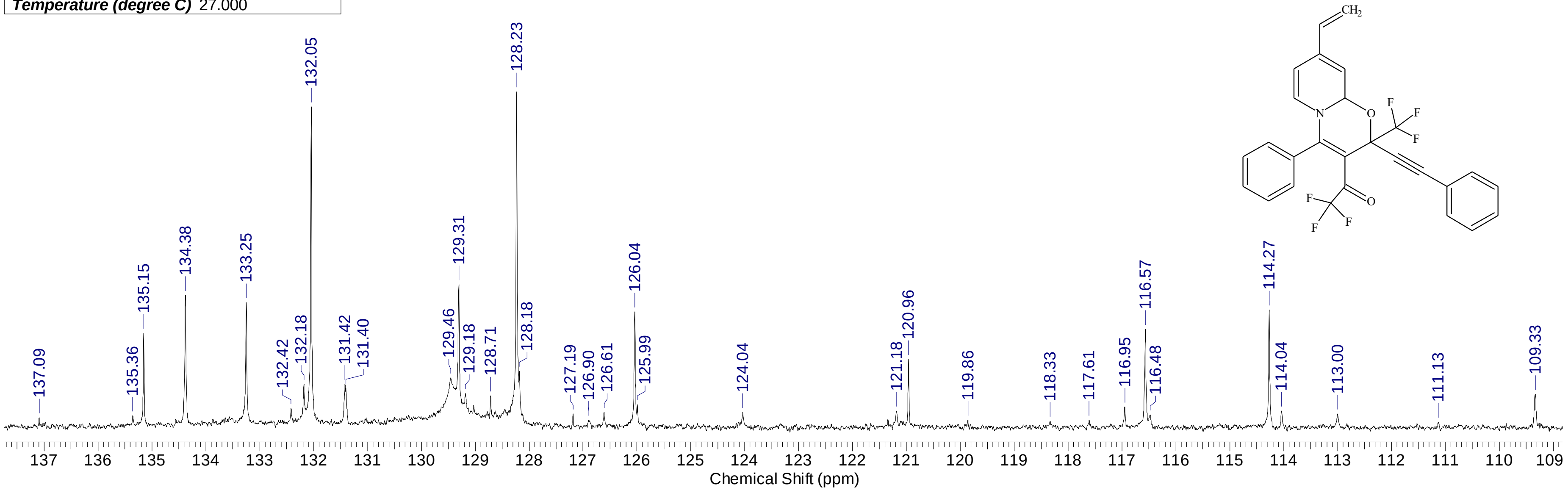

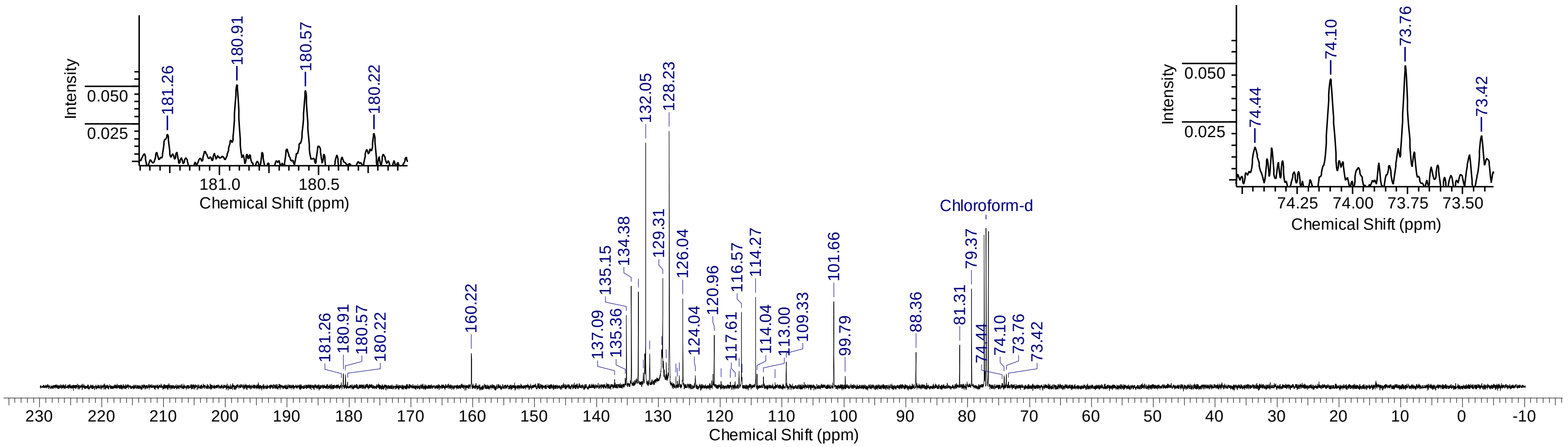

^{13}C NMR spectrum of **3j** (100.6 MHz, $CDCl_3$)

Acquisition Time (sec)	2.3069	***Date***	Apr 11 2019	***File Name***	C:\DOCS\OUTPUT_301\F19\2019.04.11\SZA-093-1P-F_20190411_01\FLUORINE_01		
Frequency (MHz)	376.32	***Nucleus***	19F	***Number of Transients***	8	***Original Points Count***	262144
Points Count	262144	***Pulse Sequence***	s2pul	***Solvent***	CHLOROFORM-D		
Sweep Width (Hz)	113636.37	***Temperature (degree C)***	22.000				

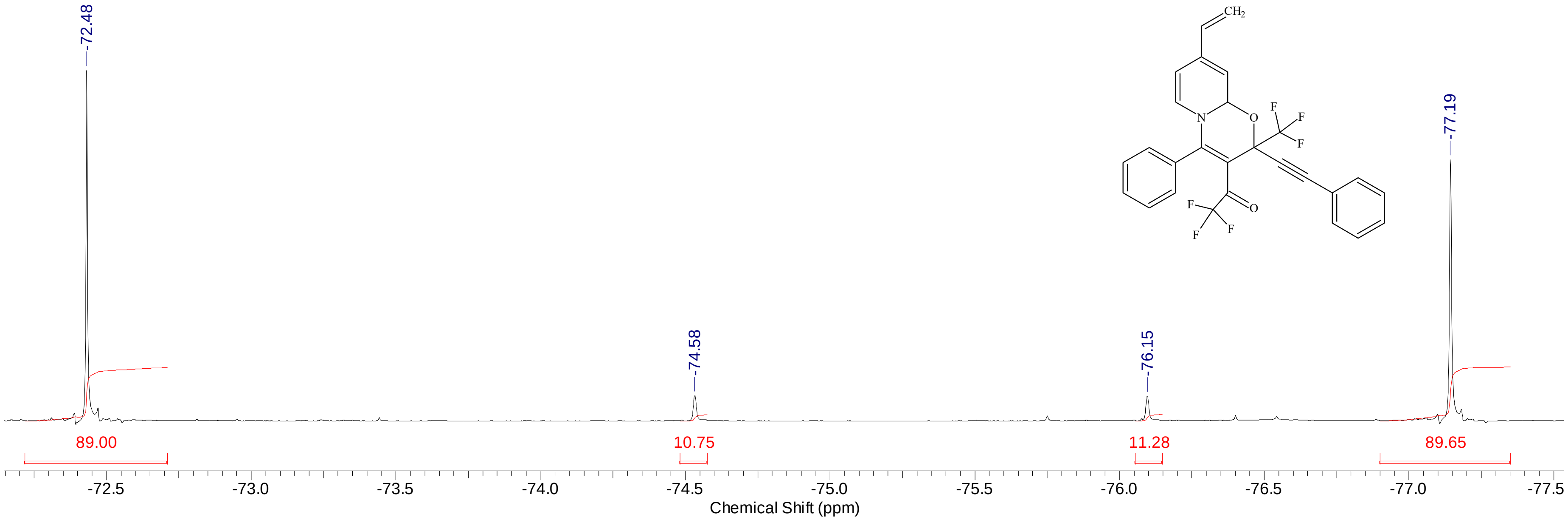

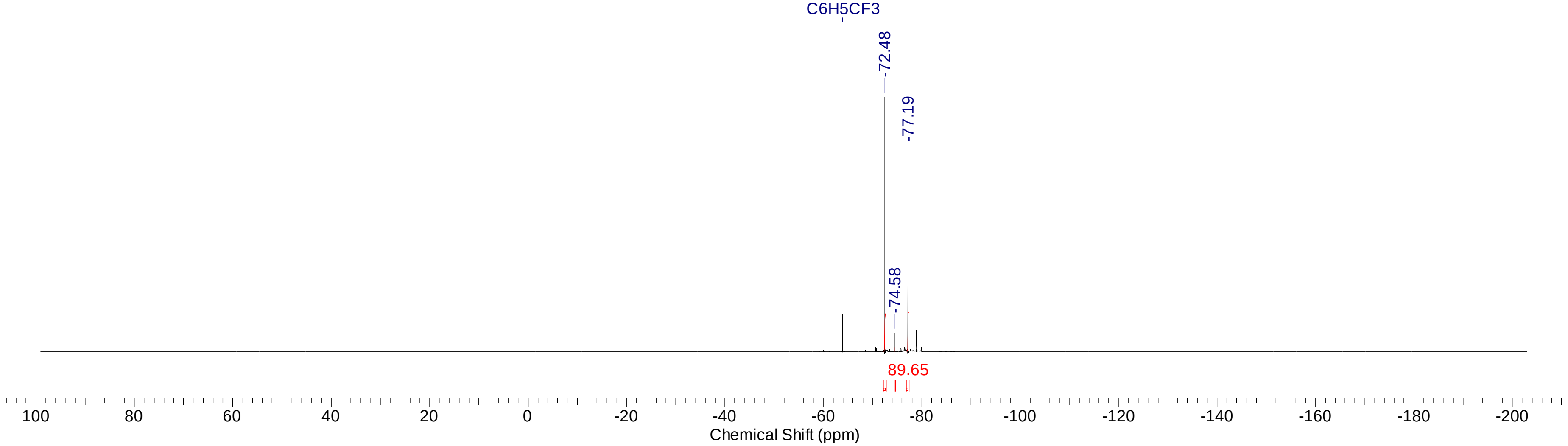

^{19}F NMR spectrum of **3j** (376.3 MHz, $CDCl_3$)

17 Jun 2019

FW 503.3927	*Formula* $C_{26}H_{15}F_6NO_3$

Acquisition Time (sec)	4.0894	***Comment***	Imported from UXNMR.	***Date***	19 Jan 2019 16:59:38
File Name	F:\COMP_PRAK\DOCS\OUTPUT_301\2019\01.ÿí âàðü\190119 (1)\BM-1496-2_001001r			***Frequency (MHz)***	400.13
Nucleus	1H	***Number of Transients***	8 ***Original Points Count*** 32768	***Points Count***	131072
Pulse Sequence	zg30	***Solvent***	ACETONITRILE-D3	***Sweep Width (Hz)***	8012.82
Temperature (degree C)	27.000				

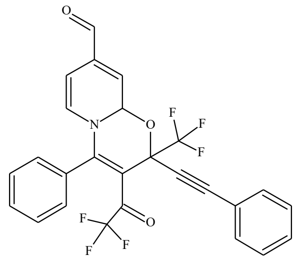

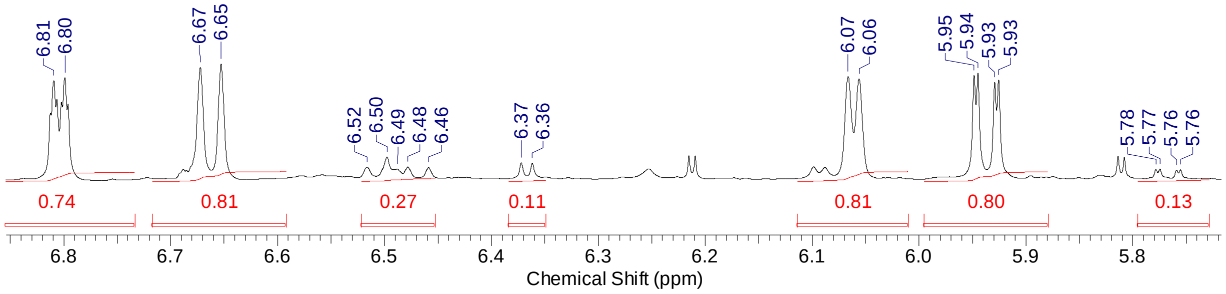

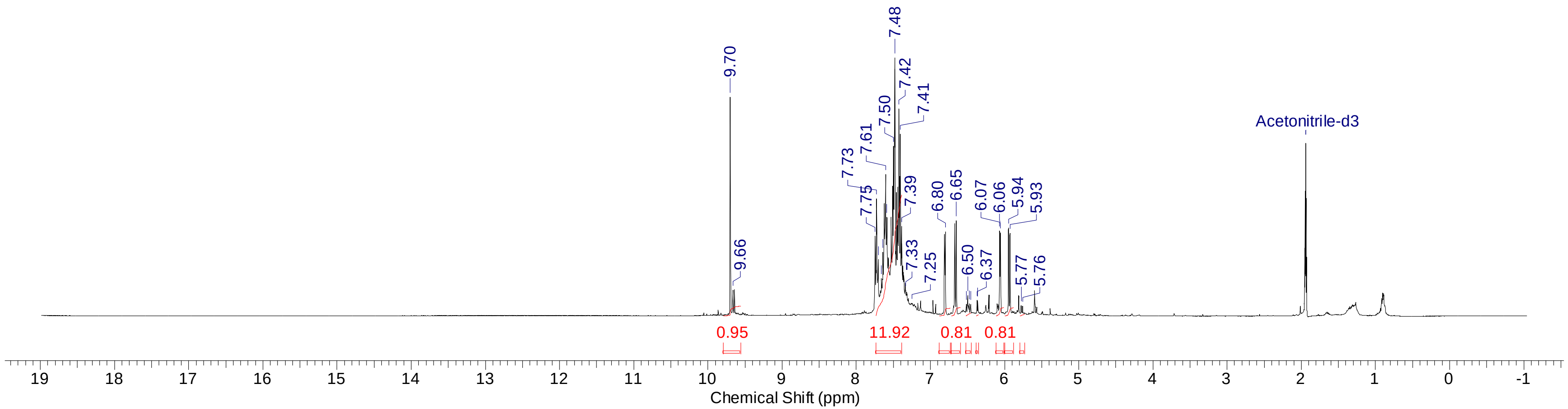

^{1}H NMR spectrum of **3k** (400.1 MHz, CD_3CN)

17 Jun 2019

FW	503.3927	Formula $C_{26}H_{15}F_6NO_3$

Acquisition Time (sec)	0.6783	Comment	Imported from UXNMR.			Date	19 Jan 2019 17:07:42
File Name	F:\COMP_PRAK\DOCS\OUTPUT_301\2019\01.ÿí âàðü\190119 (1)\BM-1496-2_002001r					Frequency (MHz)	100.61
Nucleus	13C	Number of Transients	136	Original Points Count	16384	Points Count	131072
Pulse Sequence	zgpg30	Solvent	CHLOROFORM-D	Sweep Width (Hz)	24154.59	Temperature (degree C)	27.000

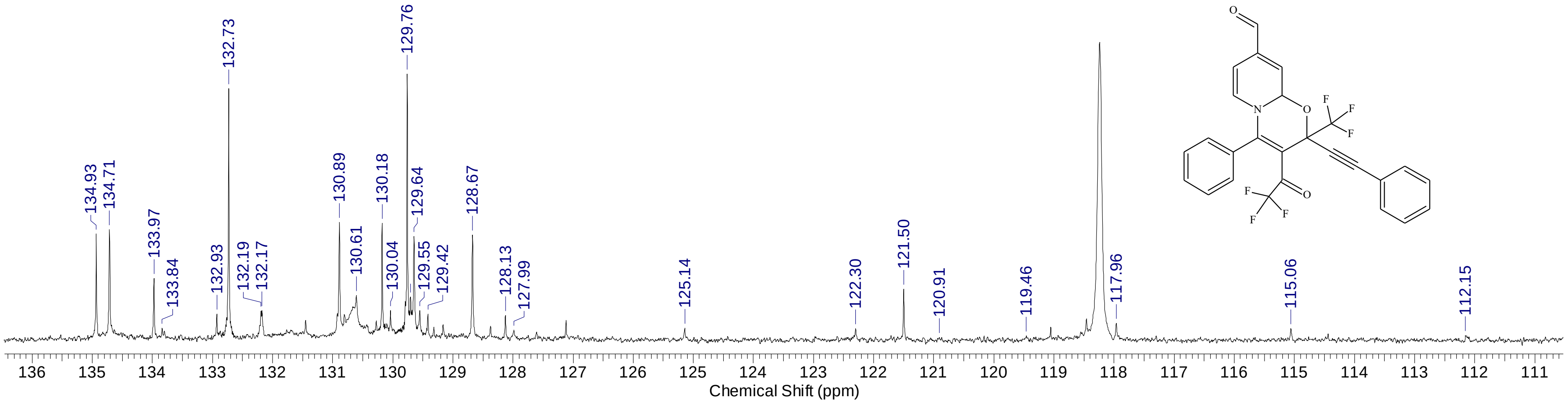

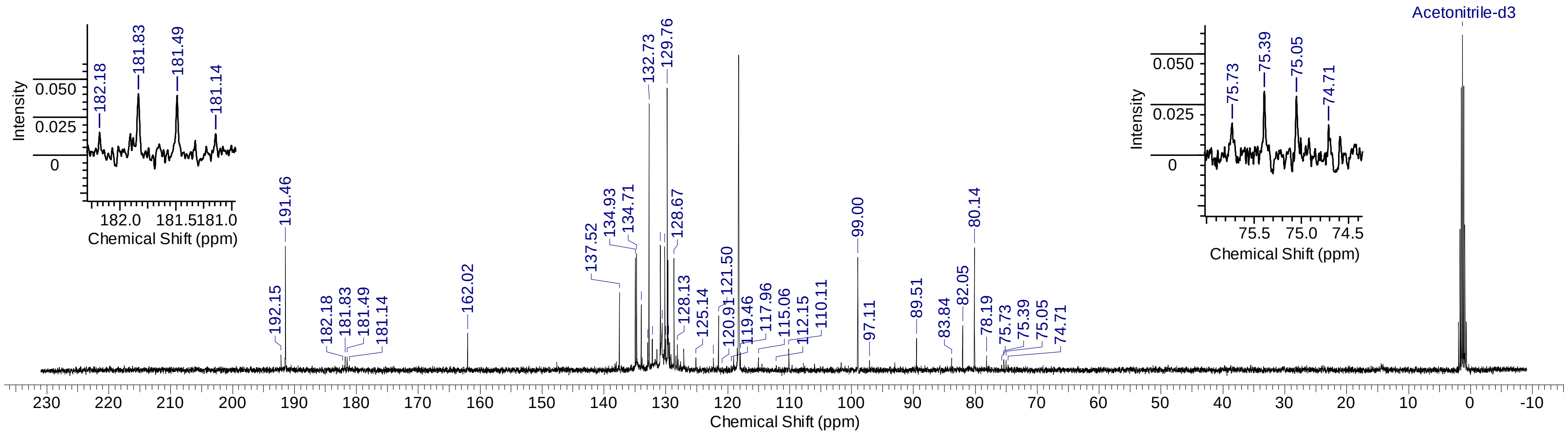

^{13}C NMR spectrum of **3k** (100.6 MHz, CD_3CN)

17 Jun 2019

FW	503.3927	***Formula***	$C_{26}H_{15}F_6NO_3$

Acquisition Time (sec)	1.3664	***Comment***	Imported from UXNMR.			***Date***	21 Jan 2019 15:54:40
File Name	F:\COMP_PRAK\DOCS\OUTPUT_301\2019\01.ÿí âàðü\BM-1496-2.APT_004001r					***Frequency (MHz)***	100.61
Nucleus	13C	***Number of Transients***	325	***Original Points Count***	32768	***Points Count***	131072
Pulse Sequence	jmod	***Solvent***	ACETONITRILE-D3			***Sweep Width (Hz)***	23980.81
Temperature (degree C)	27.000						

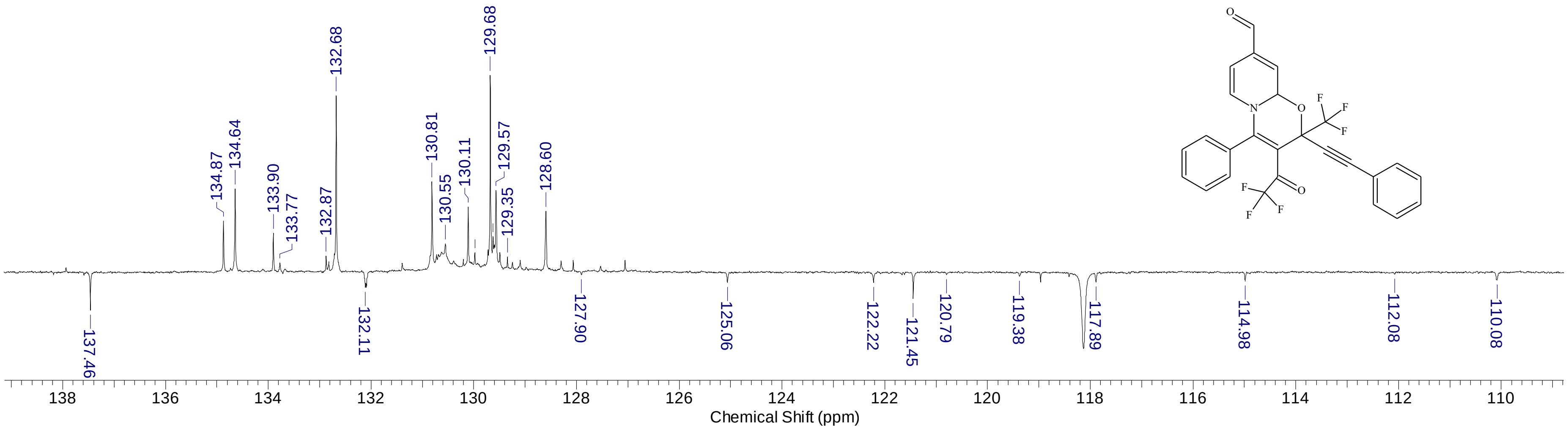

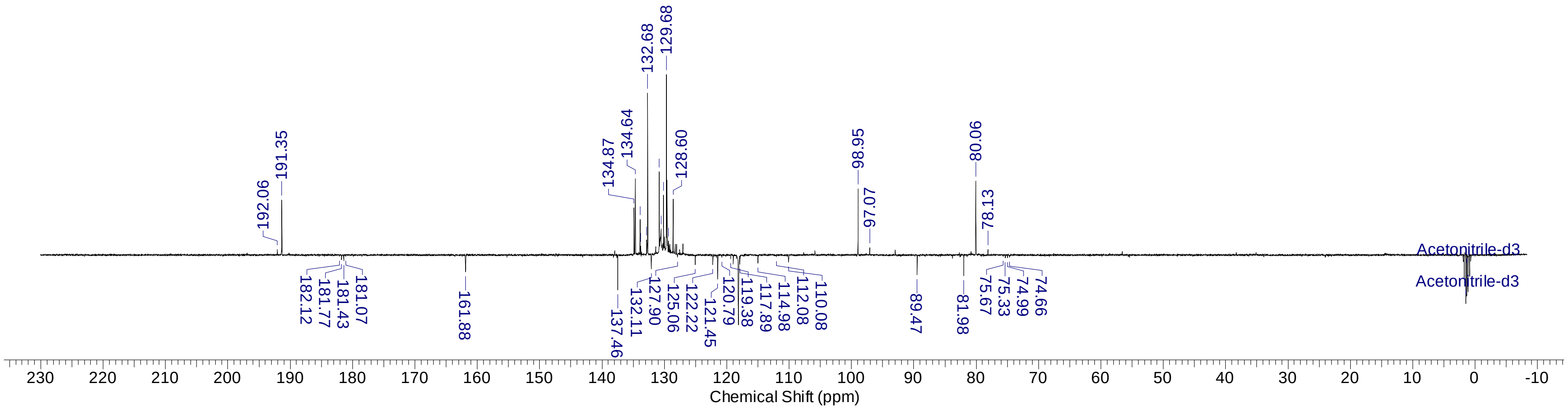

^{13}C APT NMR spectrum of **3k** (100.6 MHz, CD_3CN)

17 Jun 2019

FW	503.3927	Formula	$C_{26}H_{15}F_6NO_3$

Acquisition Time (sec)	1.5000	Date	Jan 21 2019	File Name	F:\COMP_PRAK\DOCS\OUTPUT_301\F19\2019.01.21\BM-1496-2_20190121_01\FLUORINE_01		
Frequency (MHz)	376.31	Nucleus	19F	Number of Transients	16	Original Points Count	133929
Points Count	262144	Pulse Sequence	s2pul	Solvent	ACETONITRILE-D3		
Sweep Width (Hz)	89285.71	Temperature (degree C)	22.000				

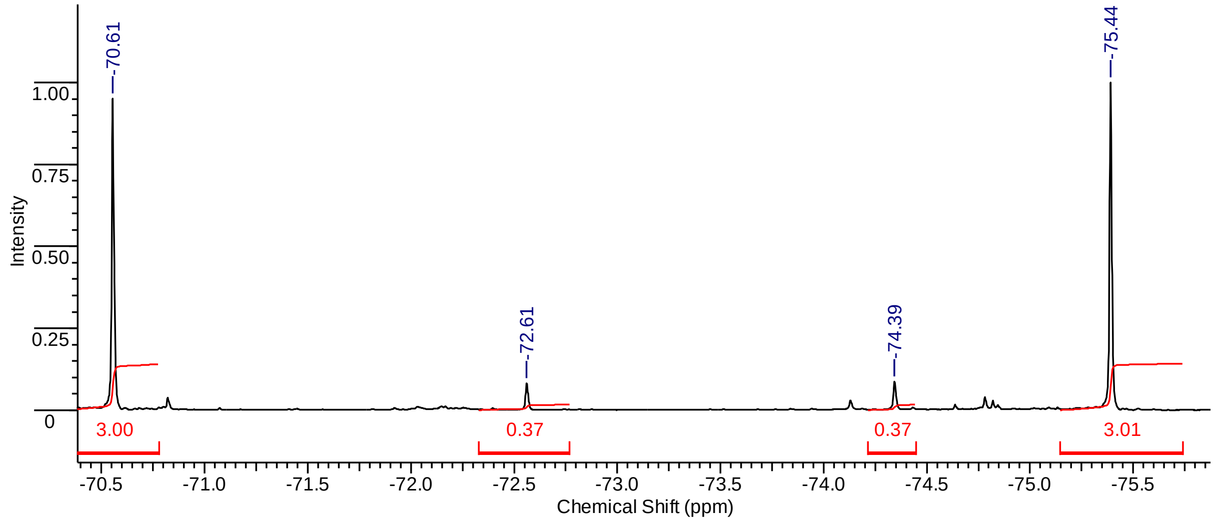

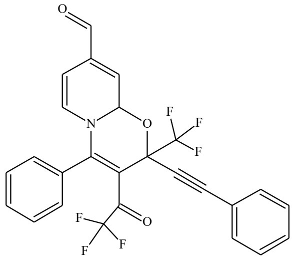

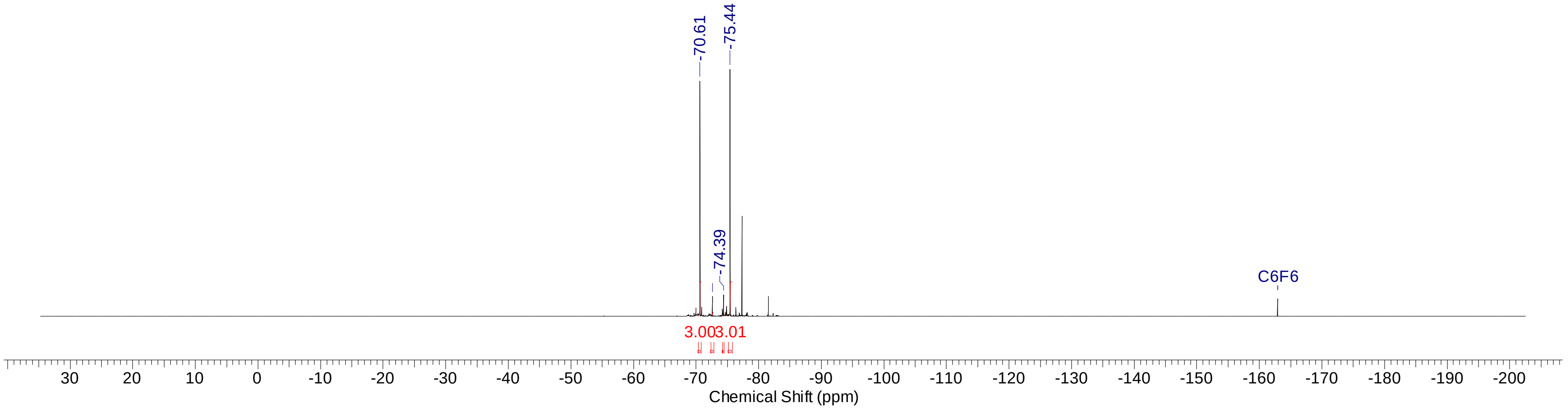

^{19}F NMR spectrum of **3k** (376.3 MHz, CD_3CN)

17 Jun 2019

FW	503.3927	Formula	$C_{26}H_{15}F_6NO_3$

Acquisition Time (sec)	2.3069	Date	Jan 24 2019	File Name	F:\COMP_PRAK\DOCS\OUTPUT_301\F19\2019.01.24\BM-1498-F_20190124_01\FLUORINE_01		
Frequency (MHz)	376.31	Nucleus	19F	Number of Transients	8	Original Points Count	221814
Points Count	262144	Pulse Sequence	s2pul	Solvent	CHLOROFORM-D		
Sweep Width (Hz)	96153.84	Temperature (degree C)	22.000				

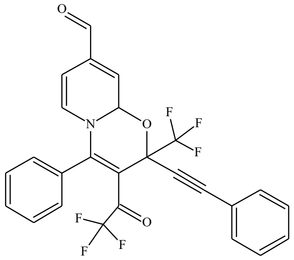

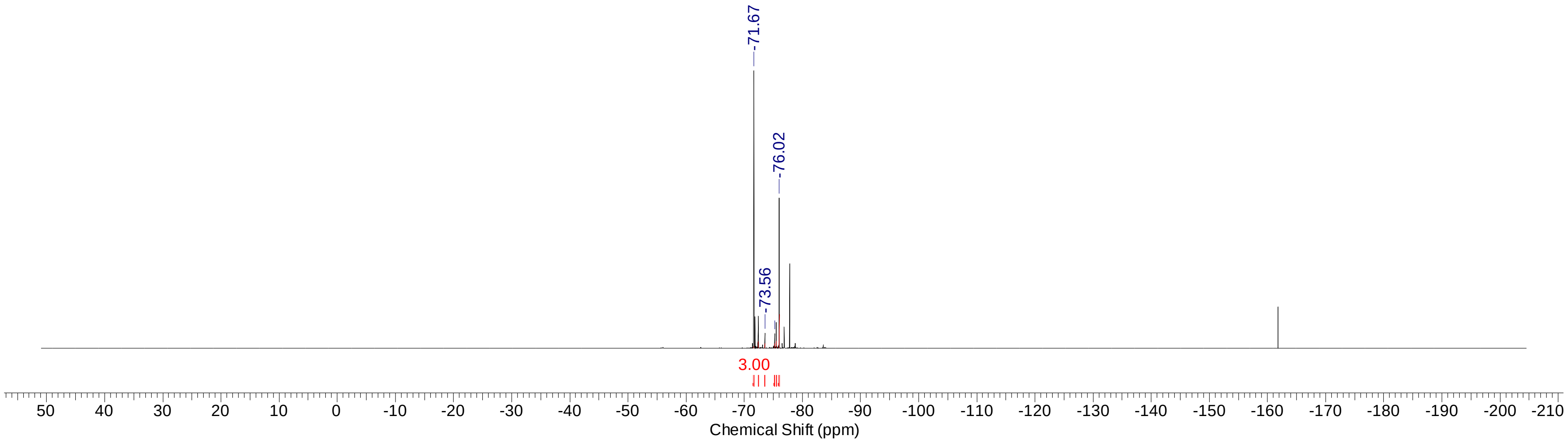

^{19}F NMR spectrum of **3k** (376.3 MHz, $CDCl_3$)

29 Aug 2019

**Acquisition Time (sec)**	4.0894	**Comment**	Imported from UXNMR.			**Date**	22 Jan 2019 14:16:22
**File Name**	I:\SPEC_2019_H,C\01.ÿí âàðü\SZA-048-1.H_001001r			**Frequency (MHz)**	400.13	**Nucleus**	1H
**Number of Transients**	5	**Original Points Count**	32768	**Points Count**	131072	**Pulse Sequence**	zg30
**Solvent**	CHLOROFORM-D			**Sweep Width (Hz)**	8012.82	**Temperature (degree C)**	27.000

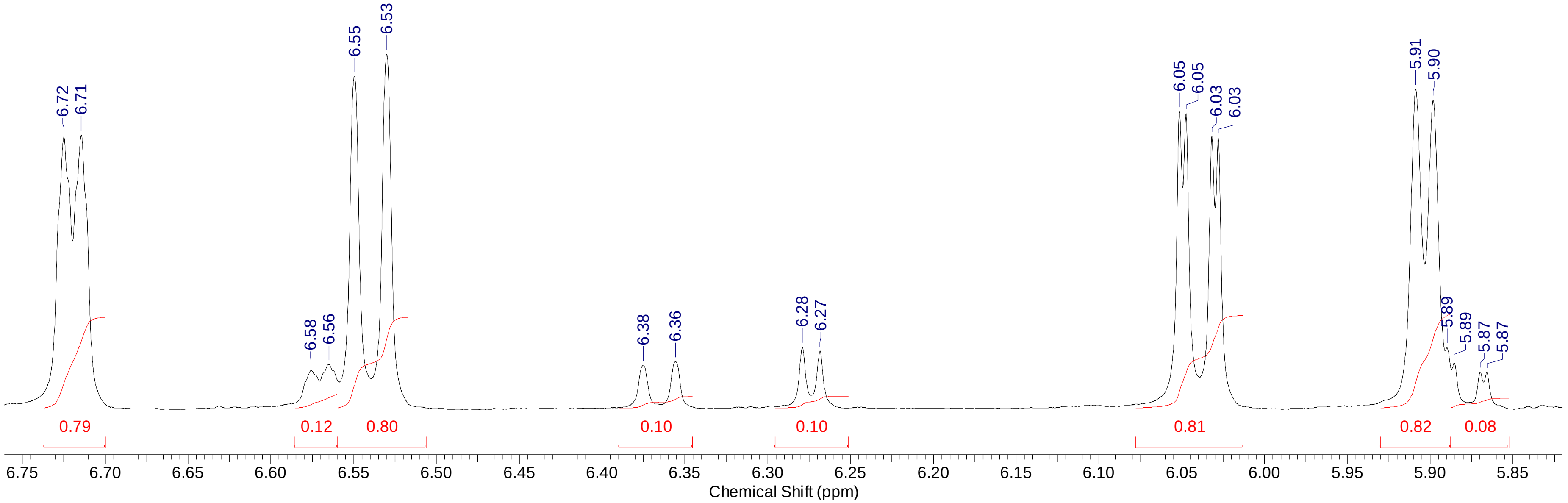

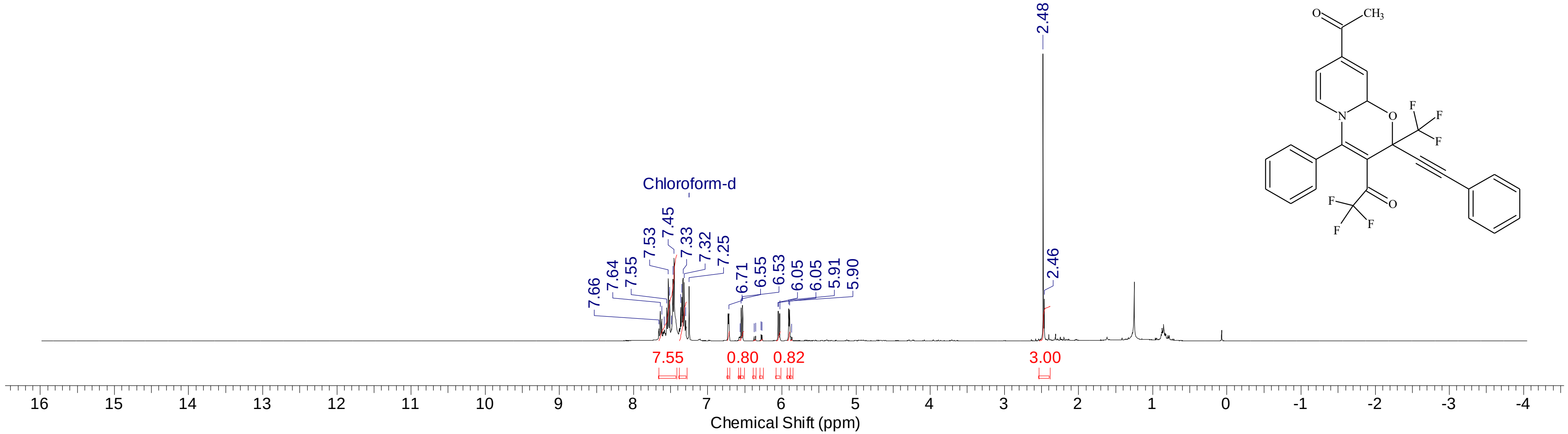

^{1}H NMR spectrum of **3l** (400.1 MHz, $CDCl_3$)

29 Aug 2019

Acquisition Time (sec)	0.6783	Comment	Imported from UXNMR.			Date	22 Jan 2019 14:29:58
File Name	C:\DOCS\OUTPUT_301\2019\01.ÿí âàðü\SZA-048-1.C_002001r					Frequency (MHz)	100.61
Nucleus	13C	Number of Transients	321	Original Points Count	16384	Points Count	131072
Pulse Sequence	zgpg30	Solvent	DMSO-D6	Sweep Width (Hz)	24154.59	Temperature (degree C)	27.000

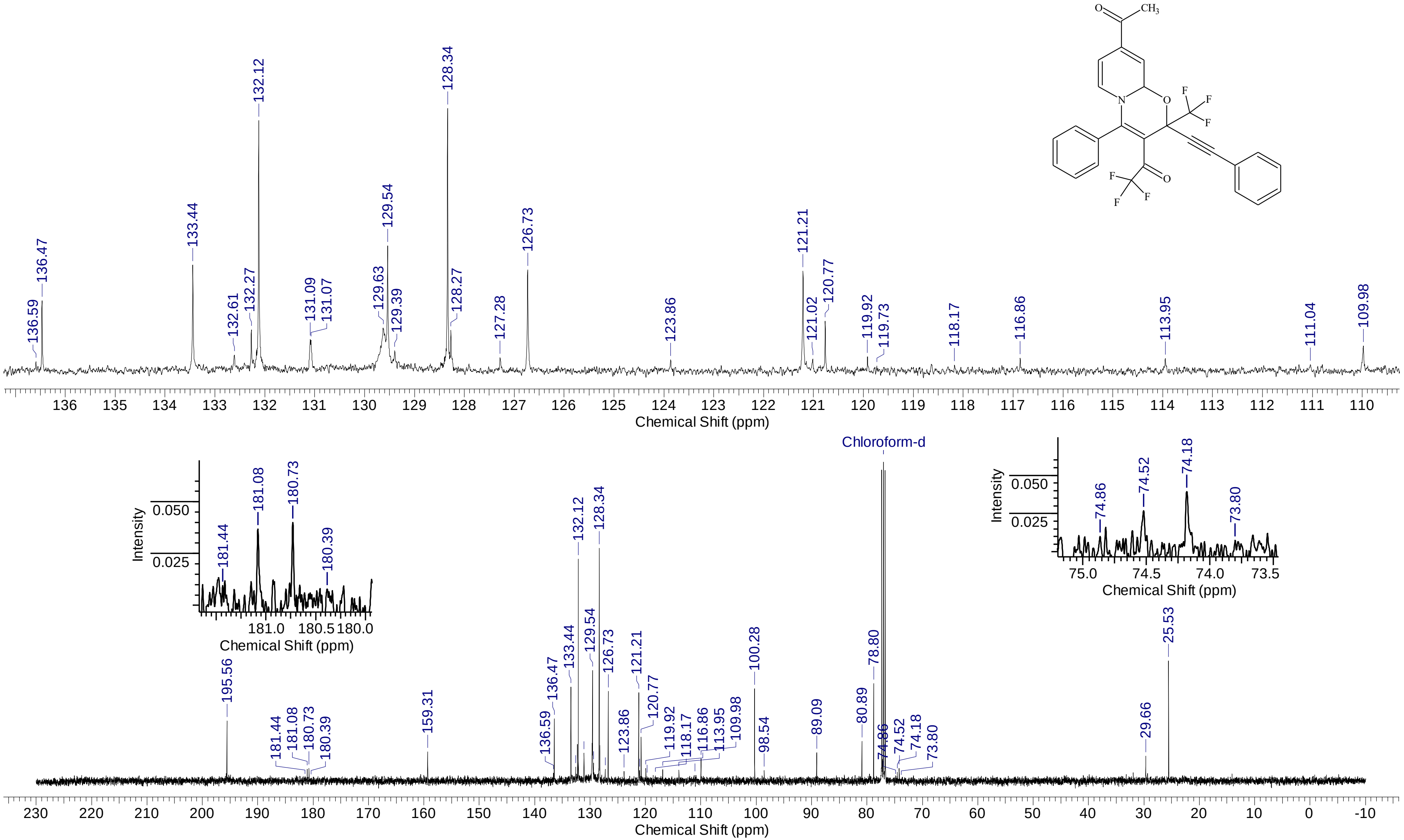

^{13}C NMR spectrum of **3l** (100.6 MHz, $CDCl_3$)

29 Aug 2019

Acquisition Time (sec)	1.5000	Date	Jan 21 2019	File Name	F:\COMP_PRAK\DOCS\OUTPUT_301\F19\2019.01.21\SZA-048_20190121_01\FLUORINE_01		
Frequency (MHz)	376.31	Nucleus	19F	Number of Transients	16	Original Points Count	133929
Points Count	262144	Pulse Sequence	s2pul	Solvent	CHLOROFORM-D		
Sweep Width (Hz)	89285.71	Temperature (degree C)	22.000				

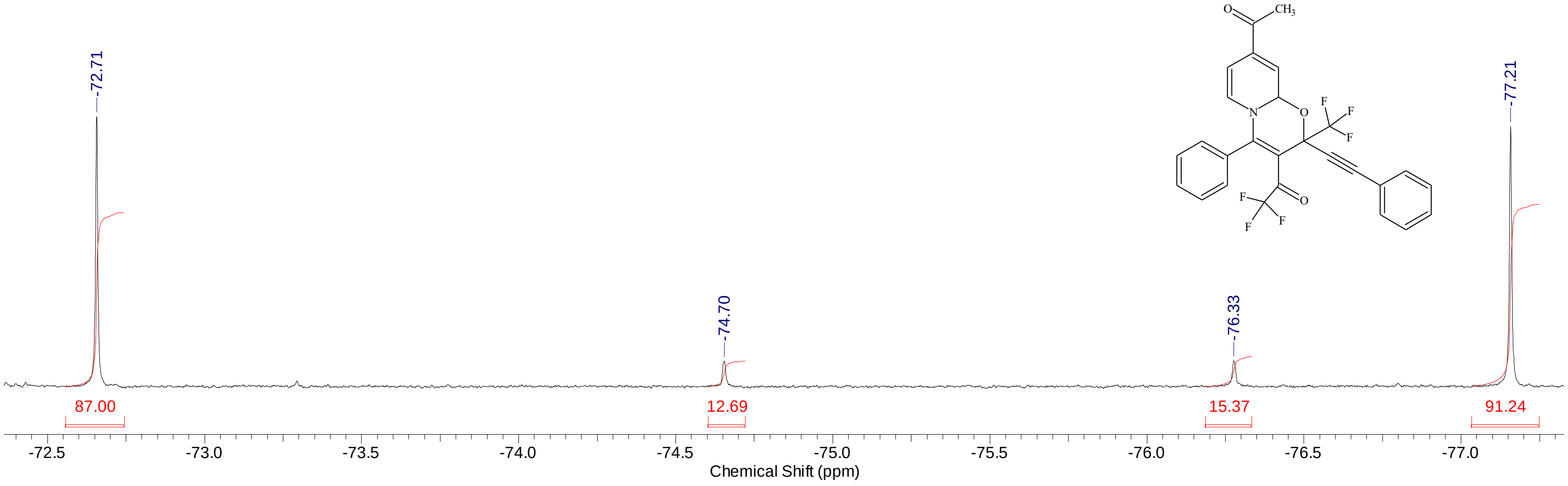

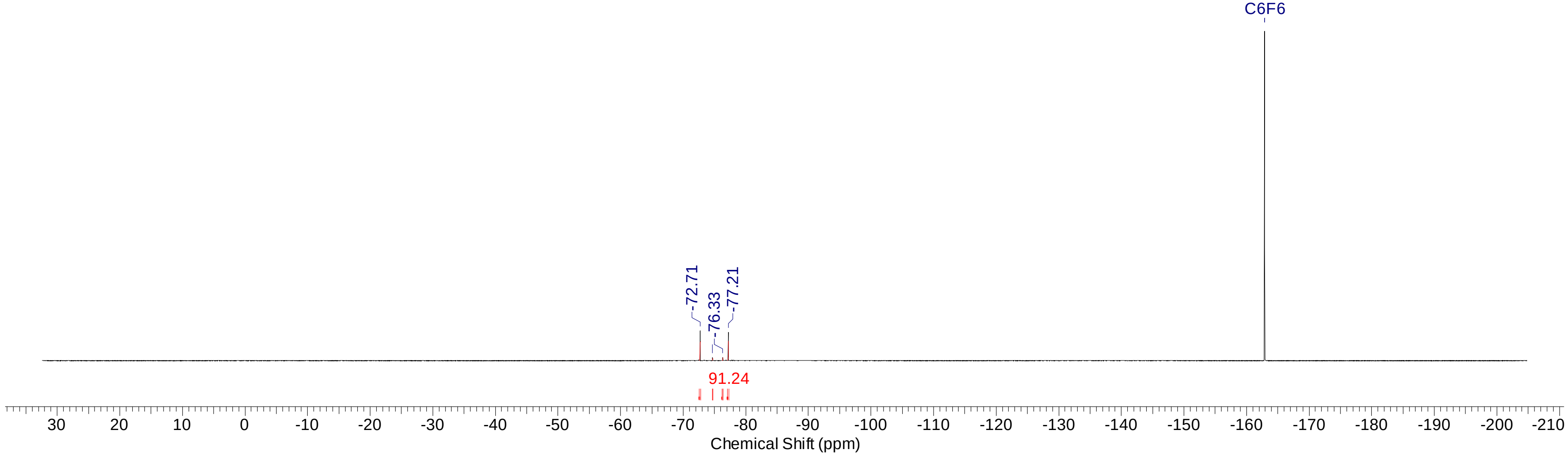

^{19}F NMR spectrum of **3l** (376.3 MHz, $CDCl_3$)

29 Aug 2019

Acquisition Time (sec)	4.0894	***Comment***	Imported from UXNMR.			***Date***	22 Jan 2019 15:30:22
File Name	I:\SPEC_2019_H,C\01.ÿí âàðü\SZA-042-2-1.H_001001r			***Frequency (MHz)***	400.13	***Nucleus***	1H
Number of Transients	4	***Original Points Count***	32768	***Points Count***	131072	***Pulse Sequence***	zg30
Solvent	CHLOROFORM-D			***Sweep Width (Hz)***	8012.82	***Temperature (degree C)***	27.000

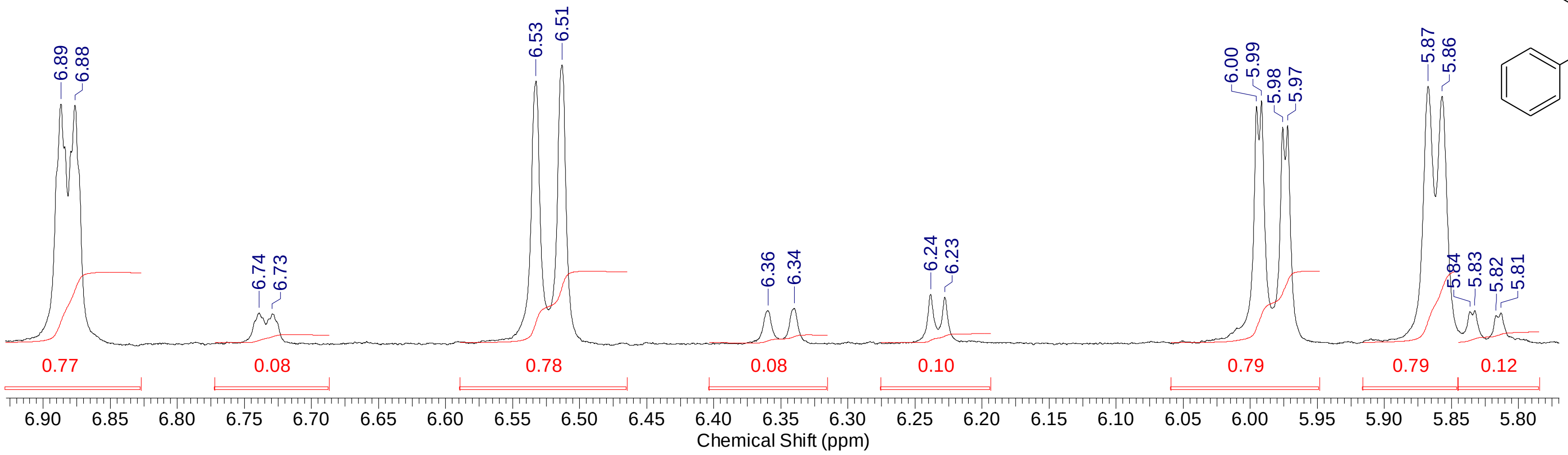

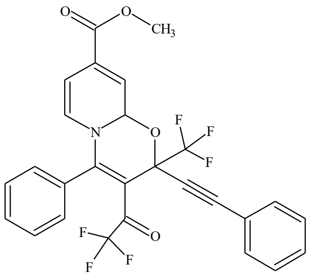

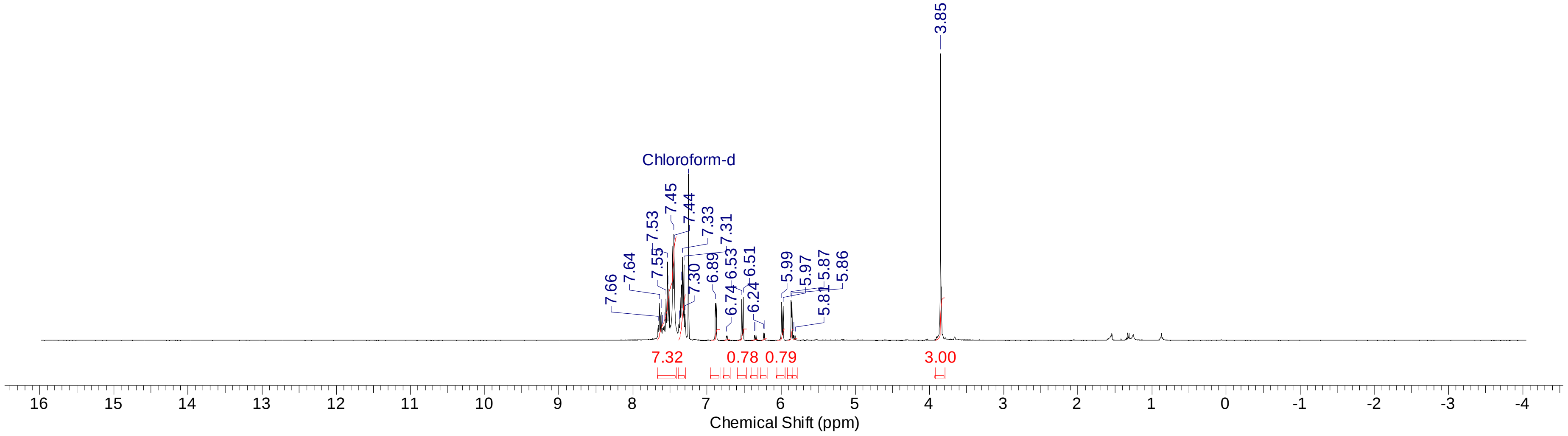

^{1}H NMR spectrum of **3m** (400.1 MHz, $CDCl_3$)

29 Aug 2019

Acquisition Time (sec)	0.6783	Comment	Imported from UXNMR.			Date	24 Jan 2019 12:36:52
File Name	I:\SPEC_2019_H,C\01.ÿí âàðü\SZA-042-2-1.C_002001r			Frequency (MHz)	100.61	Nucleus	13C
Number of Transients	1040	Original Points Count	16384	Points Count	131072	Pulse Sequence	zgpg30
Solvent	DMSO-D6	Sweep Width (Hz)	24154.59	Temperature (degree C)	27.000		

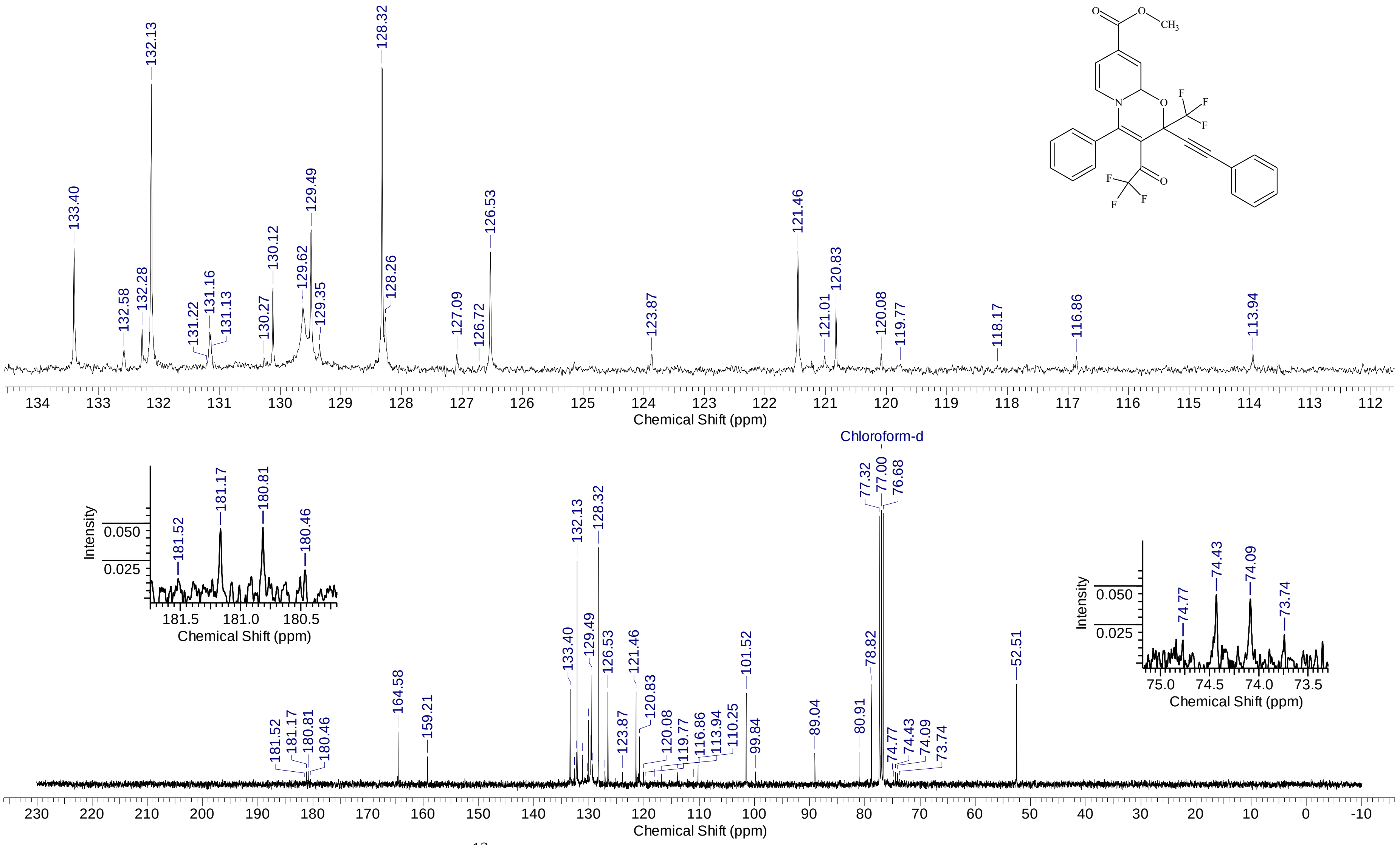

^{13}C NMR spectrum of **3m** (100.6 MHz, $CDCl_3$)

29 Aug 2019

Acquisition Time (sec)	1.5000	Date	Jan 21 2019	File Name	F:\COMP_PRAK\DOCS\OUTPUT_301\F19\2019.01.21\SZA-042-2_20190121_01\FLUORINE_01		
Frequency (MHz)	376.31	Nucleus	19F	Number of Transients	16	Original Points Count	133929
Points Count	262144	Pulse Sequence	s2pul	Solvent	CHLOROFORM-D		
Sweep Width (Hz)	89285.71	Temperature (degree C)	22.000				

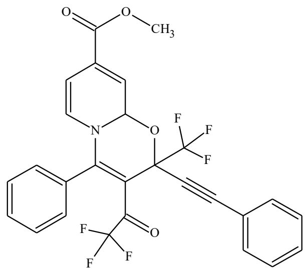

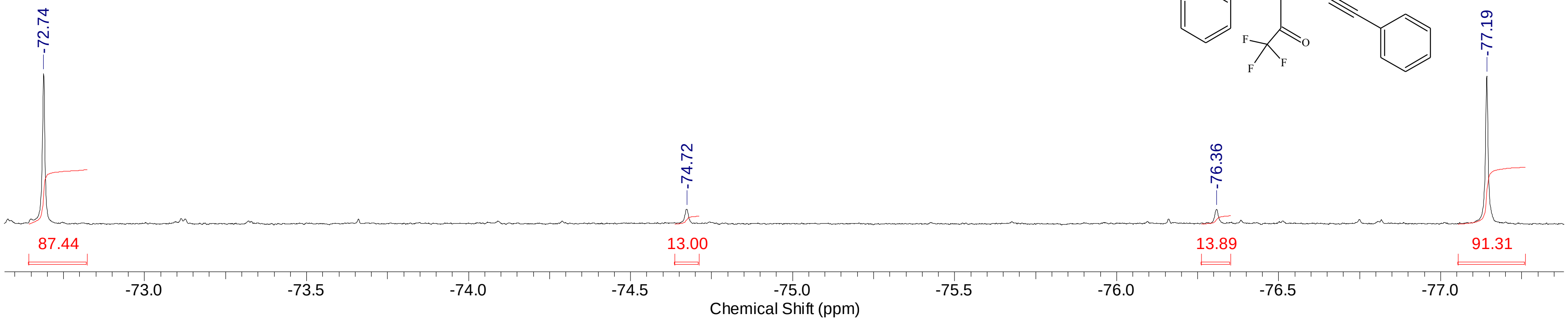

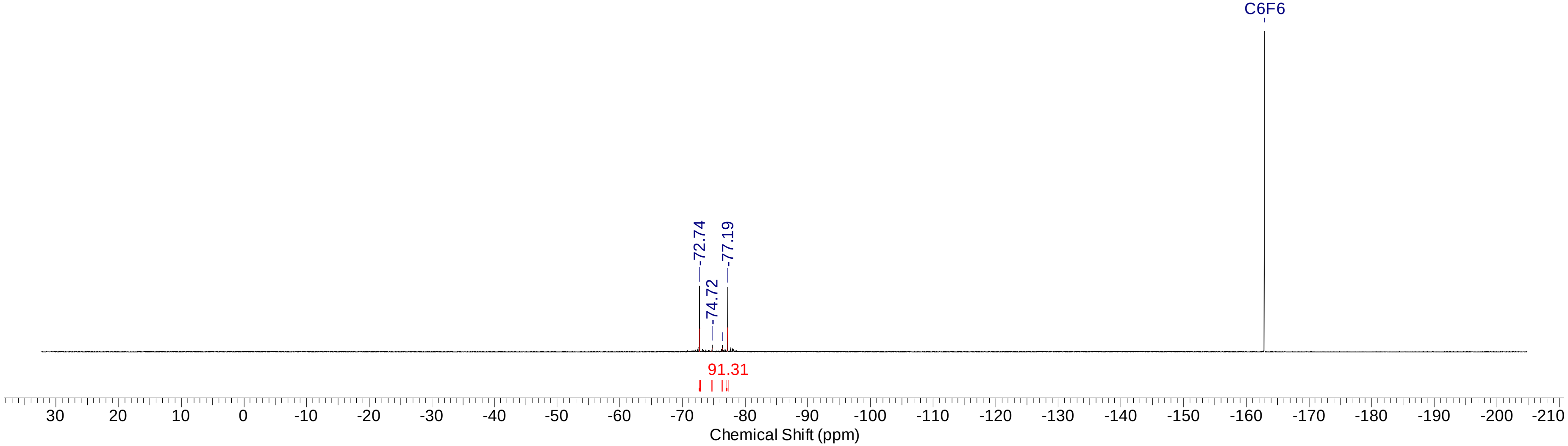

^{19}F NMR spectrum of **3m** (376.3 MHz, $CDCl_3$)

Acquisition Time (sec)	4.0894	*Comment*	Imported from UXNMR.			*Date*	29 Mar 2019 15:16:56
File Name	I:\SPEC_2019_H,C\03.ì àðò\SZA-094.H_001001r			*Frequency (MHz)*	400.13	*Nucleus*	1H
Original Points Count	32768	*Points Count*	131072	*Pulse Sequence*	zg30	*Solvent*	CHLOROFORM-D
Sweep Width (Hz)	8012.82	*Temperature (degree C)*	27.000				

Number of Transients 4

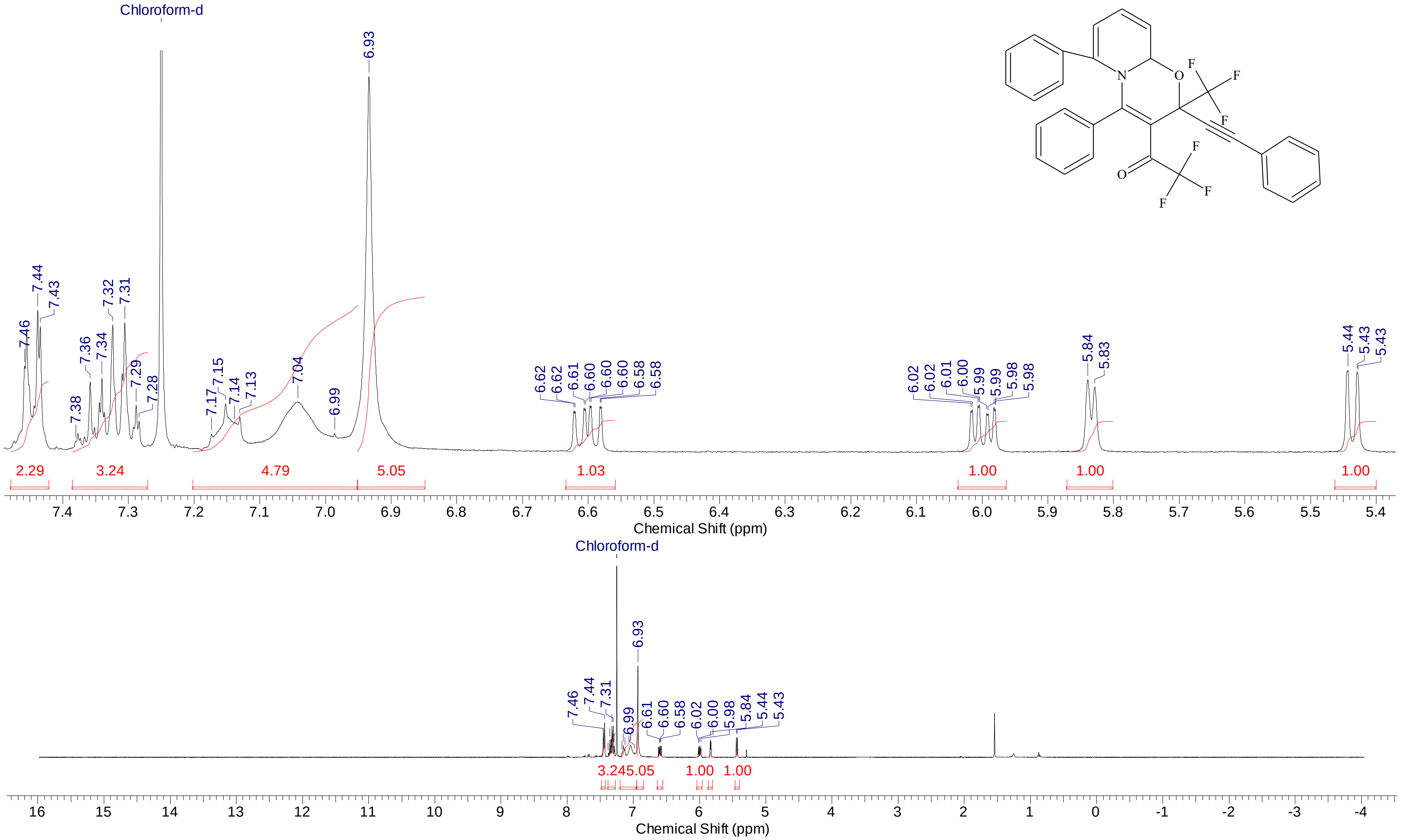

^{1}H NMR spectrum of **3n** (400.1 MHz, $CDCl_3$)

28 Aug 2019

Acquisition Time (sec)	0.6783	Comment	Imported from UXNMR.			Date	02 Apr 2019 14:51:28
File Name	I:\SPEC_2019_H,C\04.àï ðâëü\SZA-094.C_002001r			Frequency (MHz)	100.61	Nucleus	13C
Number of Transients	562	Original Points Count	16384	Points Count	131072	Pulse Sequence	zgpg30
Solvent	CHLOROFORM-D			Sweep Width (Hz)	24154.59	Temperature (degree C)	27.000

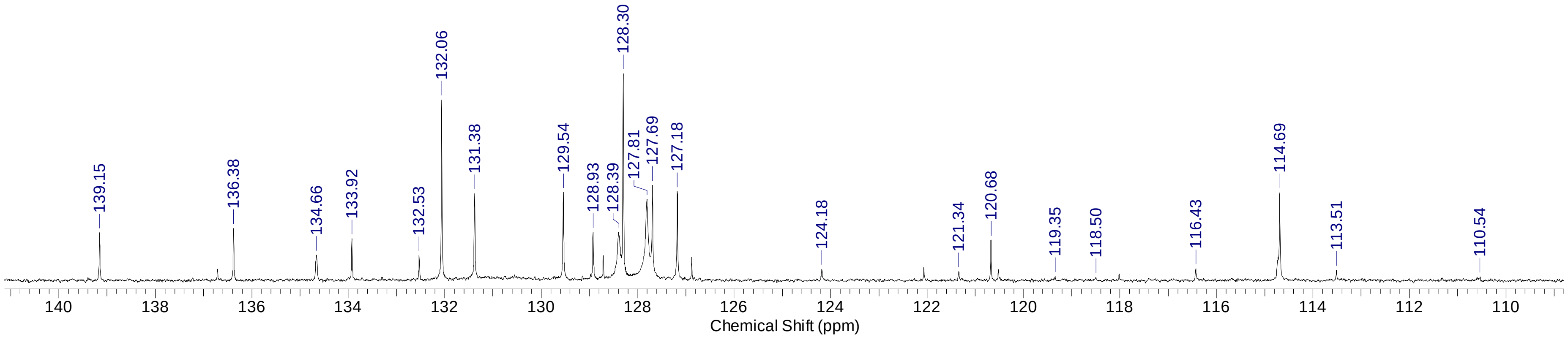

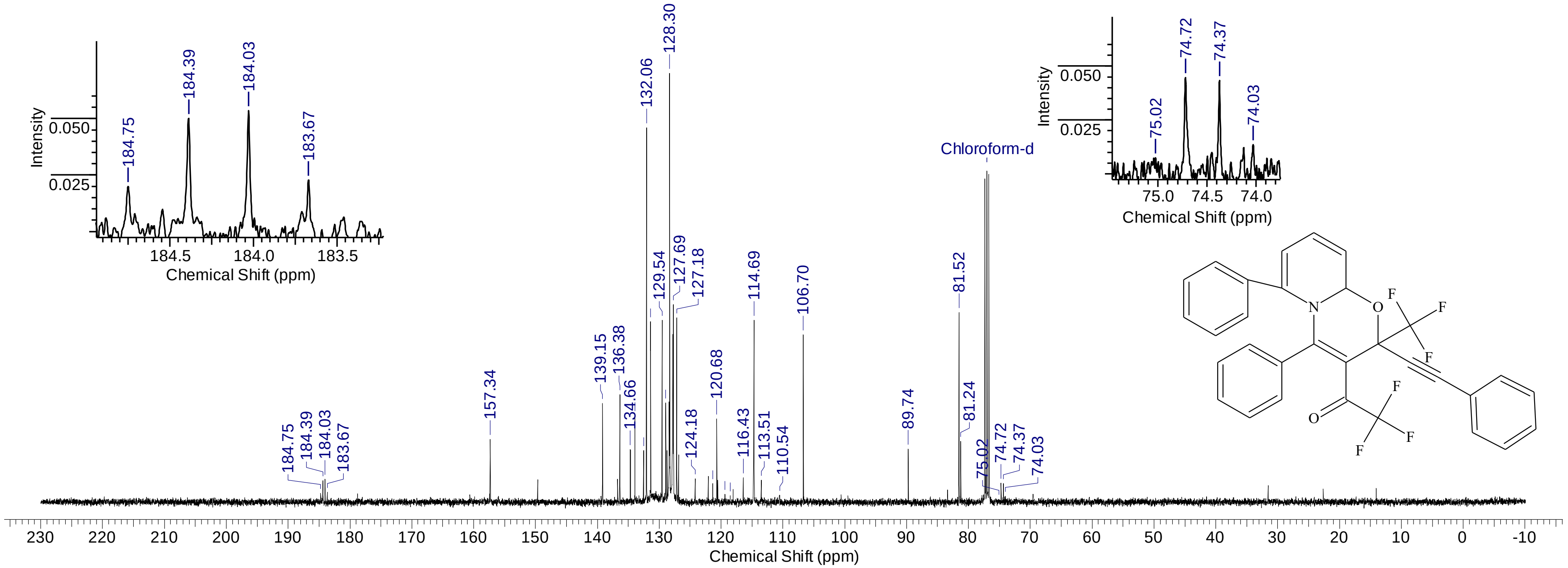

^{13}C NMR spectrum of **3n** (100.6 MHz, $CDCl_3$)

28 Aug 2019

Acquisition Time (sec)	1.5000	Date	Apr 15 2019	File Name	I:\SPEC_F_2019\2019.04.15\SZA-094k_20190415_01\FLUORINE_01		
Frequency (MHz)	376.31	Nucleus	19F	Number of Transients	4	Original Points Count	133929
Points Count	262144	Pulse Sequence	s2pul	Solvent	CHLOROFORM-D		
Sweep Width (Hz)	89285.71	Temperature (degree C)	22.000				

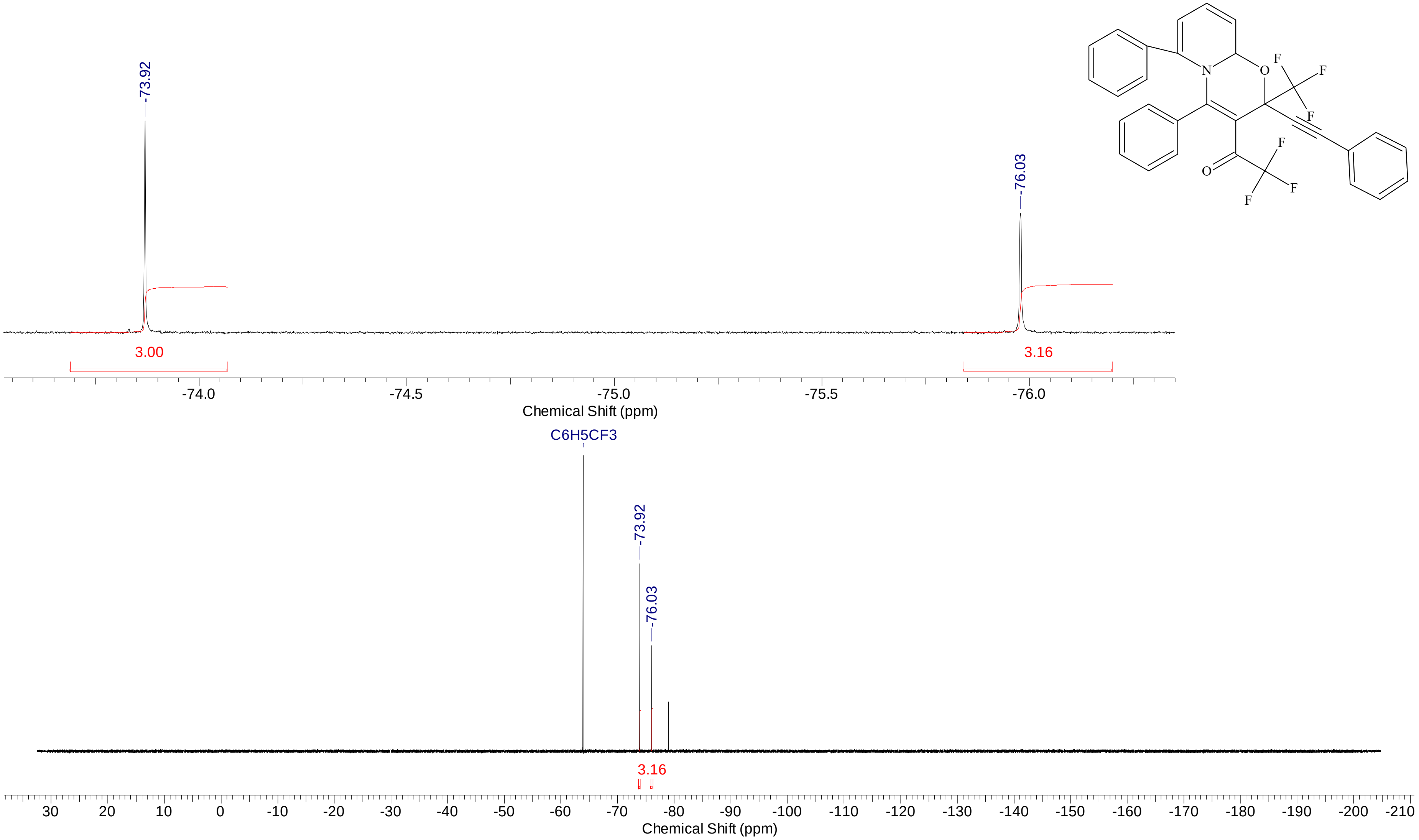

^{19}F NMR spectrum of **3n** (376.3 MHz, $CDCl_3$)

9 Sep 2019

Acquisition Time (sec)	4.0894	***Comment***	Imported from UXNMR.			***Date***	21 Feb 2019 12:43:54
File Name	I:\SPEC_2019_H,C\02.ô âãðàëü\SZA-035-3k.H_001001r			***Frequency (MHz)***	400.13	***Nucleus***	1H
Number of Transients	5	***Original Points Count***	32768	***Points Count***	131072	***Pulse Sequence***	zg30
Solvent	CHLOROFORM-D			***Sweep Width (Hz)***	8012.82	***Temperature (degree C)***	27.000

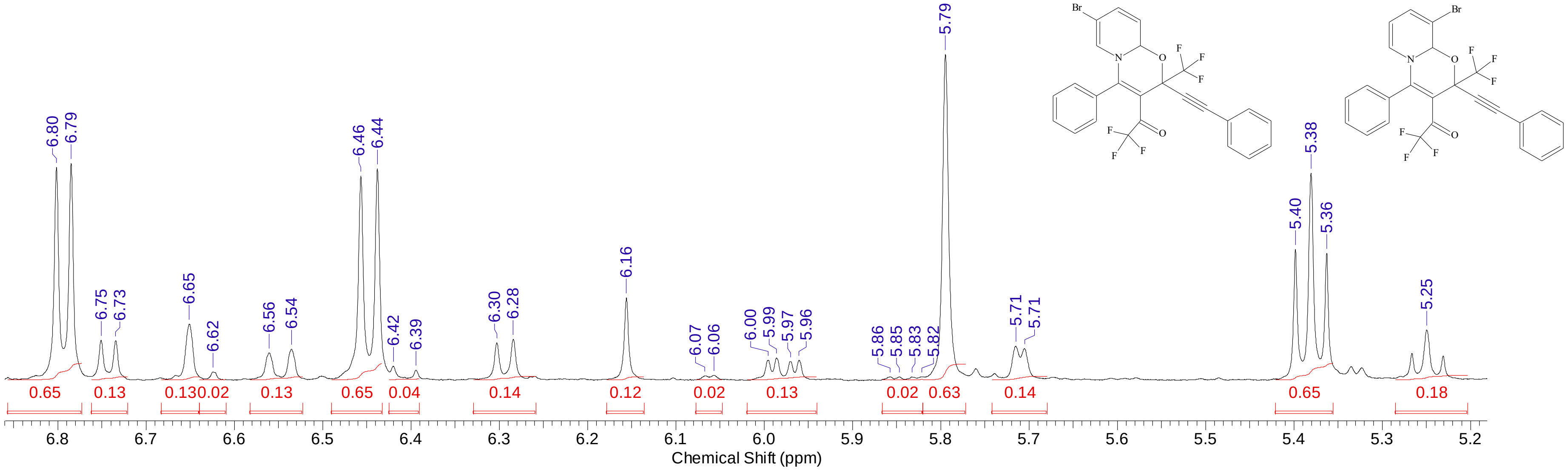

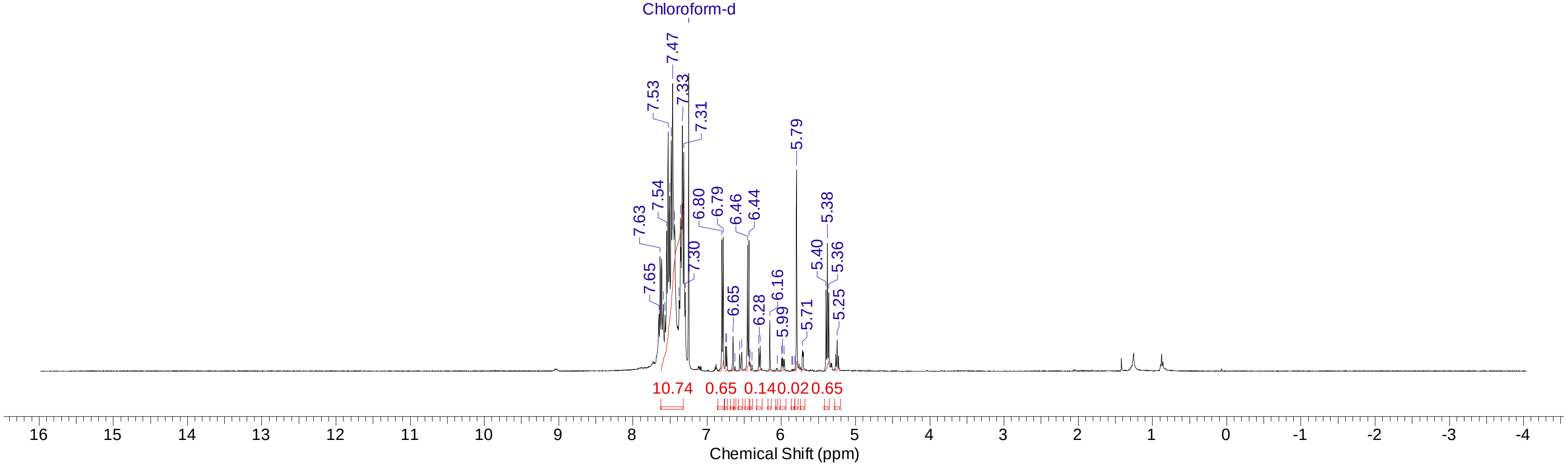

^{1}H NMR spectrum of **3o** and **3p** (400.1 MHz, $CDCl_3$)

Acquisition Time (sec)	0.6783	***Comment***	Imported from UXNMR.			***Date***	21 Feb 2019 14:02:20
File Name	F:\COMP_PRAK\DOCS\OUTPUT_301\2019\02.ô åâðàëü\SZA-035-3k.C_002001r					***Frequency (MHz)***	100.61
Nucleus	13C	***Number of Transients***	2074	***Original Points Count***	16384	***Points Count***	131072
Pulse Sequence	zgpg30	***Solvent***	DMSO-D6	***Sweep Width (Hz)***	24154.59	***Temperature (degree C)***	27.000

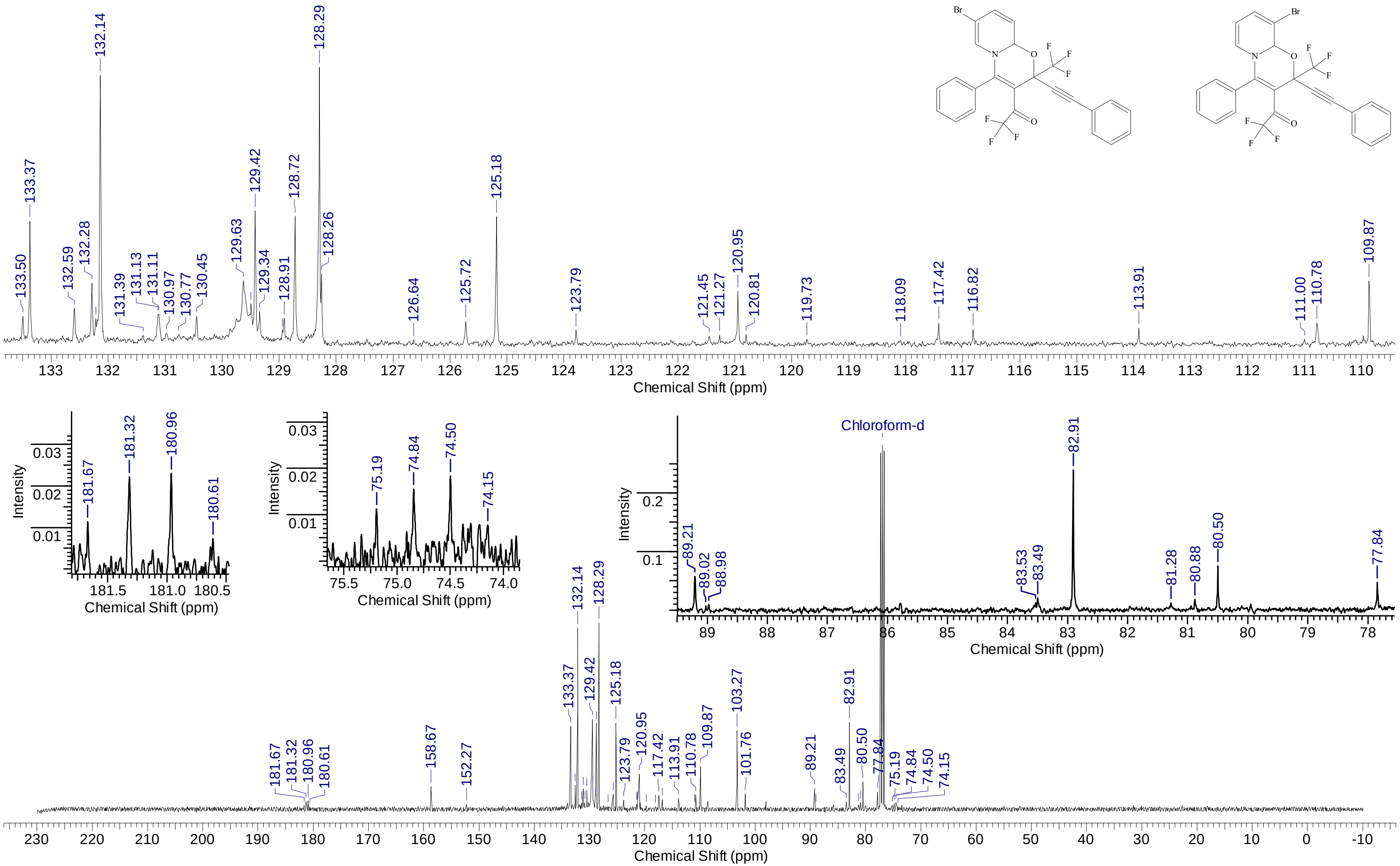

^{13}C NMR spectrum of **3o** and **3p** (100.6 MHz, $CDCl_3$)

9 Sep 2019

Acquisition Time (sec)	1.0000	Date	Feb 25 2019	File Name	I:\SPEC_F_2019\2019.02.25\SZA-035-3k_20190225_01\FLUORINE_01		
Frequency (MHz)	376.31	Nucleus	19F	Number of Transients	16	Original Points Count	89286
Points Count	131072	Pulse Sequence	s2pul	Solvent	CHLOROFORM-D		
Sweep Width (Hz)	89285.71	Temperature (degree C)	24.000				

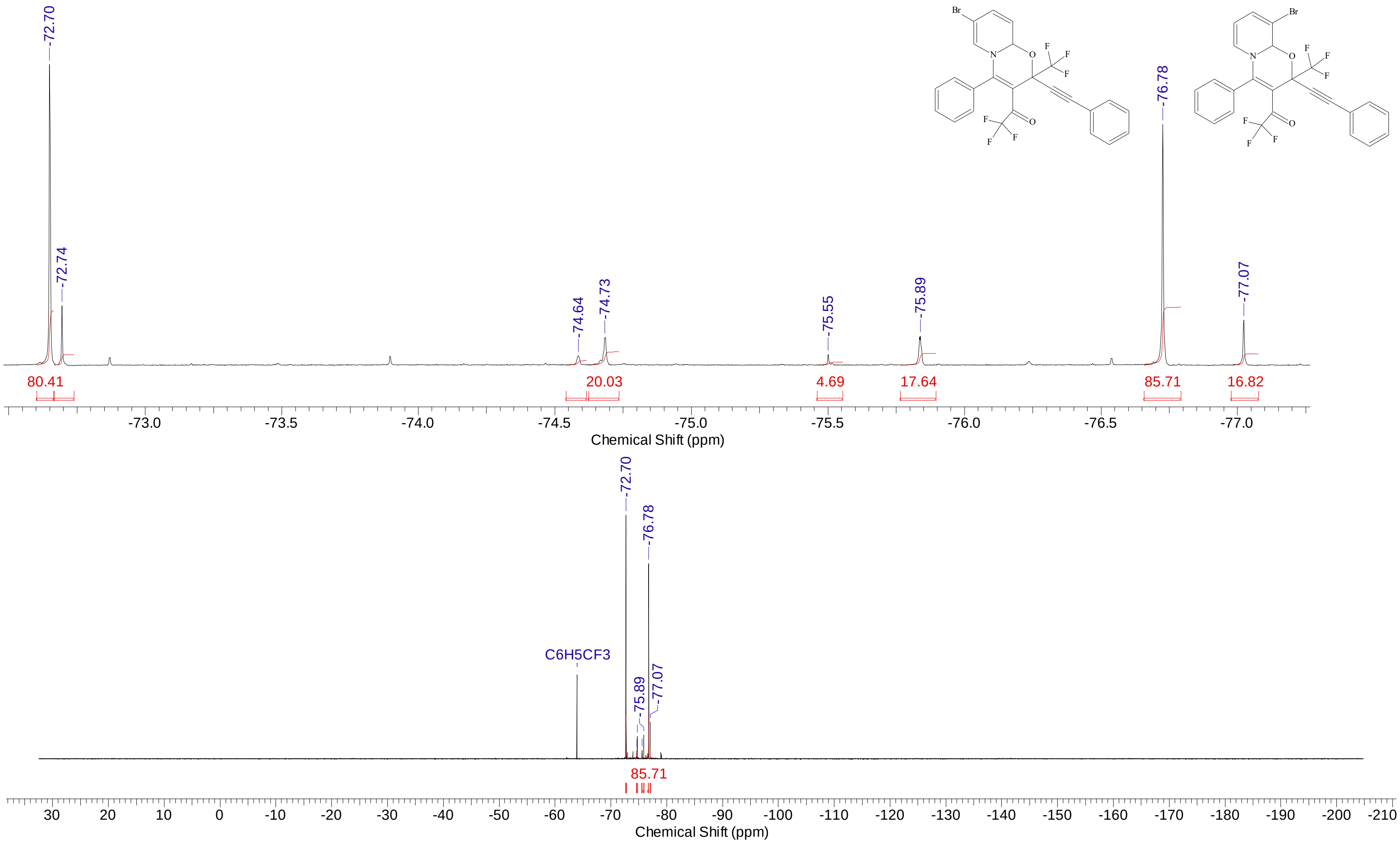

^{19}F NMR spectrum of **3o** and **3p** (376.3 MHz, $CDCl_3$)

31 Aug 2019

Acquisition Time (sec)	4.0894	***Comment***	Imported from UXNMR.	***Date***	04 Feb 2019 15:11:38
File Name	F:\COMP_PRAK\DOCS\OUTPUT_301\2019\02.ô åâðàëü\SZA-047-3.H_001001r			***Frequency (MHz)***	400.13
Nucleus	1H	***Number of Transients***	4 ***Original Points Count*** 32768	***Points Count***	131072
Pulse Sequence	zg30	***Solvent***	CHLOROFORM-D	***Sweep Width (Hz)***	8012.82
Temperature (degree C)	27.000				

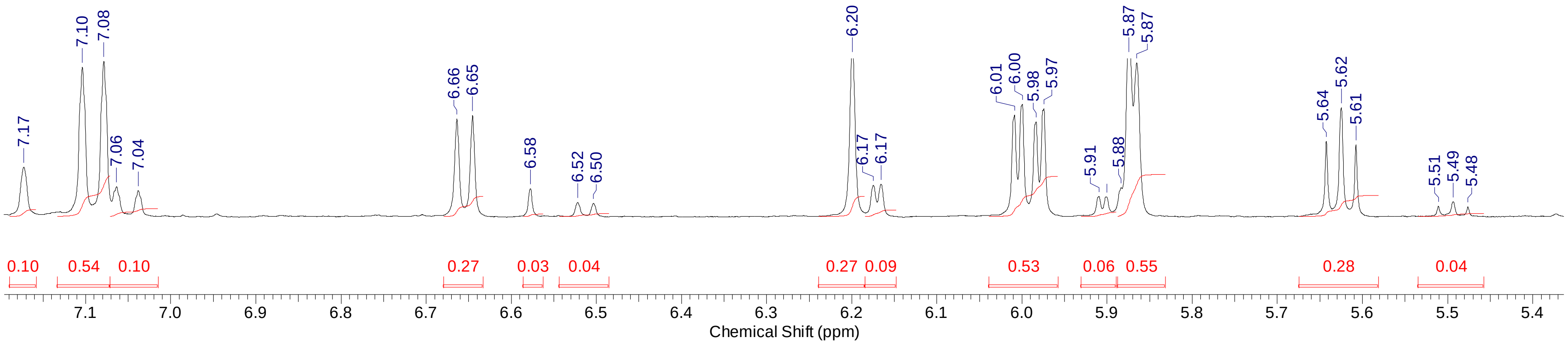

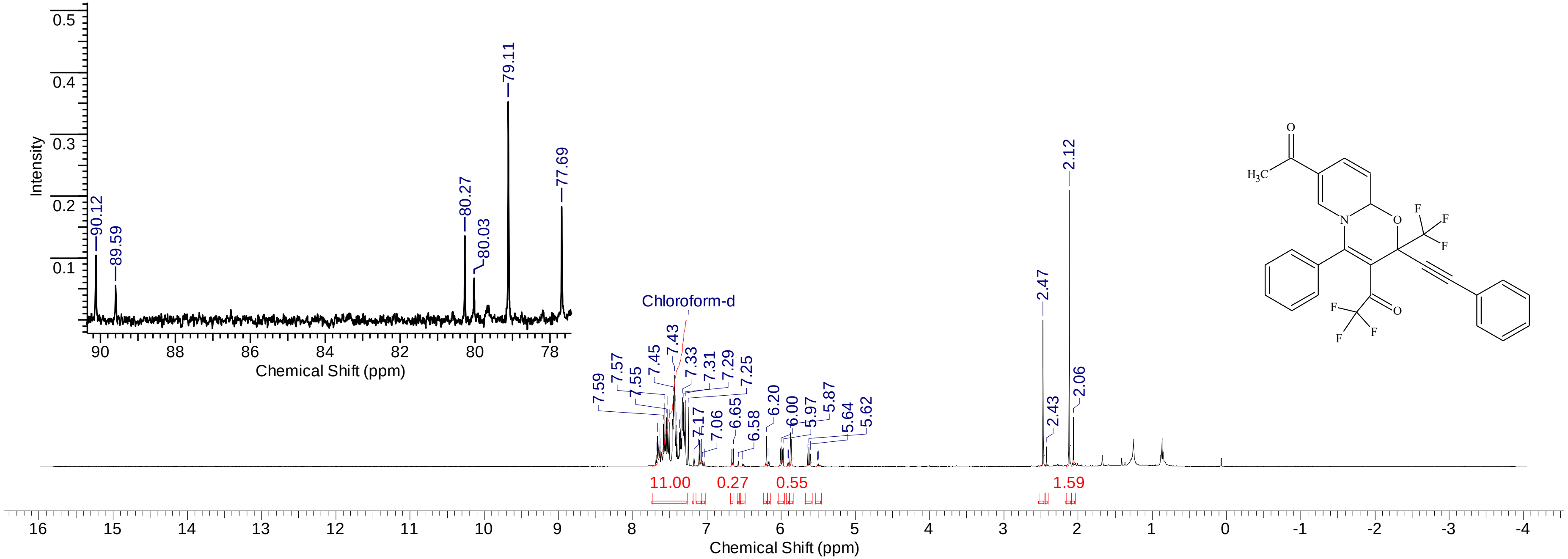

^{1}H NMR spectrum of **3q** and **3r** (400.1 MHz, $CDCl_3$)

Acquisition Time (sec)	0.6783	***Comment***	Imported from UXNMR.			***Date***	04 Feb 2019 15:30:44
File Name	I:\SPEC_2019_H,C\02.ô âãðàëü\SZA-047-3.C_002001r			***Frequency (MHz)***	100.61	***Nucleus***	13C
Number of Transients	585	***Original Points Count***	16384	***Points Count***	131072	***Pulse Sequence***	zgpg30
Solvent	DMSO-D6	***Sweep Width (Hz)***	24154.59	***Temperature (degree C)***	27.000		

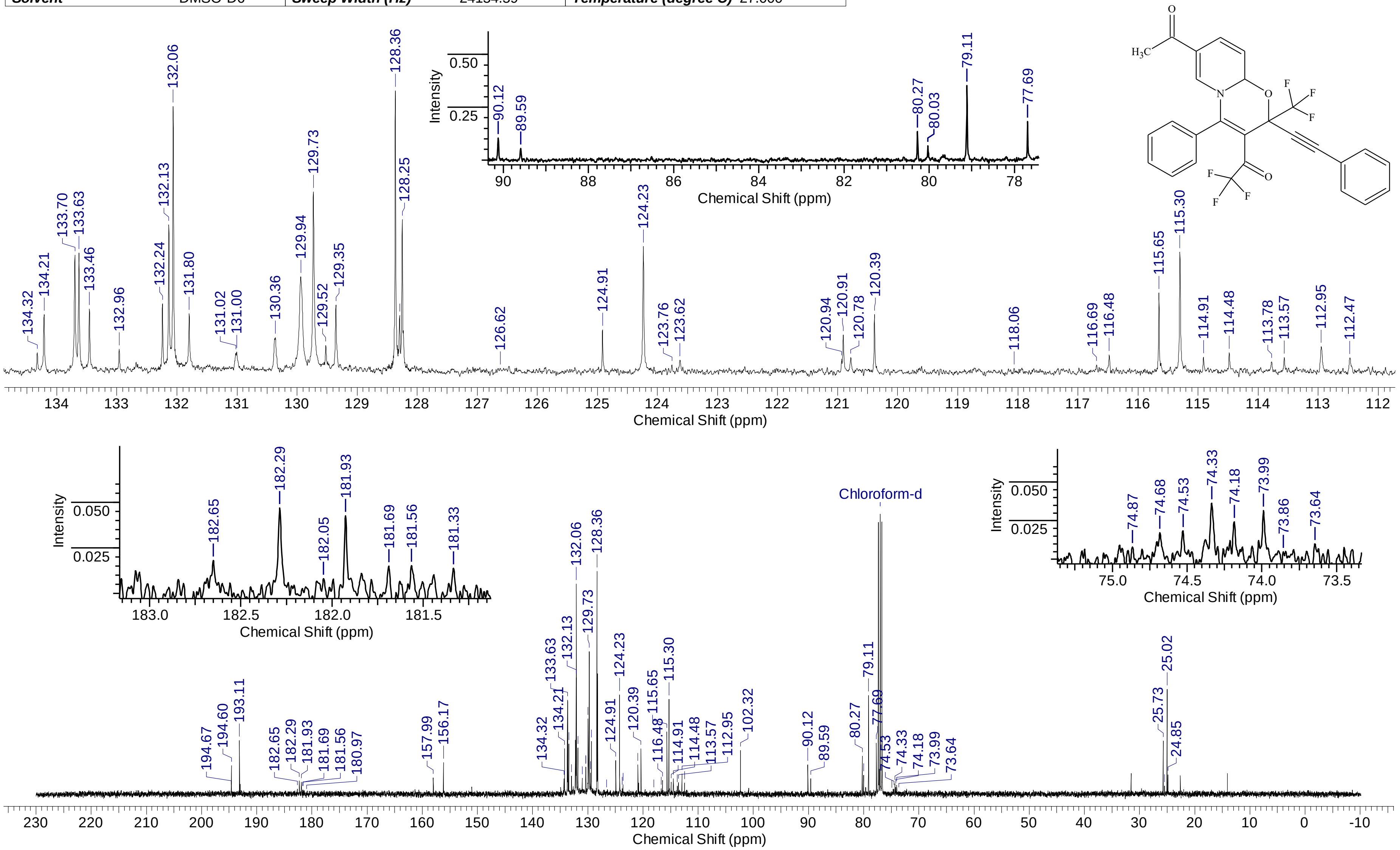

^{13}C NMR spectrum of **3q** and **3r** (100.6 MHz, $CDCl_3$)

31 Aug 2019

Acquisition Time (sec)	1.0000	***Date***	Feb 1 2019				
File Name	F:\COMP_PRAK\DOCS\OUTPUT_301\F19\2019.02.01\SZA-047-31_20190201_01\FLUORINE_01					***Frequency (MHz)***	376.31
Nucleus	19F	***Number of Transients***	16	***Original Points Count***	89286	***Points Count***	131072
Pulse Sequence	s2pul	***Solvent***	CHLOROFORM-D			***Sweep Width (Hz)***	89285.71
Temperature (degree C)	22.000						

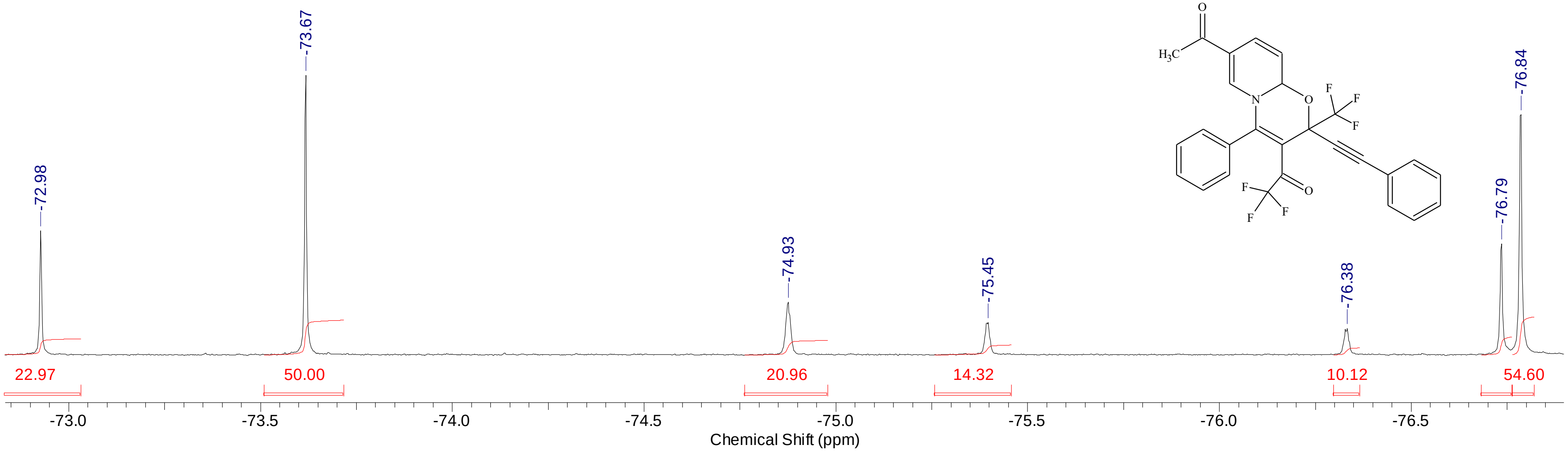

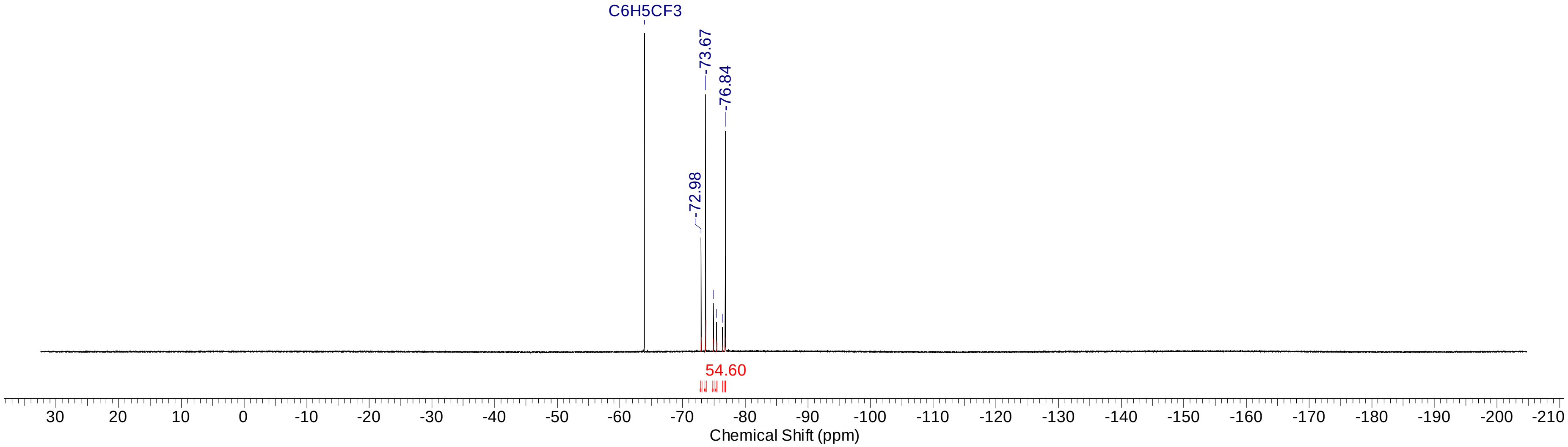

^{19}F NMR spectrum of **3q** and **3r** (376.3 MHz, $CDCl_3$)

8 Sep 2019

**Acquisition Time (sec)**	4.0894	**Comment**	Imported from UXNMR.			**Date**	11 Apr 2019 12:19:14
**File Name**	I:\SPEC_2019_H,C\04.àï ðâëü\SZA-089.H_001001r			**Frequency (MHz)**	400.13	**Nucleus**	1H
**Number of Transients**	4	**Original Points Count**	32768	**Points Count**	131072	**Pulse Sequence**	zg30
**Solvent**	CHLOROFORM-D			**Sweep Width (Hz)**	8012.82	**Temperature (degree C)**	27.000

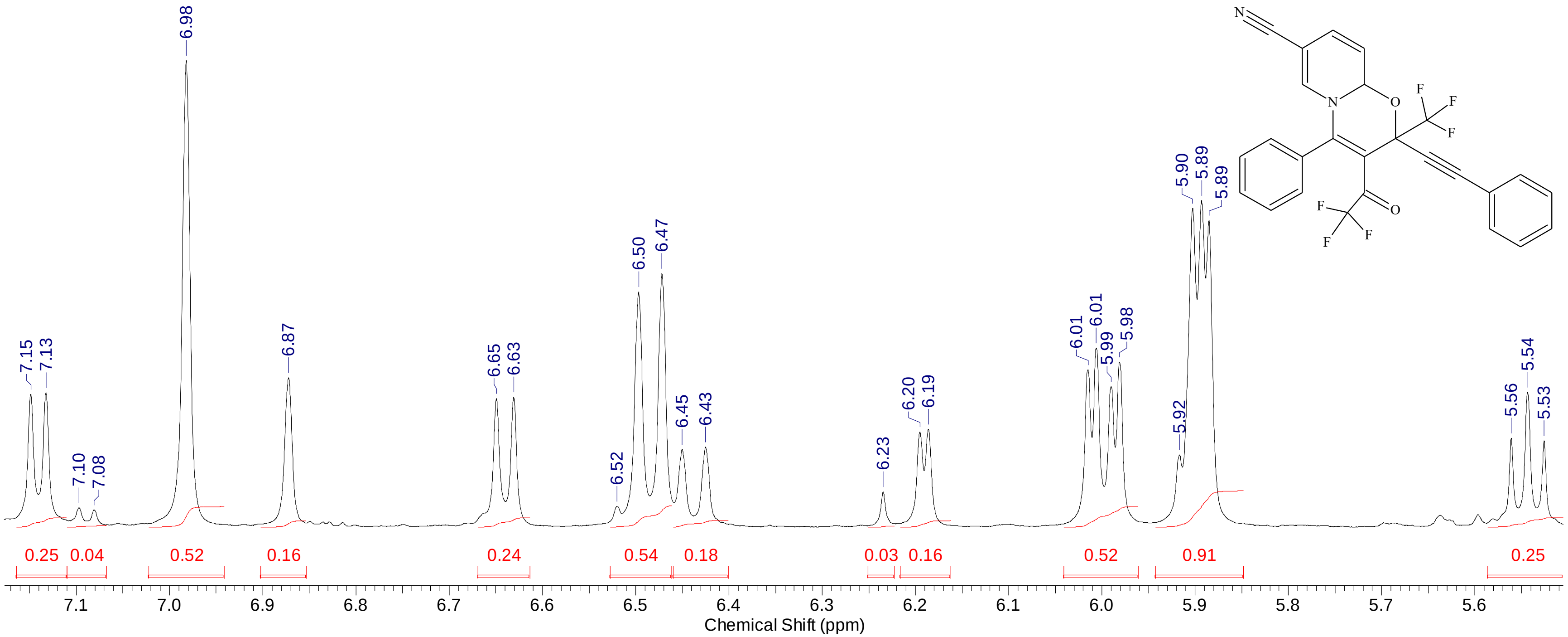

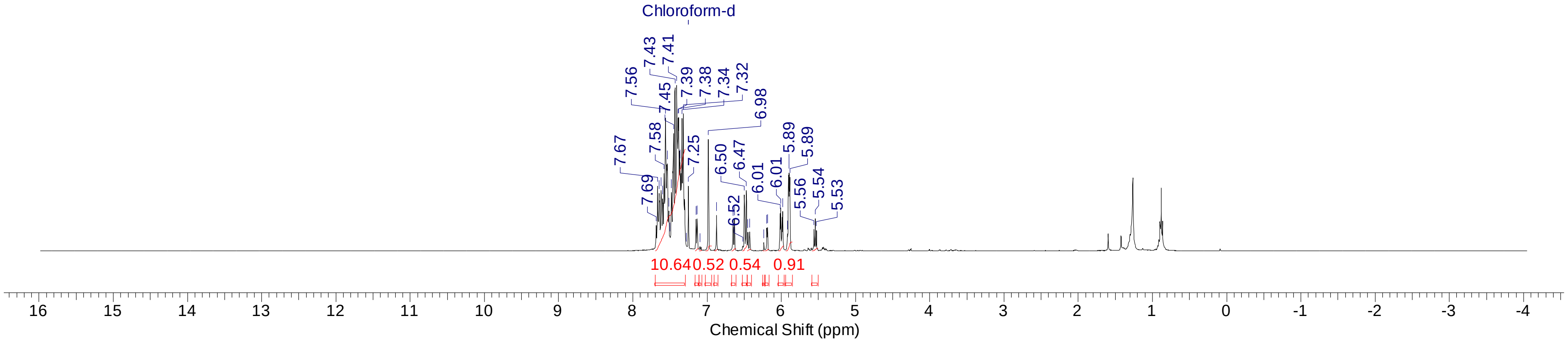

^{1}H NMR spectrum of **3s** and **3t** (400.1 MHz, $CDCl_3$)

8 Sep 2019

Acquisition Time (sec)	0.6783	***Comment***	Imported from UXNMR.			***Date***	11 Apr 2019 12:48:28
File Name	I:\SPEC_2019_H,C\04.àï ðåëü\SZA-089.C_002001r			***Frequency (MHz)***	100.61	***Nucleus***	13C
Number of Transients	746	***Original Points Count***	16384	***Points Count***	131072	***Pulse Sequence***	zgpg30
Solvent	CHLOROFORM-D			***Sweep Width (Hz)***	24154.59	***Temperature (degree C)***	27.000

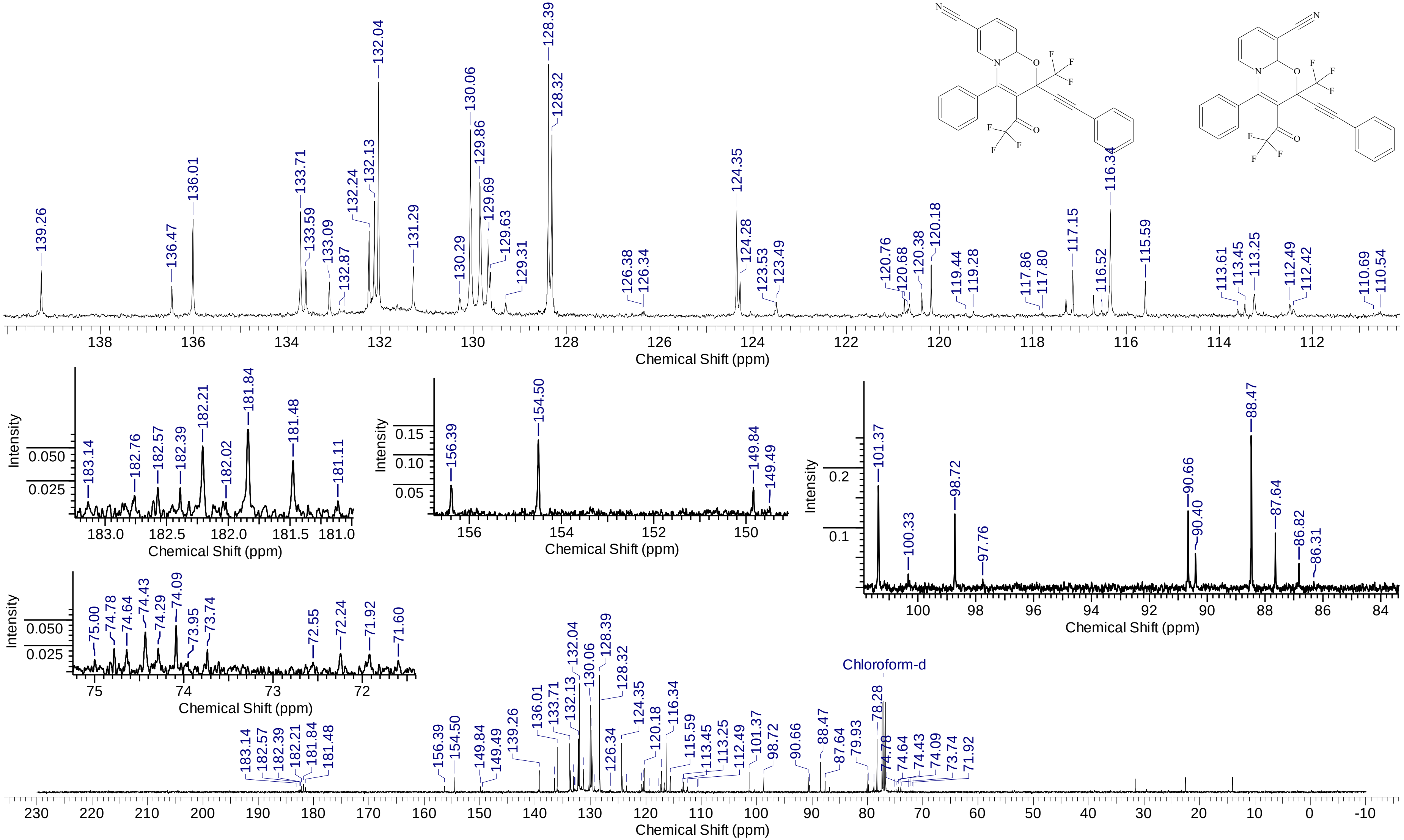

^{13}C NMR spectrum of **3s** and **3t** (100.6 MHz, $CDCl_3$)

8 Sep 2019

Acquisition Time (sec)	2.3069	Date	Apr 11 2019	File Name	I:\SPEC_F_2019\2019.04.11\SZA-089-F_20190411_01\FLUORINE_01		
Frequency (MHz)	376.32	Nucleus	19F	Number of Transients	8	Original Points Count	262144
Points Count	262144	Pulse Sequence	s2pul	Solvent	CHLOROFORM-D		
Sweep Width (Hz)	113636.37	Temperature (degree C)	22.000				

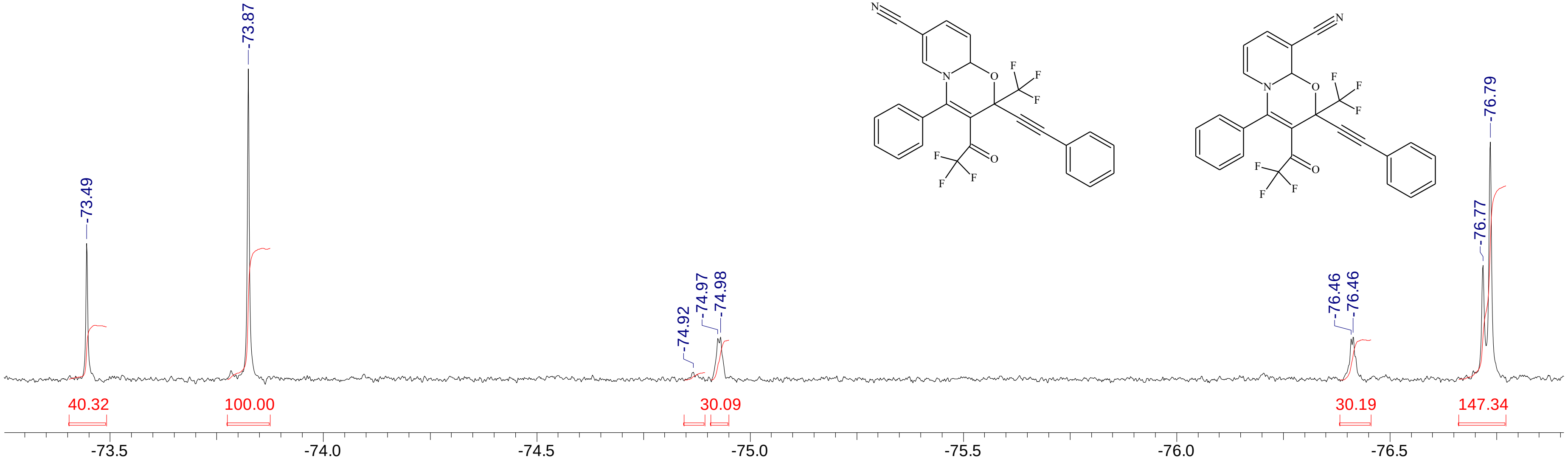

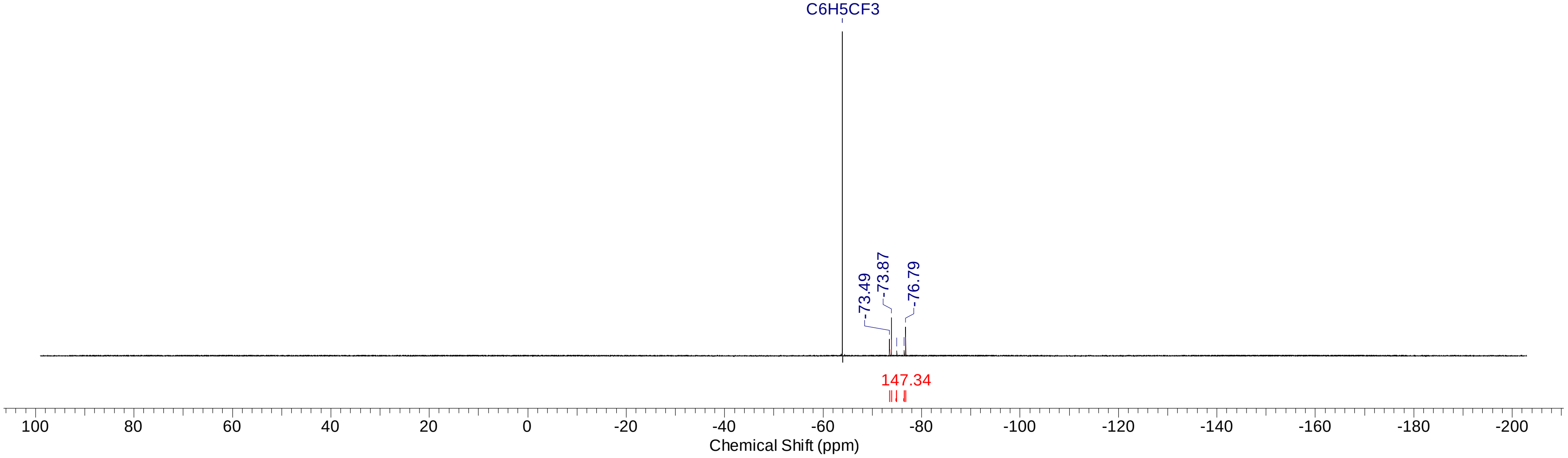

^{19}F NMR spectrum of **3s** and **3t** (376.3 MHz, $CDCl_3$)
